# Selective Adsorption of *o*‐Xylene From all C8 Aromatics via Secondary Connectivity Manipulation in B←N Frameworks

**DOI:** 10.1002/anie.1406923

**Published:** 2026-08-19

**Authors:** Xiang‐Shuai Li, Yizhou Wang, Wing‐Laam Luk, Jieying Hu, Rong Fu, Zhao Zhang, Zenan Chen, Jian Zhao, Wan Chan, Yangjian Quan

**Affiliations:** ^1^ Department of Chemistry The Hong Kong University of Science and Technology (HKUST) Hong Kong SAR China; ^2^ School of Chemical Engineering and Light Industry Guangdong University of Technology Guangzhou China; ^3^ College of Environmental Science and Engineering Ministry of Education Key Laboratory of Pollution Processes and Environmental Criteria Tianjin Key Laboratory of Environmental Remediation and Pollution Control Nankai University Tianjin China; ^4^ Frontiers Science Center for New Organic Matter State Key Laboratory of Advanced Chemical Power Sources Key Laboratory of Advanced Energy Materials Chemistry (Ministry of Education) Haihe Laboratory of Sustainable Chemical Transformations Renewable Energy Conversion and Storage Center College of Chemistry Nankai University Tianjin China; ^5^ Ganjiang Innovation Academy Jiangxi Institute of Rare Earths Chinese Academy of Sciences Ganzhou China

**Keywords:** B←N frameworks, benzene derivative separation, metal‐free framework, secondary connectivity, selective *o*‐xylene adsorption

## Abstract

Separating *o*‐xylene from all C8 aromatic isomers (*m*‐xylene, *p*‐xylene, and ethylbenzene) is critical for industry but remains a formidable challenge due to their quite similar physicochemical properties. While metal‐free frameworks offer an ideal solution via selective adsorption, their development lags behind metal‐based systems. For instance, due to the limited connection modes and lack of suitable linkers, B←N frameworks (BNFs) struggle to handle complex separations. To address this limitation, we report a programmed assembly strategy by rationally integrating secondary connectivity into bridging linkers. Implementing this strategy yielded stable BNFs, **BN‐DPTA1** and **BN‐DPTA2**, featuring tunable and confined cavities. **BN‐DPTA1** exhibits specific abstraction of toluene, while **BN‐DPTA2** selectively adsorbs *o*‐xylene from benzene derivatives. Notably, **BN‐DPTA2** represents the first metal‐free framework to achieve highly selective adsorption of *o*‐xylene over all C8 aromatic isomers. The multiple non‐covalent interactions between the BNF cavities and guest molecules facilitate tight encapsulation, enabling the effective removal of toluene and *o*‐xylene from the atmosphere (97.4% and 96.0% removal efficiency at 50 ppm, respectively). The dynamic, reversible adsorption/desorption behavior of BNFs allows for single‐step purification of toluene and *o*‐xylene via solid‐phase extraction columns, achieving purities exceeding 99.9%.

## Graphical abstract

A programmed assembly strategy based on secondary connectivity integration allows modulating the B←N framework (BNF) microenvironment. Beyond possessing improved stability, the resulting BNFs achieve highly selective adsorption of *o*‐xylene from all C8 aromatics.

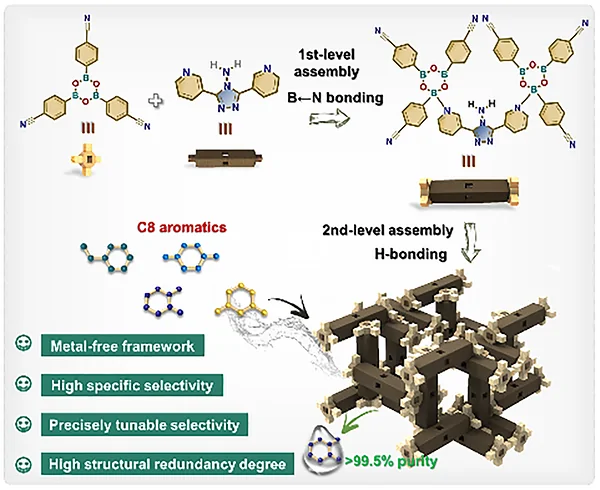

## 1 Introduction

Differentiating benzene derivatives is a long‐standing challenge due to the similarity in their physicochemical properties [1, 2, 3]. However, given their indispensable applications in industry, separating them represents one of the seven chemical separations that could change the world [4]. Indeed, it costs over 50 GW of energy every year to isolate benzene, toluene (Tol), and xylene isomers from the mixture products of the petroleum industry [5, 6, 7, 8, 9, 10, 11, 12, 13, 14, 15, 16, 17]. On the other hand, from a health perspective, benzene derivatives are harmful to human beings, and the permissible exposure limits (PELs) of benzene, Tol, and xylene are set lower than 1, 200, and 100 ppm, respectively [18, 19, 20].

Among benzene derivatives, *o*‐xylene (OX) is of great importance as a key precursor for producing phthalic anhydride, a core component in dyes, pesticides, and pharmaceuticals. Despite its importance, achieving an efficient and cost‐effective OX separation from all C8 aromatics remains a formidable challenge. While rationally designed metal–organic frameworks (MOFs) have advanced OX adsorption [21, 22, 23, 24, 25, 26, 27, 28, 29, 30, 31, 32, 33, 34], the development of metal‐free alternatives lags. This disparity stems from two main limitations: (1) metal‐free matrices lack host–guest active sites (e.g., open metal centers) to bind OX molecules; and (2) their relatively lower structural crystallinity reduces the adsorption selectivity among structurally similar isomers.

B←N frameworks (BNFs) are an emerging class of crystalline porous materials constructed according to dative boron–nitrogen (B←N) bonds [35, 36, 37, 38, 39, 40, 41, 42], providing a metal‐free platform for manipulating adsorption behaviors [43, 44, 45, 46, 47, 48, 49]. Severin reported the first synthesis of molecular networks via B←N coordination [46]. In 2023, Zhang et al. pioneered the research of microporous BNFs for effective C_2_H_2_/CO_2_ separation [37]. Subsequently, F. Huang [44], Q. Zhang [41], H.‐L. Jiang [48], and J. Zhang [43] groups further expanded the structural space of BNFs with promising applications. Specific to separation, beyond recognizing guest molecules through multiple non‐covalent interactions, BNFs can tightly trap specified guests within their cavities via co‐crystallization [49, 50]. Despite significant achievements, B←N dative bonds dominate the core structures of previously reported BNFs. This relatively weak dative bonding likely limits both structural diversity and stability of the resulting frameworks, thereby hindering their potential applications in complex separation tasks [51, 52, 53, 54, 55, 56, 57, 58, 59, 60]. Inspired by the principle of mortise‐and‐tenon jointing, where multiple relatively weak interactions collectively enable the construction of stable and flexible architectures with a high degree of redundancy [61, 62], we hypothesized that introducing secondary connectivity into BNF formation would not only enhance the stability of the resulting porous materials through extra interlockings, but also create confined host microenvironments by additional oriented connections. Such a foundational design would advance their performance in complex chemical separations.

Taking the above two aspects into consideration, we herein report the design of BNF crystalline sponges **BN‐DPTA1** and **BN‐DPTA2** featuring 4‐amino‐3,5‐di(4‐pyridyl)‐1,2,4‐triazole (DPTA1) and 4‐amino‐3,5‐di(3‐pyridyl)‐1,2,4‐triazole (DPTA2) multifunctional linkers, respectively (Figure 1). In the first‐level assembly, terminal pyridinyl units of DPTA form dative bonds with the boroxine building block 4,4′,4″‐(1,3,5,2,4,6‐trioxatriborinane‐2,4,6‐triyl)tribenzonitrile (TBTBN). Afterwards, the middle amino‐triazole secondary junction permits additional N─H⋯N≡C hydrogen bonding with the TBTBN part. This programmed assembly leads to an interlocking porous structure, mimicking an artificial mortise‐and‐tenon joint. Notably, fine‐tuning the relative coordination directions of the pyridine moieties in the DPTA ligand enables the construction of distinct cavities within the BNFs, thereby achieving molecule‐specific adsorptions. In contrast, BNFs based on 4,4′‐bipyridine (BP) or Salen‐Ni, where the amino‐triazole unit in the DPTA ligand was either removed or replaced with a four‐coordinate Ni^II^ motif, failed to form well‐defined cavities. Consequently, they exhibited no selectivity toward benzene derivatives (Figure 2, vide infra). Under ambient conditions, **BN‐DPTA1** realizes 97.4% removal of trace‐level Tol (50 ppm), while **BN‐DPTA2** competently removes 96.0% of *o*‐xylene (OX) at 50 ppm. More importantly, **BN‐DPTA1** selectively separates Tol from mixtures containing Tol, xylenes, and ethylbenzene (EB), whereas **BN‐DPTA2** is capable of distinguishing OX from *m*‐xylene (MX), *p*‐xylene (PX), and EB. To the best of our knowledge, **BN‑DPTA2** addresses a gap in the use of metal‑free frameworks for highly selective adsorption of OX over all C8 aromatic isomers. Solid‐phase extraction (SPE) columns with **BN‐DPTA1** and **BN‐DPTA2** as stationary phases further achieve the isolation of Tol and OX (>99.9% purity) from simulated petroleum products. Single‐crystal x‑ray diffraction (SC‐XRD) analyses of BNFs containing the corresponding guest molecules reveal the host–guest interactions, combined with density functional theory (DFT) calculations, rationalizing the observed selective adsorption behaviors of **BN‐DPTA1** and **BN‐DPTA2**. Furthermore, powder x‐ray diffraction (PXRD) and in situ Fourier‐transform infrared (FT‐IR) spectroscopy further clarify the adsorption kinetics.

**FIGURE 1:**
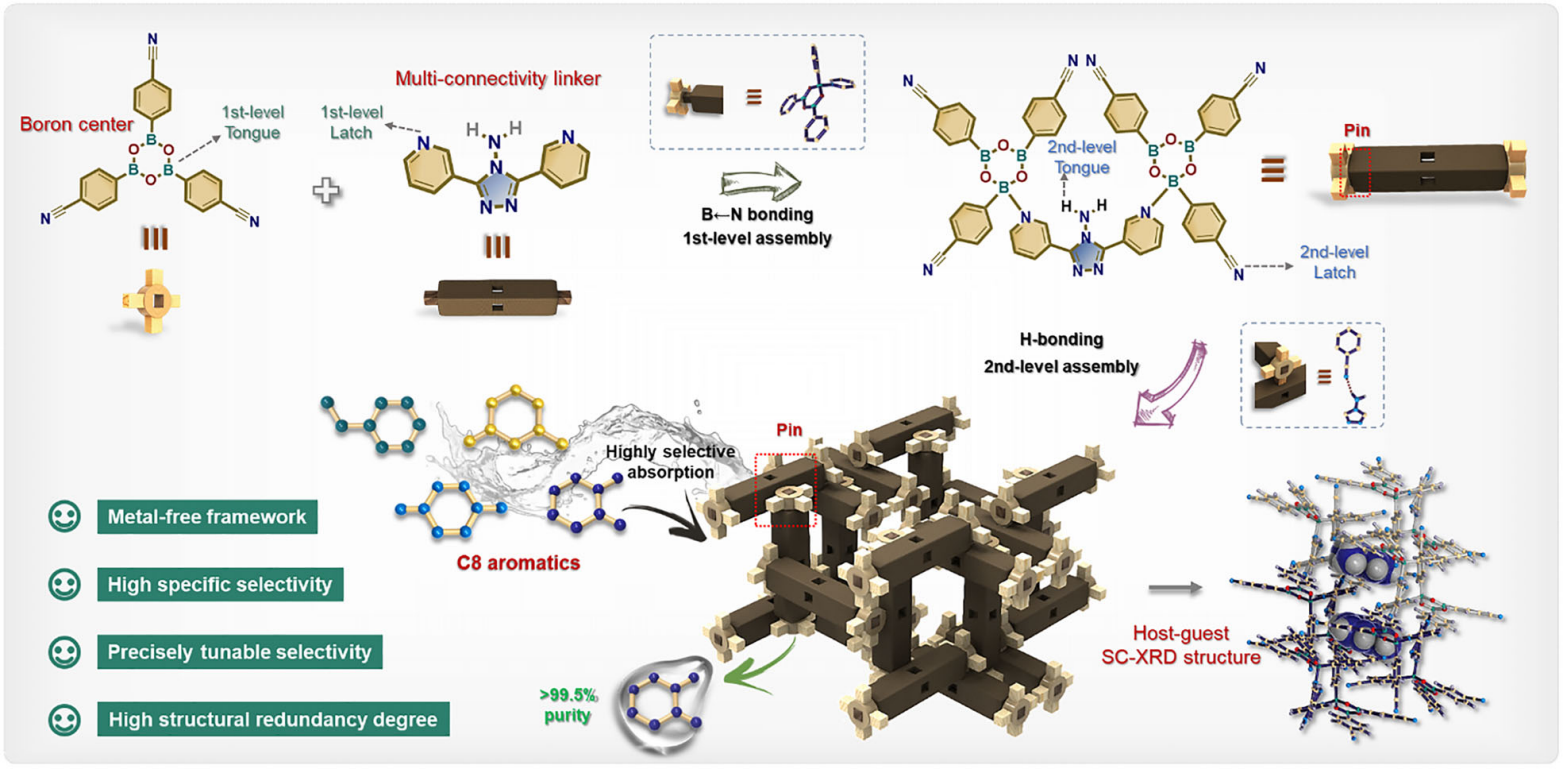
Sunmao (mortise‐and‐tenon) structure‐inspired program‐assembly strategy for crafting BNFs and their applications in selective *o*‐xylene adsorption over all C8 aromatic isomers.

**FIGURE 2:**
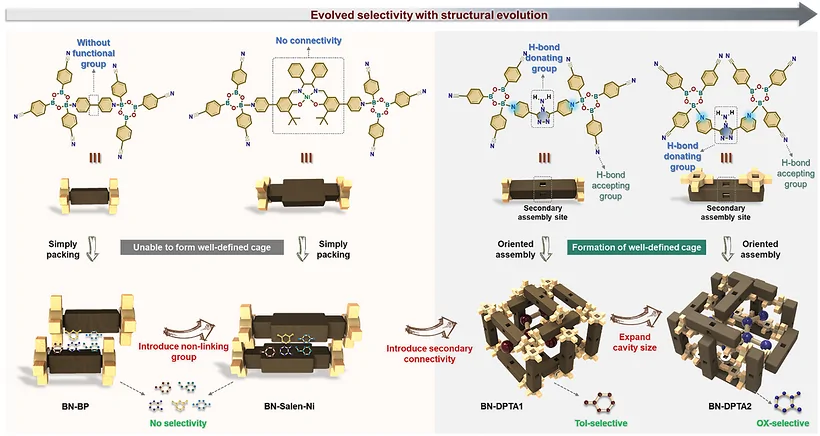
Adsorption performance evolution via structural optimization of **BN‐BP**, **BN‐Salen‐Ni**, **BN‐DPTA1**, and **BN‐DPTA2**.

## 2 Results and Discussion

Treatment of 4‐cyanophenylboronic acid with BP, Salen‐Ni, DPTA1, or DPTA2 at 100°C produced **BN‐BP**, **BN‐Salen‐Ni**, **BN‐DPTA1**, and **BN‐DPTA2**, respectively. A program‐assembly pathway was proposed for the generation of **BN‐DPTA1** and **BN‐DPTA2**, including (1) condensation of 4‐cyanophenylboronic acid to generate a TBTBN block featuring a [B_3_O_3_] ring, (2) coordination of the pyridine motif to the [B_3_O_3_] core, and (3) extra N─H⋯N≡C hydrogen bonding between DPTA and TBTBN. Single‐crystal x‑ray diffraction analyses of newly prepared BNFs were sequentially performed. Both **BN‐DPTA1** and **BN‐DPTA2** crystallized in the triclinic system (space group *P*‐1; Figures S1 and S2 and Tables S1 and S2). In **BN‐DPTA1**, the predicted N─H⋯N≡C hydrogen bonding is observed, together with significant π⋯π overlap between phenyl rings in TBTBN blocks. These non‐covalent interactions ultimately contribute to the construction of well‐defined [B_60_] cages. Meanwhile, **BN‐DPTA2** also exhibits marked N─H⋯N≡C hydrogen bonding yet without π⋯π overlap, and relatively larger [B_36_] cages have formed. The cages in **BN‐DPTA1** and **BN‐DPTA2** are connected, forming regular three‐dimensional frameworks (Figures S3 and S4). **BN‐DPTA1** co‐crystallizes with Tol; specifically, one [B_60_] cage accommodates two Tol molecules through multiple C─H⋯π and π⋯π interactions (Figure 3a). A similar host–guest interaction between **BN‐DPTA2** and OX also exists, as evidenced by crystallographic analyses (Figure 3b).

**FIGURE 3:**
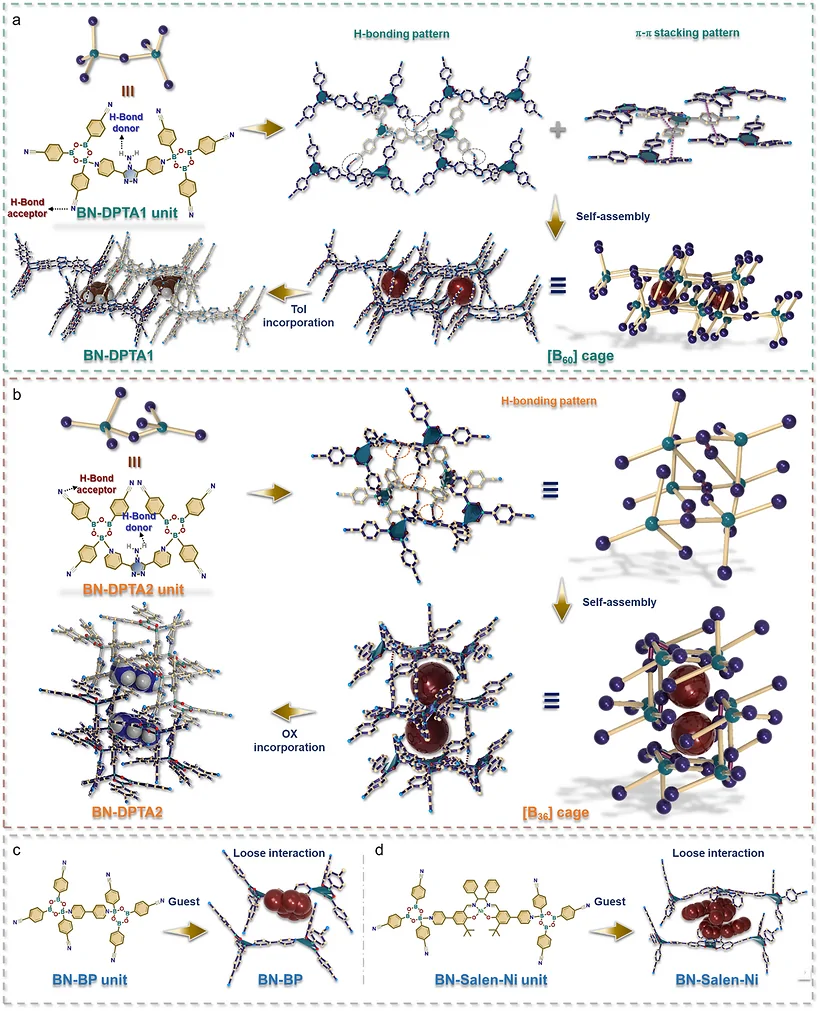
(a) SC‐XRD analyses of the B←N unit, the hydrogen bonding and π⋯π interaction, [B_60_] cage, and host–guest complex of [B_60_] and Tol in **BN‐DPTA1**. (b) SC‐XRD analyses of the B←N unit, the hydrogen bonding, the [B_36_] cage, and the host–guest complex of [B_36_] and OX in **BN‐DPTA2**. SC‐XRD analyses of the host–guest complexes in (c) **BN‐BP** and (d) **BN‐Salen‐Ni** reveal looser host–guest interactions compared to the close encapsulation in **BN‐DPTA1** and **BN‐DPTA2**.

The secondary connectivity plays a critical role in the high adsorption selectivity of **BN‑DPTA1** and **BN‑DPTA2**, which enabled the construction of well‑defined cavities, a challenge when relying solely on B←N dative bonding. To demonstrate this, we designed two control materials where the NH_2_‑functionalized triazole group on the pyridine ligands was either removed or replaced with a Salen‐Ni unit, namely **BN‑BP** and **BN‑Salen‑Ni**, respectively. In both cases, no well‐defined cavities were observed due to the lack of secondary connectivity‐induced interlockings (Figures S5–S10 and Tables S3 and S4). As a result, they showed no selectivity for benzene derivatives (Figure 3c). Optical and scanning electron microscopy imaging revealed that **BN‐DPTA1** adopts a bulk morphology, while **BN‐DPTA2**, **BN‐BP**, and **BN‐Salen‐Ni** display needle shapes, respectively (Figures S11–S13). PXRD analyses further confirmed the high phase purity of BNFs, whose patterns match simulated ones derived from single‐crystal data (Figures S14–S17). **BN‐DPTA1** and **BN‐DPTA2** exhibited good stability in various non‑polar solvents (including benzene, benzotrifluoride, Tol, hexane, xylenes mixtures, and light oil) with PXRD patterns remaining nearly unchanged after immersion for at least 3 days (Figures S18–S20).

According to their single‐crystal structure, the chemical formulas of **BN‐DPTA1**, **BN‐DPTA2, BN‐BP**, and **BN‐Salen‐Ni** were identified as {[(TBTBN)_2_DPTA1]·C_7_H_8_}, {[(TBTBN)_2_DPTA2]·C_8_H_10_}, {[(TBTBN)_2_BP]·C_7_H_8_}, and {[(TBTBN)_2_Salen‐Ni]·(C_7_H_8_)_2_]} respectively. Thermogravimetric analysis (TGA) further verified these chemical formulas. The observed weight loss before 130°C/220°C/220°C/280°C amounts to 7.6%/7.8%/9.0%/10.3% for **BN‐DPTA1**/**BN‐DPTA2/BN‐BP/BN‐Salen‐Ni**, corresponding to the release of guest molecules per formula unit (Figures S21–S24). Fourier‑transform infrared (FT‑IR) spectra confirmed the formation of the TBTBN cluster, the coordination of the pyridine units to TBTBN in **BN‐DPTA1**, **BN‐DPTA2**, **BN‐BP**, and **BN‐Salen‐Ni**, as well as N─H⋯N≡C hydrogen bonding in **BN‐DPTA1** and **BN‐DPTA2**, outlining their generation procedure (Figures S25–S28).

BNFs exhibited dynamic structural changes toward guest removal/re‐adsorption. For example, **BN‐DPTA1** and **BN‐DPTA** released the adsorbed Tol or OX molecules upon heating to reach the activated state denoted as **BN‐DPTA1‐A** and **BN‐DPTA2‐A**, respectively, as demonstrated by the solid ^13^C NMR spectra (Figure 4b). After re‐exposure to Tol or OX, these activated states reverted to the pristine thermodynamically stable host–guest complex **BN‐DPTA1** and **BN‐DPTA2** (Figure 4a–c). This dynamic behavior was monitored by using PXRD analyses at different temperatures. For **BN‐DPTA1**, its characteristic peaks assignable to the (101), (1$\bar{1}$0), (210), (022), (220), and (310) lattice planes gradually disappeared as the heating temperature increased from 25°C, 50°C, 75°C to 100°C. After immersing the generated **BN‐DPTA1‐A** in hot Tol for 1 h, the signals of the corresponding lattice planes reappeared, indicating the recovery of the pristine structure (Figures 4d and S29). **BN‐DPTA2** displayed analogous behavior. Heating induced the gradual disappearance of reflections from the ($\bar{1}$01), (002), (102), (2$\bar{1}$1), and (2$\bar{3}$3) planes, corresponding to the loss of encapsulated OX. Subsequent exposure to hot OX restored the original diffraction patterns, demonstrating full recovery (Figures 4d and S30). This phase transition was further verified by thermogravimetric analysis (TGA, Figures S31–S34). No solvent‐loss stage occurred for either **BN‐DPTA1‐A** or **BN‐DPTA2‐A**. After exposure to guest solvents, their TGA profiles mirrored those of the pristine **BN‐DPTA1** and **BN‐DPTA2**. Such a reversible switch between “swell” and “shrink” states did not involve chemical changes [10, 49], as monitored by single‐crystal unit‐cell tests, computational study and XPS analyses (Table S5 and Figures S35–S38). Tol and OX were then collected via a simple heating process (Figures S39 and S40).

**FIGURE 4:**
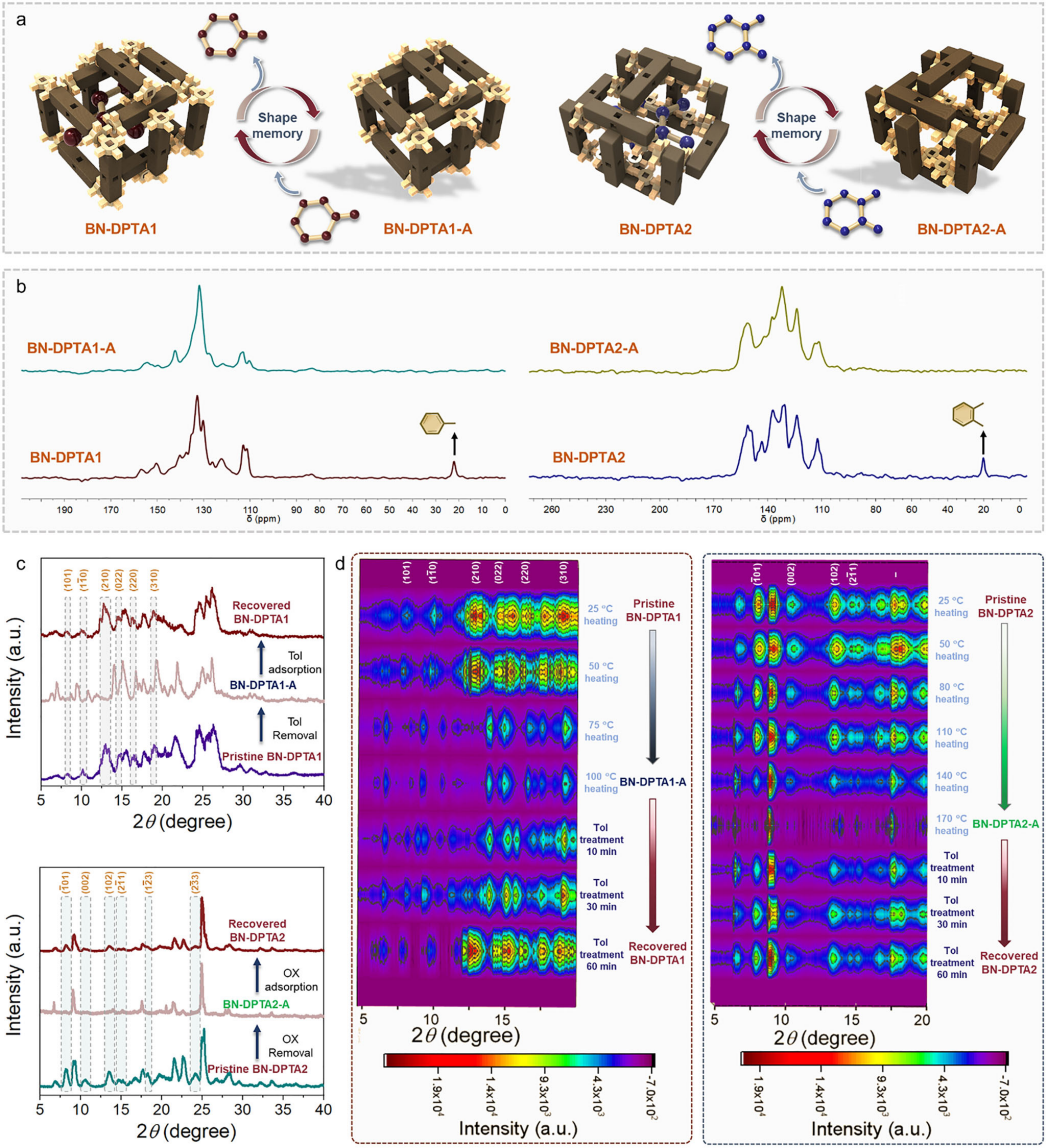
(a) Schematic representation showing the dynamic crystal‐phase transformation with guest removal/re‐adsorption. (b) Solid ^13^C NMR spectra of **BN‐DPTA1**, **BN‐DPTA1‐A**, **BN‐DPTA2**, and **BN‐DPTA2‐A**. (c, d) PXRD tracking of the dynamic crystal‐phase change.

**BN‑DPTA1** and **BN‑DPTA2** presented selective, recyclable guest adsorption ability due to the shape‑memory and size‑exclusive characteristics. Specifically, **BN‐DPTA1‐A** exhibited little adsorption of C8 aromatics, whereas **BN‐DPTA2‐A** almost did not capture MX, PX, and EB. Their PXRD patterns remained virtually unchanged after exposure to these molecules (Figures S41 and S42). The highly selective adsorption capability of **BN‐DPTA1** and **BN‐DPTA2**, along with the reversible adsorption/desorption behavior, makes them promising candidates for Tol and OX separation. We subsequently evaluated their separation performance. Adsorption curves monitored over time revealed that **BN‐DPTA1‐A** preferentially adsorbed Tol over xylene isomers when exposed to their equimolar binary mixtures (Tol/OX, Tol/MX, Tol/PX, and Tol/EB). The saturated adsorption capacities toward Tol were measured as 0.92, 0.87, 0.93, and 0.60 mol/mol within 300 min from these mixtures, with markedly low xylene and EB adsorption (Figures 5a and S43–S66). Meanwhile, adsorption curves of **BN‐DPTA2‐A** illustrated its selective adsorption of OX, achieving saturated capacities of 0.68, 0.62, and 0.63 mol/mol within 300 min. Other xylene isomers showed weak affinity with **BN‐DPTA2‐A** (Figures 5b and S67–S84).

**FIGURE 5:**
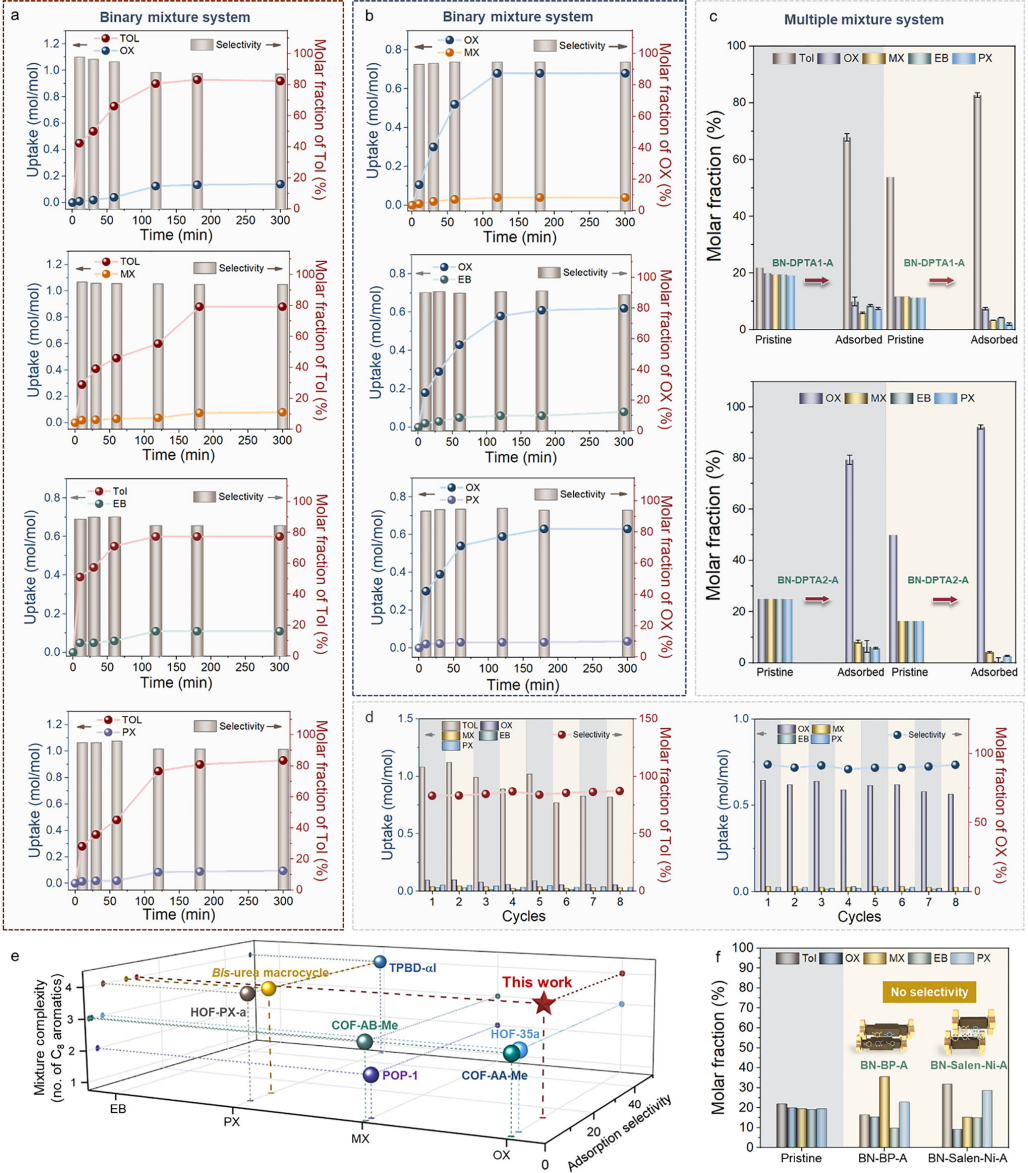
(a) Selective adsorption of Tol by **BN‐DPTA1** from binary mixtures. (b) Selective adsorption of OX by **BN‐DPTA2** from binary mixtures. (c) Selective adsorption performance of **BN‐DPTA1** and **BN‐DPTA2** from multiple‐component mixtures. (d) Cyclic adsorption tests from multiple‐component mixtures. (e) Comparison in C8 aromatics’ separation among metal‐free crystalline frameworks; adsorption selectivity of component *i* relative to *j* is defined as: S*_i_*_/_ *_j_* = (x*_i_*/x*_j_*)×(y*_j_*/y*_i_*), where x*_i_* and x*_j_* are the mole fractions of components *i* and *j* in the guest‐loaded phase, and y*_i_* and y*_j_* are the mole fractions of components *i* and *j* in the liquid or vapor phase. (f) Adsorption performance of **BN‐BP** and **BN‐Salen‐Ni** from equimolar multiple‐component mixtures.

Encouraged by their adsorption performance, multi‐component competitive adsorption tests were later conducted. To our delight, when exposed to equimolar (each ∼20%) and 50%/50% molar ratio Tol/xylene isomer quinary mixtures, **BN‐DPTA1‐A** achieved Tol adsorption proportions of 67.9% ± 1.3% and 82.8% ± 0.8% with adsorption selectivity of *S*_Tol/PX_ = 8.9 ± 0.5, *S*_Tol/EB_ = 8.0 ± 0.3, *S*_Tol/MX_ = 11.3 ± 0.6, and *S*_Tol/OX_ = 6.8 ± 1.0, respectively (Figures 5c, S85 and S86). Meanwhile, **BN‐DPTA2‐A** effectively differentiated OX from all C8 aromatic isomers, achieving 79.4% ± 1.8% and 92.2% ± 0.8% selectivity from equimolar (each ∼25%) and 50/50 molar ratio OX/xylene isomer quaternary mixtures with adsorption selectivity of *S*_OX/PX_ = 13.7 ± 0.8, *S*_OX/EB_ = 12.4 ± 4.5, and *S*_OX/MX_ = 9.5 ± 0.7, respectively (Figures 5c, S87 and S88). The saturated adsorption capacities were calculated to be approximately 0.95 molecules of Tol per host unit for **BN‑DPTA1** and 0.65 molecules of OX per host unit for **BN‑DPTA2** (Figure S89). Notably, **BN‑DPTA2‑A** represents a rare example of a metal‑free framework capable of selectively separating OX from all C8 aromatic isomers, with adsorption selectivity superior to that of other metal‑free frameworks reported to date (Figure 5e and Table S6). Both **BN‐DPTA1** and **BN‐DPTA2** showed selective adsorption/desorption for at least eight cycles without obvious loss of performance or structural change (Figures 5d and S90–S95). In contrast, **BN‐BP‐A** and **BN‐Salen‐Ni‐A**, activated states of **BN‐BP** and **BN‐Salen‐Ni** (Figures S96–S99), showed no obvious affinity toward particular guest molecules (Figures 5f, S100, and S101).

Gram‐scale (> 10 g) synthesis of phase‐pure **BN‐DPTA1** and **BN‐DPTA2** was readily achieved in a one‐pot procedure (Figures 6a and Figures S102–S104). To evaluate their potential practical applications, both BNFs were immersed in artificial light oil for over 1 week. SC‐XRD analyses confirmed that their crystalline structures remained stable, with no discernible changes in unit cell parameters (Figure 6b and Tables S7 and S8). Solid phase extraction (SPE) columns were then prepared using **BN‐DPTA1‐A** or **BN‐DPTA2‐A** as packings, respectively (Figure S105). Mixtures mimicking crude petroleum products (consisting of multiple C7‐C9 components derived from petroleum; see Supporting Information for detailed composition) went through these SPE columns to deliver Tol and OX with >99.5% purity. The adsorption capacity was calculated to be 0.91 mmol/g and 0.78 mmol/g (guest/host) for **BN‐DPTA1‐A** and **BN‐DPTA2‐A**‐based columns, respectively (Figures 6c,d, S106, and S107). The separation performance of both columns remained undiminished after five consecutive extraction cycles, demonstrating their excellent reusability (Figures S108 and S109). This separation technology might offer a more energy‐efficient and eco‐friendly solution, complementing traditional distillation. Although several BNFs have been reported to selectively adsorb Tol [42, 43], differentiation among xylene isomers by BNFs remains unexplored to the best of our knowledge.

**FIGURE 6:**
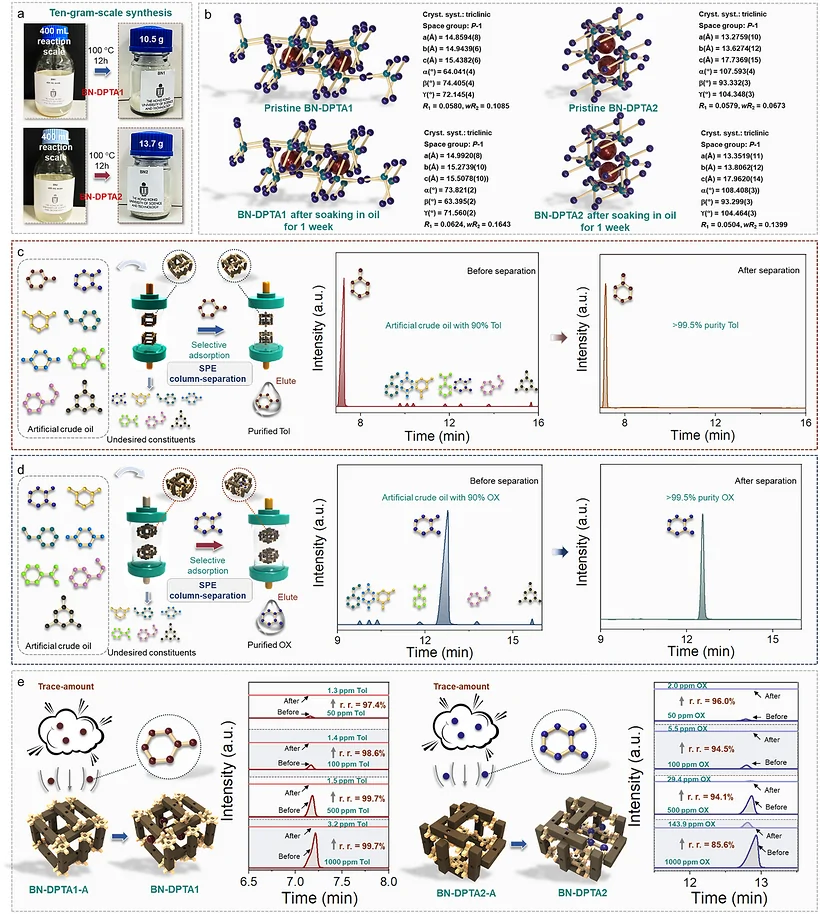
(a) Large‐scale syntheses of **BN‐DPTA1** and **BN‐DPTA2**. (b) SC‐XRD structural parameters of **BN‐DPTA1** and **BN‐DPTA2** before and after soaking in light oil for 1 week. SPE column separation of Tol/OX using **BN‐DPTA1‐A** (c) and **BN‐DPTA2‐A** (d) as stationary phases. (e) Removal of Tol/OX at trace‐amount level by **BN‐DPTA1‐A**/**BN‐DPTA2‐A**, r. r. = removal ratio.

As benzene derivatives are classified as neurotoxins and developmental toxicants, their efficient removal from the environment is critical. For context, the National Institute for Occupational Safety and Health (NIOSH, USA) recommends exposure limits of approximately 200 and 100 ppm for Tol and OX in the workplace [63, 64]. Given their remarkable adsorption capability, synthetic accessibility, and exceptional stability, **BN‐DPTA1‐A** and **BN‐DPTA2‐A** were explored as reusable adsorbents to remove trace amounts of Tol and OX. When exposed to atmospheres containing 50, 100, 500, and 1000 ppm of these toxic contaminants, removal efficiencies of BNFs toward Tol/OX were measured as 97.4%/96.0%, 98.6%/94.5%, 99.7%/94.1%, and 99.7%/85.6%, respectively (Figures 6e and S110, and S111). At the 50‐ppm level, only 1.3 ppm of Tol and 2.0 ppm of OX remained in the purified air.

In situ FT‐IR spectroscopy was conducted to illustrate the adsorption kinetics. A continuous increase in the intensity of the band at 3023.0 cm^−1^ (for **BN‐DPTA1‐A**) and 3016.5 cm^−1^ (for **BN‐DPTA2‐A**) was observed upon exposure to the corresponding guest molecules. These bands are assigned to the C−H stretching vibration of the phenyl ring, indicating the progressive uptake of these aromatic guests into BNFs (Figure 7a,b) [20]. To further shed light on host–guest interactions between BNFs and aromatic guests, DFT calculations were performed. We hypothesized that electrostatic attraction could be one contributor and thereby calculated the electrostatic potential (ESP) maps. The positive charge of DPTA linkers is located mainly at the middle triazole part, which was computed as 0.07 and 0.05 eV for **DPTA1** and **DPTA2**, respectively. Upon coordination with [B_3_O_3_] clusters to form BNFs, the positive potentials increase to 0.11 and 0.08 eV. In contrast, Tol and OX exhibit negative ESP values of −0.03 eV, suggesting a favorable electrostatic complementarity that facilitates adsorption (Figures 7c,  S112, and S113).

**FIGURE 7:**
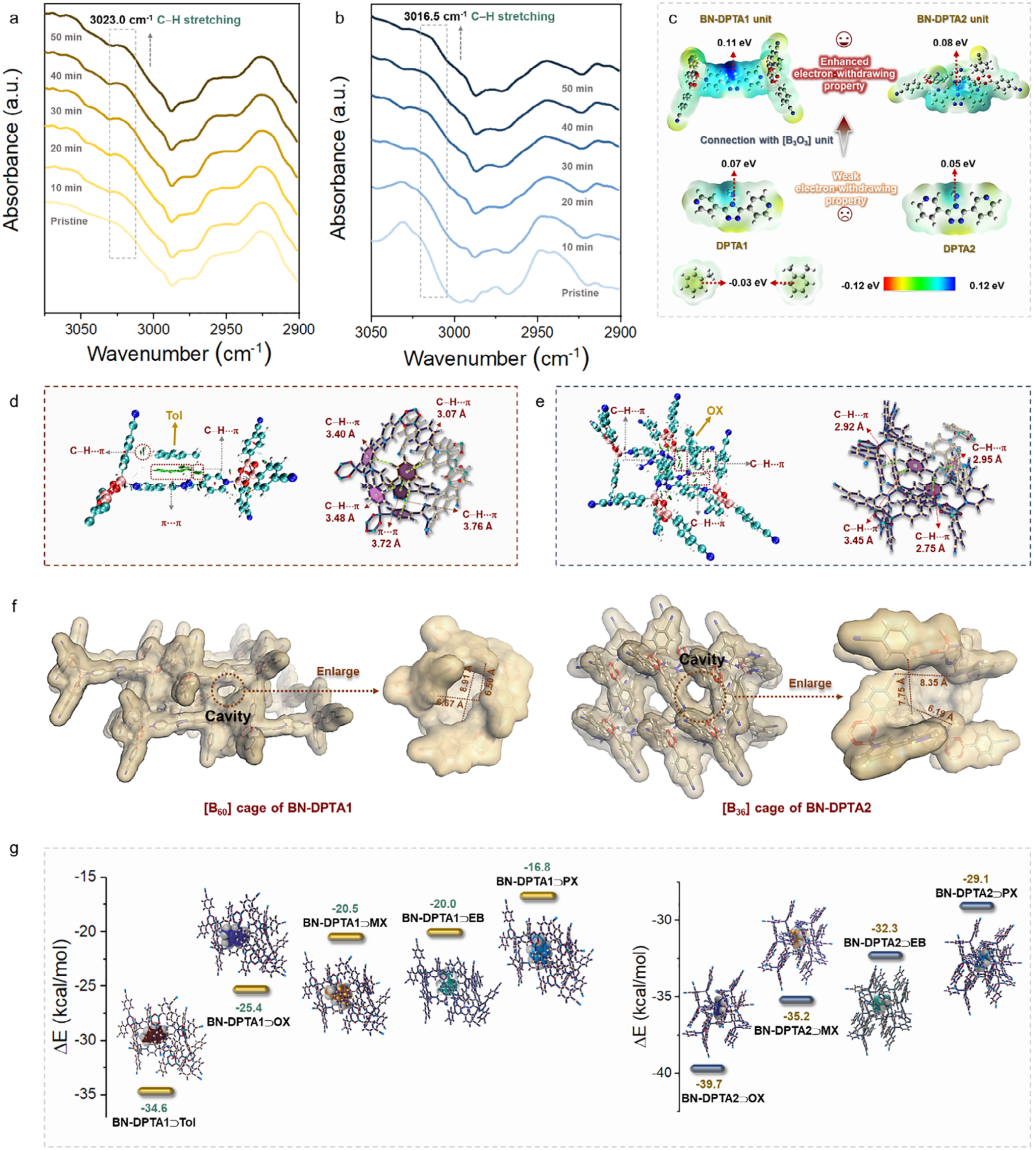
In situ FT‐IR monitoring of the adsorption process of **BN‐DPTA1‐A** (a) and **BN‐DPTA2‐A** (b) toward Tol and OX. (c) ESP analyses of B←N units in **BN‐DPTA1**/**BN‐DPTA2** and Tol/OX. IG‐MH analyses and SC‐XRD structure of the host–guest complex in **BN‐DPTA1**⊃Tol (d) and **BN‐DPTA2**⊃OX (e). (f) Accessible Connolly surface of the [B_60_] and [B_36_] cages in **BN‐DPTA1** and **BN‐DPTA2**, respectively. (g) Binding energy of **BN‐DPTA1** and **BN‐DPTA2** toward different guests.

As we obtained single‐crystal structures of **BN‐DPTA1** and **BN‐DPTA2** containing guest aromatics, further analyses were built upon them. In the single crystal of **BN‐DPTA1**, the methyl and phenyl C−H groups of Tol engage with three adjacent phenyl rings through C─H⋯π interactions, and its phenyl ring participates in additional C─H⋯π and π⋯π interactions with TBTBN aryl C─H and the triazole ring, respectively. These multiple interactions lead to its tight encapsulation within the **BN‐DPTA1** cavity. In **BN‐DPTA2**, C─H groups of OX form C─H⋯π interactions with the adjacent three phenyl rings of the host, resulting in its tight confinement within the cavity (Figure 7d,e). These interactions were also demonstrated by computation. An independent gradient model based on the Hirshfeld partition (IGMH) revealed multiple C─H⋯π and π⋯π interactions between BNFs and aromatic rings [65, 66]. Reduced density gradient (RDG) analyses corroborated these findings, confirming the non‐covalent nature of host–guest interactions (Figures S114 and S115).

According to crystal structures, the cavity in **BN‐DPTA1** measures approximately 8.9 Å × 6.7 Å × 6.5 Å, which accommodates the Tol molecule (kinetic dimensions: 8.1 Å × 6.5 Å × 4.0 Å) but excludes xylene isomers with larger dimensions (OX: 7.7 Å × 7.2 Å × 4.0 Å; MX: 8.6 Å × 7.2 Å × 4.0 Å; PX: 9.0 Å × 6.5 Å × 4.0 Å; EB: 9.4 Å × 6.9 Å × 4.0 Å). **BN‐DPTA2** features the cavity dimensions of 8.4 Å × 7.8 Å× 6.2 Å, perfectly matching the size of OX while excluding the bulkier MX, PX, and EB isomers (Figures 7f and S116–S118). To quantify the thermodynamic basis of the observed selectivity, we computed the binding energies between BNFs and aromatic guests. **BN‐DPTA1** shows a binding energy of −34.6 kcal/mol for Tol, substantially lower than those of −25.4, −20.5, −20.0, and −16.8 kcal/mol for OX, MX, EB, and PX. For **BN‐DPTA2**, its binding energy with OX (−39.7 kcal/mol) is significantly more favorable than those with MX (−35.2 kcal/mol), EB (−32.3 kcal/mol), and PX (−29.1 kcal/mol) (Figure 7g). These calculated results match the experimental observations, providing a thermodynamic rationale for the adsorption selectivity. To further validate the importance of the confined cavity, the ESP maps of **BN‐BP** and **BN‐Salen‐Ni** were evaluated. Both frameworks exhibit positive ESPs (0.08 and 0.04, respectively), which enables them to attract electron‐rich guest molecules (Figure S119). However, due to the absence of ordered and confined cavities, differentiating among Tol, OX, MX, EB, and PX has become quite difficult for these BNFs.

A programmed assembly strategy has been developed to synthesize BNFs with improved stability and versatile adsorption functions. The key to success lies in the rational integration of secondary connectivity in the bridging linkers. The insights into the structure/performance relationship gained from mechanistic study might implicate the design of next‐generation separation materials beyond BNFs in this work.

## Author Contributions

**Xiang‐Shuai Li**: conceptualization, investigation, writing – original draft, methodology. **Yizhou Wang**: investigation. **Wing‐Laam Luk**: investigation. **Jieying Hu**: investigation. **Rong Fu**: investigation. **Zhao Zhang**: investigation. **Zenan Chen**: investigation. **Jian Zhao**: investigation. **Wan Chan**: writing – review and editing. **Yangjian Quan**: writing – review and editing, conceptualization.

## Conflicts of Interest

The authors declare no conflicts of interest.

## Supporting information



Full details of the experimental methods, PXRD, NMR, TGA, FT‐IR, XPS, GC spectra, SEM images, x‐ray crystallographic data, and DFT calculations.**Supporting File 1**: anie74292‐sup‐0001‐SuppMat.zip.

**Supporting File 2**: anie74292‐sup‐0002‐SuppMat.pdf.

## Data Availability

The data that support the findings of this study are available in the Supporting Information of this article.
