# Supplementary material for: Selective Adsorption of o‐Xylene From all C8 Aromatics via Secondary Connectivity Manipulation in B←N Frameworks
Source: Angew Chem Int Ed Engl. 2026 Aug 19;65(42):e1406923. doi: 10.1002/anie.1406923 (PMC13653566; doi:10.1002/anie.1406923)
Supplement: Supplementary file 1 [file ANIE-65-e1406923-s002.pdf]

Selective adsorption of *o*-xylene from all C8 aromatics via secondary connectivity manipulation in B←N frameworks

Xiang-Shuai Li,[1] Yizhou Wang,[1] Wing-Laam Luk,[1] Jieying Hu,[1,3] Rong Fu,[2] Zhao Zhang,[4] Zenan Chen,[1] Jian Zhao,[5] Wan Chan,[1] and Yangjian Quan[1]*

[1]Department of Chemistry, The Hong Kong University of Science and Technology (HKUST), Clear Water Bay, Kowloon, Hong Kong SAR (China)

[2]College of Environmental Science and Engineering, Ministry of Education Key Laboratory of Pollution Processes and Environmental Criteria, Tianjin Key Laboratory of Environmental Remediation and Pollution Control, Nankai University, Tianjin 300350, China

[3]School of Chemical Engineering and Light Industry, Guangdong University of Technology, Guangzhou 510006, China

[4]Frontiers Science Center for New Organic Matter, State Key Laboratory of Advanced Chemical Power Sources, Key Laboratory of Advanced Energy Materials Chemistry (Ministry of Education), Haihe Laboratory of Sustainable Chemical Transformations, Renewable Energy Conversion and Storage Center, College of Chemistry, Nankai University, Tianjin 300071, P. R. China

[5]Ganjiang Innovation Academy, Jiangxi Institute of Rare Earths, Chinese Academy of Sciences, Ganzhou 341000, P. R. China

**Corresponding author, e-mail:* chyjquan@ust.hk

Content

1. General information **S3**

2. Experimental methods **S3**

3. Computational details **S8**

4. Supplementary tables and figures **S9**

5. References **S109**

1. General information

All starting materials, reagents, and solvents were obtained from commercial sources and used without further purification. Fourier Transform infrared (FT-IR) spectra were recorded in the range of 400–4000 cm^{-1} on a Nicolet Avatar 360 spectrophotometer. ^1H NMR spectra were acquired using a Bruker AVII 400 MHz NMR spectrometer. Thermogravimetric analysis (TGA) was performed under a nitrogen atmosphere using a PerkinElmer STA 6000 thermal analysis system at a heating rate of 15 °C/min in the range of R.T.-800 °C. Scanning electron microscopy (SEM) was conducted using a JSM-6390 (JEOL) instrument. Powder X-ray diffraction (XRD) data were collected in reflection mode at room temperature on a Rigaku Miniflex 600 diffractometer with Cu Kα radiation (λ = 1.54056 Å). Single-crystal X-ray diffraction (SC-XRD) data were collected on a Bruker D8 VENTURE diffractometer. X-ray photoelectron spectroscopy (XPS) analyses were carried out using an Axis Supra$^+$ (Kratos Analytical Limited) spectrometer. Purified products obtained via solid-phase extraction (SPE) column were analyzed by Gas chromatography (GC). GC analyses were performed on a Scion GC 456i system equipped with a SCION-WAXMS column (60 m × 0.32 mm × 0.50 μm).

2. Experimental methods

2.1 Synthesis of BN-DPTA1: A glass bottle was charged with 4-cyanophenylboronic acid (20 mg, 0.14 mmol), 3,5-di(pyridin-4-yl)-4H-1,2,4-triazol-4-amine (DPTA1) (10 mg, 0.04 mmol), and 4 mL dry toluene (Tol) under a nitrogen atmosphere. The obtained mixture was sonicated to ensure uniform dispersion and then placed in a 100 °C oven for 24 hours. After the reaction, transparent crystals were collected by centrifugation, washed three times with Tol, and dried to obtain pure product, designated as **BN-DPTA1** (19.8 mg). Yield: 79.2%

(calculated based on 4-cyanophenylboronic acid).

2.2 Synthesis of BN-DPTA2: A glass bottle was charged with 4-cyanophenylboronic acid (10 mg, 0.07 mmol), 3,5-di(pyridin-3-yl)-4H-1,2,4-triazol-4-amine (DPTA2) (10 mg, 0.04 mmol), and 4 mL dry *o*-xylene (OX) under a nitrogen atmosphere. The obtained mixture was sonicated to ensure uniform dispersion and then placed in a 100 °C oven for 24 hours. After the reaction, transparent crystals were collected by centrifugation, washed three times with OX, and dried to obtain pure product, designated as **BN-DPTA2** (10.0 mg). Yield: 76.9% (calculated based on 4-cyanophenylboronic acid).

2.3 Synthesis of BN-BP: A glass bottle was charged with 4-cyanophenylboronic acid (10 mg, 0.07 mmol), 4,4'-bipyridine (BP) (10 mg, 0.06 mmol), and 4 mL dry Tol under a nitrogen atmosphere. The obtained mixture was sonicated to ensure uniform dispersion and then placed in a 100 °C oven for 24 hours. After the reaction, transparent crystals were collected by centrifugation, washed three times with Tol, and dried to obtain pure product, designated as **BN-BP** (6.8 mg). Yield: 57.1% (calculated based on 4-cyanophenylboronic acid).

2.4 Synthesis of Salen-Ni ligand: The synthetic procedure was according to a previous report with slightly modification.[1] In a 100 mL round-bottom flask equipped with a magnetic stirrer, 3-(*tert*-butyl)-2-hydroxy-5-(pyridin-4-yl)benzaldehyde (220 mg, 0.9 mmol) and 1,2-diphenylethane-1,2-diamine (83 mg, 0.4 mmol) were dissolved in methanol (20 mL). The reaction mixture was then stirred at 70 °C for 6 h under nitrogen atmosphere. Subsequently, the solution was dropwise added into a methanol solution (10 mL) of $Ni(OAc)_2 \cdot 4H_2O$ (150 mg, 0.6 mmol) under N_2 atmosphere. The resulting mixture was stirred at 70 °C for 8 h. After cooling down to room temperature, red powder was formed and collected by filtration. After being washed by methanol for three time, the pure product was obtained (yield: 215.6 mg, 75.0%). ^{1}H NMR (400 MHz, $CDCl_3$): δ 8.53 (d, *J* = 8.5 Hz, 4H), 7.57 (d, *J* = 7.6 Hz, 6H), 7.37-7.45 (m, 10H), 7.32 (s, 2H), 7.15 (d, *J* = 7.2 Hz, 2H),

4.59 (s, 2H), 1.50 (s, 18H).

2.5 Synthesis of BN-Salen-Ni: A glass bottle was charged with 4-cyanophenylboronic acid (5 mg, 0.034 mmol), Salen-Ni (5 mg, 0.007 mmol), and 4 mL dry Tol under a nitrogen atmosphere. The obtained mixture was sonicated to ensure uniform dispersion and then placed in a 100 °C oven for 24 hours. After the reaction, transparent crystals were collected by centrifugation, washed three times with Tol, and dried to obtain pure product, designated as **BN-Salen-Ni** (8.8 mg). Yield: 88.9% (calculated based on 4-cyanophenylboronic acid).

2.6 Large scale synthesis of BN-DPTA1: A glass bottle was charged with 4-cyanophenylboronic acid (10 g, 68.0 mmol), 3,5-di(pyridin-4-yl)-4H-1,2,4-triazol-4-amine (DPTA1) (5 g, 21.0 mmol), and 400 mL dry toluene (Tol) under a nitrogen atmosphere. The obtained mixture was sonicated to ensure uniform dispersion and then placed in a 100 °C oven for 24 hours. After the reaction, transparent crystals were collected by centrifugation, washed three times with Tol, and dried to obtain pure product (10.5 g). Yield: 84.0% (calculated based on 4-cyanophenylboronic acid).

2.7 Large scale synthesis of BN-DPTA2: A glass bottle was charged with 4-cyanophenylboronic acid (12.5 g, 85.0 mmol), 3,5-di(pyridin-3-yl)-4H-1,2,4-triazol-4-amine (DPTA2) (12.5 g, 52.5 mmol), and 400 mL dry OX under a nitrogen atmosphere. The obtained mixture was sonicated to ensure uniform dispersion and then placed in a 100 °C oven for 24 hours. After the reaction, transparent crystals were collected by centrifugation, washed three times with OX, and dried to obtain pure product (13.7 g). Yield: 86.2% (calculated based on 4-cyanophenylboronic acid).

2.8 Collection of Tol/OX from BN-DPTA1/BN-DPTA2: Under N_2 atmosphere, **BN-DPTA1** or **BN-DPTA2** were placed in a round-bottom flask fitted with a condenser and heated to 120 °C or 200 °C, respectively. The released Tol and OX from BNFs were condensed and collected.

2.9 Syntheses of activated BNFs: The BNF solids were placed in a glass bottle, purged with N_2 three times via vacuum-N_2 cycles, and then heated under vacuum at 110 °C/180 °C/160 °C/140 °C for 24 hours to yield the activated **BN-DPTA1-A/BN-DPTA2-A/BN-BP-A/BN-Salen-Ni-A**.

2.10 Revert from BN-DPTA1-A and BN-DPTA2-A to BN-DPTA1 and BN-DPTA2: BN-DPTA1-A or **BN-DPTA2-A** was added into a glass bottle containing dry Tol or OX under N_2 protection. The resulting mixture was sonicated for well dispersion and then placed in a 100 °C oven for 24 hours. After that, the solid was separated by centrifugation and washed with Tol or OX for 3 time to give **BN-DPTA1** or **BN-DPTA2**.

2.11 Evaluation of adsorption performance for BN-DPTA1-A and BN-DPTA2-A: The adsorption evaluation was conducted according to previous reports.[2-4] **BN-DPTA1-A** or **BN-DPTA2-A** (5 mg, 0.005 mmol) was added to mixtures of benzene derivatives under N_2 atmosphere. The system was then placed in a 100 °C oven for a predetermined time to allow for selective adsorption of specific guest molecules from the mixture. After that, the solid was separated from the naturally cooled system by centrifugation and washed five times with hexane to thoroughly remove any guest molecules attached to the surface of the BNFs. The obtained solid was then transferred into a mixed solvent of $CDCl_3$/EtOH (500 μL/100 μL) and sonicated for 30 min to ensure complete release of the adsorbed guests. 1,3,5-Trimethoxybenzene (0.005 mmol) in an equimolar amount to the BNFs was added as an internal standard. The mixture was then subjected to ^1H NMR analysis to determine the molar ratios of the adsorbed guest molecules.

2.12 Calculation of adsorption selectivity: The adsorption selectivity of component *i* relative to component *j* is defined as: $S_{i/j} = (x_i/x_j)\times(y_j/y_i)$. where x_i and x_j are the mole fractions of components *i* and *j* in the guest-loaded phase, and y_i and y_j are the mole fractions of components *i* and *j* in the liquid phase.[2]

2.13 Cyclic adsorption/desorption tests: To assess the adsorption recyclability, **BN-DPTA1-A** or **BN-DPTA2-A** was introduced into a mixture of benzene derivatives. After the adsorption process, the captured guest molecules were removed by vacuum at 110 °C (for **BN-DPTA1-A**) or 180 °C (for **BN-DPTA2-A**), thereby regenerating the activated BNFs for subsequent adsorption cycles.

2.14 Standard curves of Tol and OX by GC. To a series of sealed vessels containing Tol or OX was added 1 mL of hexane, giving solutions with concentrations ranging from 5 to 1000 ppm. After being thoroughly shaken, 2 μL of the resulting solutions were subjected to GC-FID analyses. Plotting the obtained peak areas with concentrations generated the standard curves of Tol and OX.

2.15 Removal of Tol/OX at trace-amount level: BN-DPTA1-A or **BN-DPTA2-A** (300 mg) was placed in a closed 10 mL bottle with atmosphere containing Tol or OX at various concentrations ranging from 50 to 1000 ppm. The system was kept under ambient conditions for three days to allow for adsorption. After that, 1 mL of hexane was added to the bottle, which was thoroughly shaken to fully extract residual Tol or OX. The mixture was then centrifuged, and the liquid supernatant was collected for GC analysis to determine the remaining amount of Tol or OX.

2.16 Preparation of SPE column: The empty column was first washed with hexane. A filter was placed at the bottom outlet, and 101.2 mg of **BN-DPTA1-A** or **BN-DPTA2-A** solid was added into the column. Another filter was then placed on top of the solid and gently pressed down to ensure the tight packing.

2.17 Preparation of artificial crude petroleum oil: The crude oil used in the **BN-DPTA1-A** based SPE column separation was prepared by mixing eight components at the following molar ratios: Tol (90%), OX (1.43%), *m*-xylene (MX, 1.43%), ethylbenzene (EB, 1.43%), *p*-xylene (PX, 1.43%), propylbenzene (1.43%), cumene (1.43%), and mesitylene (1.43%).

The crude oil used in the **BN-DPTA2-A** based SPE column separation was prepared by mixing seven components at the following molar ratios: OX (90%), MX (1.67%), EB (1.67%), PX (1.67%), propylbenzene (1.67%), cumene (1.67%), and mesitylene (1.67%).

2.18 Separation using SPE column: The **BN-DPTA1-A** or **BN-DPTA2-A** based SPE column was placed in a holder in a vertical position. Artificial crude petroleum oil (1 mL) was gradually loaded onto the column, allowing them gradually pass through from top to bottom at 323K, 101.3 kPa. After the oil had completely passed through, the column was washed with 5 mL of hexane to remove any analytes that were non-specifically adsorbed on the surface. Subsequently, 1 mL of acetonitrile was injected to elute the analytes adsorbed in the BNFs in the column. The eluate was collected and analyzed by GC to determine the purity of the isolated analytes. To determine the adsorption capacity of the SPE column, the packing material was collected from column and transferred into a mixed solvent of $CDCl_3/CH_3OH$ (800 μL/200 μL). The dispersion was sonicated for 30 min to ensure complete release of the adsorbed analytes. 1,3,5-Trimethoxybenzene (0.1 mmol) in an equimolar amount to the BNFs from the column was added as an internal standard. The mixture was then subjected to ^1H NMR analysis to determine the molar ratios of the adsorbed guest molecules.

3. Computational details

Density functional theory (DFT) calculations were carried out using the B3LYP-(D3)BJ/6-31G(d,p) level using Gaussian 16.[5] Electrostatic potential (ESP) for BNFs, guest molecules and host-guest complexes were viewed by Gauss View 6.0 with DFT optimized structures. The reduced density gradient (RDG) was evaluated by Multiwfn 3.8 program[6] and visualized using VMD software[7]. The binding energy (ΔE) between BNFs and guests were

calculated by subtracting the energy of the BNFs and the free guest molecule from the energy of the BNF-guest complex. Structural optimizations of the isolated BNFs were performed at the B3LYP-(D3)BJ/6-31G(d,p) level of theory in the gas phase. Initial guess geometries were generated from the original crystallographic information (.cif) files after removal of all guest molecules, allowing visualization of the guest-free, contracted pore architecture. Notably, the optimization of isolated **BN-DPTA1** converged fully, whereas that of isolated **BN-DPTA2** failed to achieve complete convergence. The final optimized geometries, along with the energy profile recorded at each optimization step for **BN-DPTA2**, are presented below:

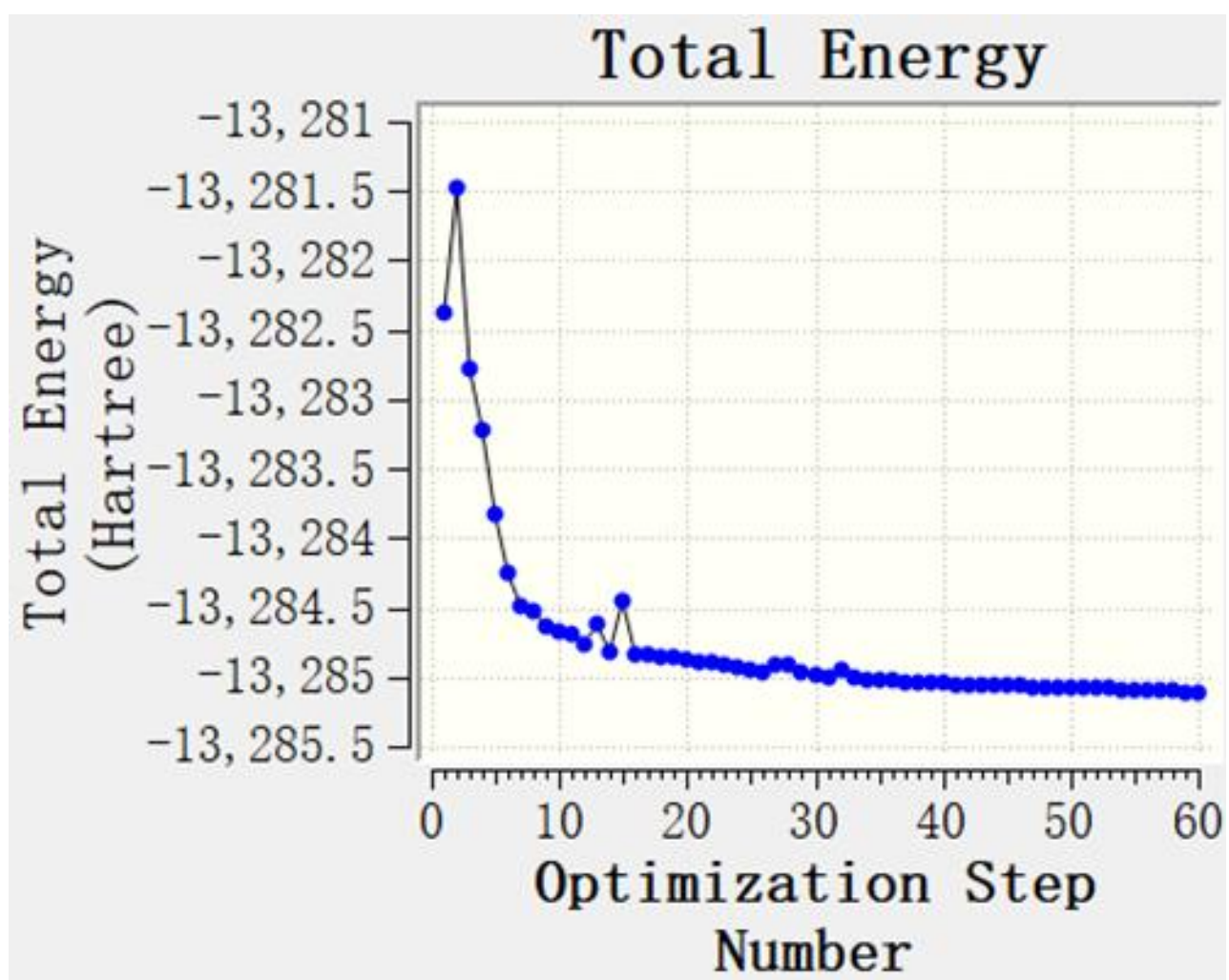

4. Supplementary tables and figures

Table S1. Crystal data and structure refinement details for **BN-DPTA1**.

Identification code	**BN-DPTA1**
Formula	$C_{61}H_{42}B_6N_{12}O_6$
Mr.	1104.036

Temperature (K)	100.00(10)
Cryst. Syst.	triclinic
Space group	*P*-1
a (Å)	14.8594(8)
b (Å)	14.9439(6)
c (Å)	15.4382(6)
α, β, γ (°)	64.0, 74.4, 72.1
Volume ($Å^3$)	2897.3(3)
Z	2
μ (mm^{-1})	0.083
$F_{(000)}$	1140.7
Radiation	MoKα (λ = 0.71073)
2θ (°)	5.06 to 52
Reflections collected	20698
Independent Refl.	11230 [R_{int} = 0.0431, R_{sigma} = 0.1004]
Data/restraints/parameters	11230/166/934
GOF on F^2	1.024
R_1, wR_2 [$I > 2\sigma(I)$]	R_1 = 0.0580, wR_2 = 0.1085
R_1, wR_2 (all data)	R_1 = 0.1048, wR_2 = 0.1257
CCDC number	2543037

Table S2. Crystal data and structure refinement details for **BN-DPTA2**.

Identification code	**BN-DPTA2**
Formula	$C_{62}H_{44}B_6N_{12}O_6$

Mr.	1118.063
Temperature (K)	99.94(13)
Cryst. Syst.	triclinic
Space group	*P*-1
a (Å)	13.2726(6)
b (Å)	13.6408(6)
c (Å)	17.7273(8)
α, β, γ (°)	107.5, 93.3, 104.4
Volume (Å^3)	2935.1(3)
Z	2
μ (mm^{-1})	0.082
$F_{(000)}$	1156.7
Radiation	MoKα (λ = 0.71073)
2θ (°)	4.24 to 52
Reflections collected	21256
Independent Refl.	11432 [R_{int} = 0.0493, R_{sigma} = 0.1189]
Data/restraints/parameters	11432/0/907
GOF on F^2	1.002
R_1, wR_2 [$I > 2\sigma(I)$]	R_1 = 0.0579, wR_2 = 0.0673
R_1, wR_2 (all data)	R_1 = 0.1147, wR_2 = 0.0797
CCDC number	2543038

Table S3. Crystal data and structure refinement details for **BN-BP**.

Identification code	**BN-BP**

Formula	$C_{59}H_{40}B_6N_8O_6$
Mr.	1023.86
Temperature (K)	100.00
Cryst. Syst.	monoclinic
Space group	$P2_1/c$
a (Å)	24.042(3)
b (Å)	8.4996(8)
c (Å)	26.442(4)
α, β, γ (°)	90, 97.2, 90
Volume (Å^3)	5360.7(12)
Z	4
μ (mm^{-1})	0.082
$F_{(000)}$	2120.0
Radiation	MoKα (λ = 0.71073)
2θ(°)	3.414 to 50
Reflections collected	100794
Independent Refl.	9404 [R_{int} = 0.0722, R_{sigma} = 0.0333]
Data/restraints/parameters	9404/50/791
GOF on F^2	1.016
R_1, wR_2 [I >2σ(I)]	R_1 = 0.0384, wR_2 = 0.0948
R_1, wR_2 (all data)	R_1 = 0.0515, wR_2 = 0.1049
CCDC number	2543039

Table S4. Crystal data and structure refinement details for **BN-Salen-Ni**.

Identification code	**BN-Salen-Ni**
Formula	$C_{88}H_{68}B_6N_{10}NiO_8$
Mr.	1517.09
Temperature (K)	100.15
Cryst. Syst.	monoclinic
Space group	*I*2/a
a (Å)	16.1375(5)
b (Å)	24.7146(7)
c (Å)	22.1396(7)
α, β, γ (°)	90, 110.18, 90
Volume (Å^3)	8288.0(5)
Z	4
μ (mm^{-1})	0.295
$F_{(000)}$	3152.0
Radiation	MoKα (λ = 0.71073)
2θ(°)	3.92 to 50.998
Reflections collected	22853
Independent Refl.	7705 [R_{int} = 0.0484, R_{sigma} = 0.0630]
Data/restraints/parameters	7705/261/577
GOF on F^2	1.088
R_1, wR_2 [I >2σ(I)]	R_1 = 0.0643, wR_2 = 0.1361
R_1, wR_2 (all data)	R_1 = 0.0835, wR_2 = 0.1425
CCDC number	2553423

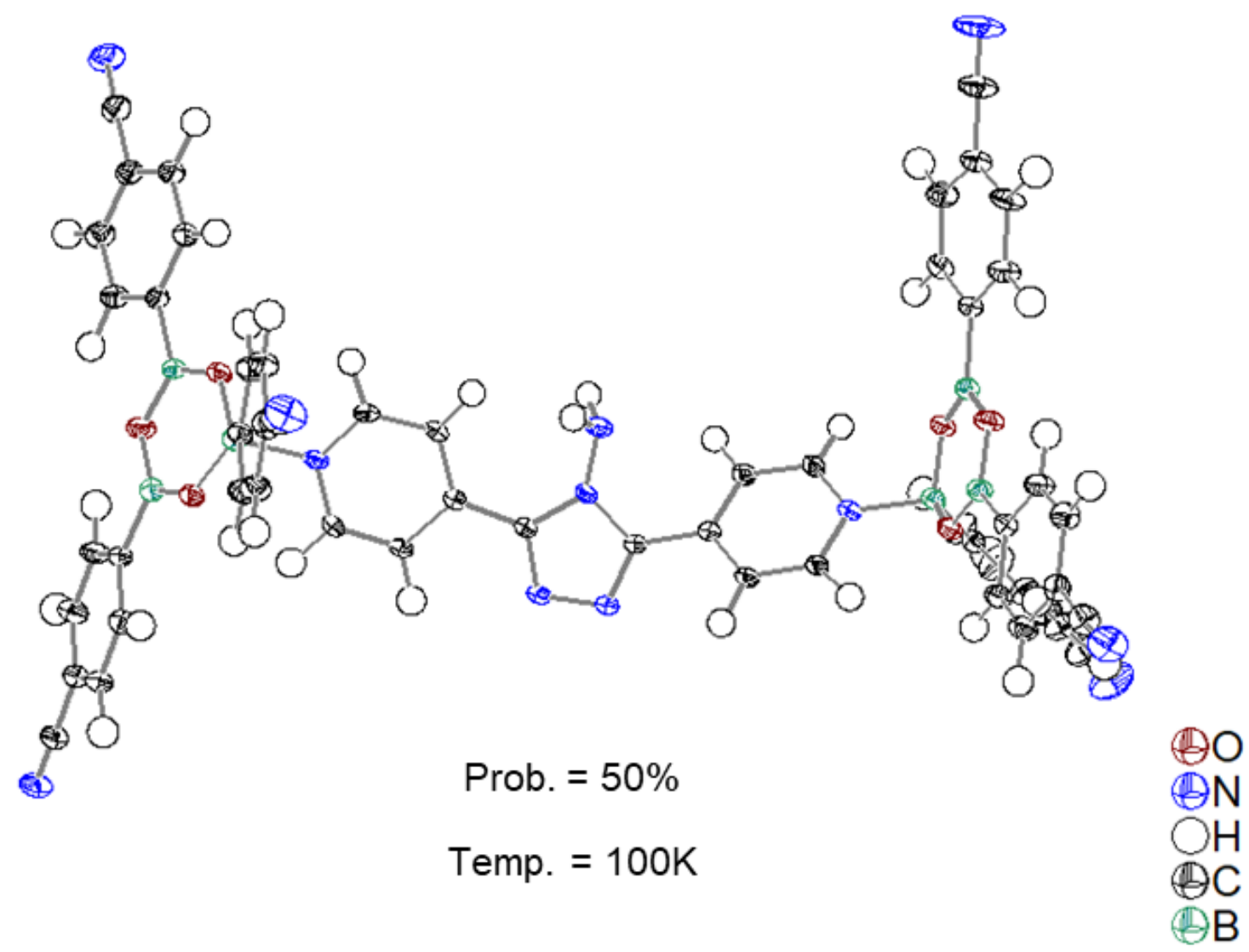

Figure S1. SC-XRD structure of **BN-DPTA1**, drawn with thermal ellipsoids at the 50% probability level.

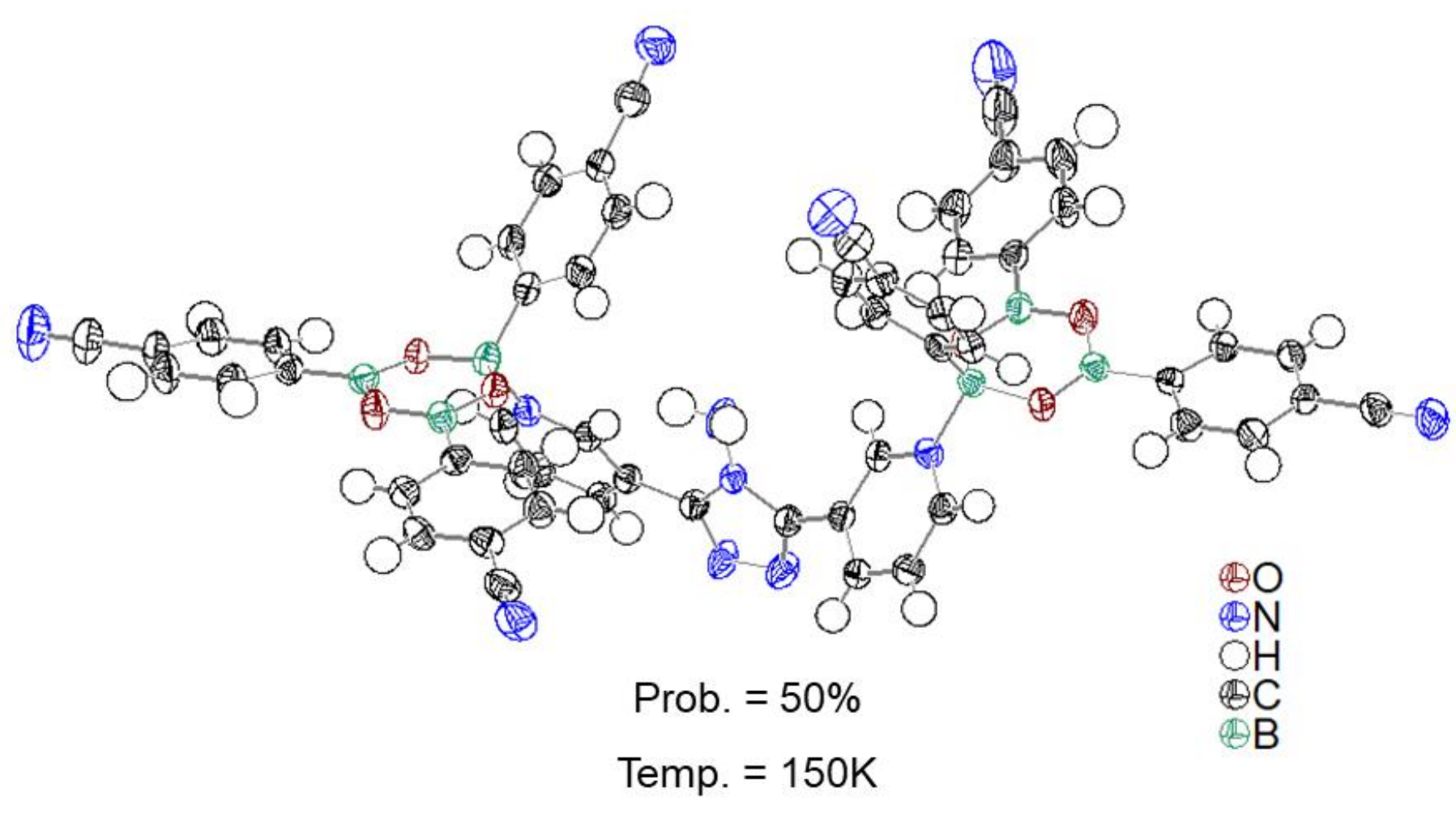

Figure S2. SC-XRD structure of **BN-DPTA2**, drawn with thermal ellipsoids at the 50% probability level.

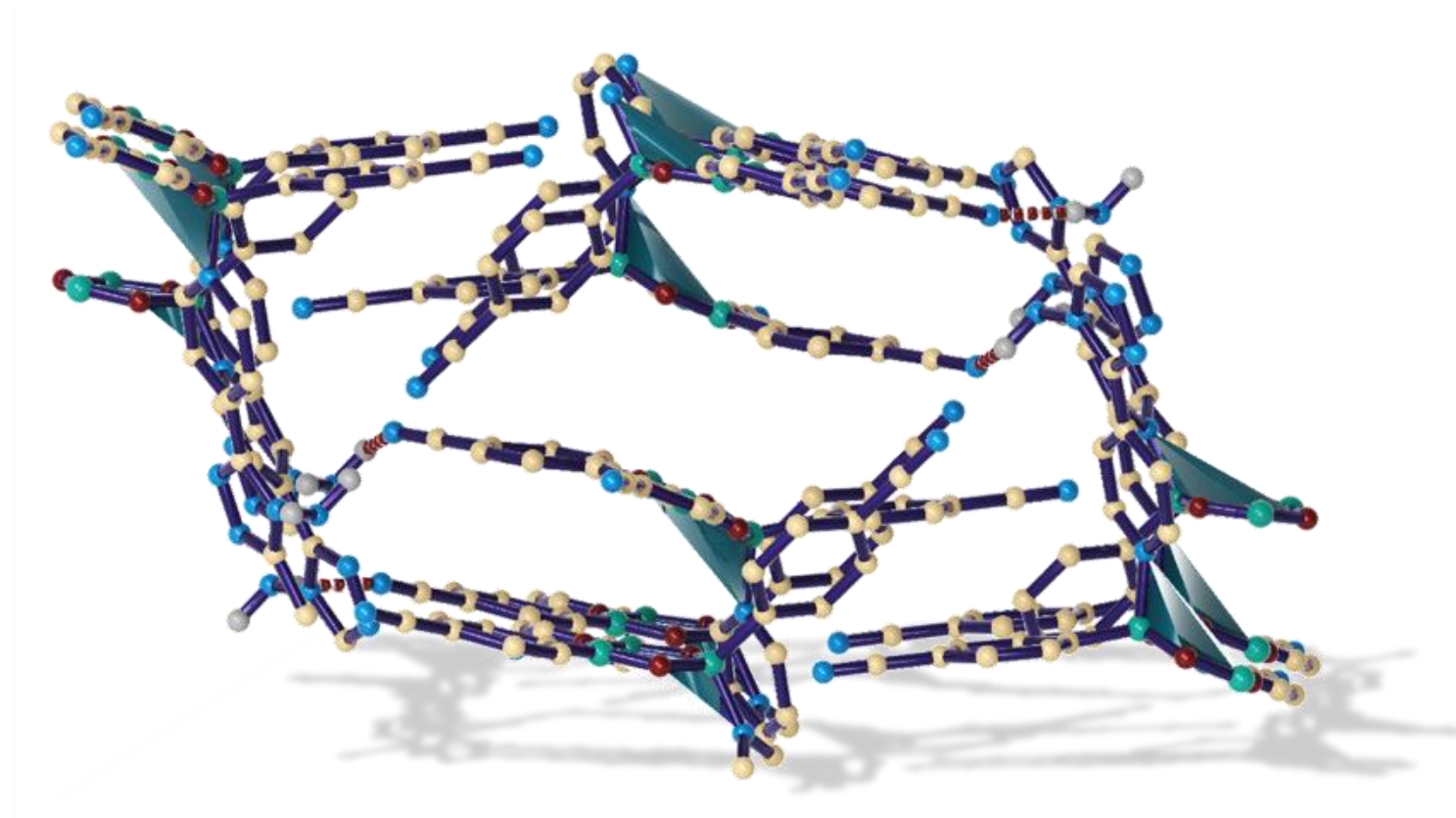

Figure S3. Three-dimensional cavity of **BN-DPTA1**.

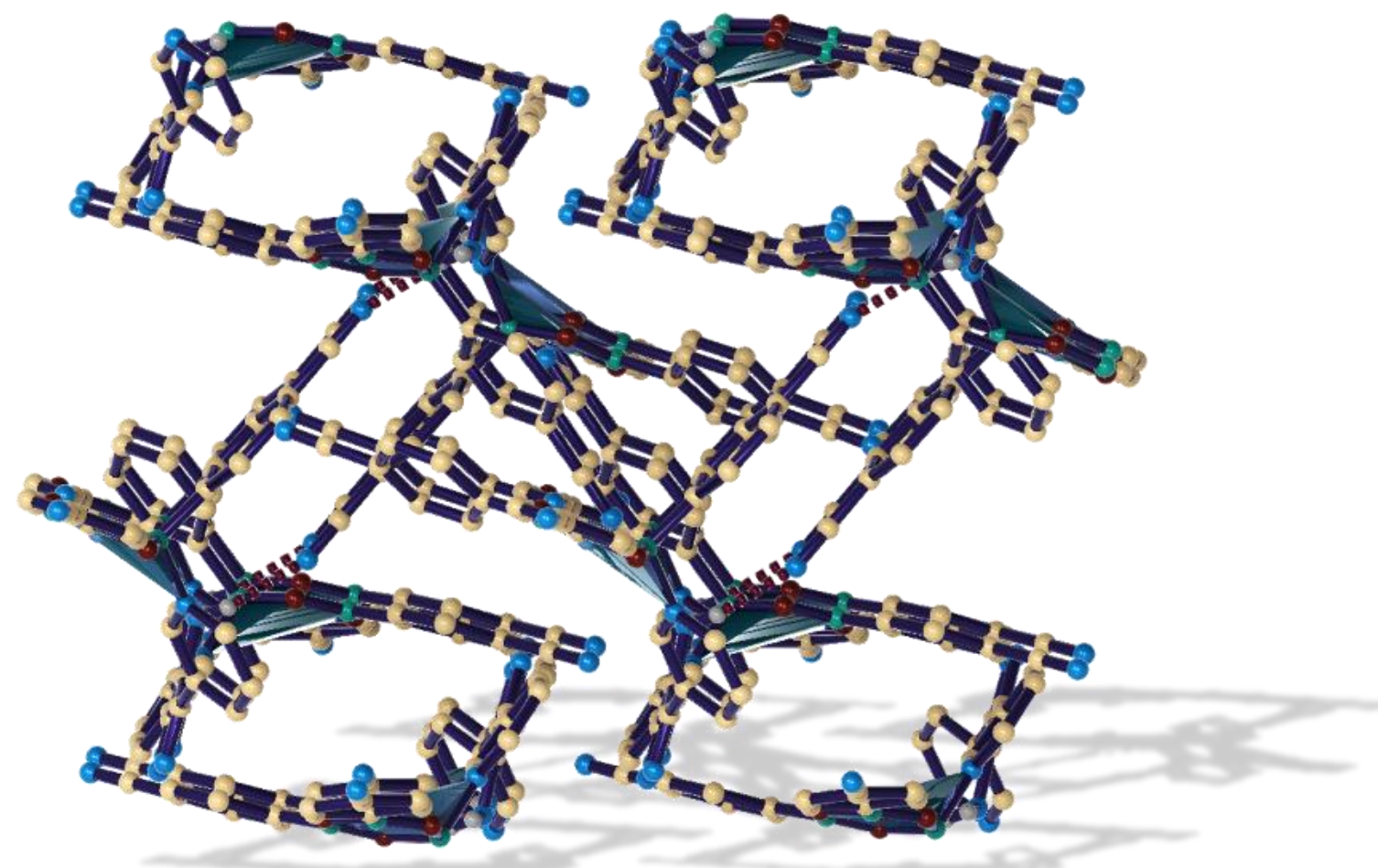

Figure S4. Three-dimensional cavity of **BN-DPTA2**

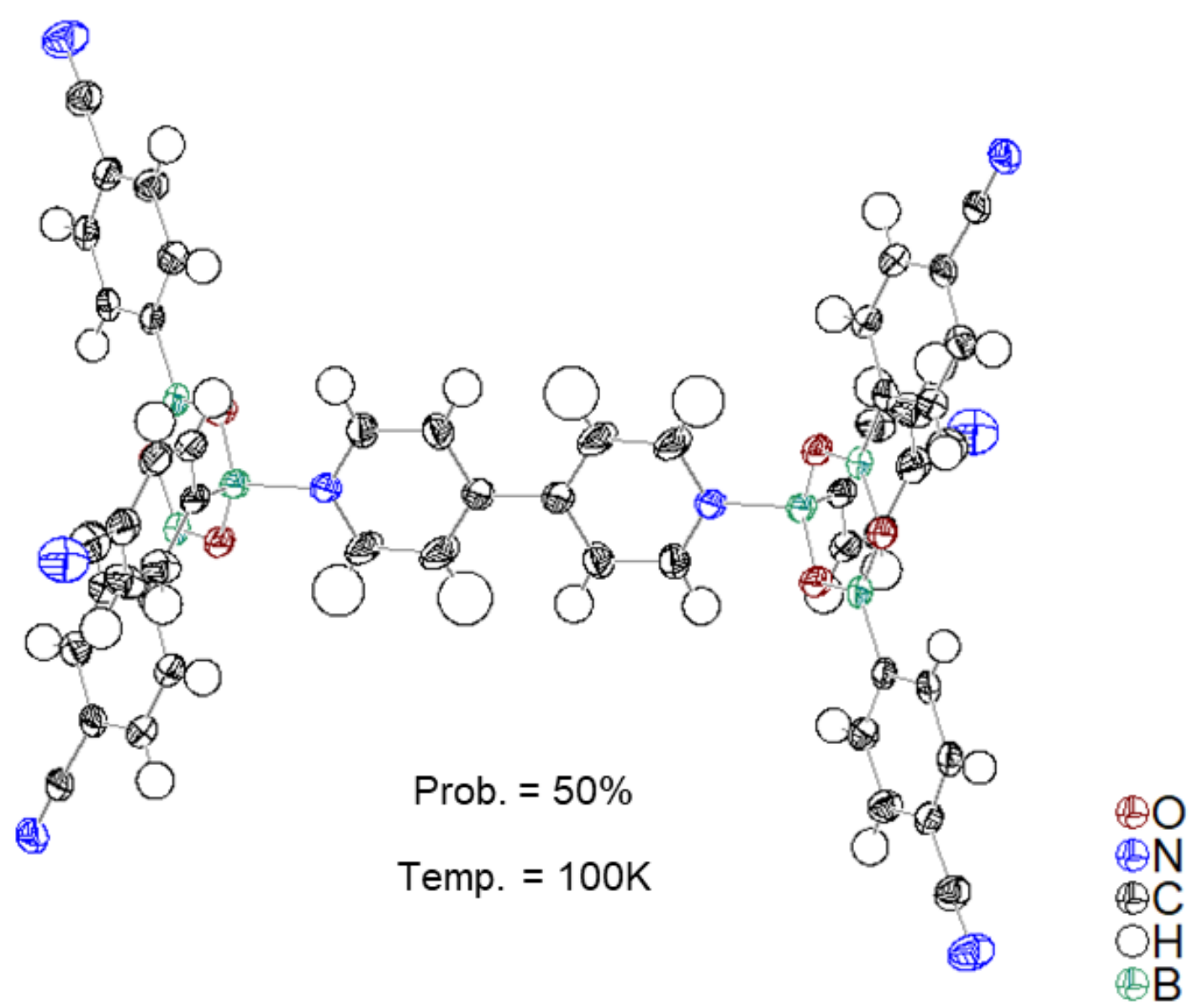

Figure S5. SC-XRD structure of **BN-BP**, drawn with thermal ellipsoids at the 50% probability level.

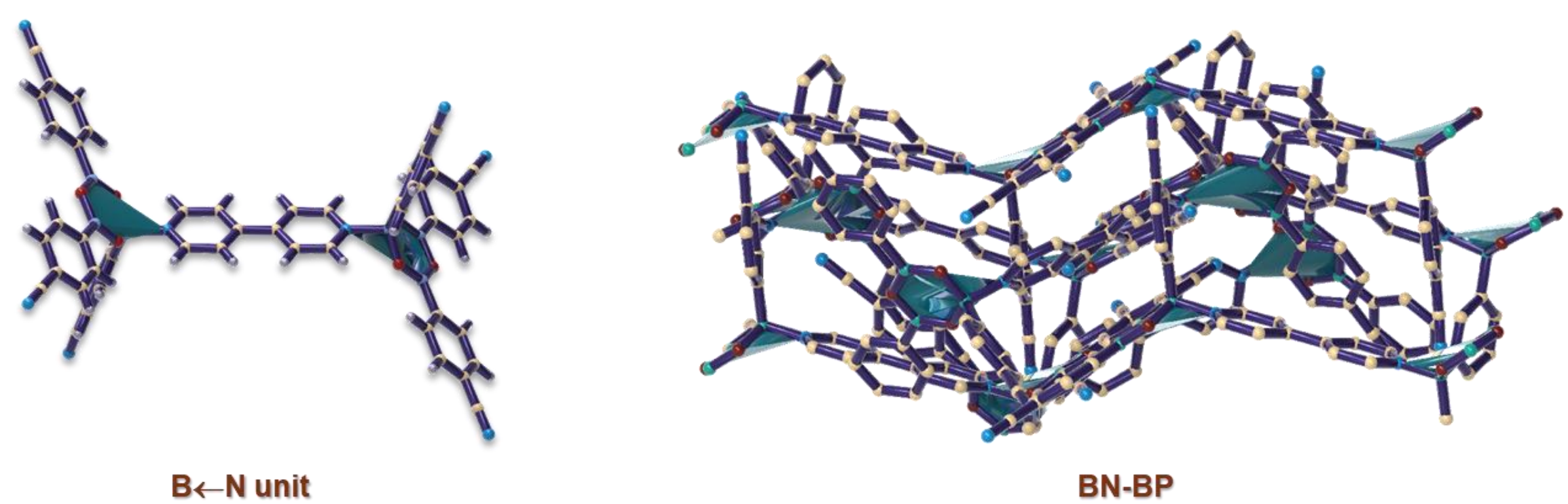

Figure S6. SC-XRD structure of B←N unit and 3D structure of **BN-BP**.

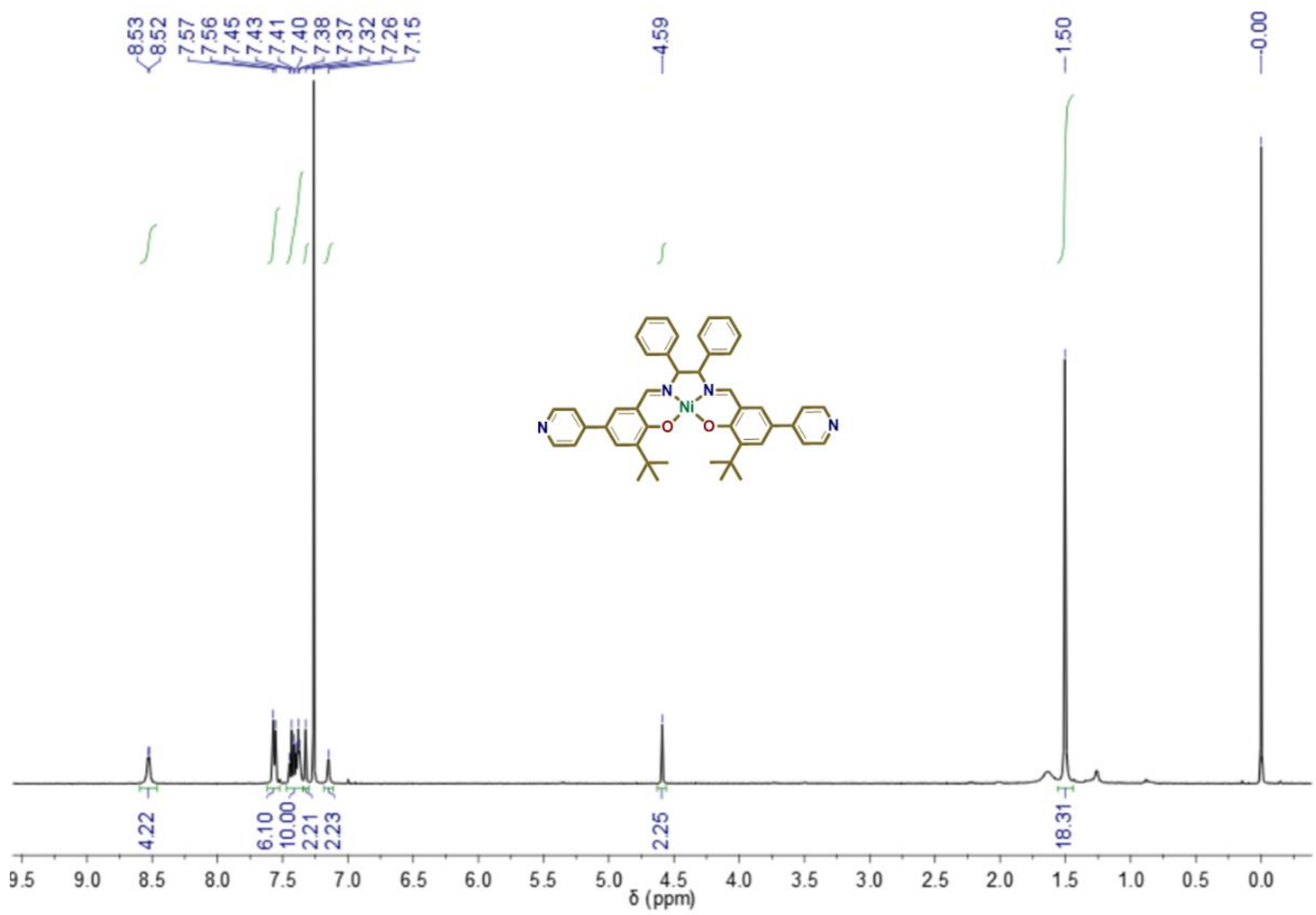

Figure S7. ^{1}H NMR spectrum of **Salen-Ni** ligand ($CDCl_3$, 400 MHz, 298K).

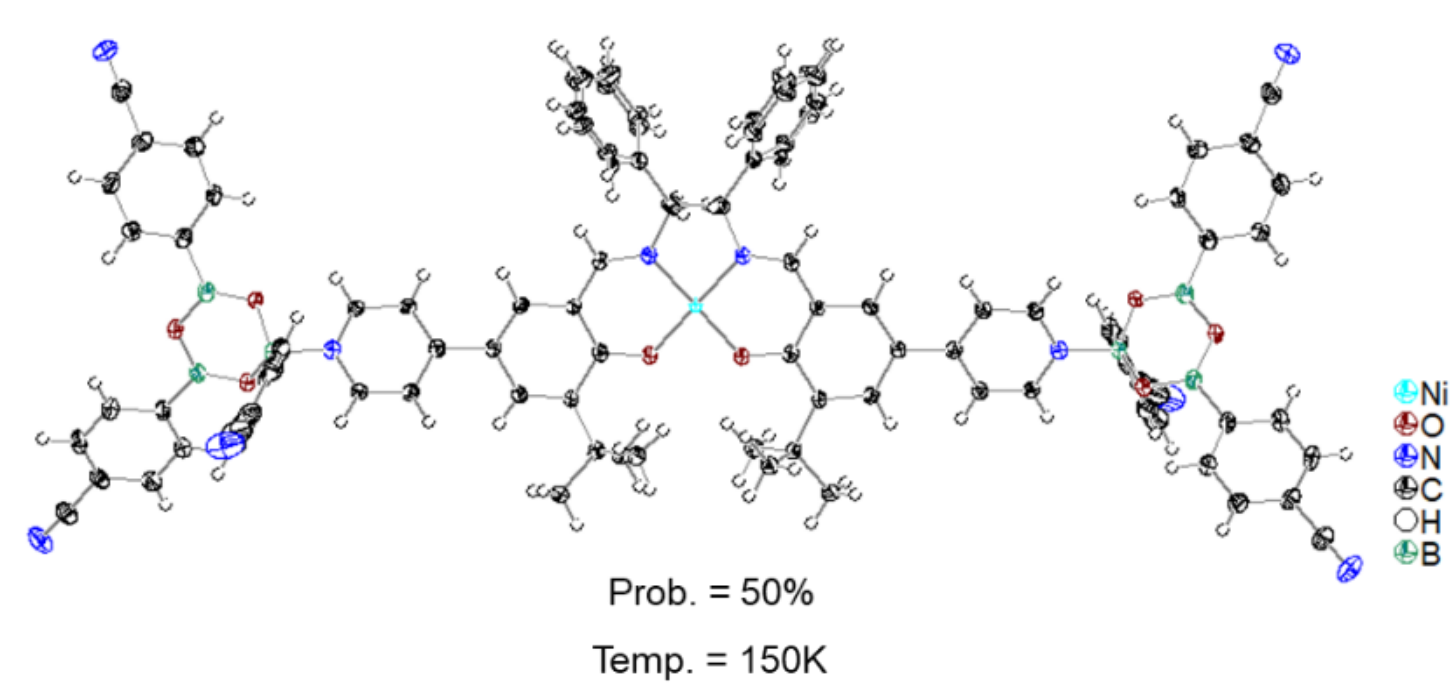

Figure S8. SC-XRD structure of **BN-Salen-Ni**, drawn with thermal ellipsoids at the 50% probability level.

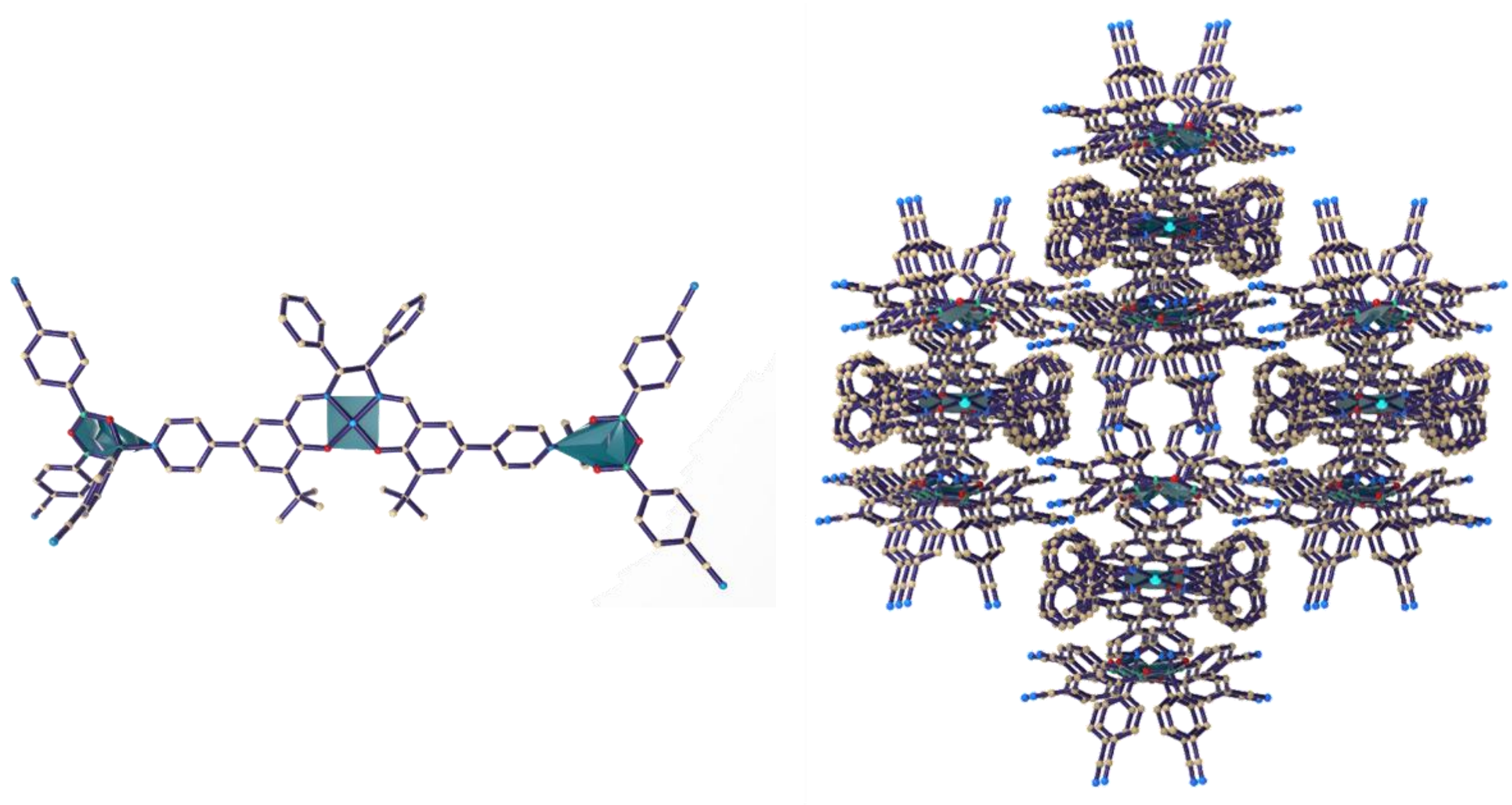

B←N unit BN-Salen-Ni

Figure S9. SC-XRD structure of B←N unit and 3D structure of **BN-Salen-Ni**.

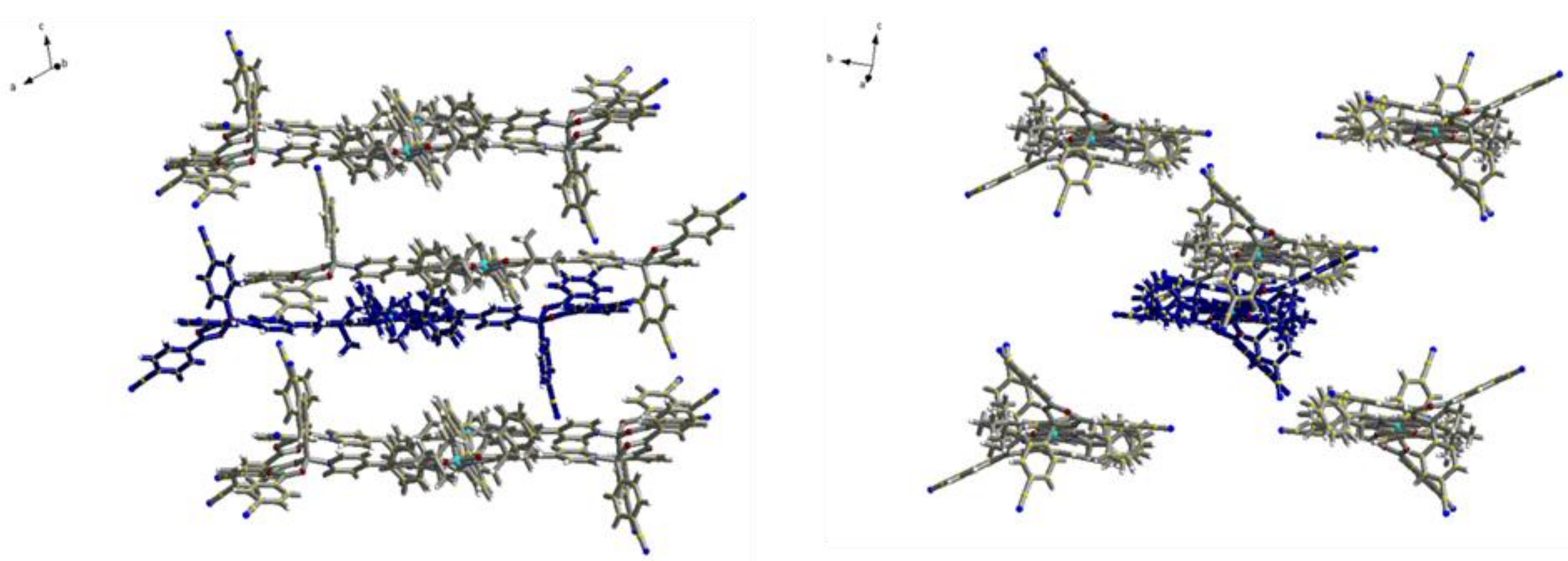

Figure S10. Assembly mode in BN-Salen-Ni showing no further connections among different B←N units.

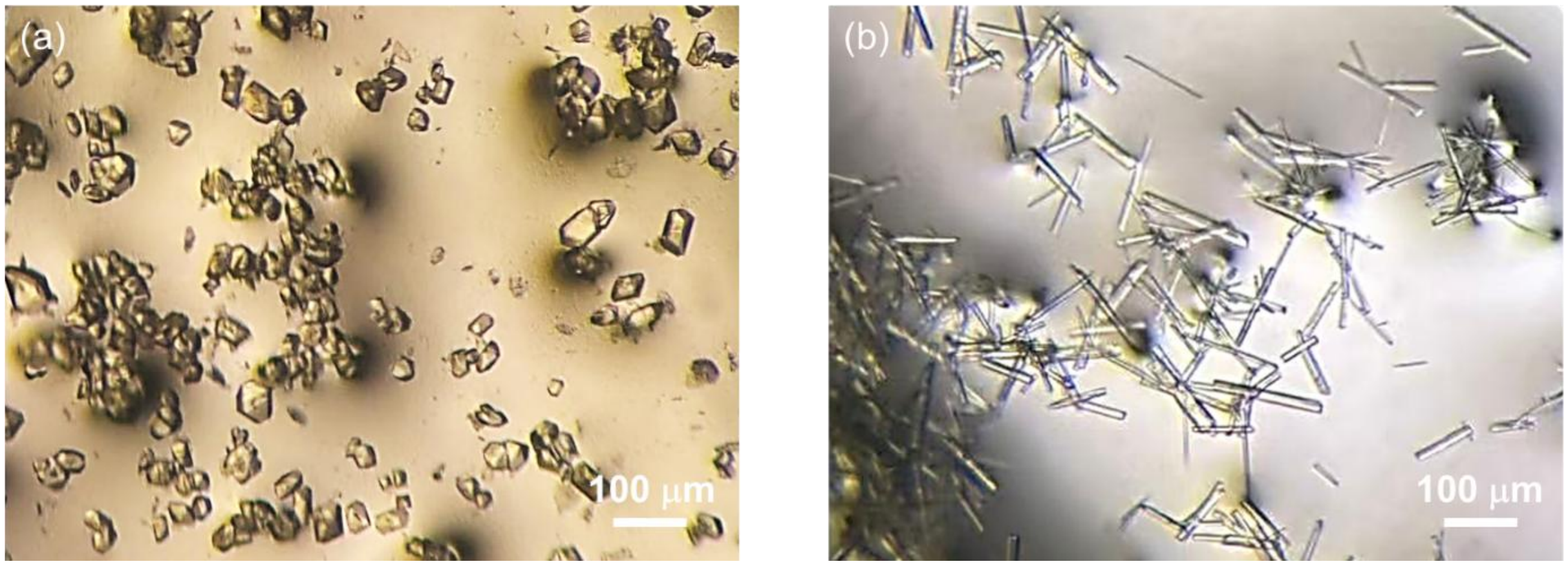

Figure S11. Digital photos of (a) **BN-DPTA1** and (b) **BN-DPTA2**.

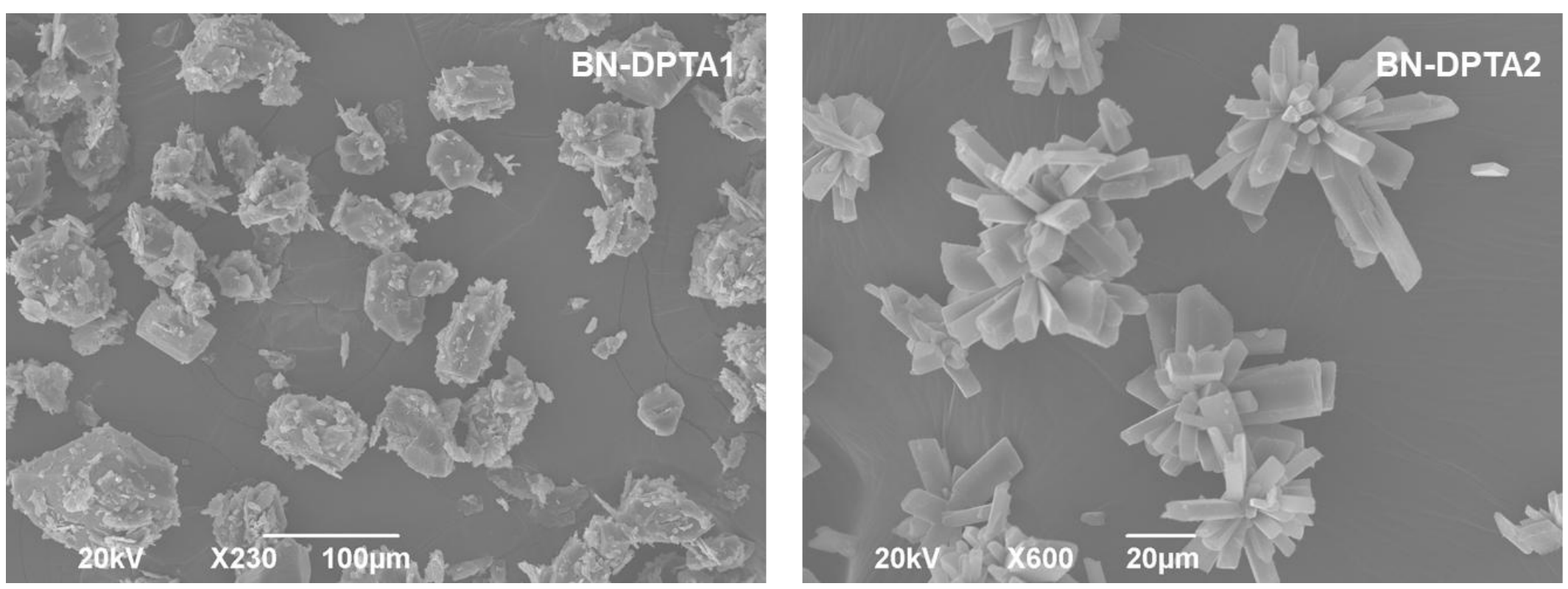

Figure S12. SEM images of **BN-DPTA1** and **BN-DPTA2**.

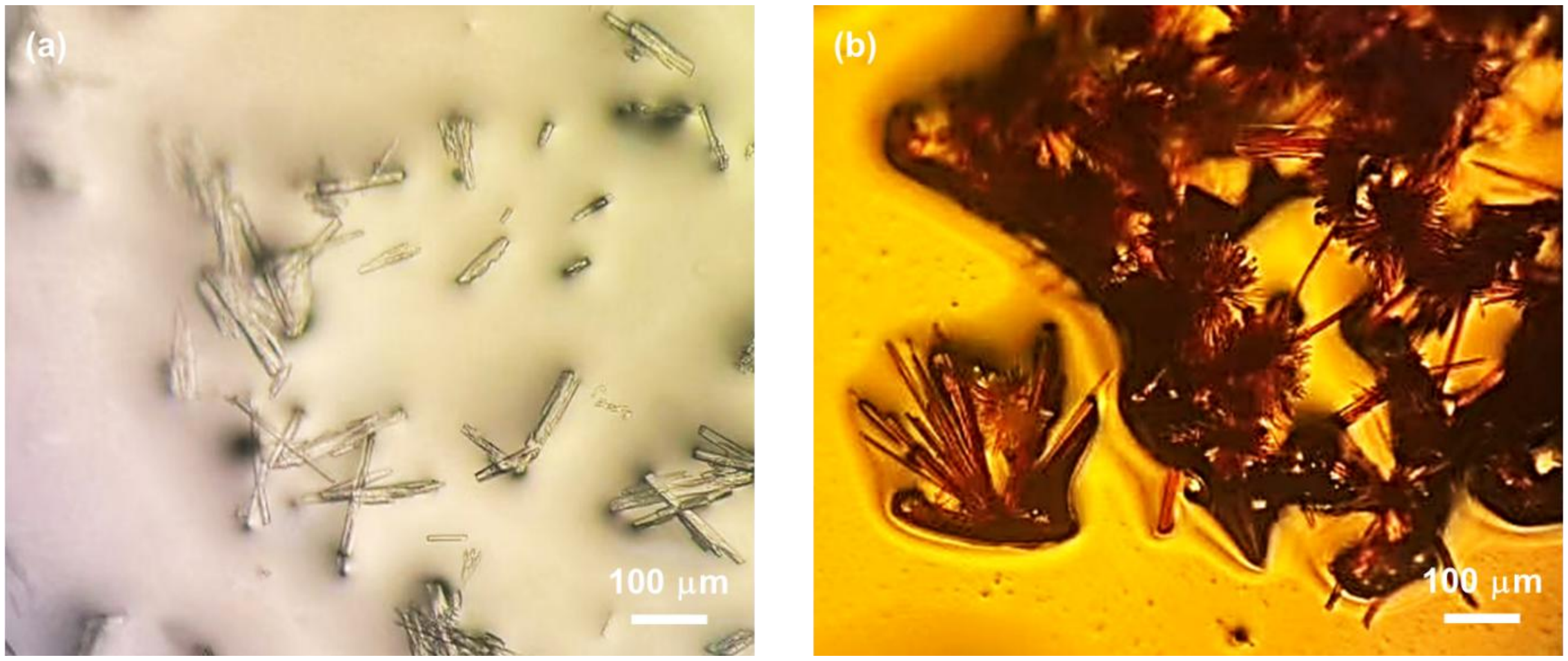

Figure S13. Digital photos of (a) **BN-BP** and (b) **BN-Salen-Ni**.

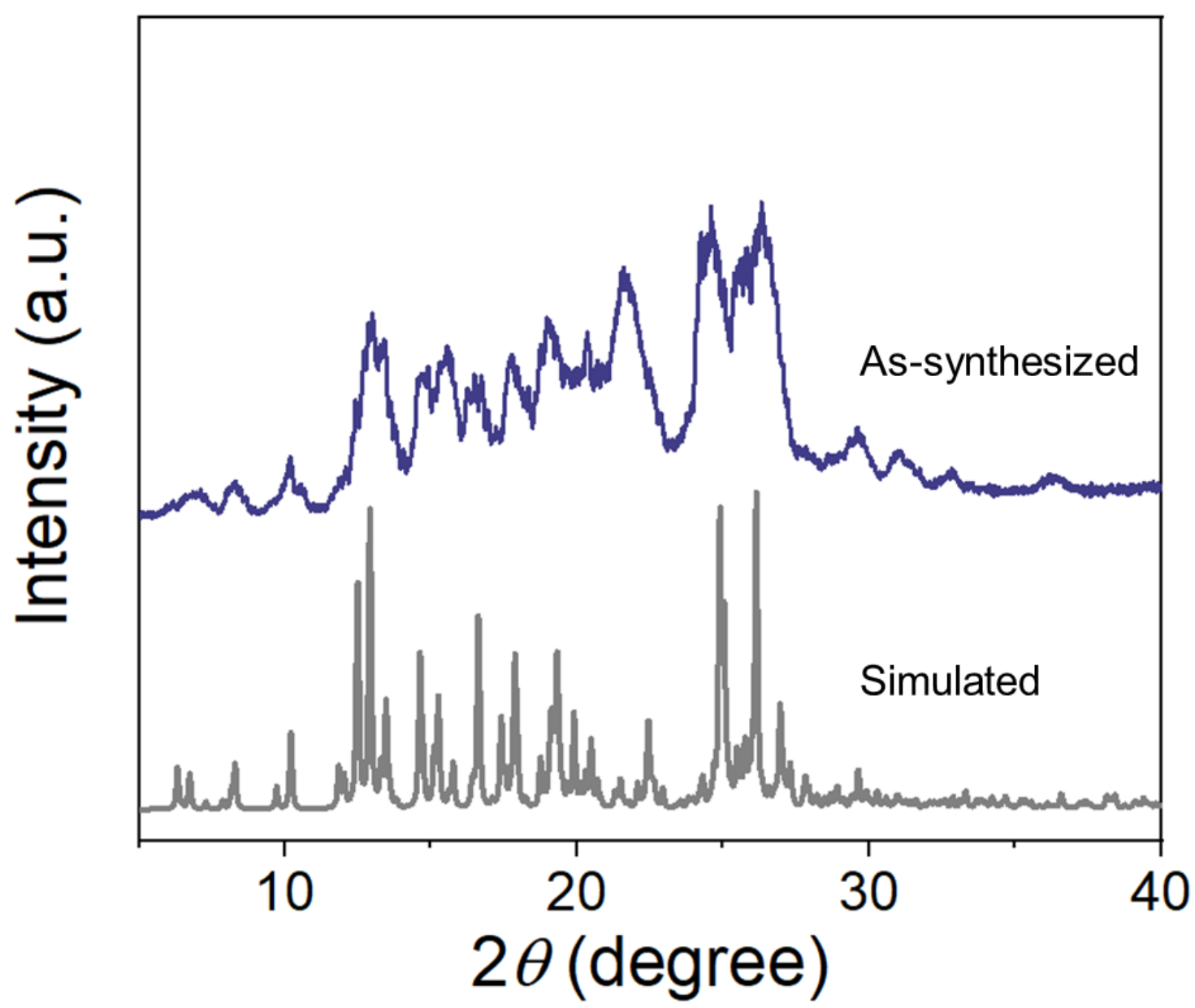

Figure S14. Experimental PXRD pattern of as-synthesized **BN-DPTA1** compared with the simulated pattern derived from the SC-XRD data.

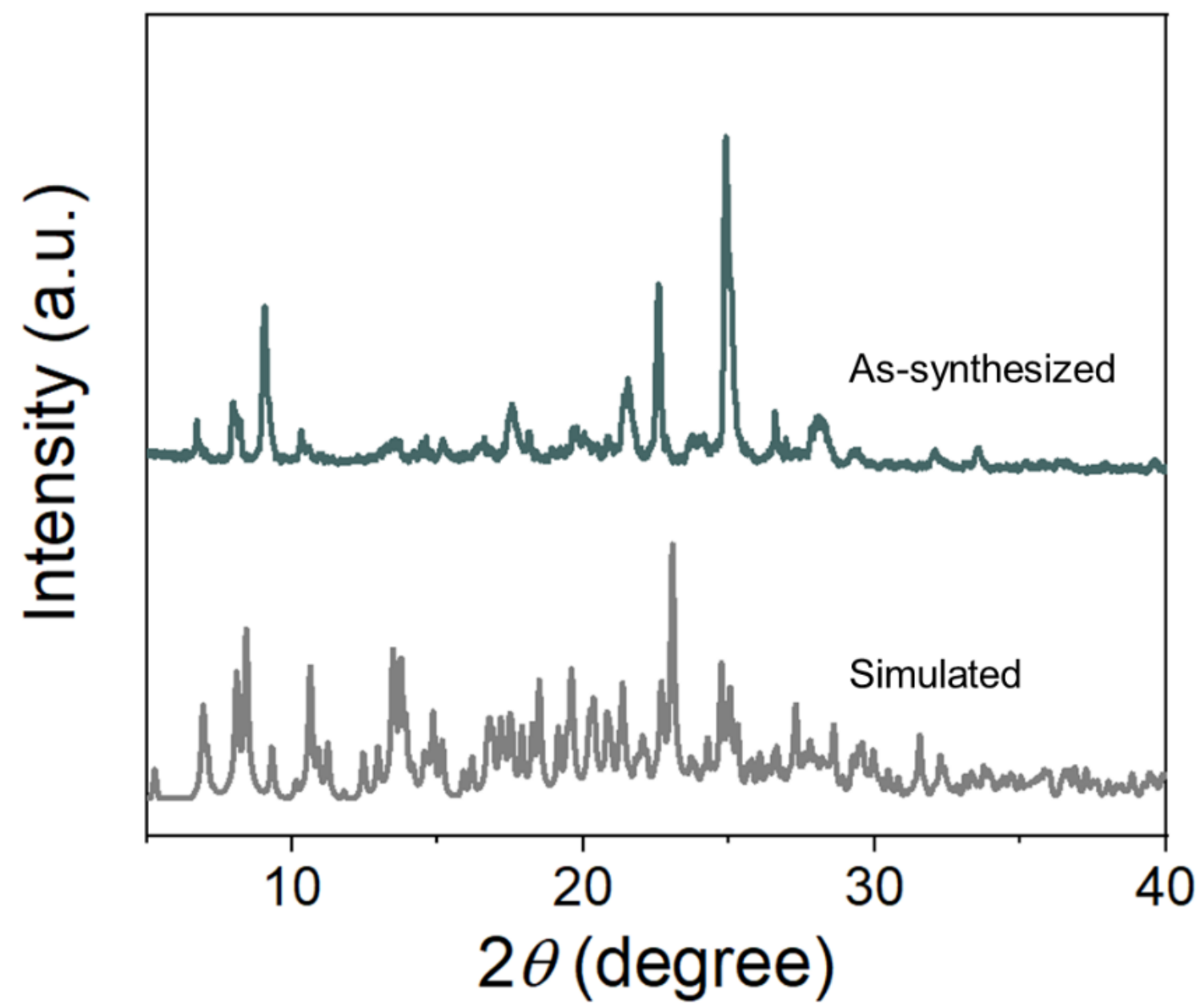

Figure S15. Experimental PXRD pattern of as-synthesized **BN-DPTA2** compared with the simulated pattern derived from the SC-XRD data.

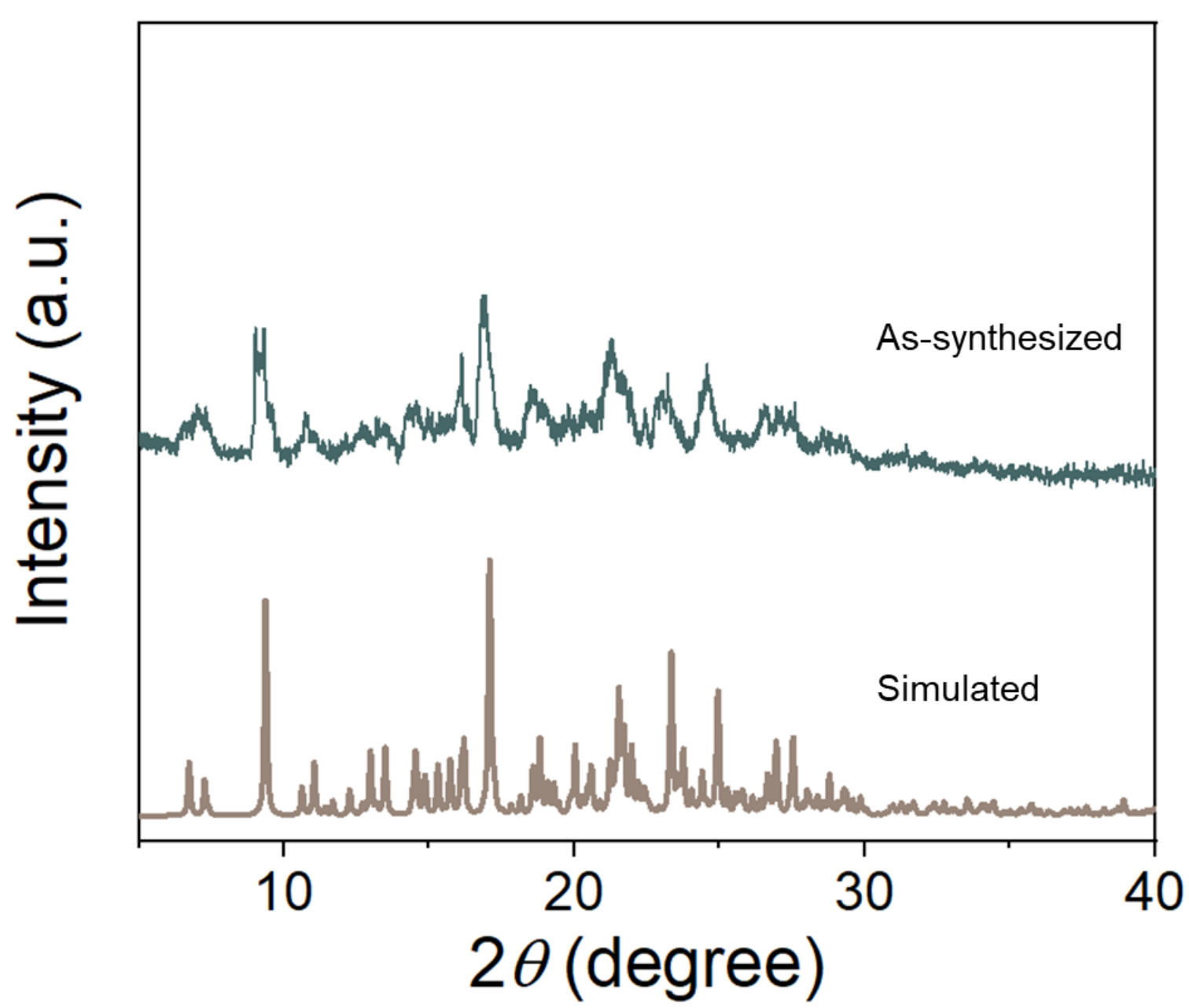

Figure S16. Experimental PXRD pattern of as-synthesized **BN-BP** compared with the simulated pattern derived from the SC-XRD data.

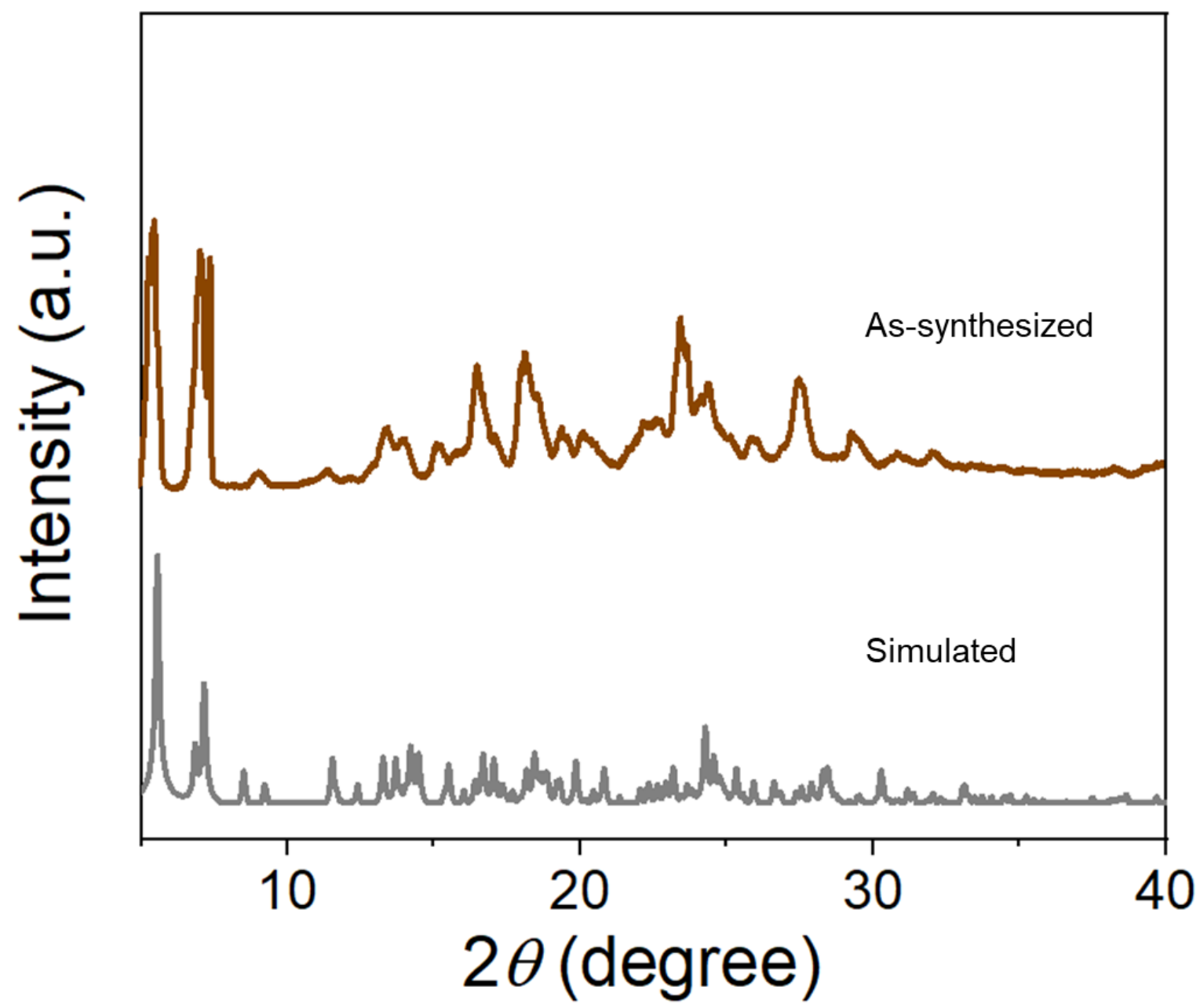

Figure S17. Experimental PXRD pattern of as-synthesized **BN-Salen-Ni** compared with the simulated pattern derived from the SC-XRD data.

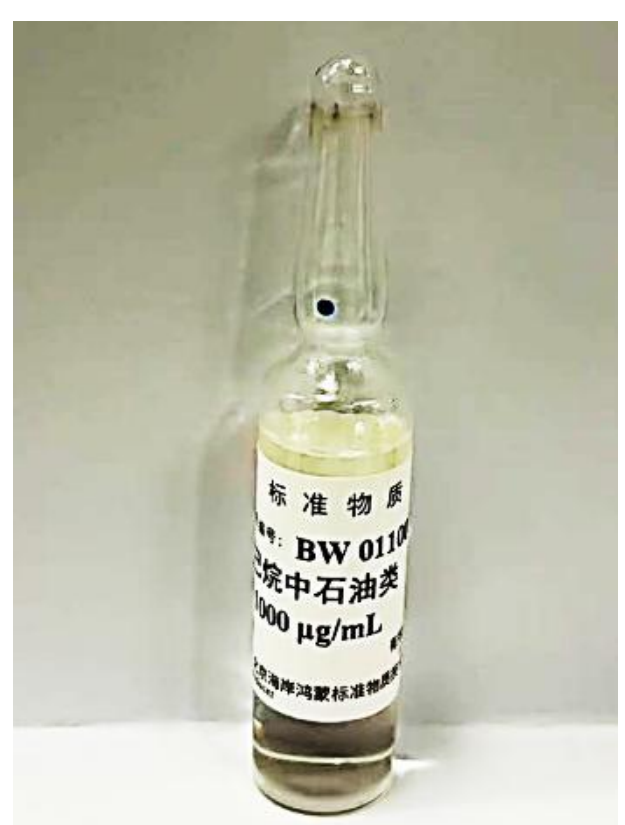

Figure S18. Digital photo of the purchased light oil standard.

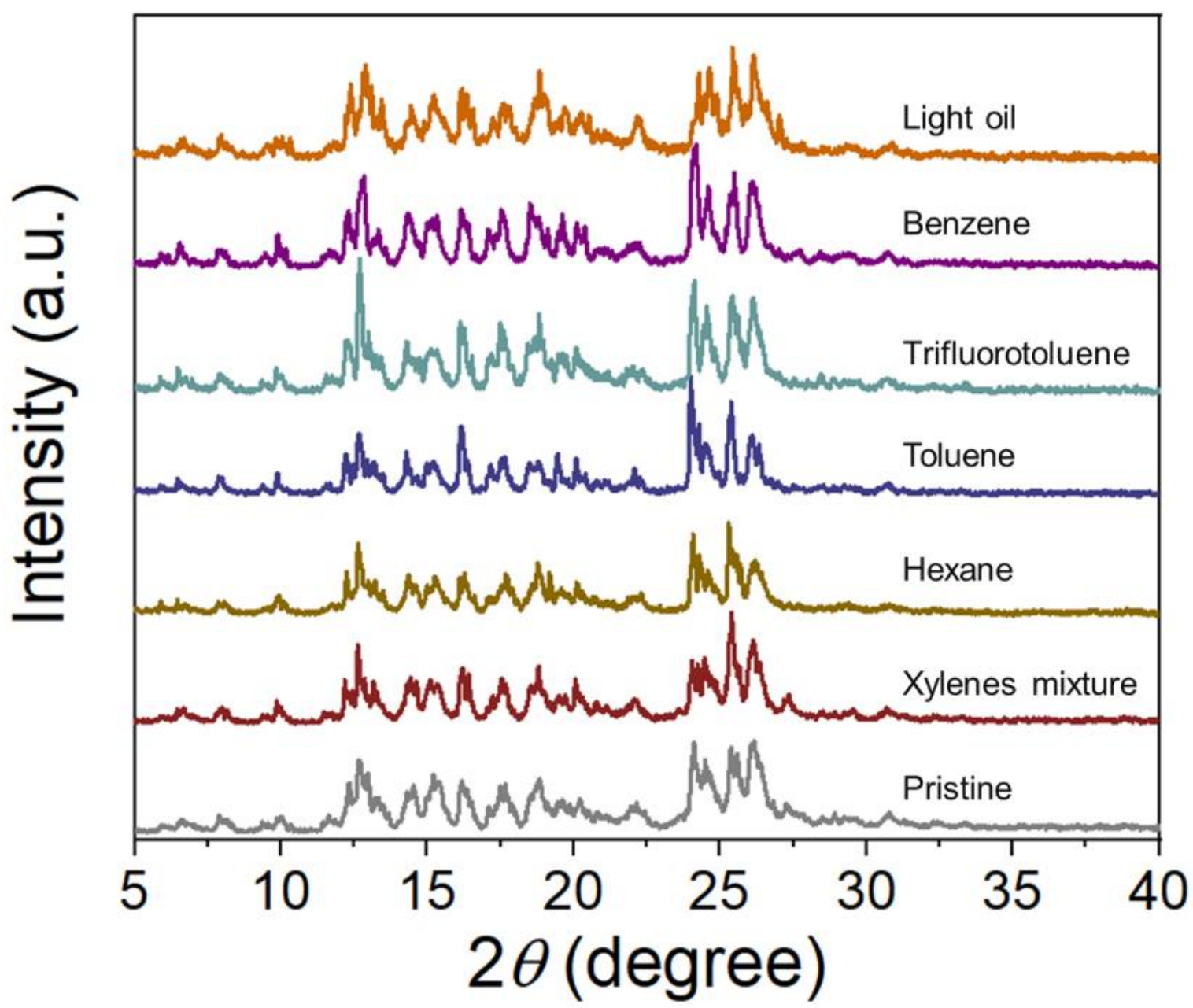

Figure S19. PXRD patterns of **BN-DPTA1** after soaking in different solvent for 3 days.

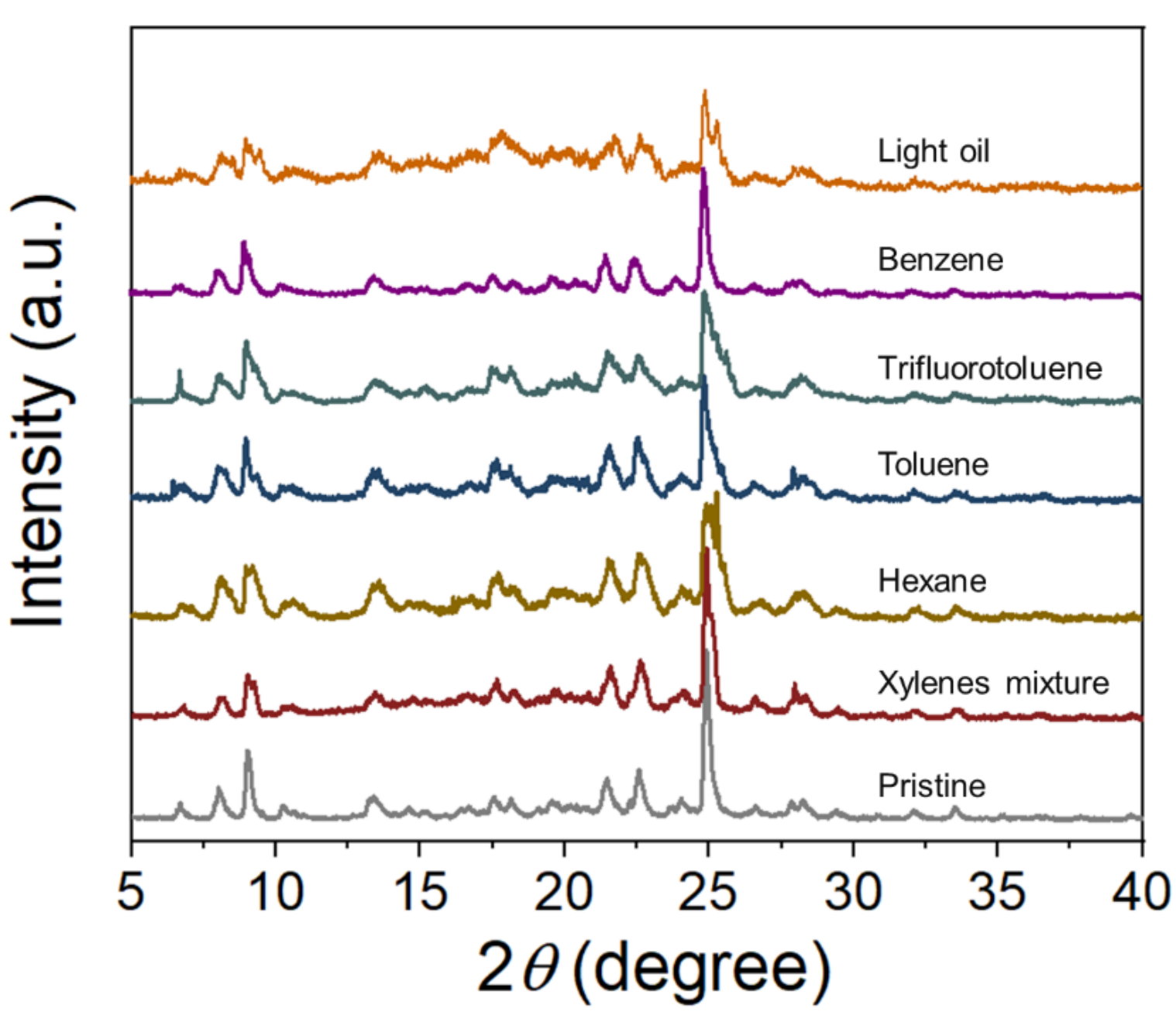

Figure S20. PXRD patterns of **BN-DPTA2** after soaking in different solvent for 3 days.

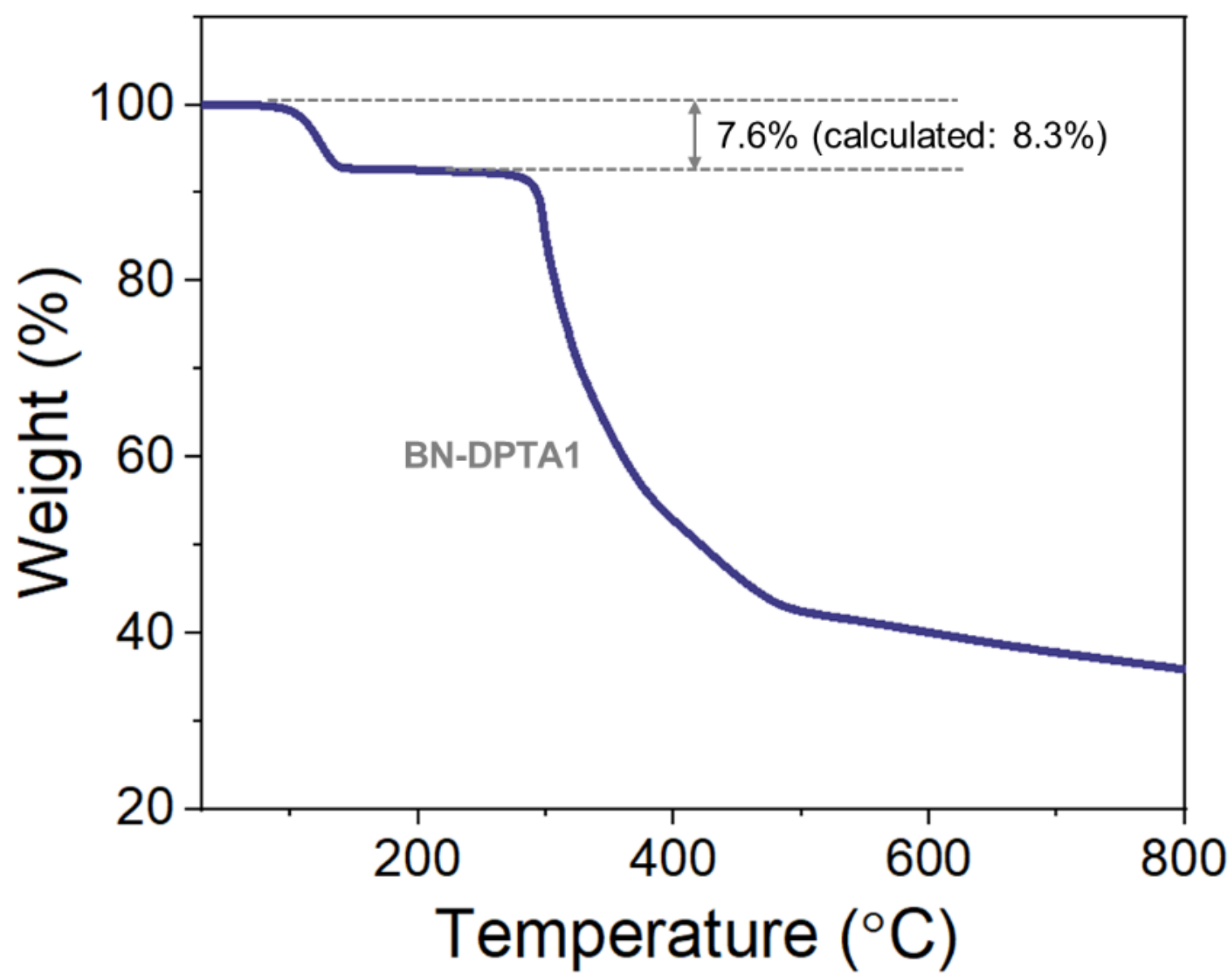

Figure S21. TGA curve of **BN-DPTA1**.

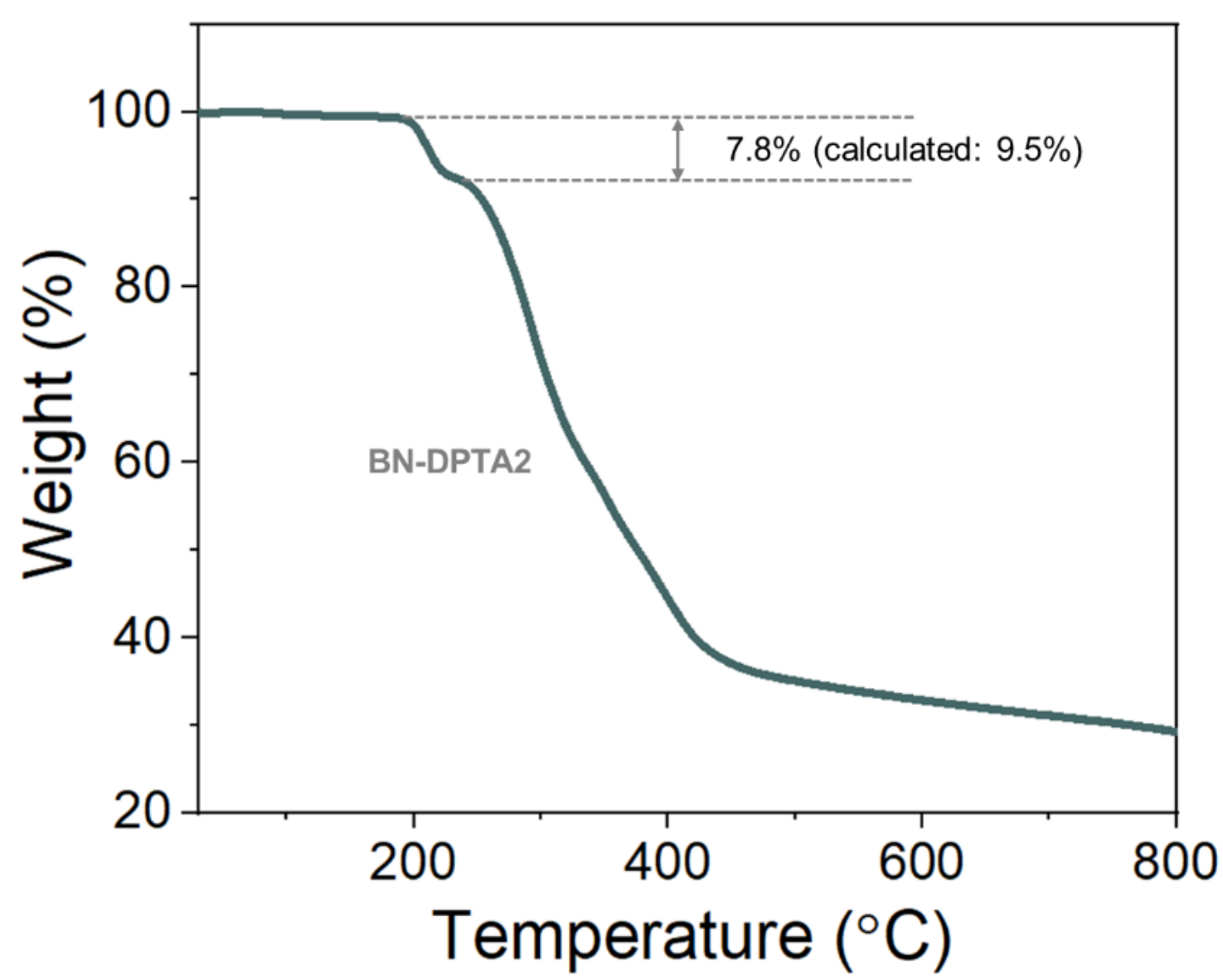

Figure S22. TGA curve of **BN-DPTA2**.

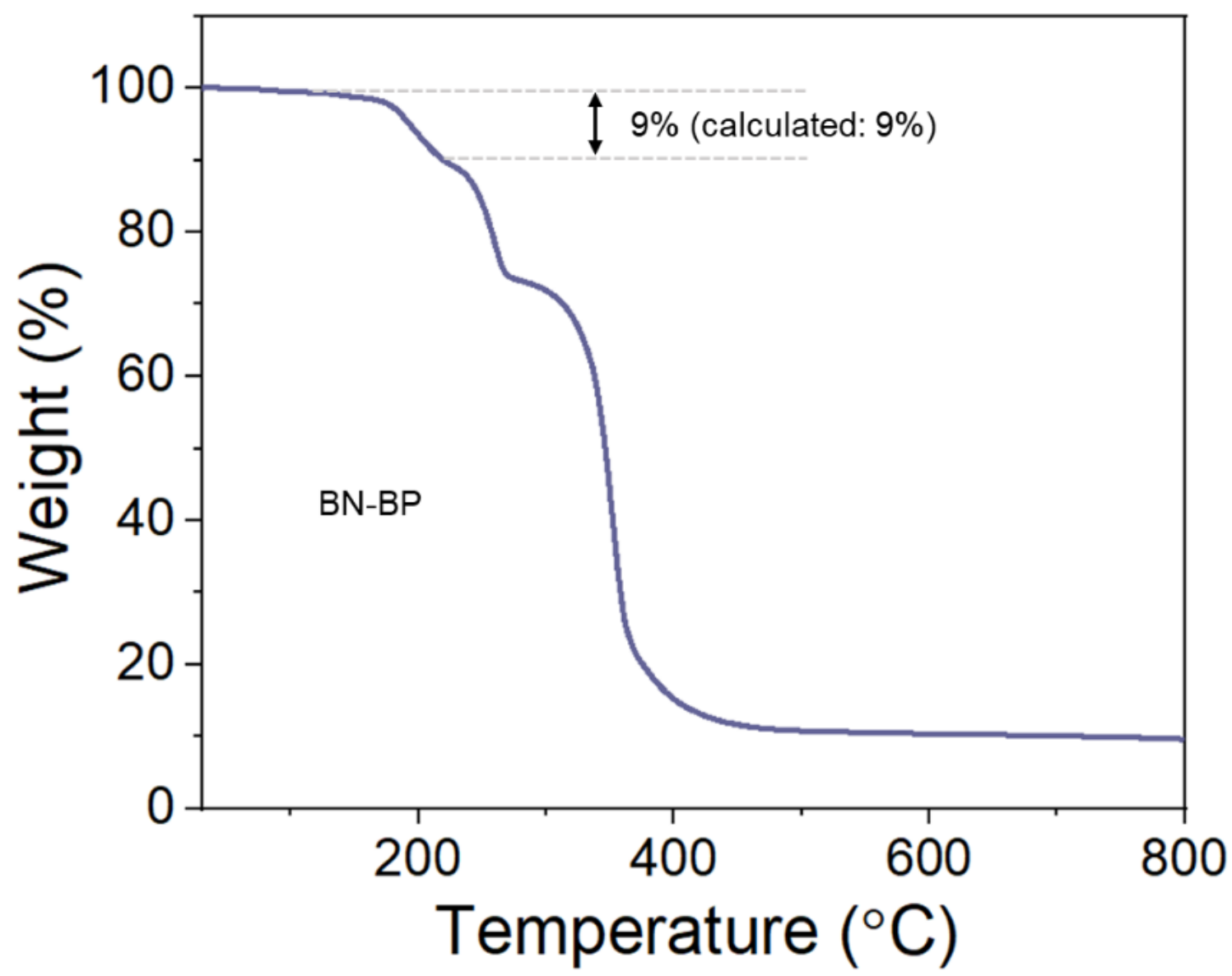

Figure S23. TGA curve of **BN-BP**.

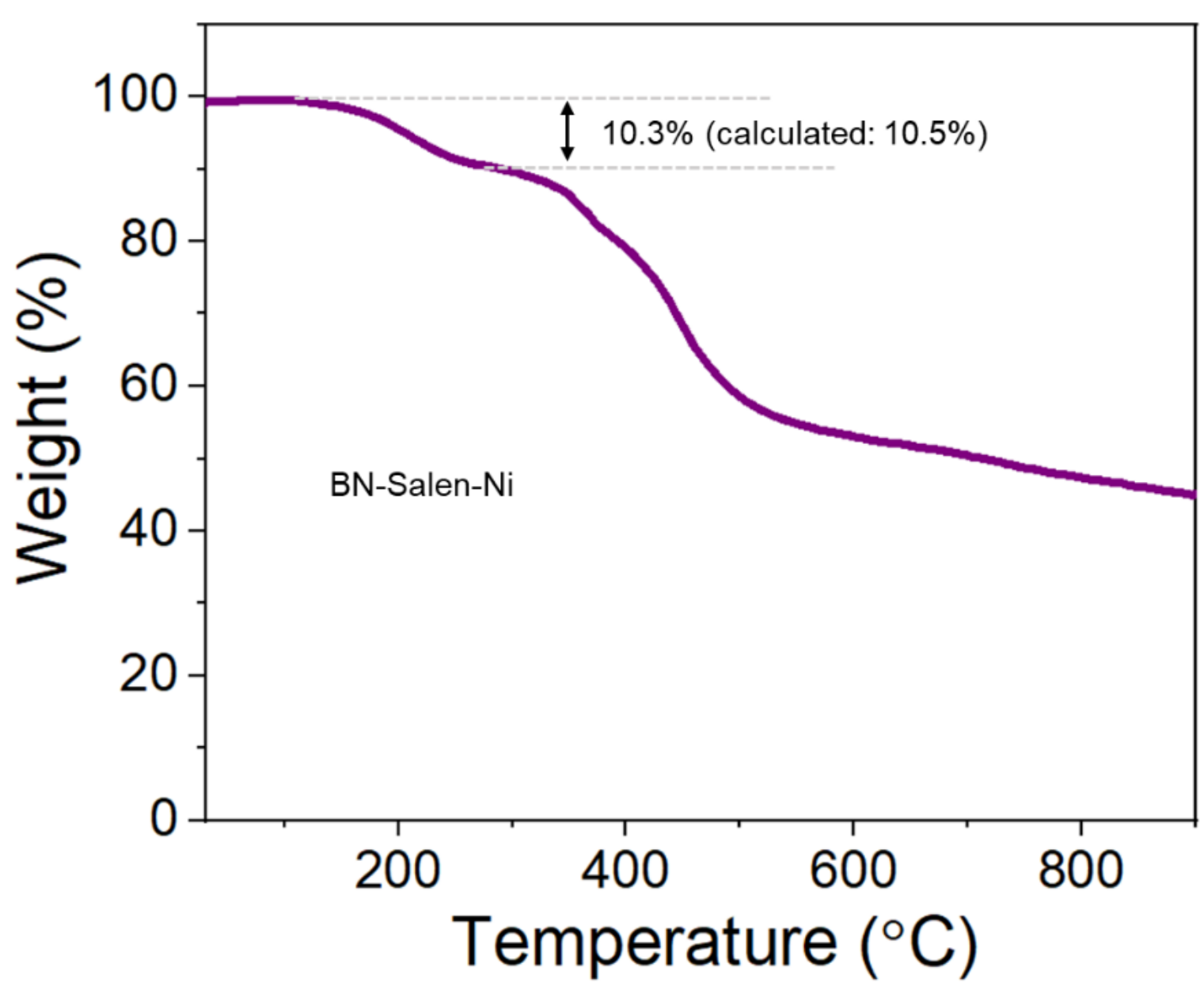

Figure S24. TGA curve of **BN-Salen-Ni**.

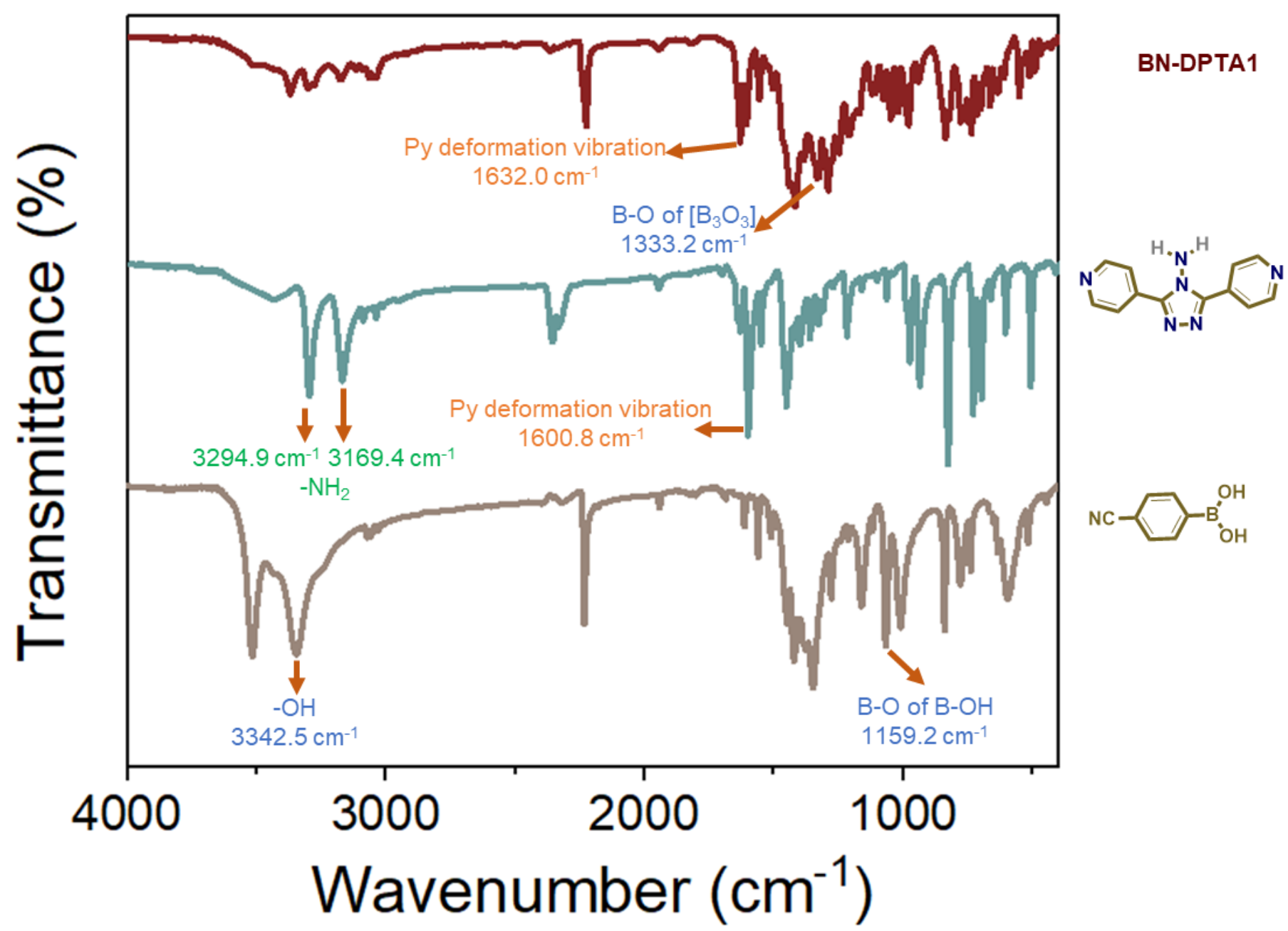

Figure S25. FT-IR spectra of 4-cyanophenylboronic acid, DPTA1, and **BN-DPTA1**, respectively. The complete disappearance of the O–H stretching band from 4-cyanophenylboronic acid at 3342.5 cm^{-1} in the spectrum of **BN-DPTA1** confirms the condensation of boronic acid groups. This is further supported by the B–O stretching vibration, which shifts from 1159.2 cm^{-1} in the monomer to a higher frequency of 1333.2 cm^{-1} in **BN-DPTA1** because of the formation of [B_3O_3] ring. The successful coordination between DPTA1 and the boroxine ring is evidenced by the pyridine ring vibration. This band shifts hypsochromically from 1600.8 cm^{-1} in free DPTA1 to 1632.0 cm^{-1} in **BN-DPTA1**. The sharp doublet characteristic of the –NH_2 stretching modes in DPTA1 (3294.9 and 3169.4 cm^{-1}) merges into broadened band in **BN-DPTA1**, suggesting that the amino groups are engaged in hydrogen bonding in the framework.

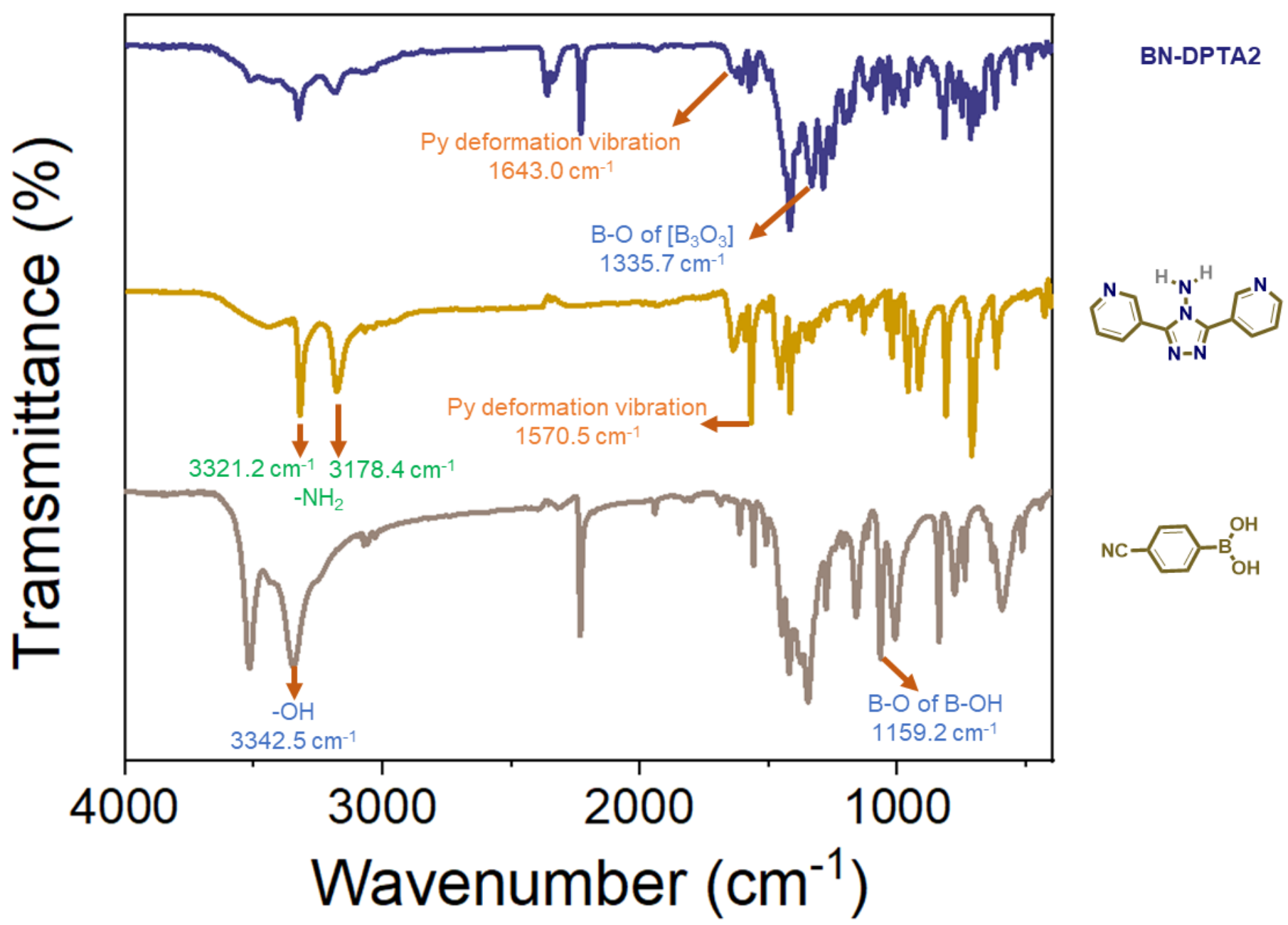

Figure S26. FT-IR spectra of 4-cyanophenylboronic acid, DPTA2, and **BN-DPTA2**, respectively. The complete disappearance of the O–H stretching band from 4-cyanophenylboronic acid at 3342.5 cm⁻¹ in the spectrum of **BN-DPTA2** confirms the condensation of boronic acid groups. This is further supported by the B–O stretching vibration, which shifts from 1159.2 cm^{-1} in the monomer to a higher frequency of 1335.7 cm^{-1} in **BN-DPTA2** because of the formation of [B_3O_3] ring. The successful coordination between DPTA2 and the boroxine ring is evidenced by the pyridine ring vibration. This band shifts hypsochromically from 1570.5 cm^{-1} in free DPTA2 to 1643.0 cm^{-1} in **BN-DPTA2**. The sharp doublet characteristic of the –NH_2 stretching modes in DPTA2 (3321.2 and 3178.4 cm^{-1}) merges into broadened band in **BN-DPTA2**, suggesting that the amino groups are engaged in hydrogen bonding in the framework.

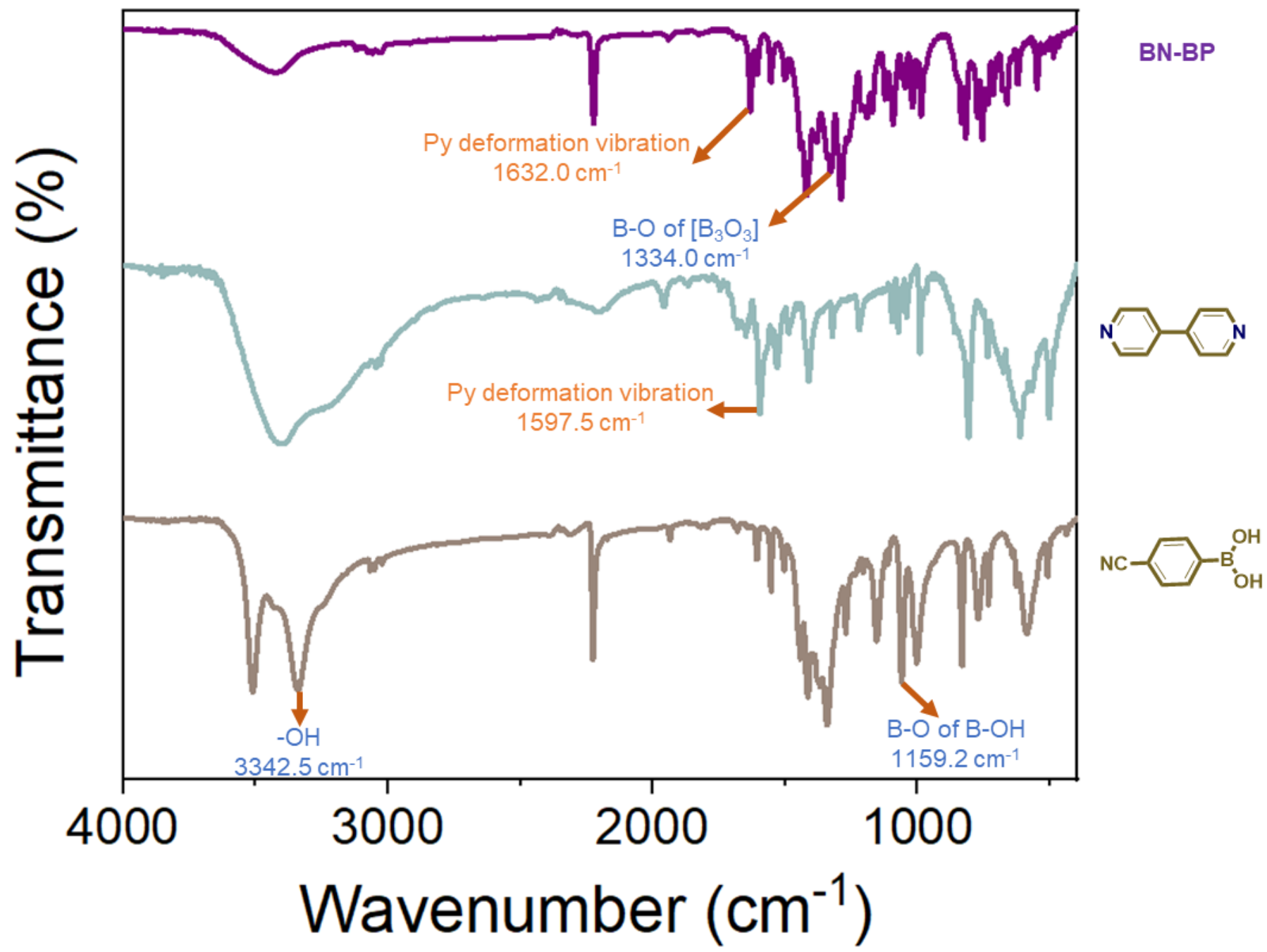

Figure S27. FT-IR spectra of 4-cyanophenylboronic acid, BP, and **BN-BP**, respectively. The complete disappearance of the O–H stretching band from 4-cyanophenylboronic acid at 3342.5 cm^{-1} in the spectrum of **BN-BP** confirms the condensation of boronic acid groups. This is further supported by the B–O stretching vibration, which shifts from 1157.5 cm^{-1} in the monomer to a higher frequency of 1334.0 cm^{-1} in **BN-BP** because of the formation of [B_3O_3] ring. The successful coordination between BP and the boroxine ring is evidenced by the pyridine ring vibration. This band shifts hypsochromically from 1597.5 cm^{-1} in free BP to 1632.0 cm^{-1} in **BN-BP**.

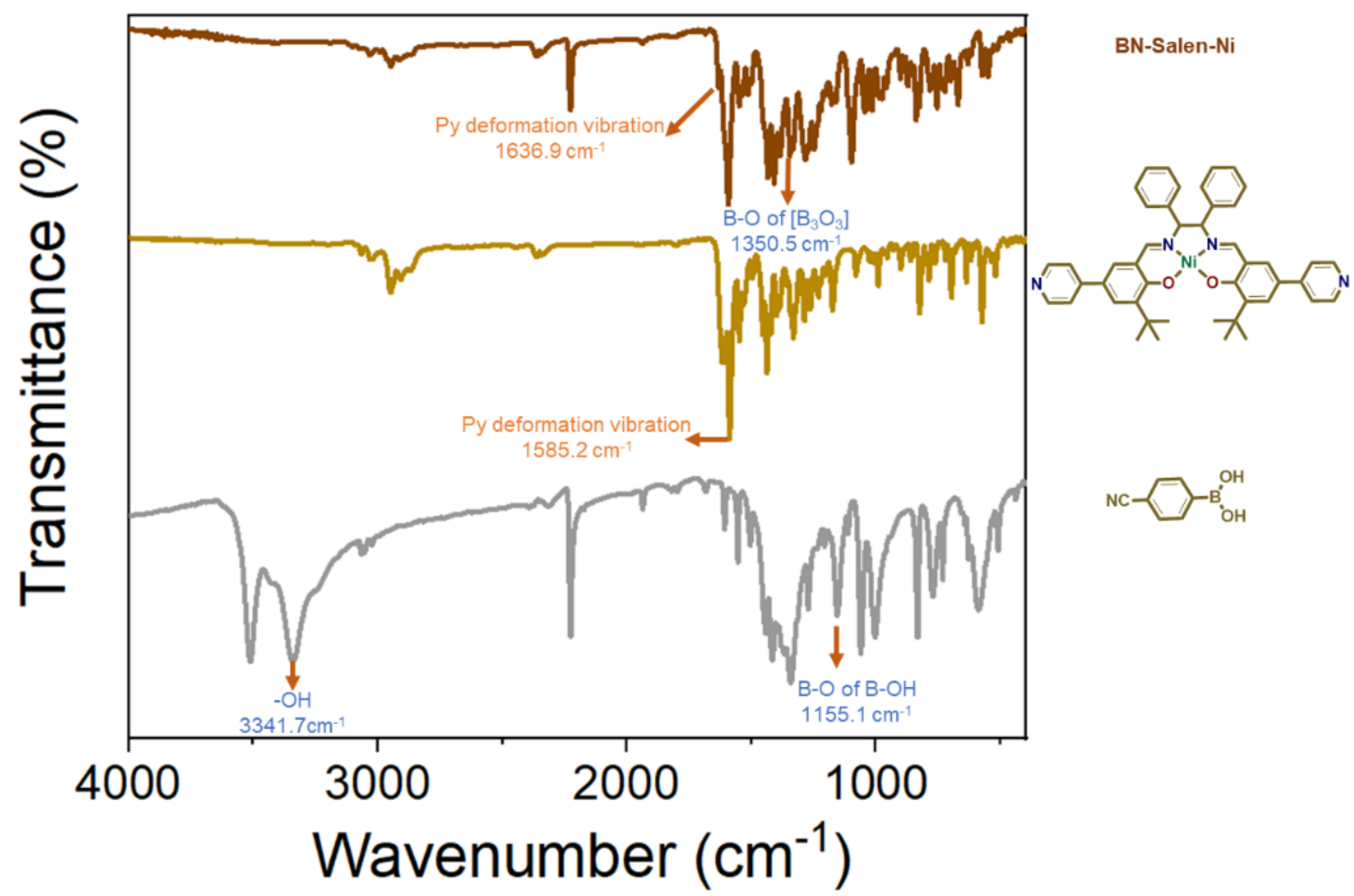

Figure S28. FT-IR spectra of 4-cyanophenylboronic acid, Salen-Ni, and **BN-Salen-Ni**, respectively. The complete disappearance of the O–H stretching band from 4-cyanophenylboronic acid at 3341.7 cm^{-1} in the spectrum of **BN-Salen-Ni** confirms the condensation of boronic acid groups. This is further supported by the B–O stretching vibration, which shifts from 1155.1 cm^{-1} in the monomer to a higher frequency of 1350.5 cm^{-1} in **BN-Salen-Ni** because of the formation of [B_3O_3] ring. The successful coordination between Salen-Ni and the boroxine ring is evidenced by the pyridine ring vibration. This band shifts hypsochromically from 1585.2 cm^{-1} in free Salen-Ni to 1636.9 cm^{-1} in **BN-Salen-Ni**.

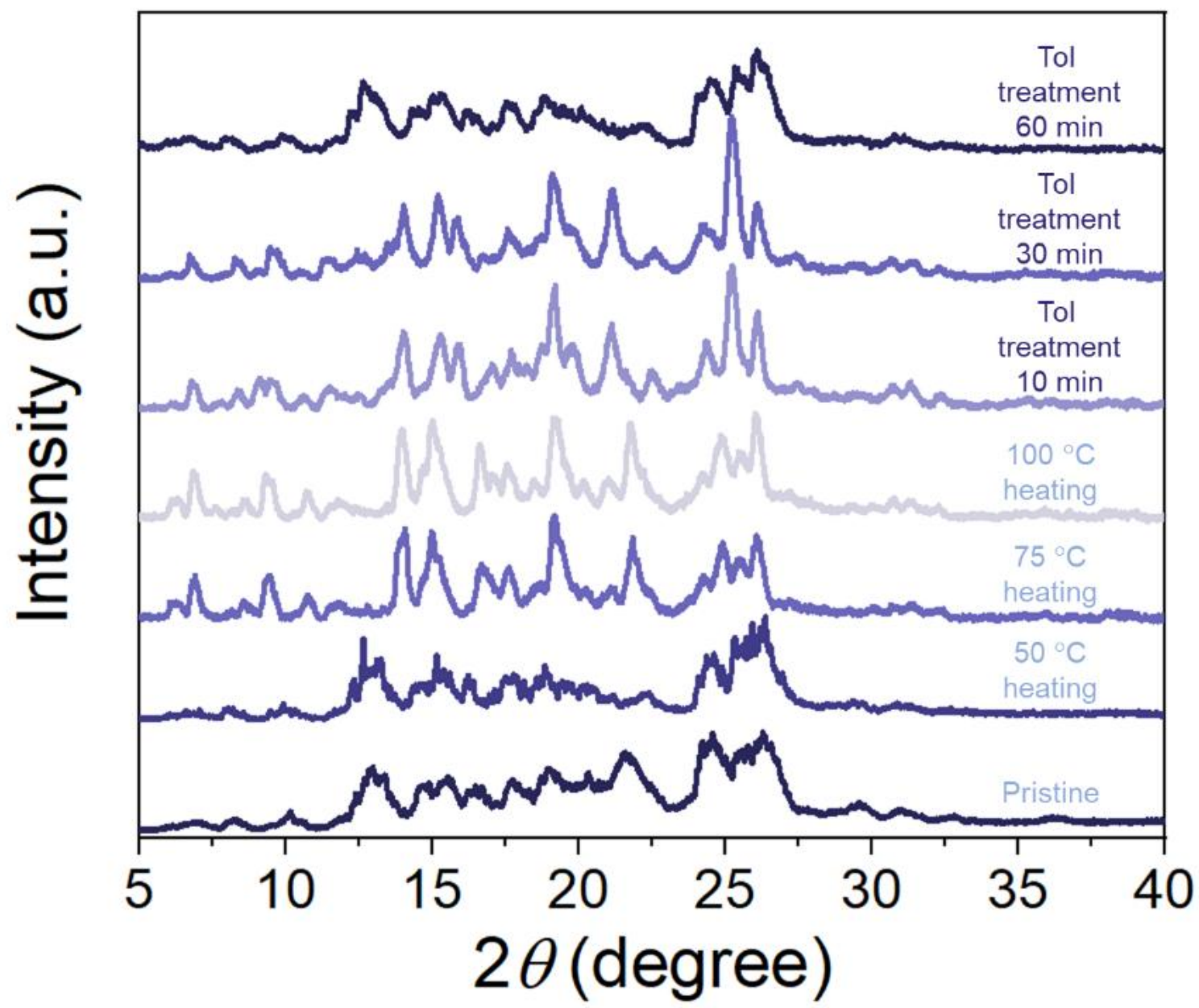

Figure S29. PXRD patterns of **BN-DPTA1** heated at various temperatures for 1 h and then soaked in Tol at 100 °C for different time.

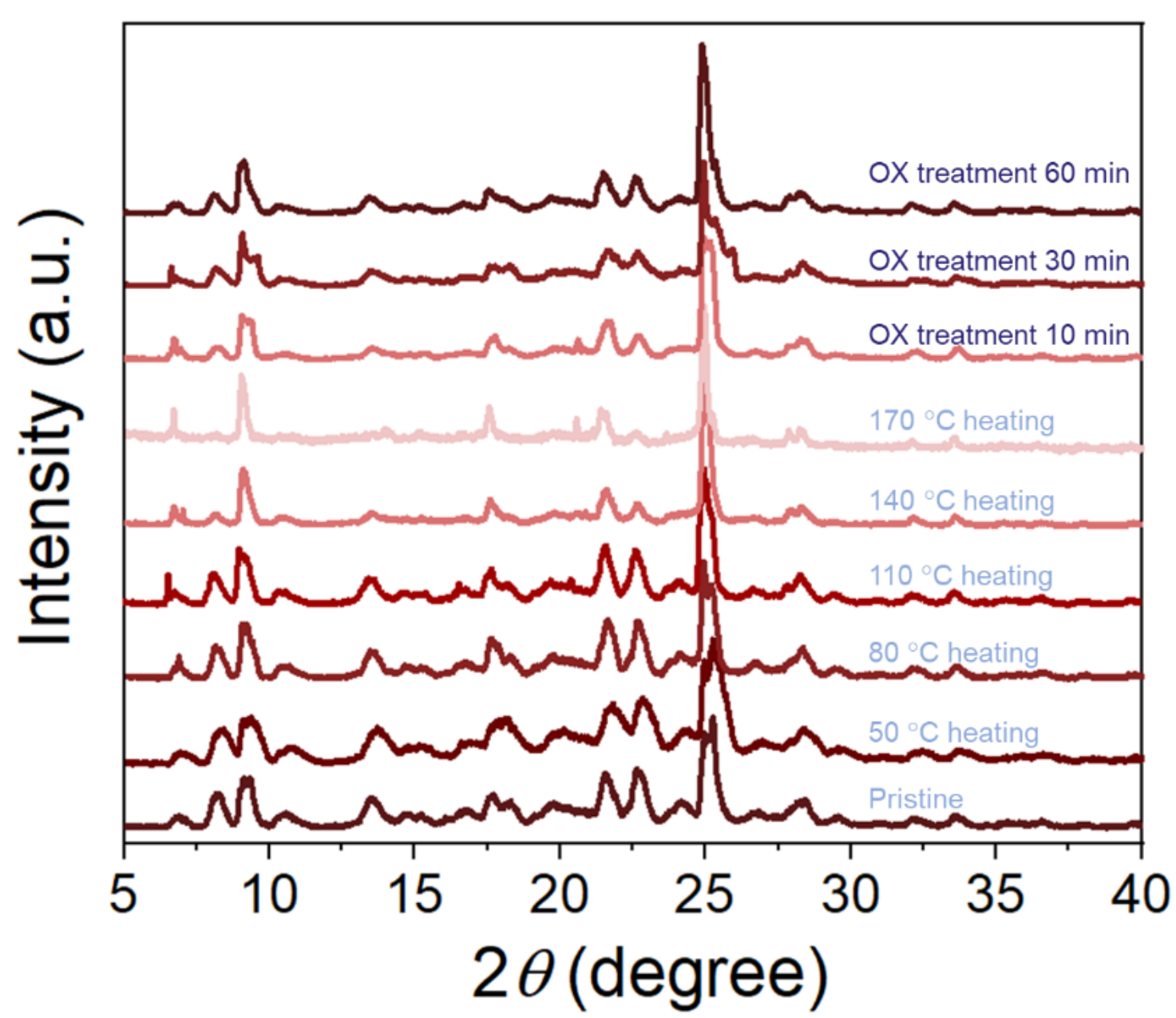

Figure S30. PXRD patterns of **BN-DPTA2** heated at various temperatures for 1 h and then

soaked in OX at 100 °C for different time.

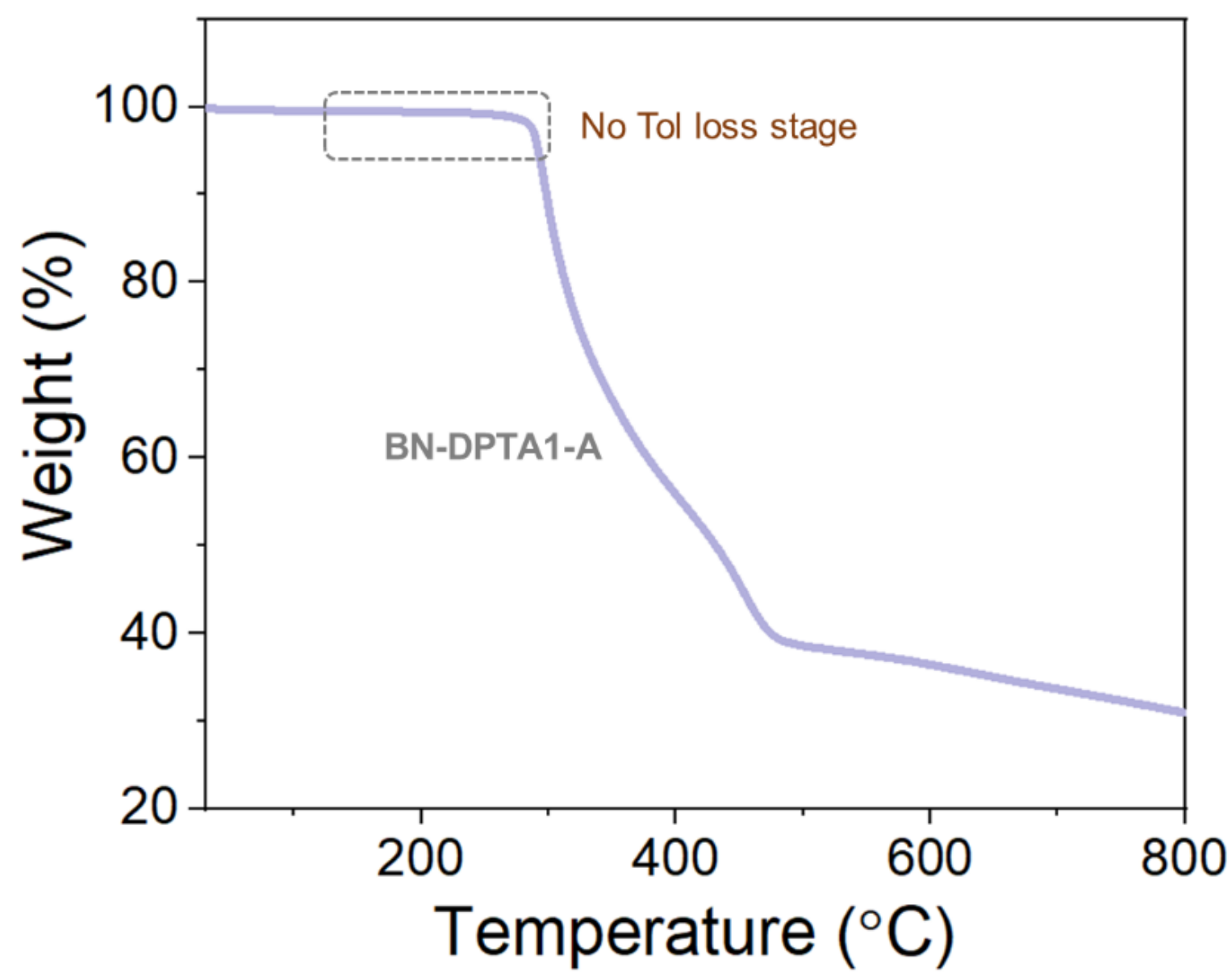

Figure S31. TGA curve of **BN-DPTA1-A**.

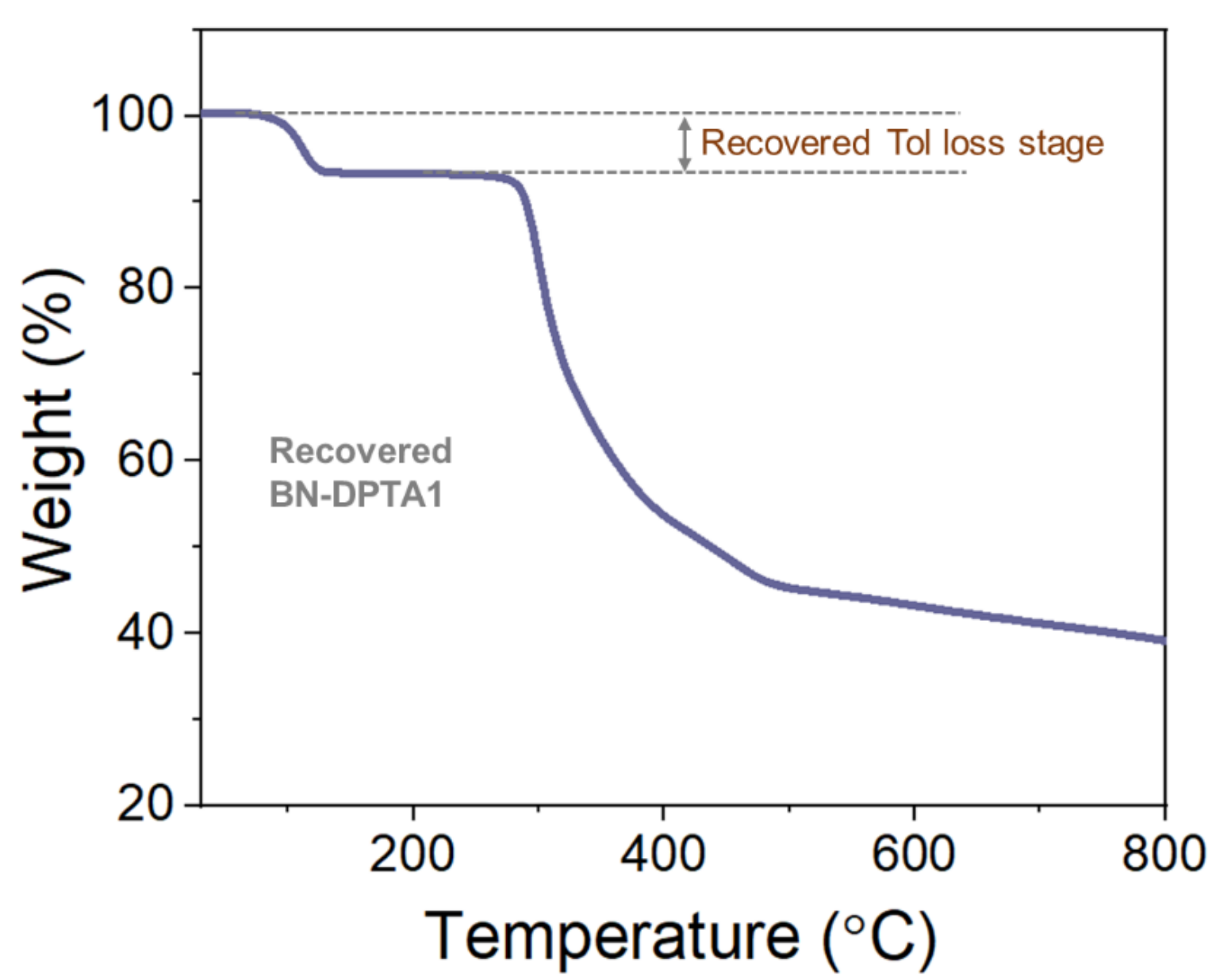

Figure S32. TGA curve of regenerated **BN-DPTA1** by soaking **BN-DPTA1-A** in Tol at

100 °C for 1 hour.

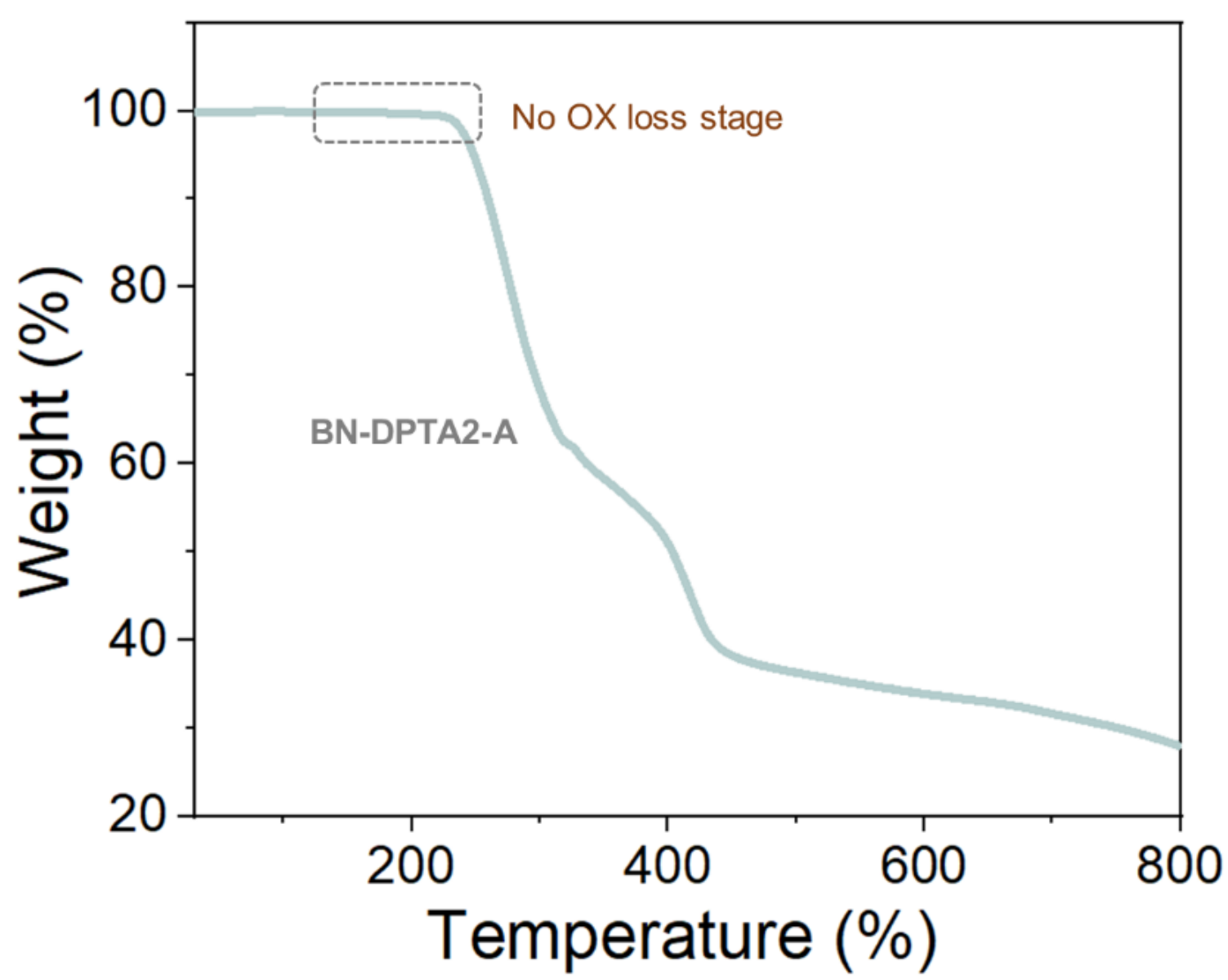

Figure S33. TGA curve of **BN-DPTA2-A**.

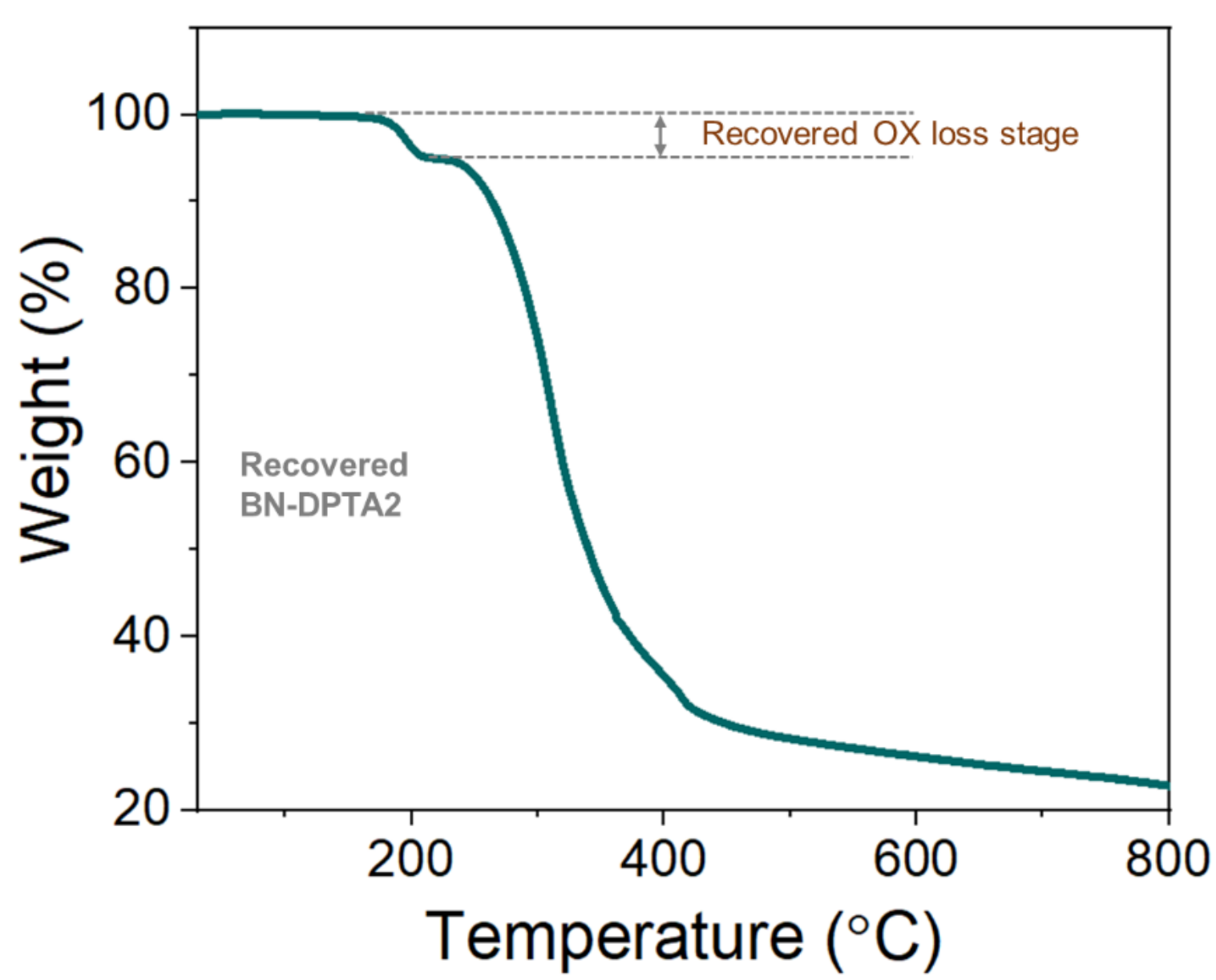

Figure S34. TGA curve of regenerated **BN-DPTA2** by soaking **BN-DPTA2-A** in OX at

100 °C for 1 hour.

(a)

(b)

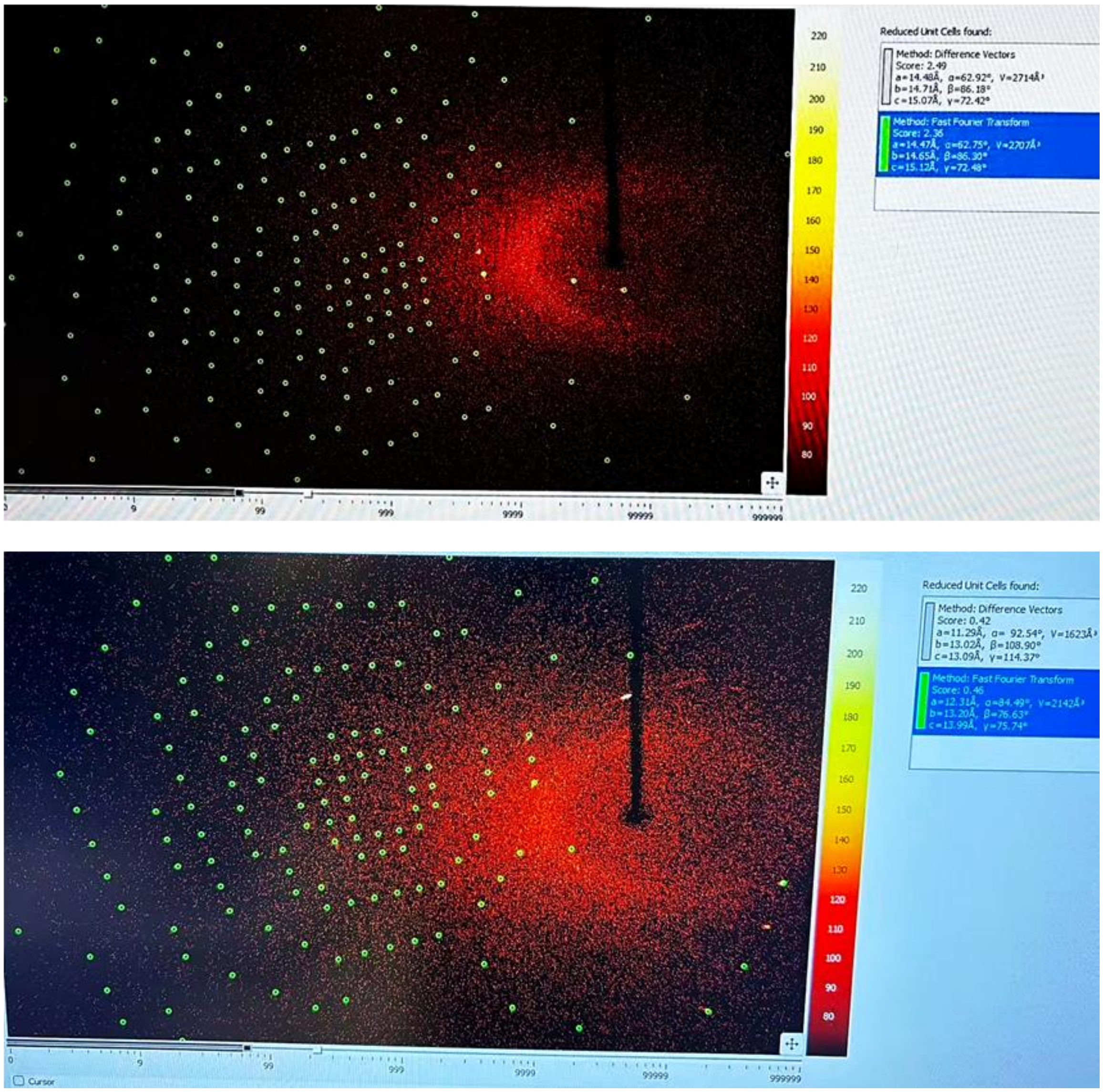

Figure S35. The unit cell parameters of guest removed state (a) **BN-DPTA1-A** and (b) **BN-DPTA2-A**.

Table S5. Comparison of unit cell parameters between **BN-DPTA1**/**BN-DPTA2** and **BN-DPTA1-A**/**BN-DPTA2-A**.

Identification code	**BN-DPTA1**	**BN-DPTA1-A**	**BN-DPTA2**	**BN-DPTA2-A**
Temperature (K)	100	100	100	100
Cryst. Syst.	triclinic	triclinic	triclinic	triclinic
a (Å)	14.86	14.47	13.27	12.31
b (Å)	14.94	14.65	13.64	13.20
c (Å)	15.43	15.12	17.73	13.99
α, β, γ (°)	64.0, 74.4, 72.1	62.8, 86.3, 72.5	107.5, 93.3, 104.4	84.5, 76.6, 75.7
Volume (Å^3)	2897	2707	2935	2142

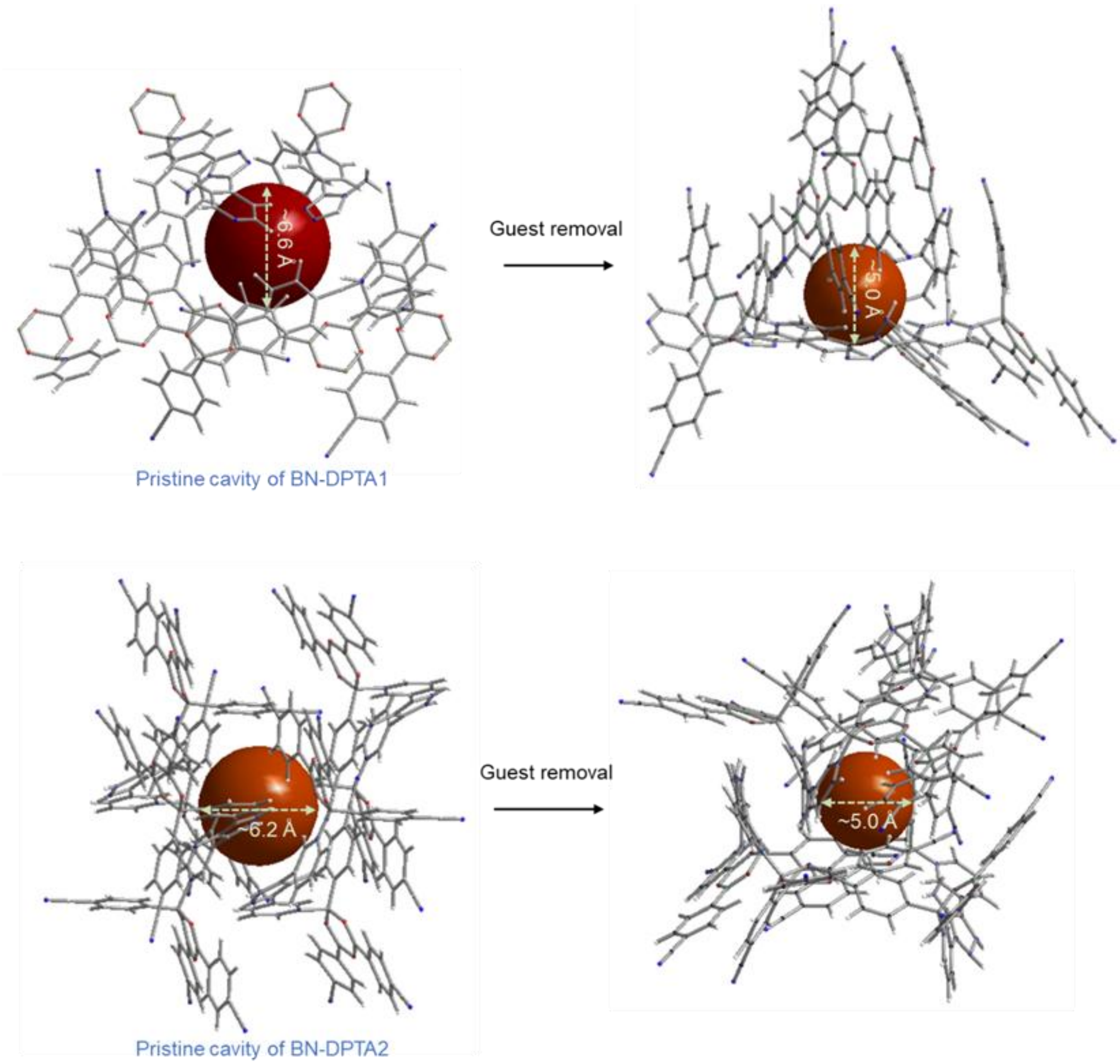

Figure S36. The cavity structures of pristine **BN-DPTA1** and **BN-DPTA2**, along with the simulated cavities after guest removal.

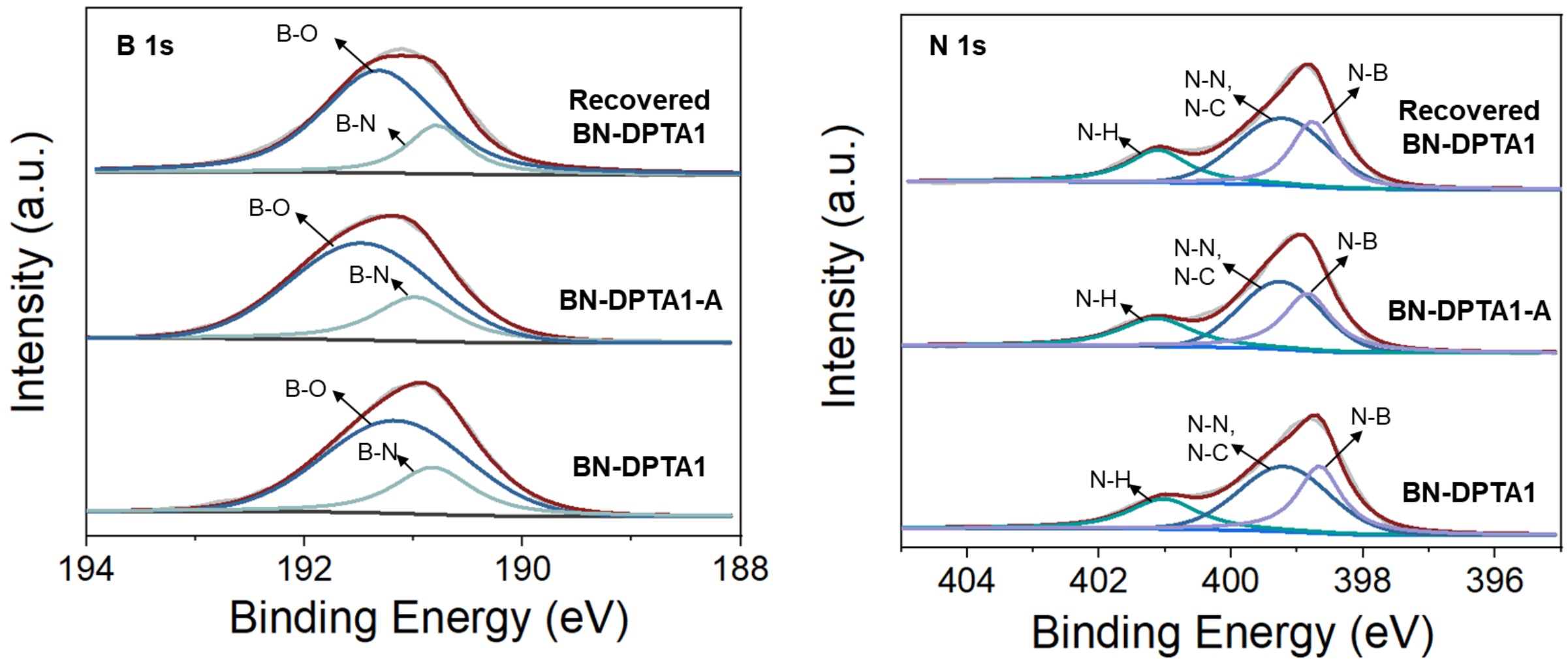

Figure S37. B 1s and N 1s XPS spectra of **BN-DPTA1**, **BN-DPTA1-A**, and recovered **BN-DPTA1**.

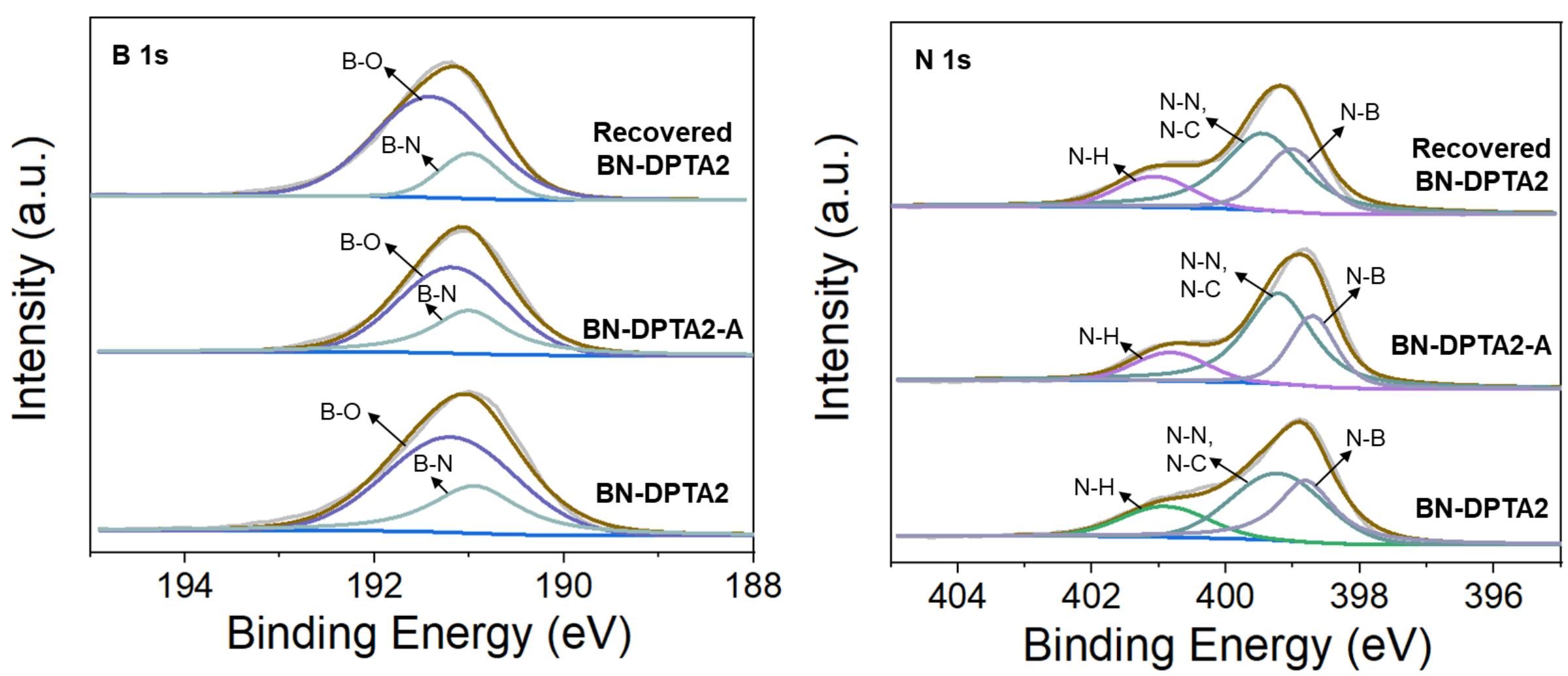

Figure S38. B 1s and N 1s XPS spectra of **BN-DPTA2**, **BN-DPTA2-A**, and recovered **BN-DPTA2**.

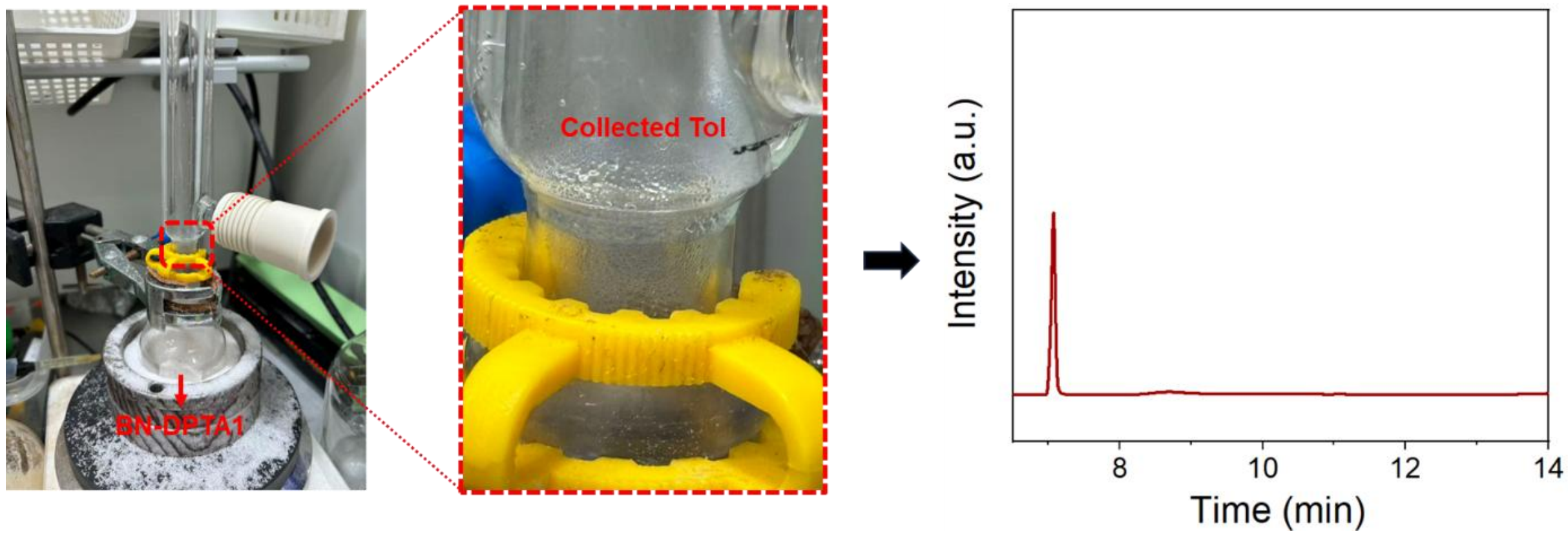

Figure S39. Digital photos showing the collection of Tol from **BN-DPTA1** via evaporation, and corresponding GC-FID analysis of the collected Tol.

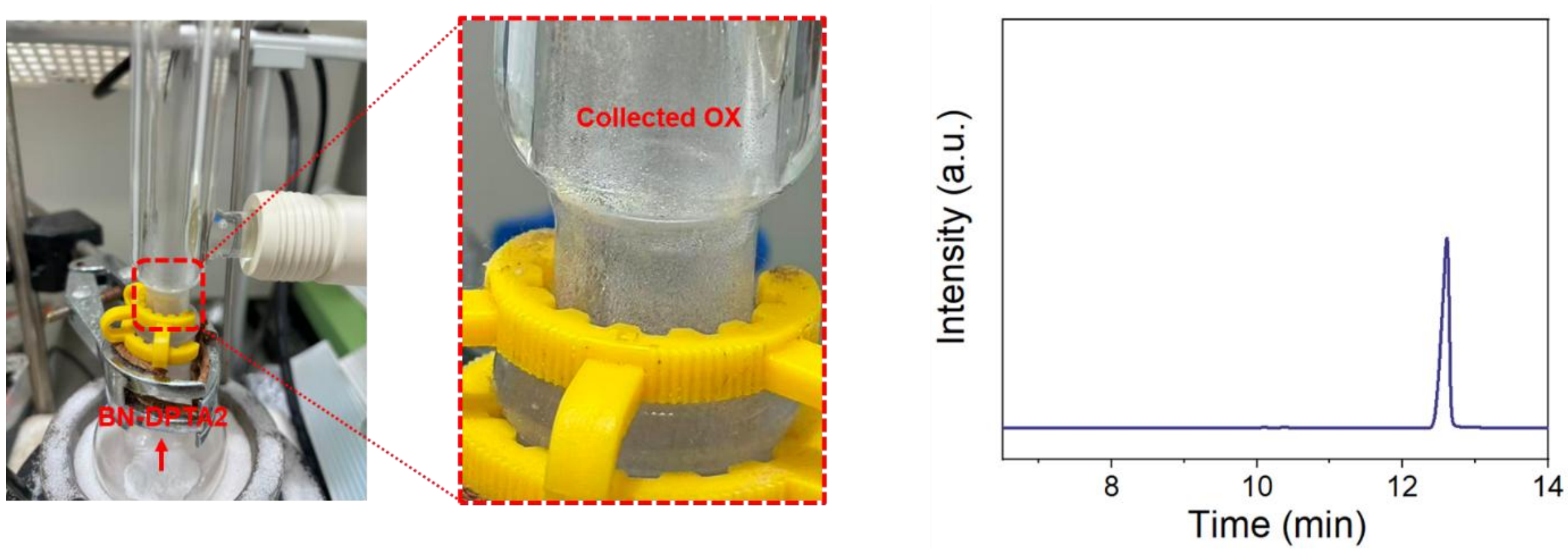

Figure S40. Digital photos showing the collection of OX from **BN-DPTA2** via evaporation, and corresponding GC-FID analysis of the collected OX.

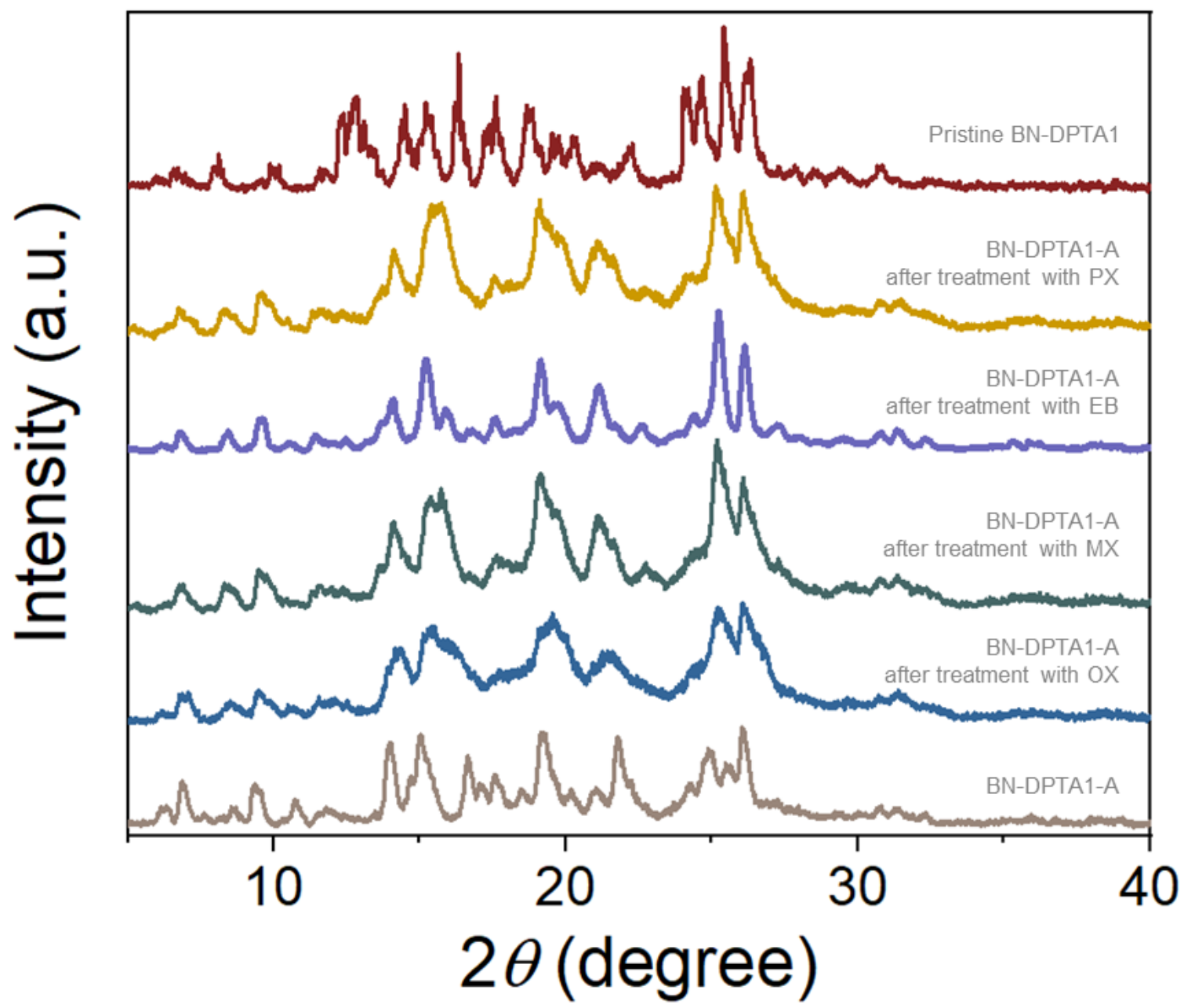

Figure S41. PXRD patterns of **BN-DPTA1-A** after soaking in OX, MX, EB and PX at 100 °C for 1 hour.

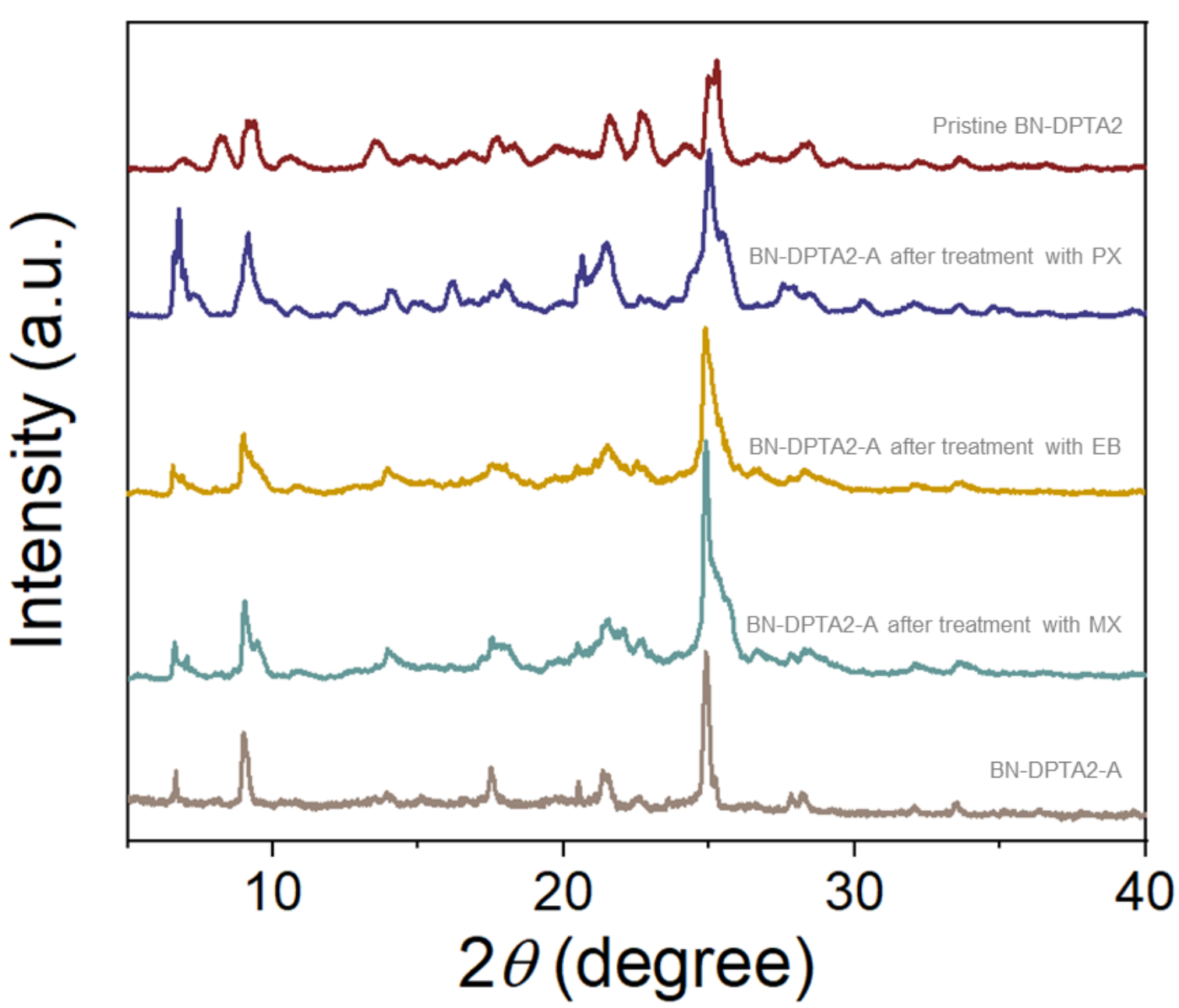

Figure S42. PXRD patterns of **BN-DPTA2-A** after soaking in MX, EB and PX at 100 °C

for 1 hour.

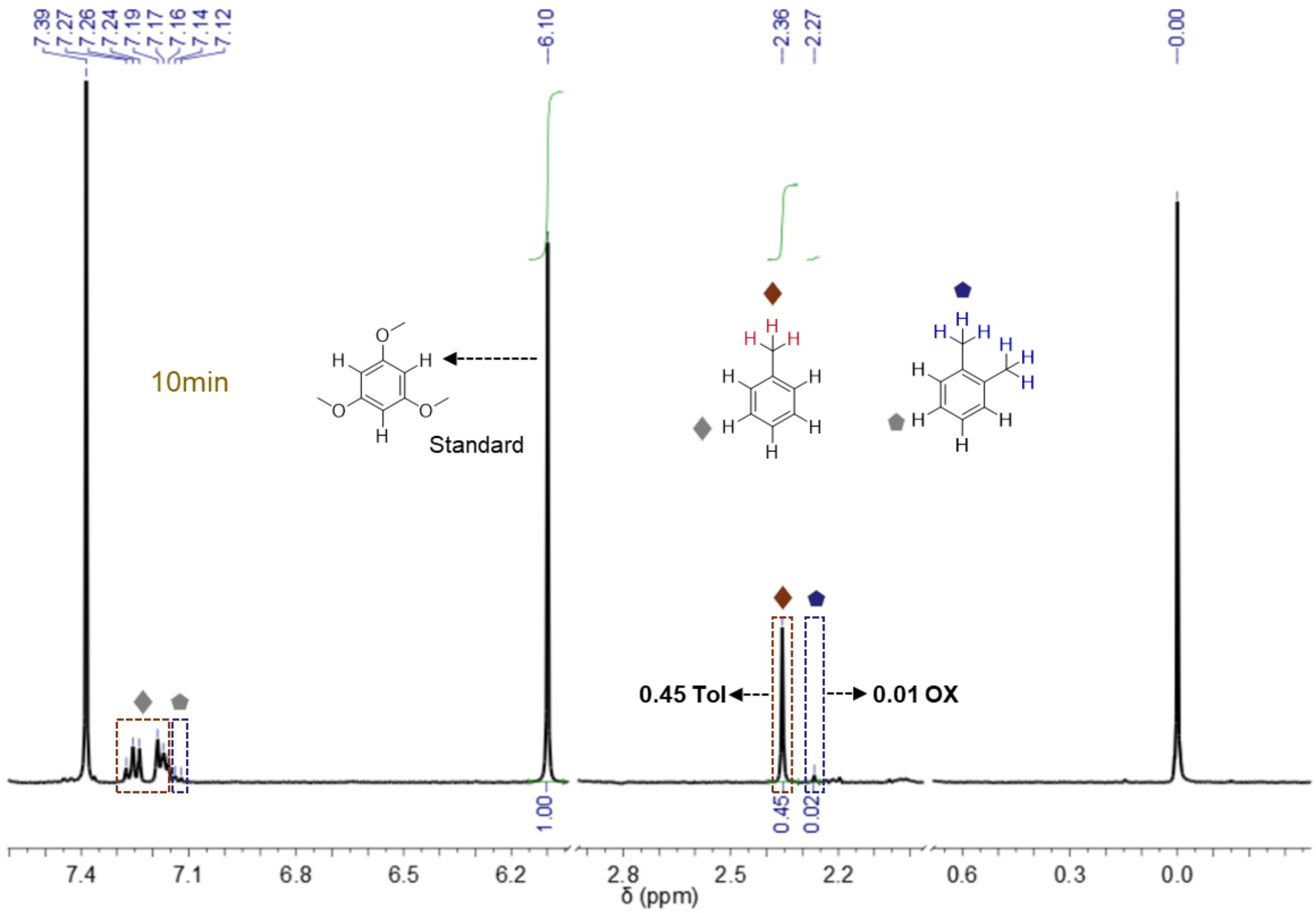

Figure S43. Relative uptake of Tol and OX by **BN-DPTA1-A** after exposure to a 1:1 (mol/mol) binary mixture for 10 minutes, as determined by ^{1}H NMR spectrum (298K, $CDCl_3$, 400 MHz).

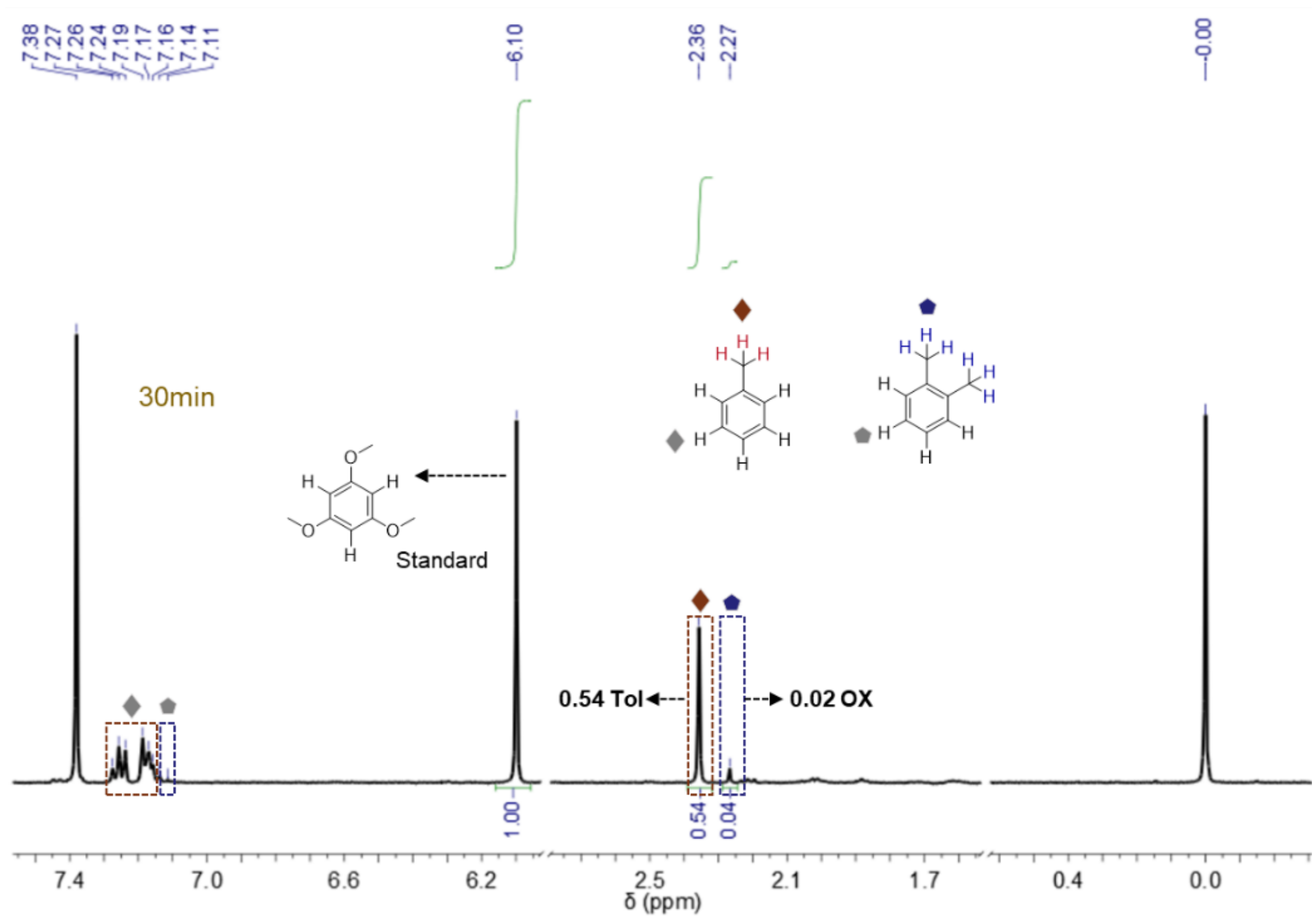

Figure S44. Relative uptake of Tol and OX by **BN-DPTA1-A** after exposure to a 1:1 (mol/mol) binary mixture for 30 minutes, as determined by ^{1}H NMR spectrum (298K, $CDCl_3$, 400 MHz).

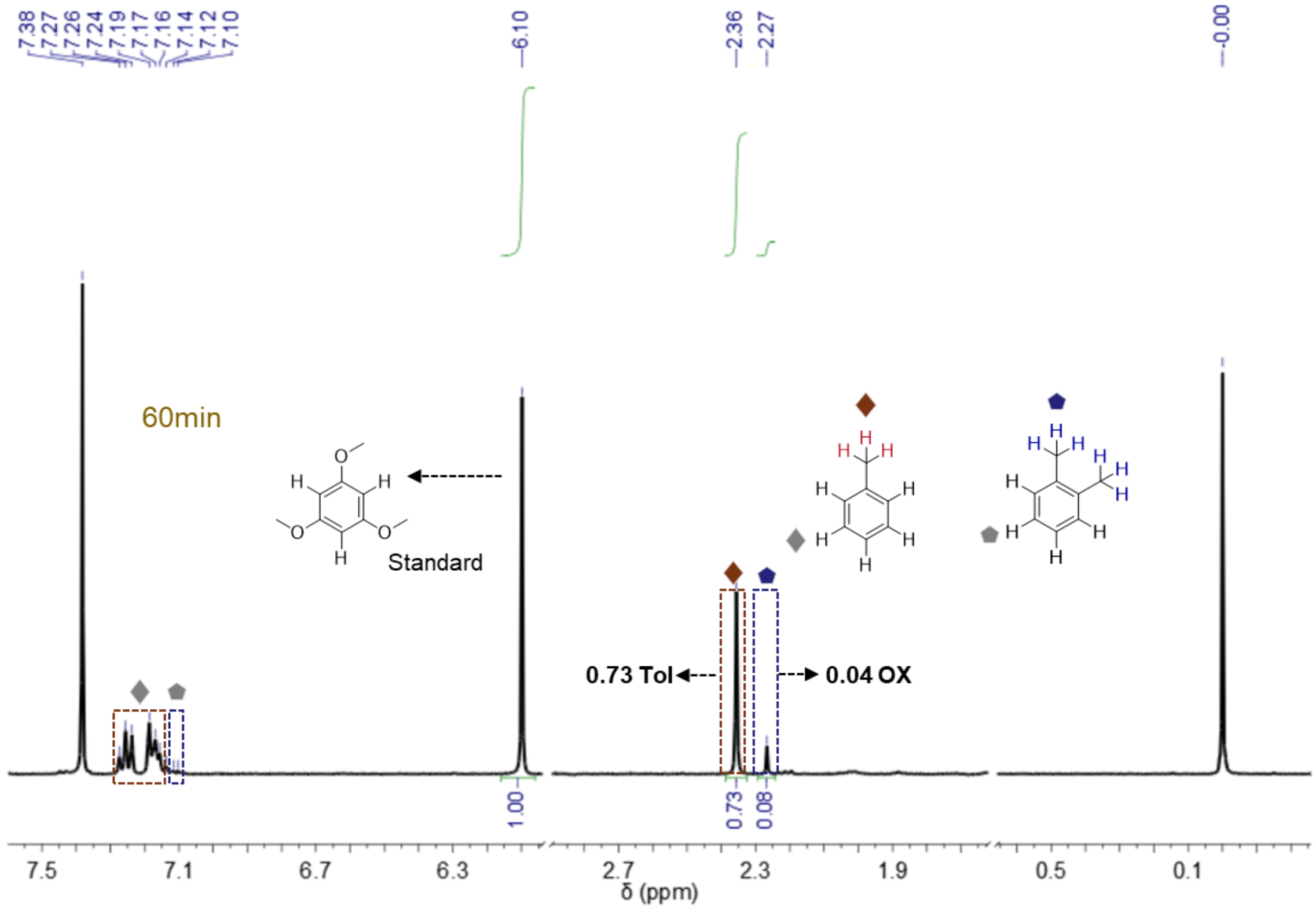

Figure S45. Relative uptake of Tol and OX by **BN-DPTA1-A** after exposure to a 1:1 (mol/mol) binary mixture for 60 minutes, as determined by ^{1}H NMR spectrum (298K, $CDCl_3$, 400 MHz).

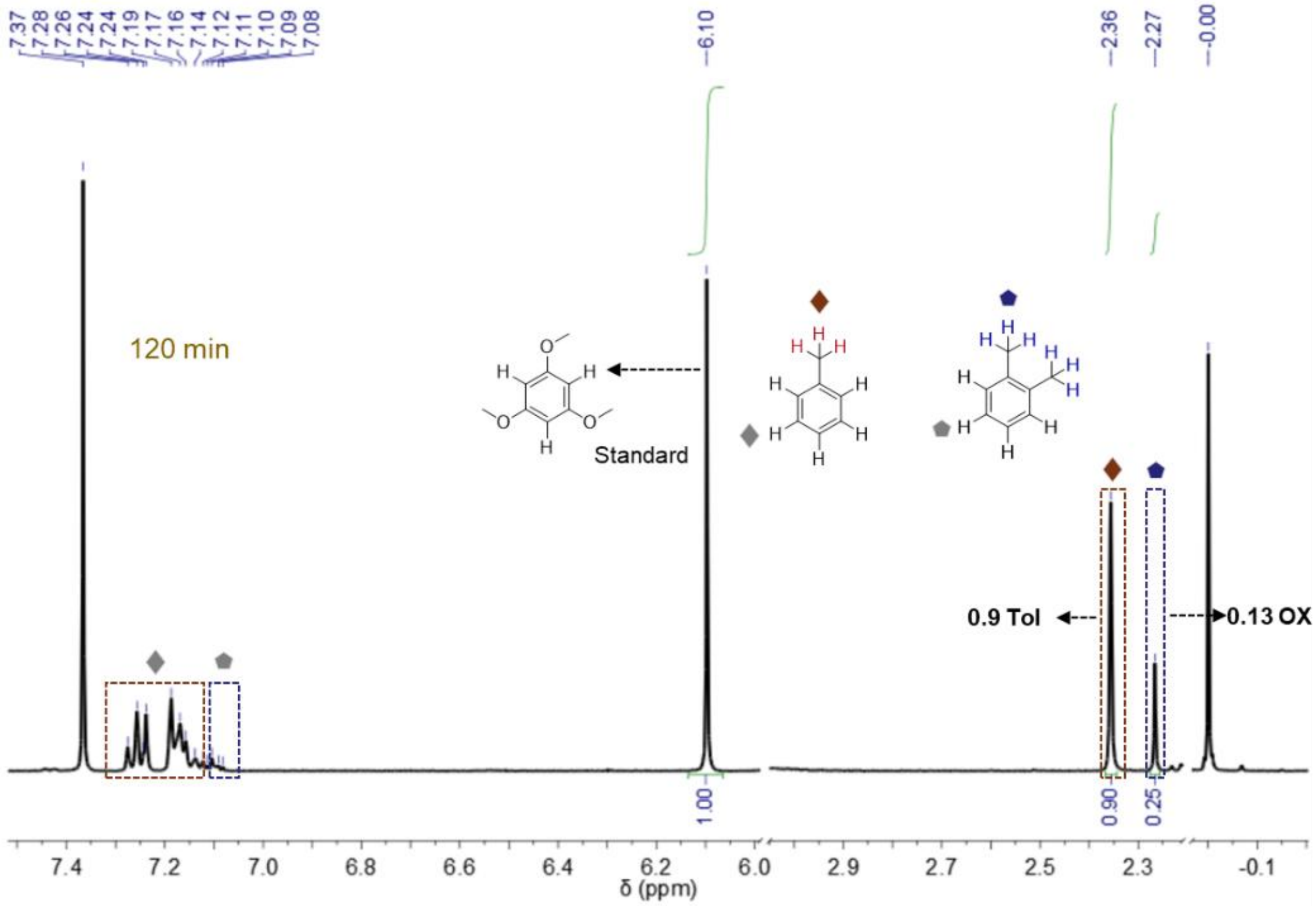

Figure S46. Relative uptake of Tol and OX by **BN-DPTA1-A** after exposure to a 1:1 (mol/mol) binary mixture for 120 minutes, as determined by ^{1}H NMR spectrum (298K, $CDCl_3$, 400 MHz).

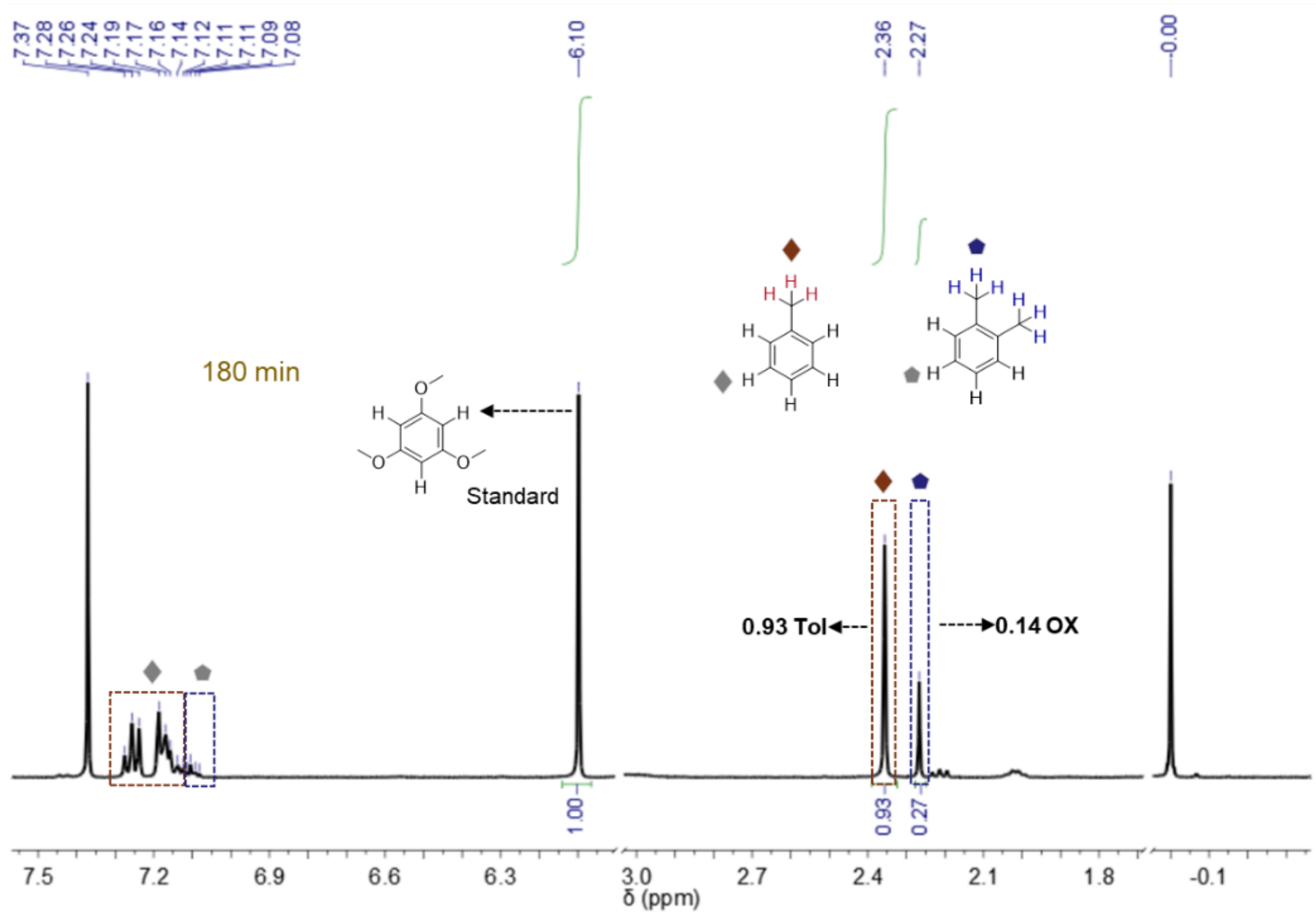

Figure S47. Relative uptake of Tol and OX by **BN-DPTA1-A** after exposure to a 1:1 (mol/mol) binary mixture for 180 minutes, as determined by ^{1}H NMR spectrum (298K, $CDCl_3$, 400 MHz).

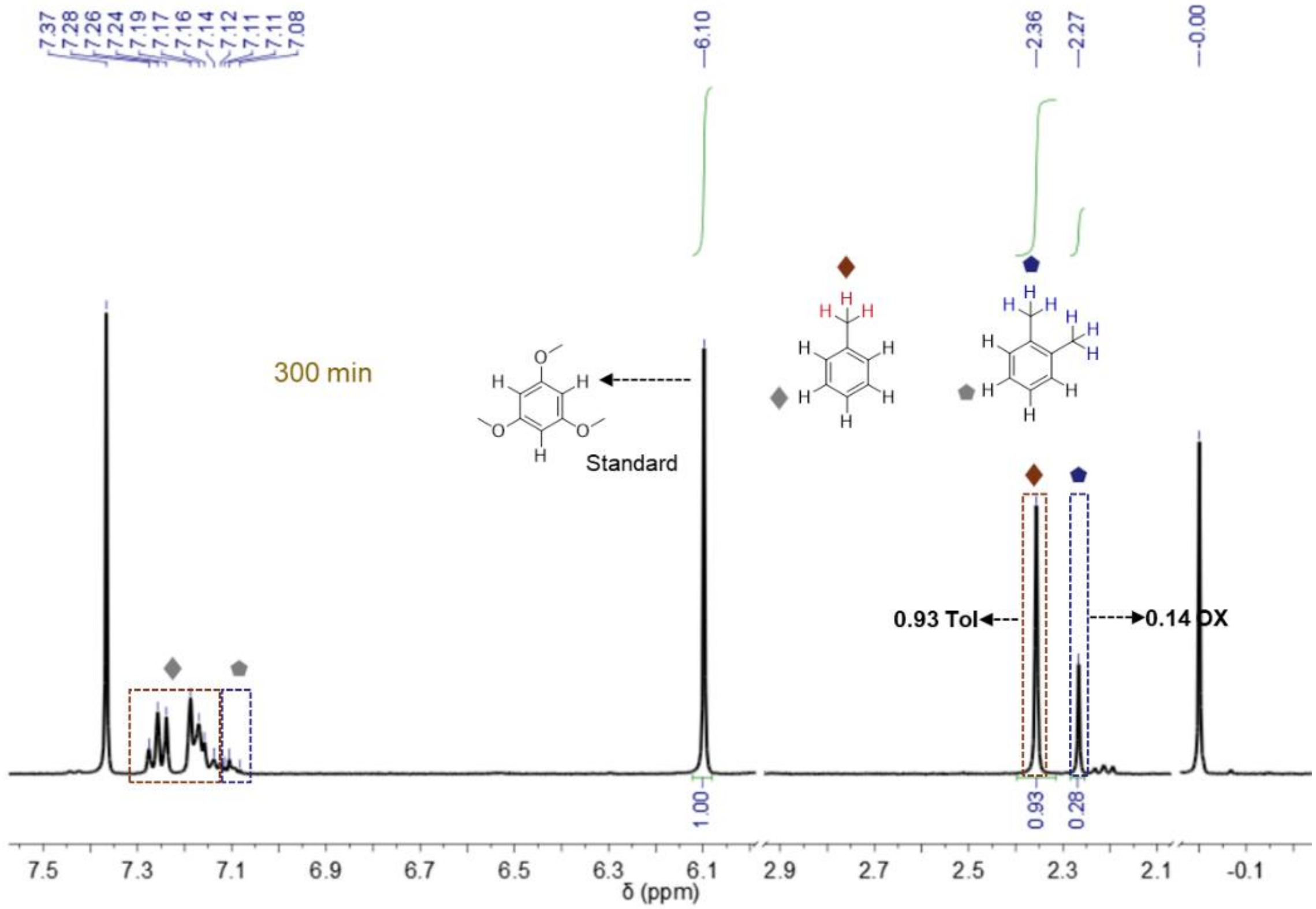

Figure S48. Relative uptake of Tol and OX by **BN-DPTA1-A** after exposure to a 1:1 (mol/mol) binary mixture for 300 minutes, as determined by ^{1}H NMR spectrum (298K, CDCl3, 400 MHz).

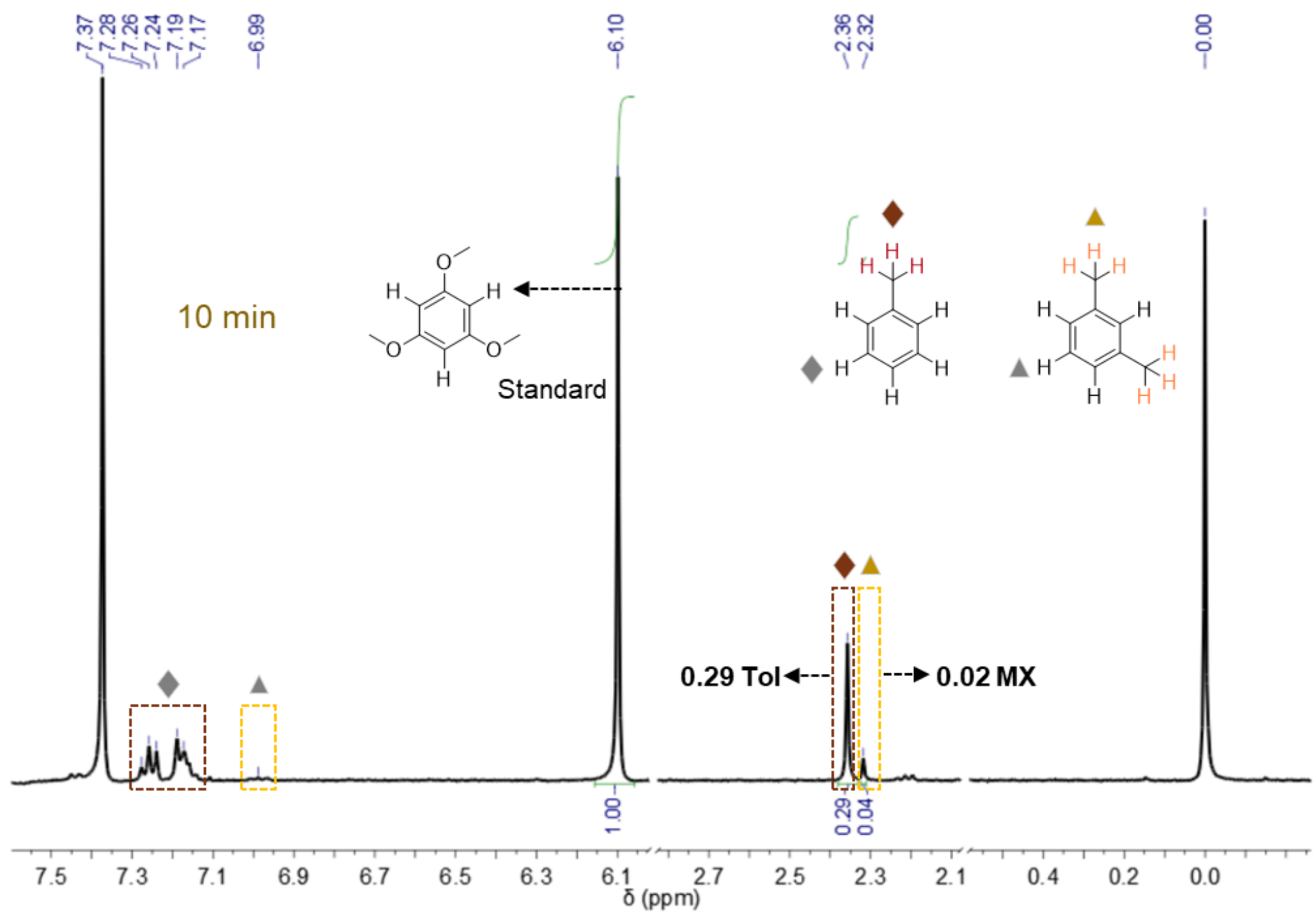

Figure S49. Relative uptake of Tol and MX by **BN-DPTA1-A** after exposure to a 1:1 (mol/mol) binary mixture for 10 minutes, as determined by ^{1}H NMR spectrum (298K, $CDCl_3$, 400 MHz).

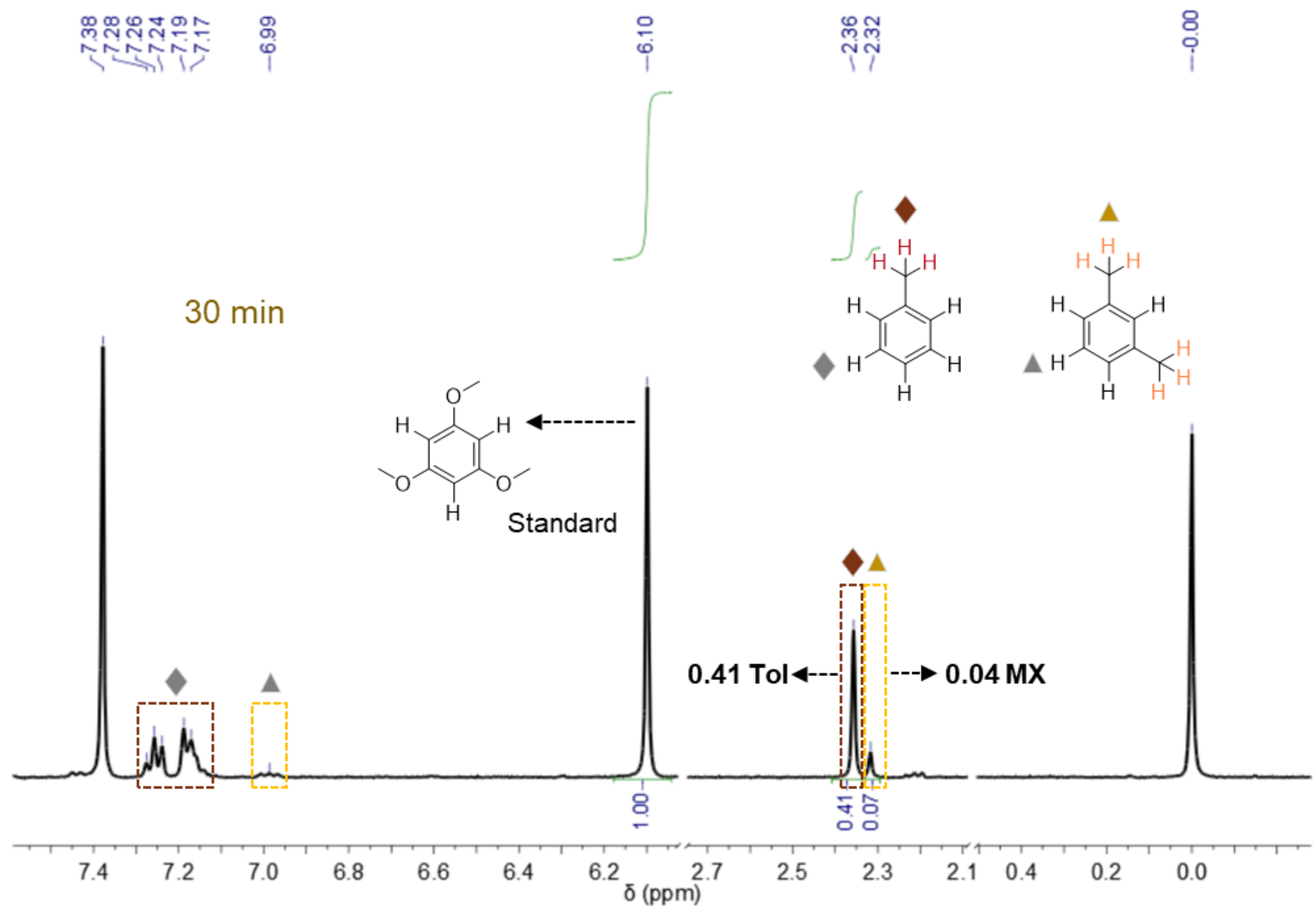

Figure S50. Relative uptake of Tol and MX by **BN-DPTA1-A** after exposure to a 1:1 (mol/mol) binary mixture for 30 minutes, as determined by ^{1}H NMR spectrum (298K, $CDCl_3$, 400 MHz).

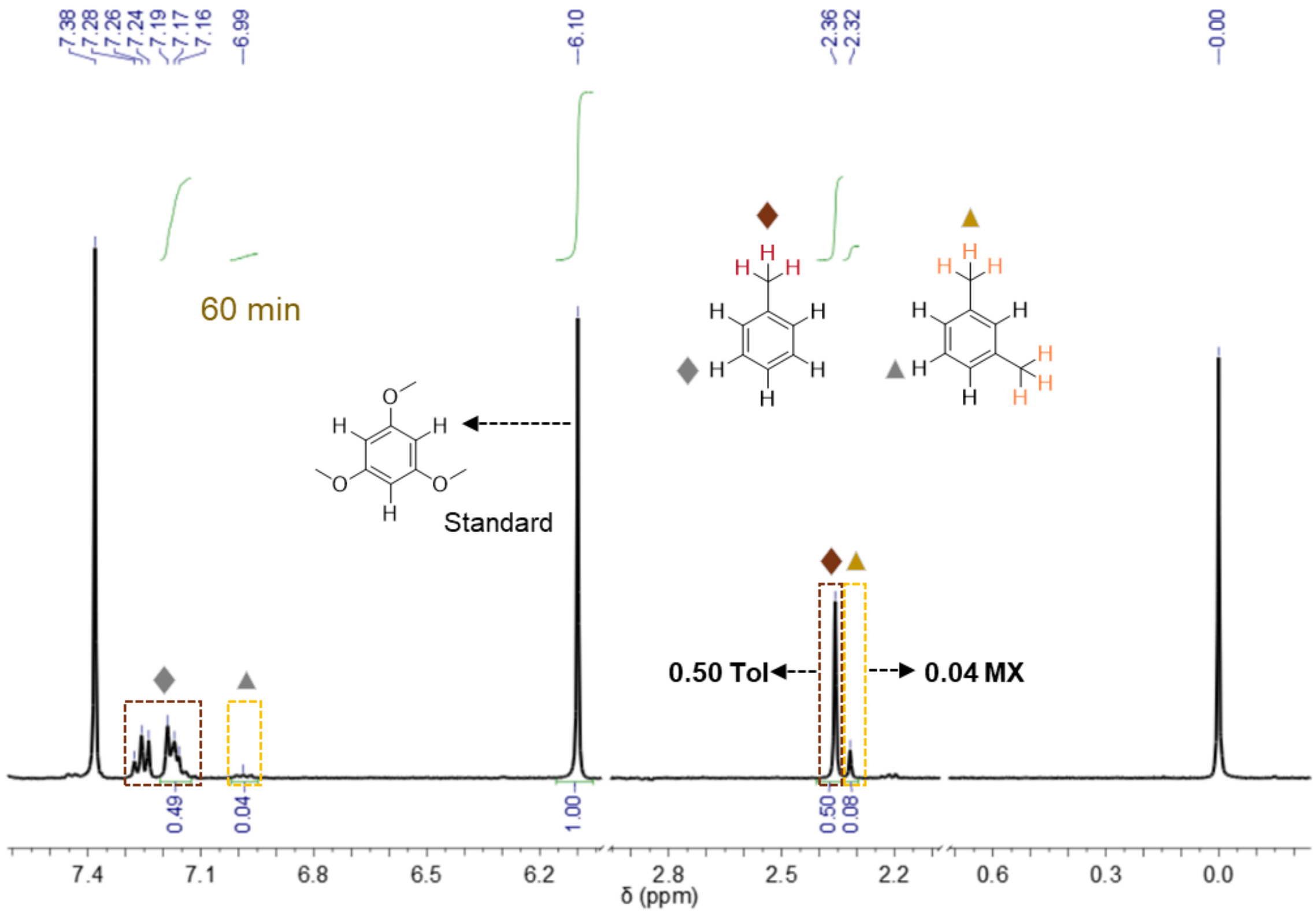

Figure S51. Relative uptake of Tol and MX by **BN-DPTA1-A** after exposure to a 1:1 (mol/mol) binary mixture for 60 minutes, as determined by ^{1}H NMR spectrum (298K, $CDCl_3$, 400 MHz).

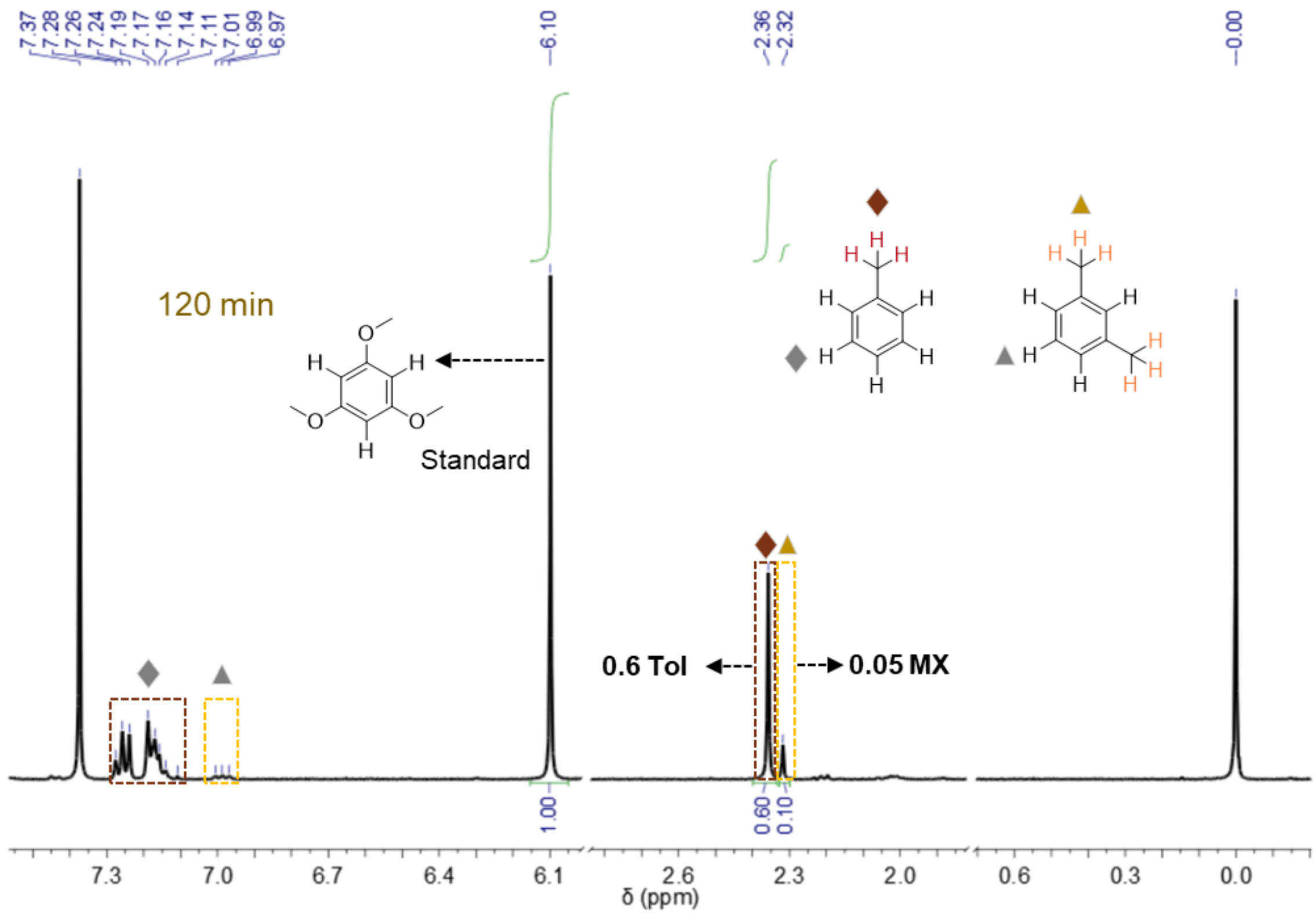

Figure S52. Relative uptake of Tol and MX by **BN-DPTA1-A** after exposure to a 1:1 (mol/mol) binary mixture for 120 minutes, as determined by ^{1}H NMR spectrum (298K, $CDCl_3$, 400 MHz).

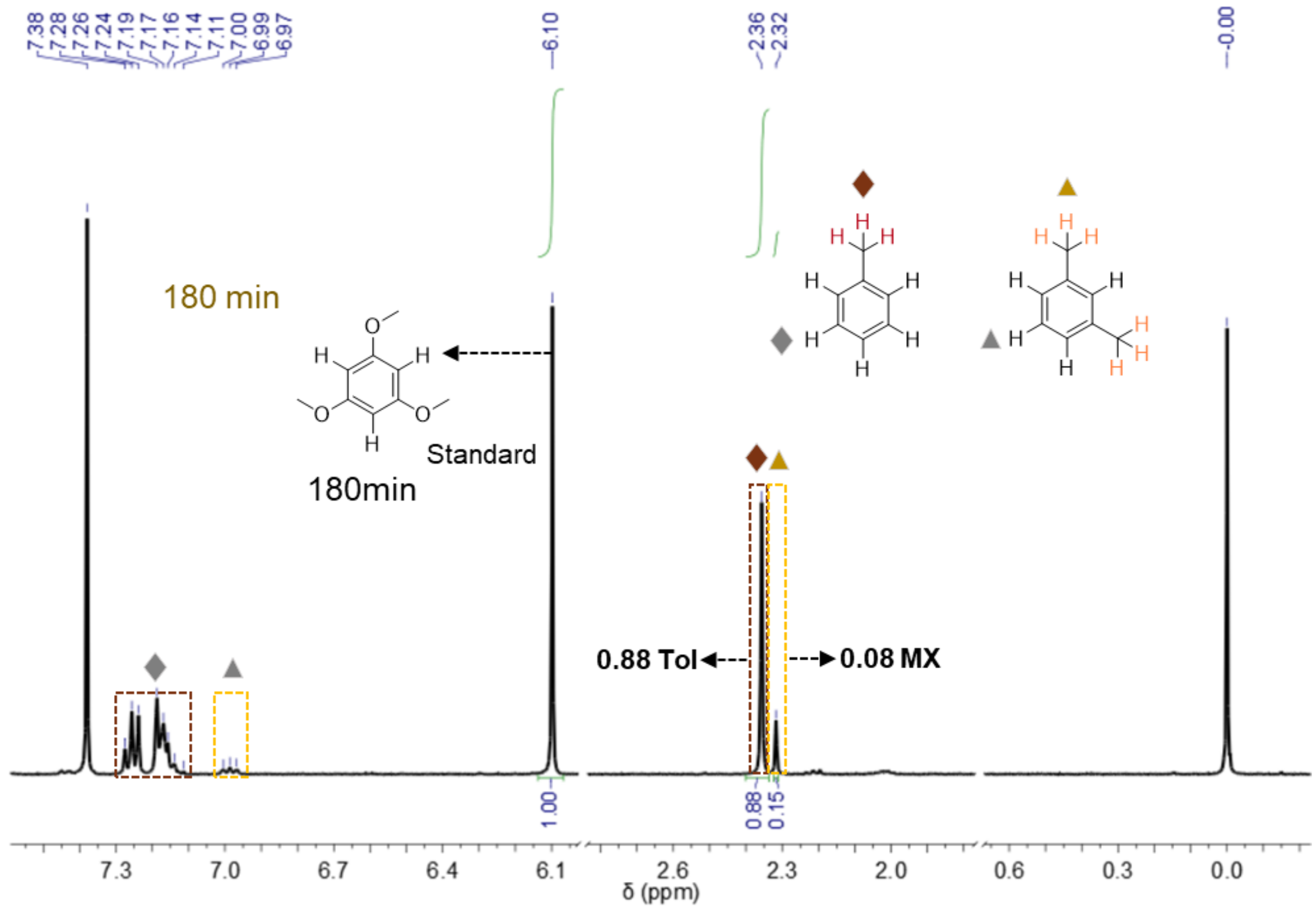

Figure S53. Relative uptake of Tol and MX by **BN-DPTA1-A** after exposure to a 1:1 (mol/mol) binary mixture for 180 minutes, as determined by ^{1}H NMR spectrum (298K, $CDCl_3$, 400 MHz).

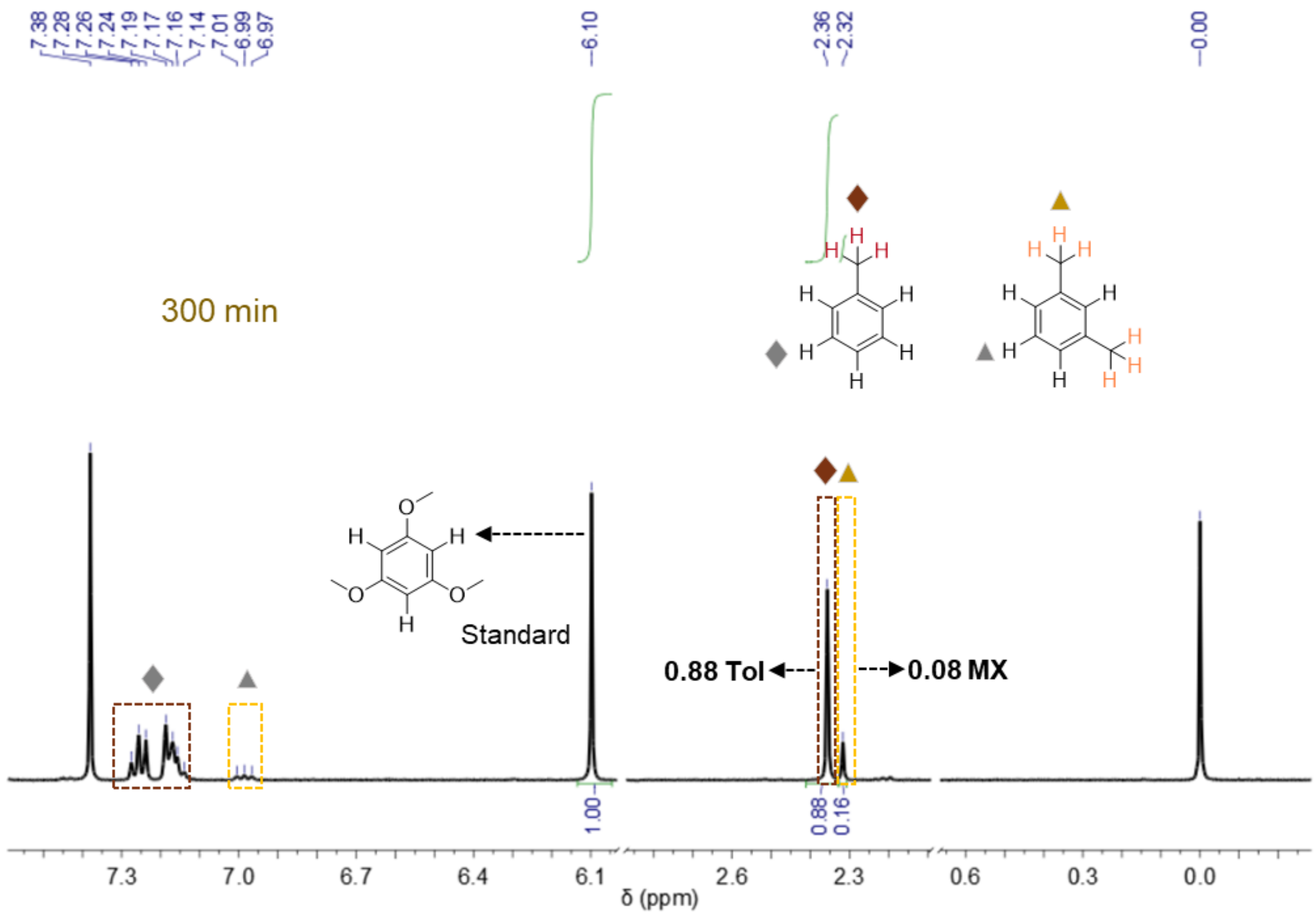

Figure S54. Relative uptake of Tol and MX by **BN-DPTA1-A** after exposure to a 1:1 (mol/mol) binary mixture for 300 minutes, as determined by ^{1}H NMR spectrum (298K, $CDCl_3$, 400 MHz).

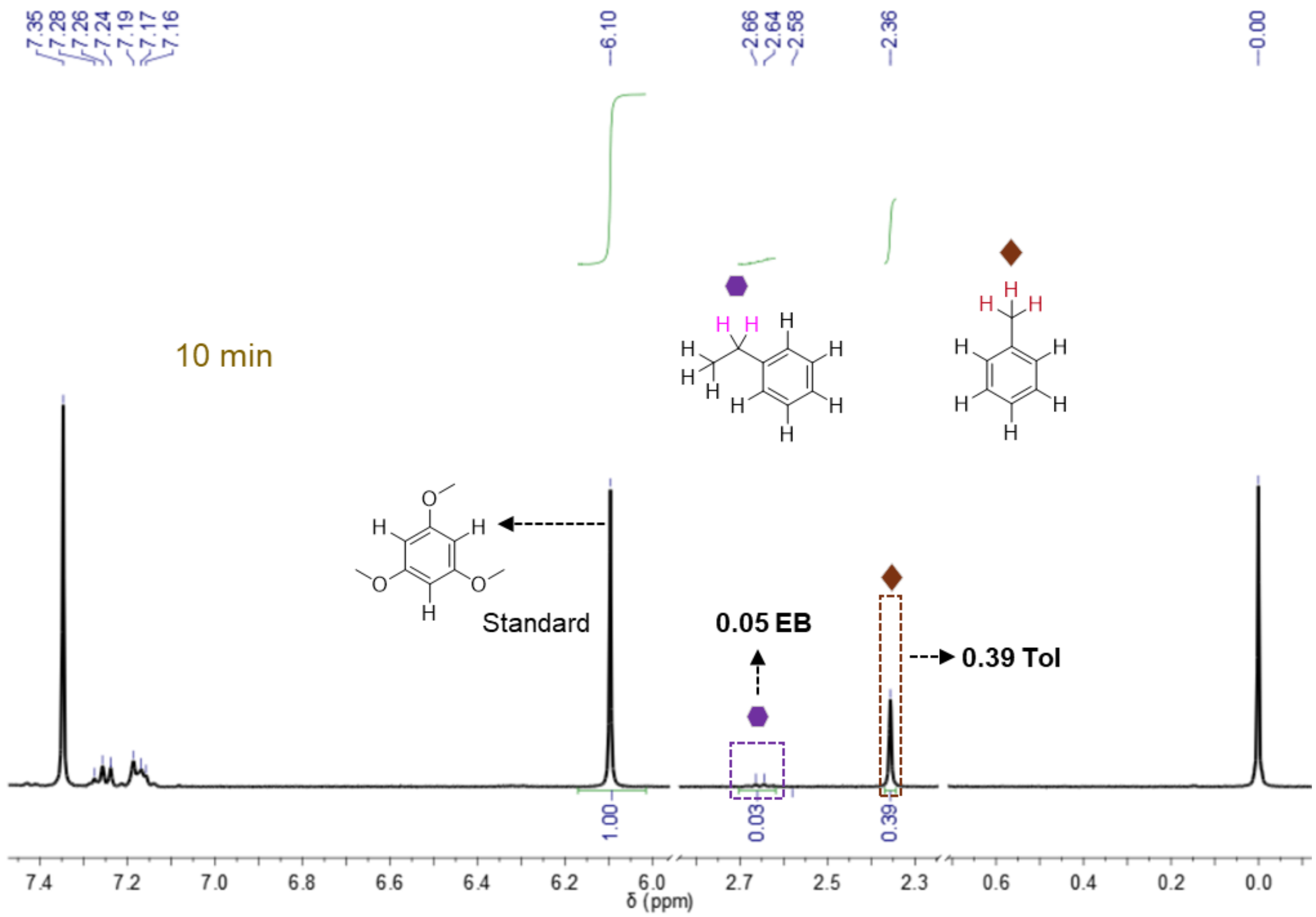

Figure S55. Relative uptake of Tol and EB by **BN-DPTA1-A** after exposure to a 1:1 (mol/mol) binary mixture for 10 minutes, as determined by ^{1}H NMR spectrum (298K, $CDCl_3$, 400 MHz).

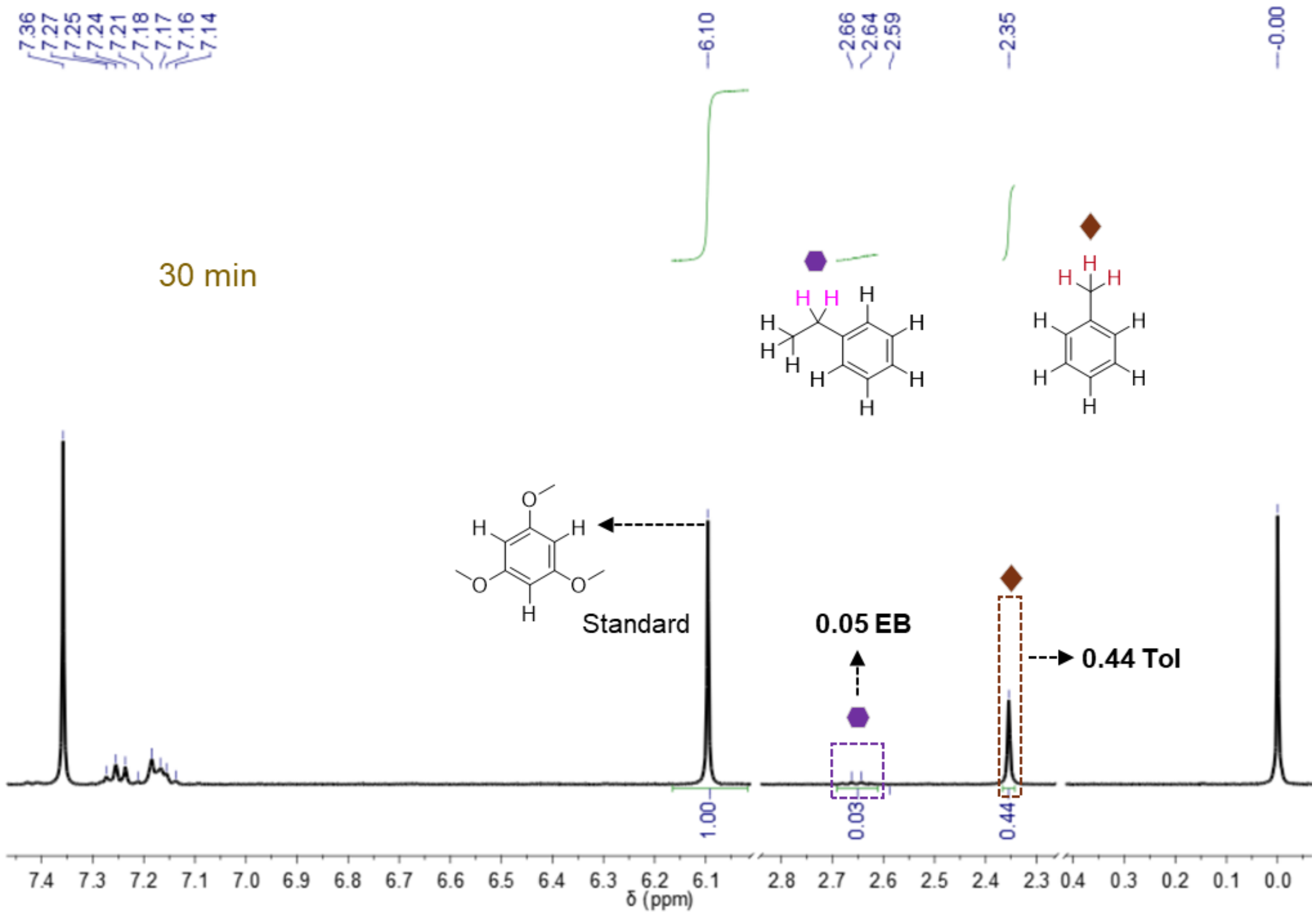

Figure S56. Relative uptake of Tol and EB by **BN-DPTA1-A** after exposure to a 1:1 (mol/mol) binary mixture for 30 minutes, as determined by ^{1}H NMR spectrum (298K, $CDCl_3$, 400 MHz).

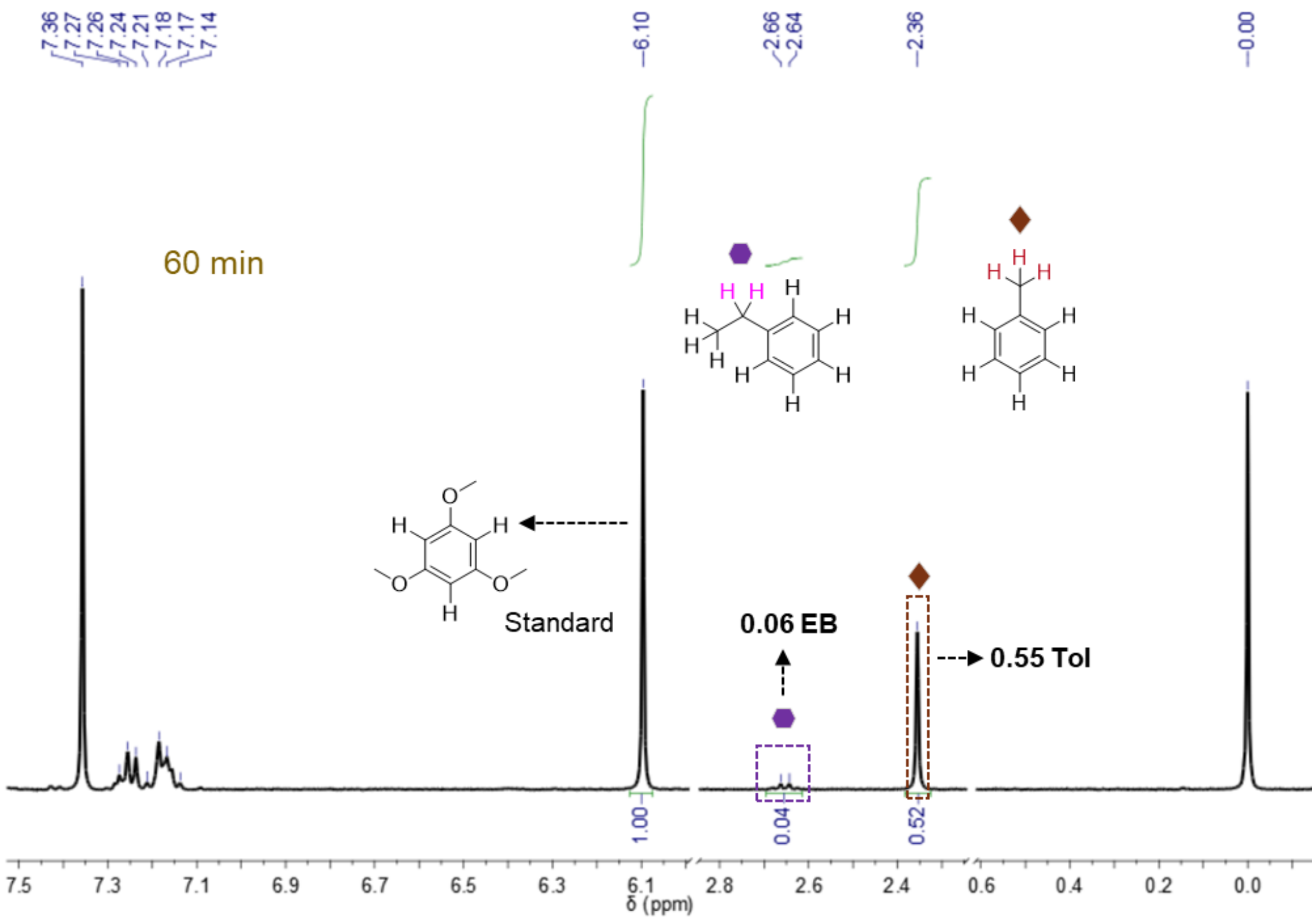

Figure S57. Relative uptake of Tol and EB by **BN-DPTA1-A** after exposure to a 1:1 (mol/mol) binary mixture for 60 minutes, as determined by ^{1}H NMR spectrum (298K, $CDCl_3$, 400 MHz).

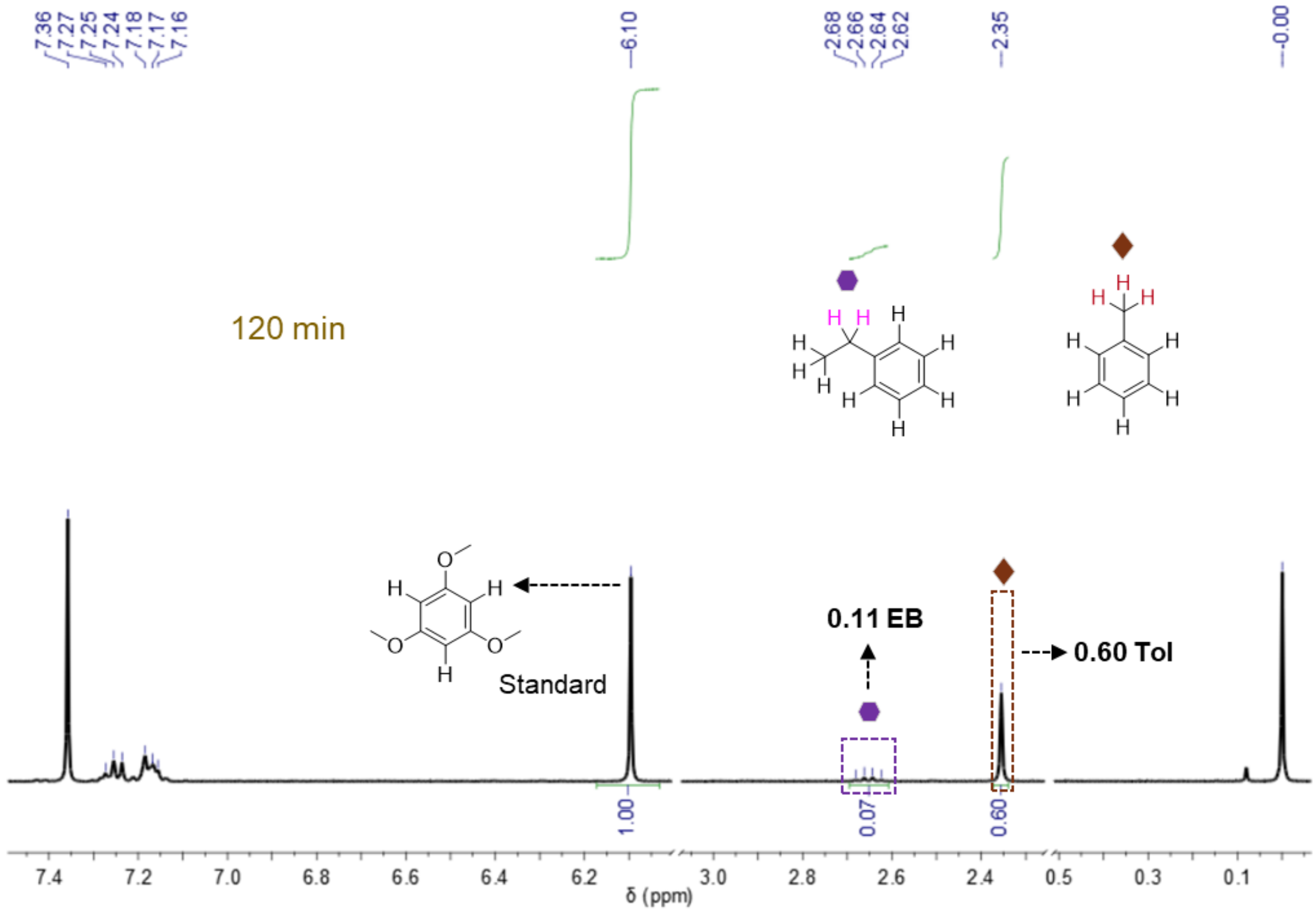

Figure S58. Relative uptake of Tol and EB by **BN-DPTA1-A** after exposure to a 1:1 (mol/mol) binary mixture for 120 minutes, as determined by ^{1}H NMR spectrum (298K, $CDCl_3$, 400 MHz).

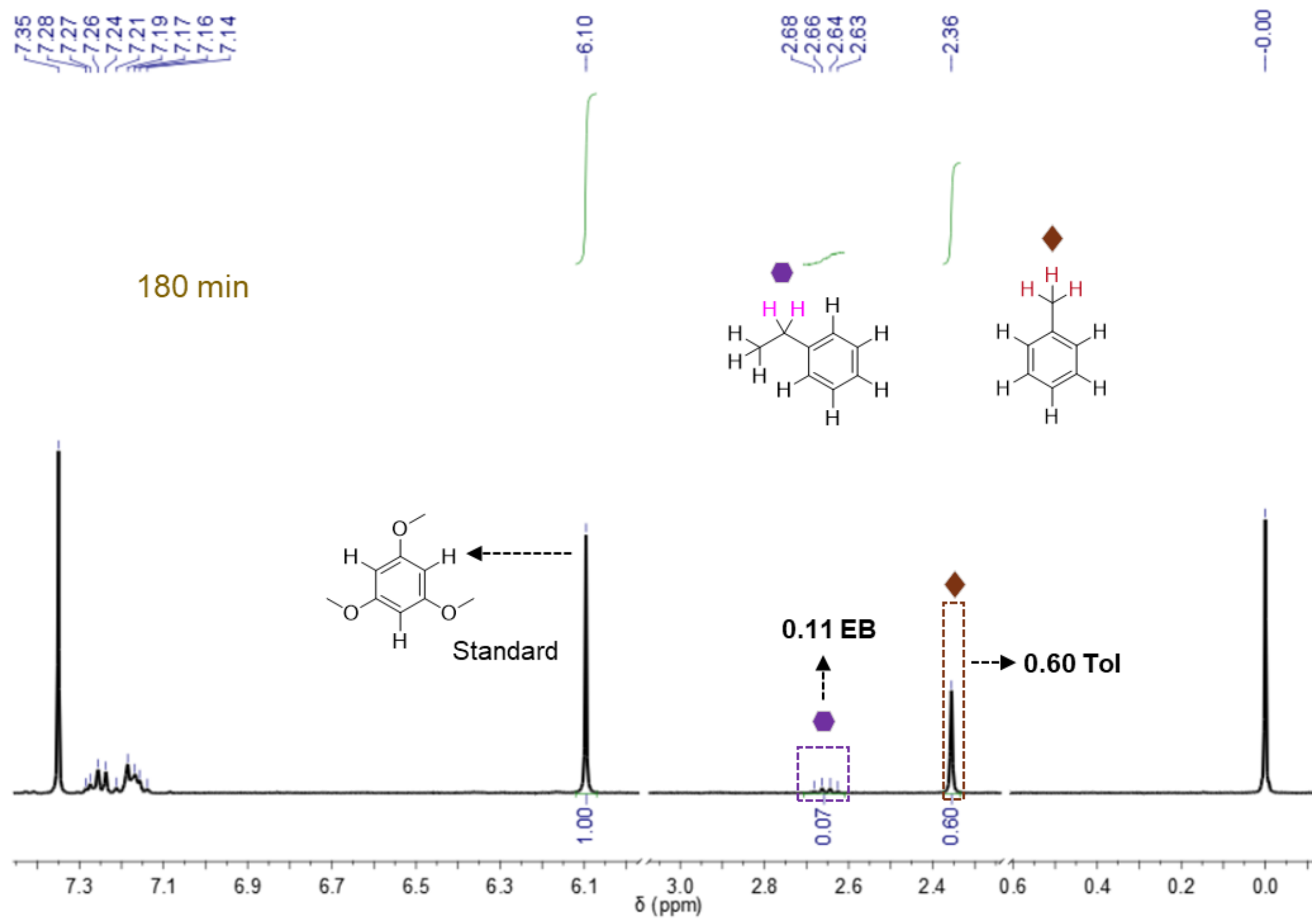

Figure S59. Relative uptake of Tol and EB by **BN-DPTA1-A** after exposure to a 1:1 (mol/mol) binary mixture for 180 minutes, as determined by ^{1}H NMR spectrum (298K, $CDCl_3$, 400 MHz).

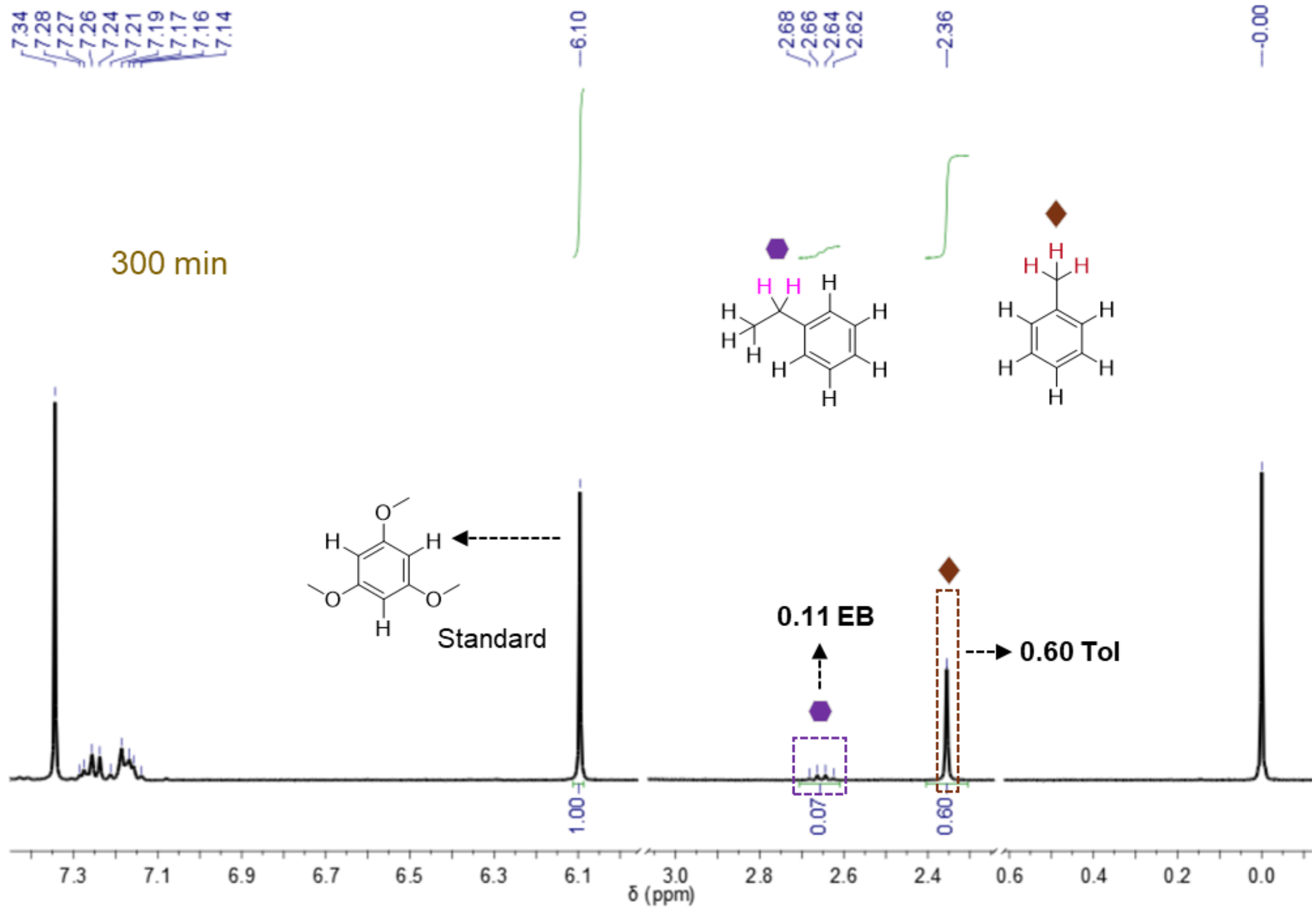

Figure S60. Relative uptake of Tol and EB by **BN-DPTA1-A** after exposure to a 1:1 (mol/mol) binary mixture for 300 minutes, as determined by ^{1}H NMR spectrum (298K, $CDCl_3$, 400 MHz).

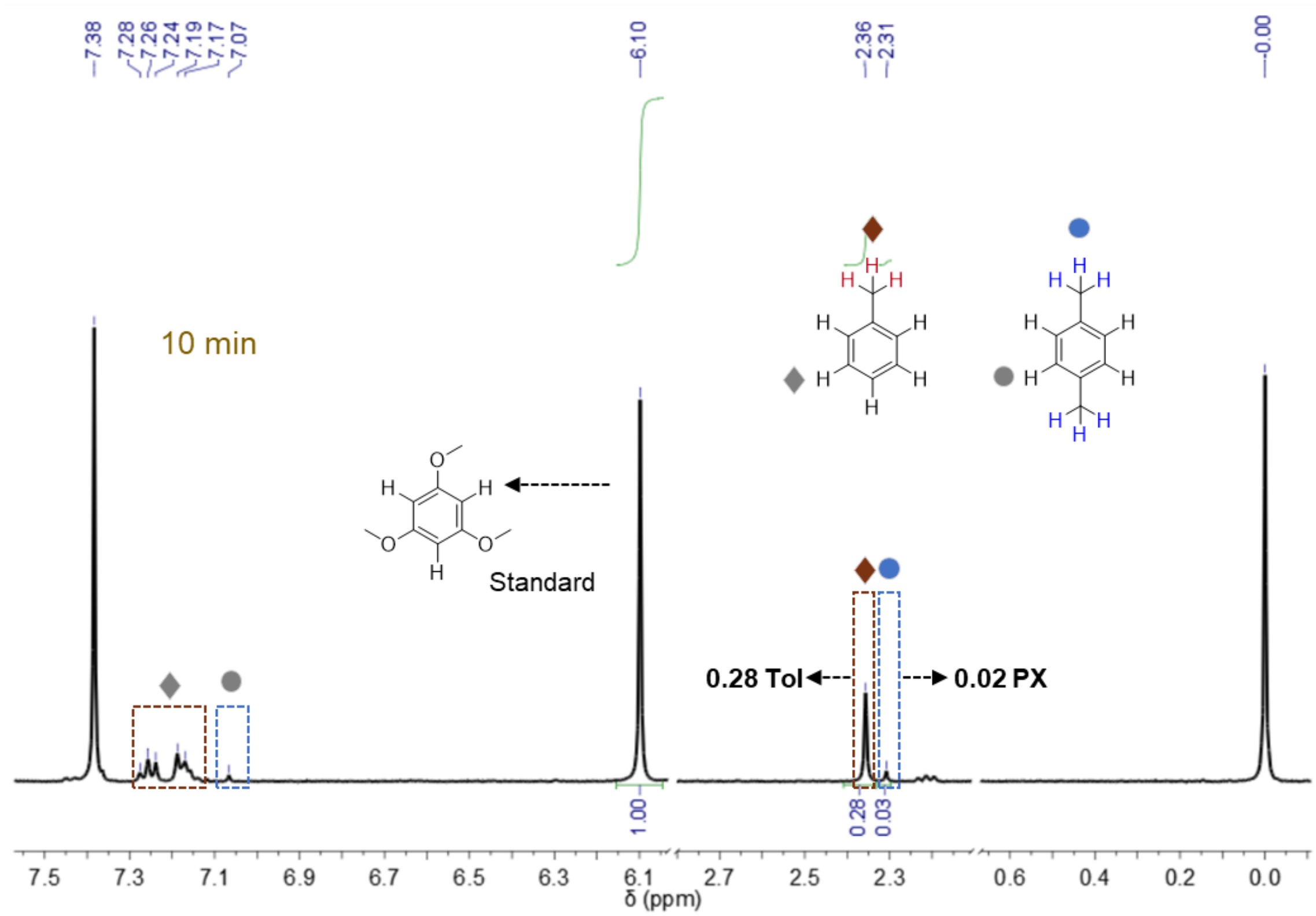

Figure S61. Relative uptake of Tol and PX by **BN-DPTA1-A** after exposure to a 1:1 (mol/mol) binary mixture for 10 minutes, as determined by ^{1}H NMR spectrum (298K, $CDCl_3$, 400 MHz).

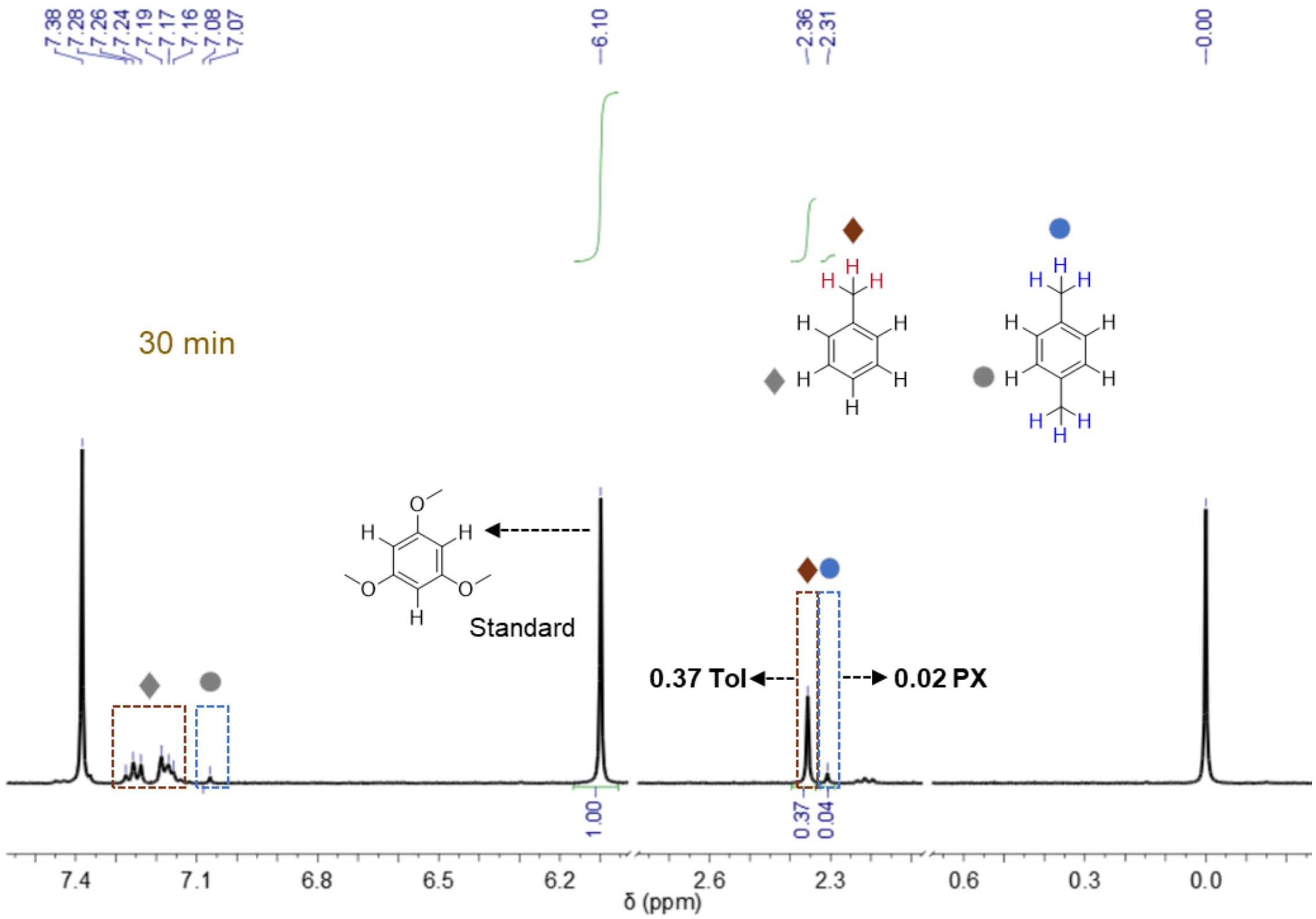

Figure S62. Relative uptake of Tol and PX by **BN-DPTA1-A** after exposure to a 1:1 (mol/mol) binary mixture for 30 minutes, as determined by ^{1}H NMR spectrum (298K, $CDCl_3$, 400 MHz).

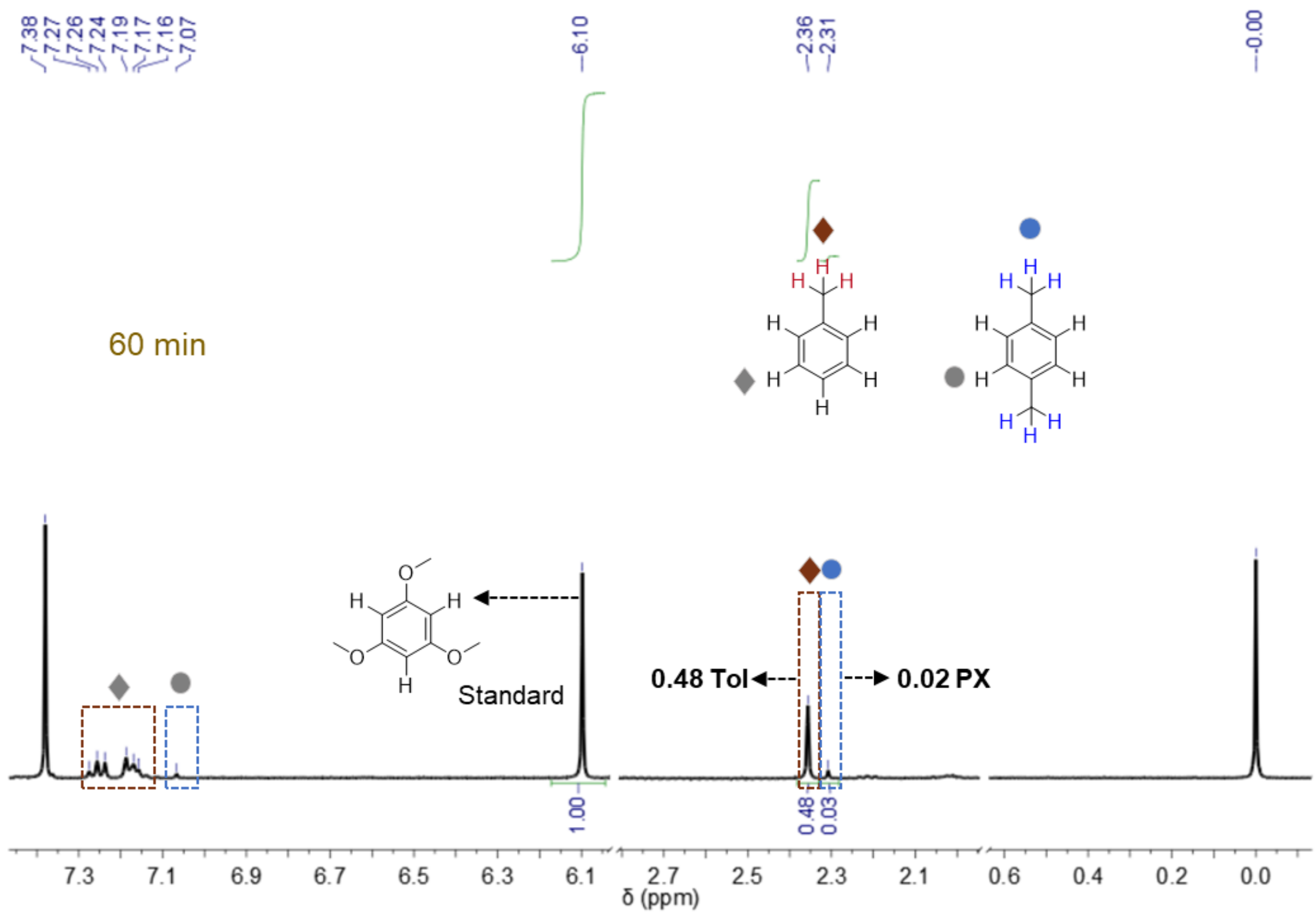

Figure S63. Relative uptake of Tol and PX by **BN-DPTA1-A** after exposure to a 1:1 (mol/mol) binary mixture for 60 minutes, as determined by ^{1}H NMR spectrum (298K, $CDCl_3$, 400 MHz).

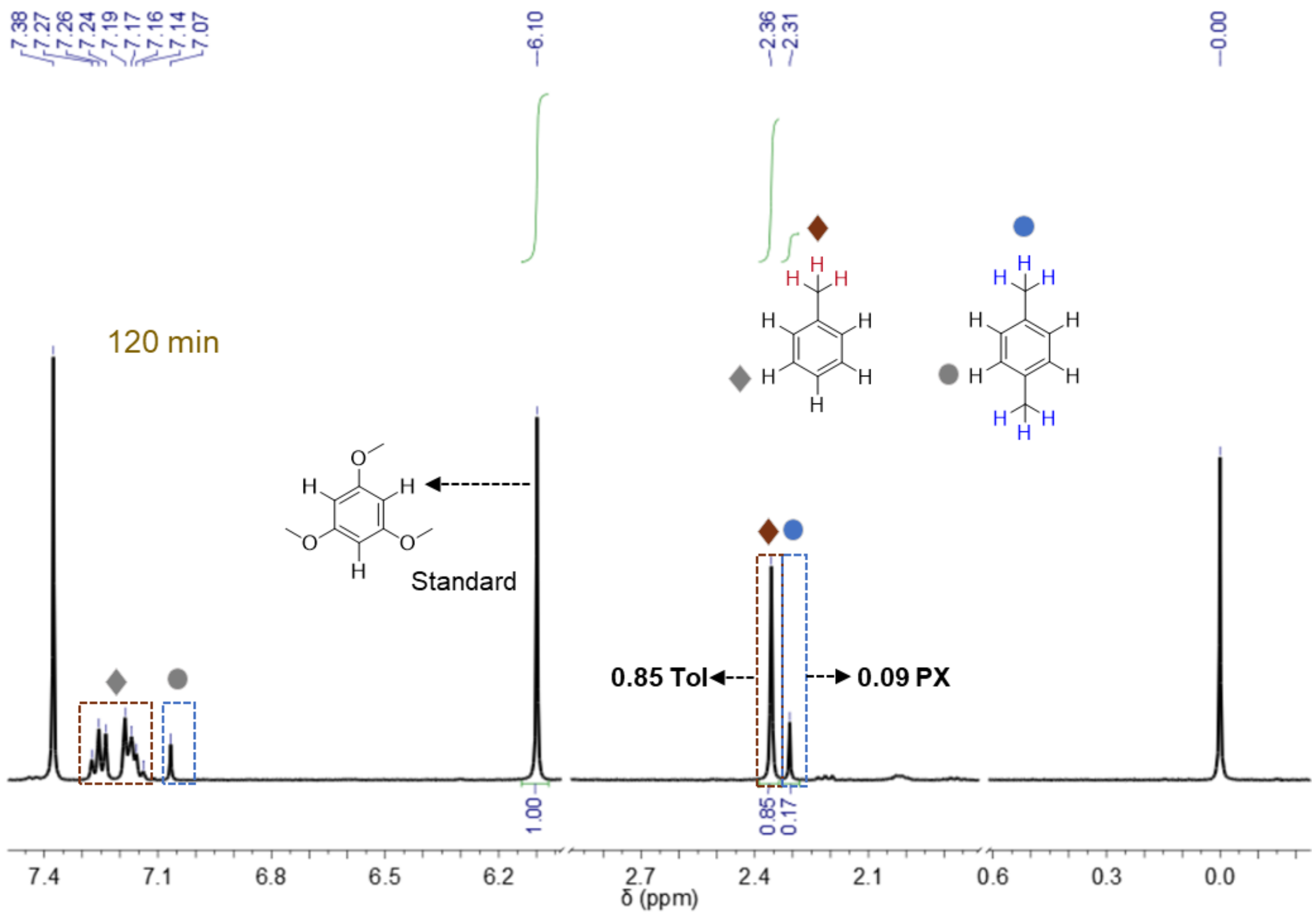

Figure S64. Relative uptake of Tol and PX by **BN-DPTA1-A** after exposure to a 1:1 (mol/mol) binary mixture for 120 minutes, as determined by ^{1}H NMR spectrum (298K, $CDCl_3$, 400 MHz).

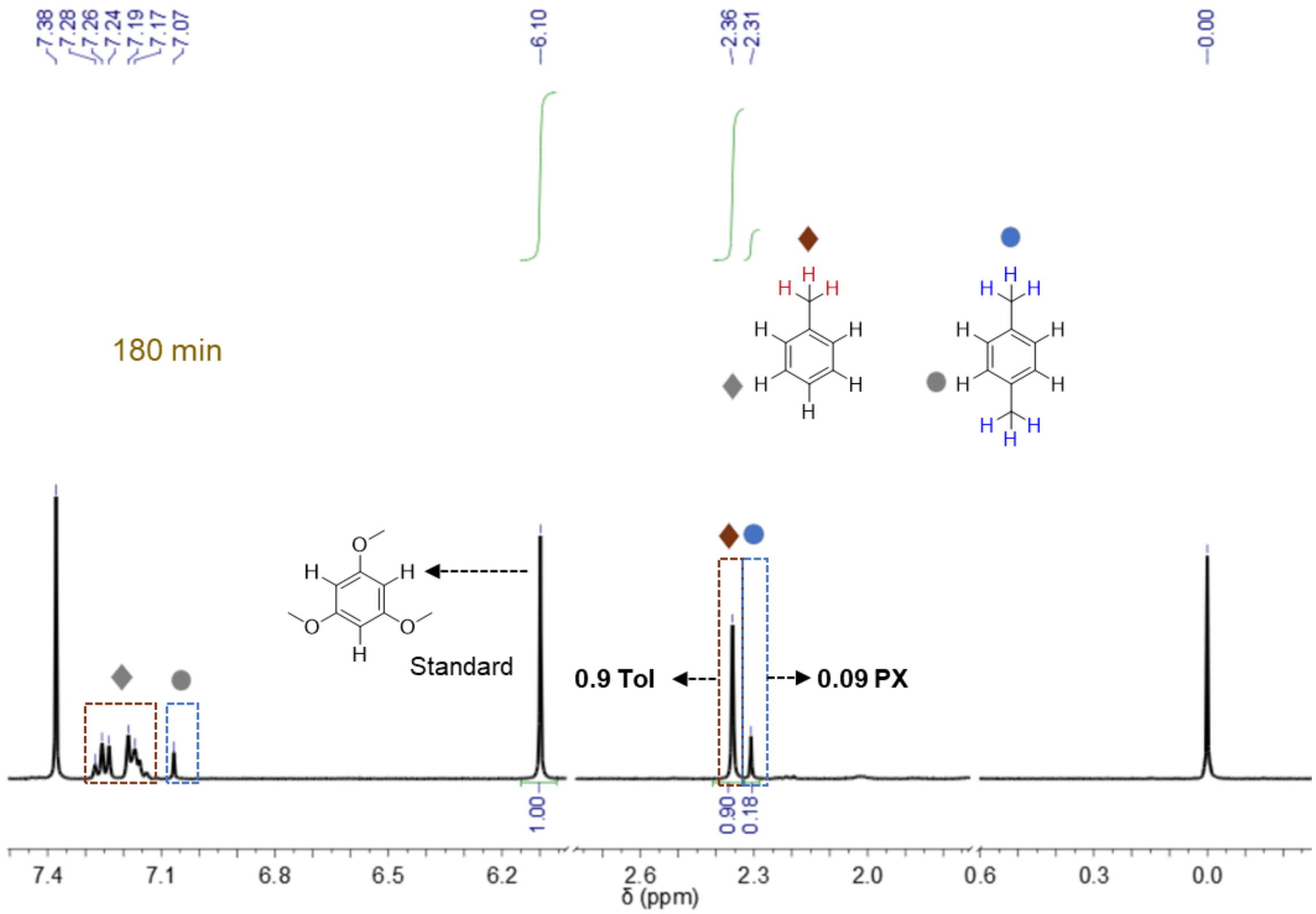

Figure S65. Relative uptake of Tol and PX by **BN-DPTA1-A** after exposure to a 1:1 (mol/mol) binary mixture for 180 minutes, as determined by ^{1}H NMR spectrum (298K, $CDCl_3$, 400 MHz).

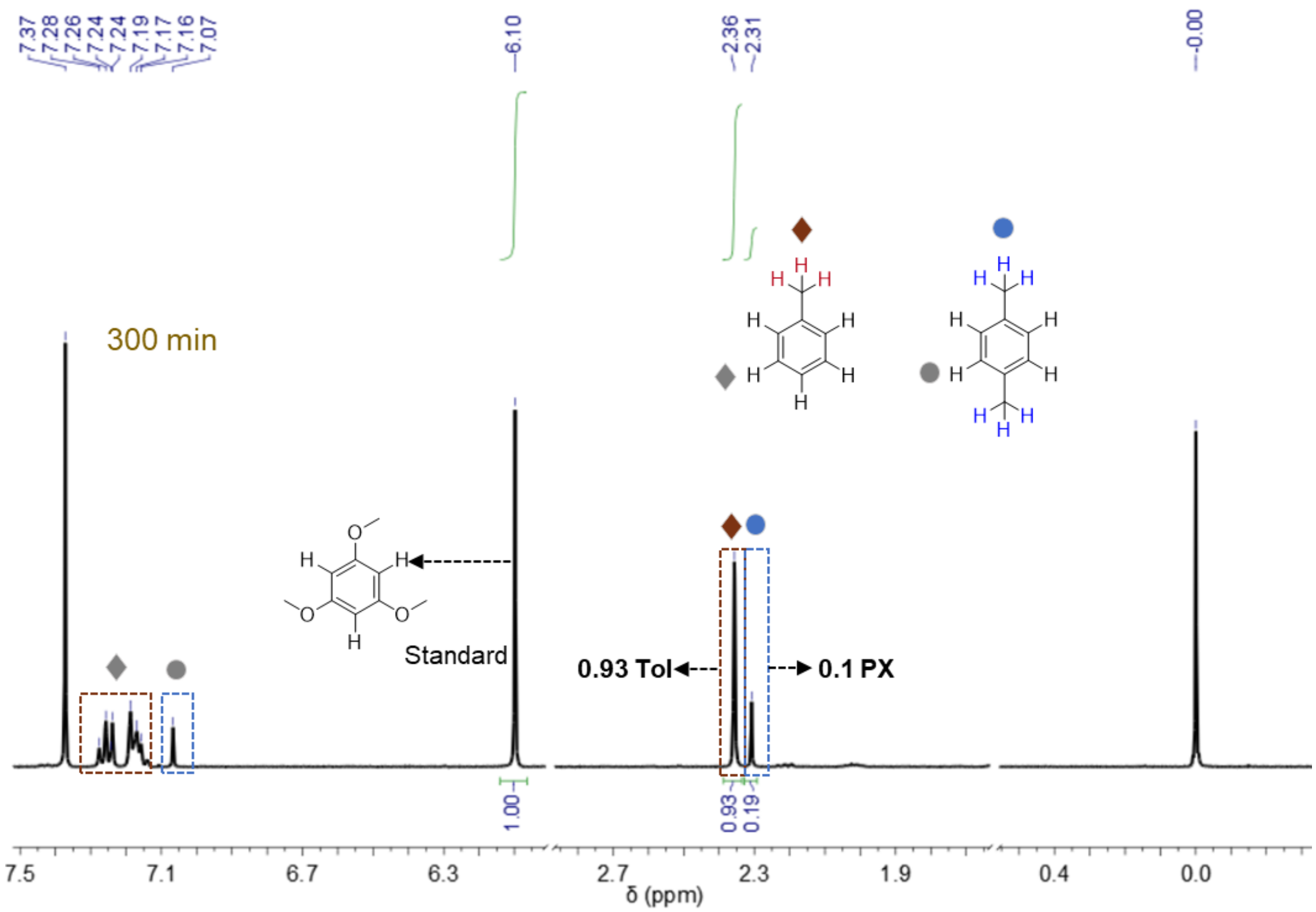

Figure S66. Relative uptake of Tol and PX by **BN-DPTA1-A** after exposure to a 1:1 (mol/mol) binary mixture for 300 minutes, as determined by ^{1}H NMR spectrum (298K, $CDCl_3$, 400 MHz).

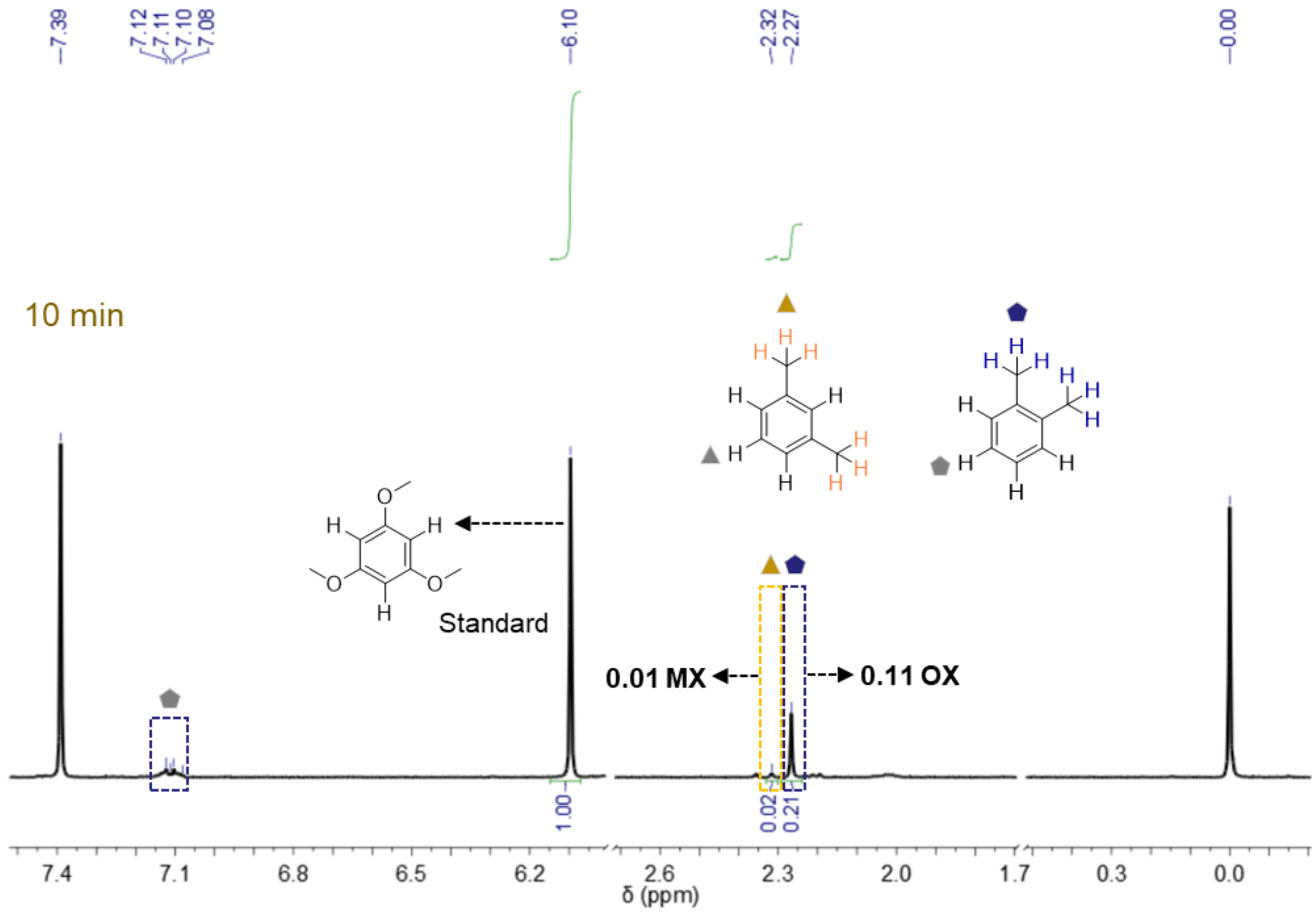

Figure S67. Relative uptake of OX and MX by **BN-DPTA2-A** after exposure to a 1:1 (mol/mol) binary mixture for 10 minutes, as determined by ^{1}H NMR spectrum (298K, $CDCl_3$, 400 MHz).

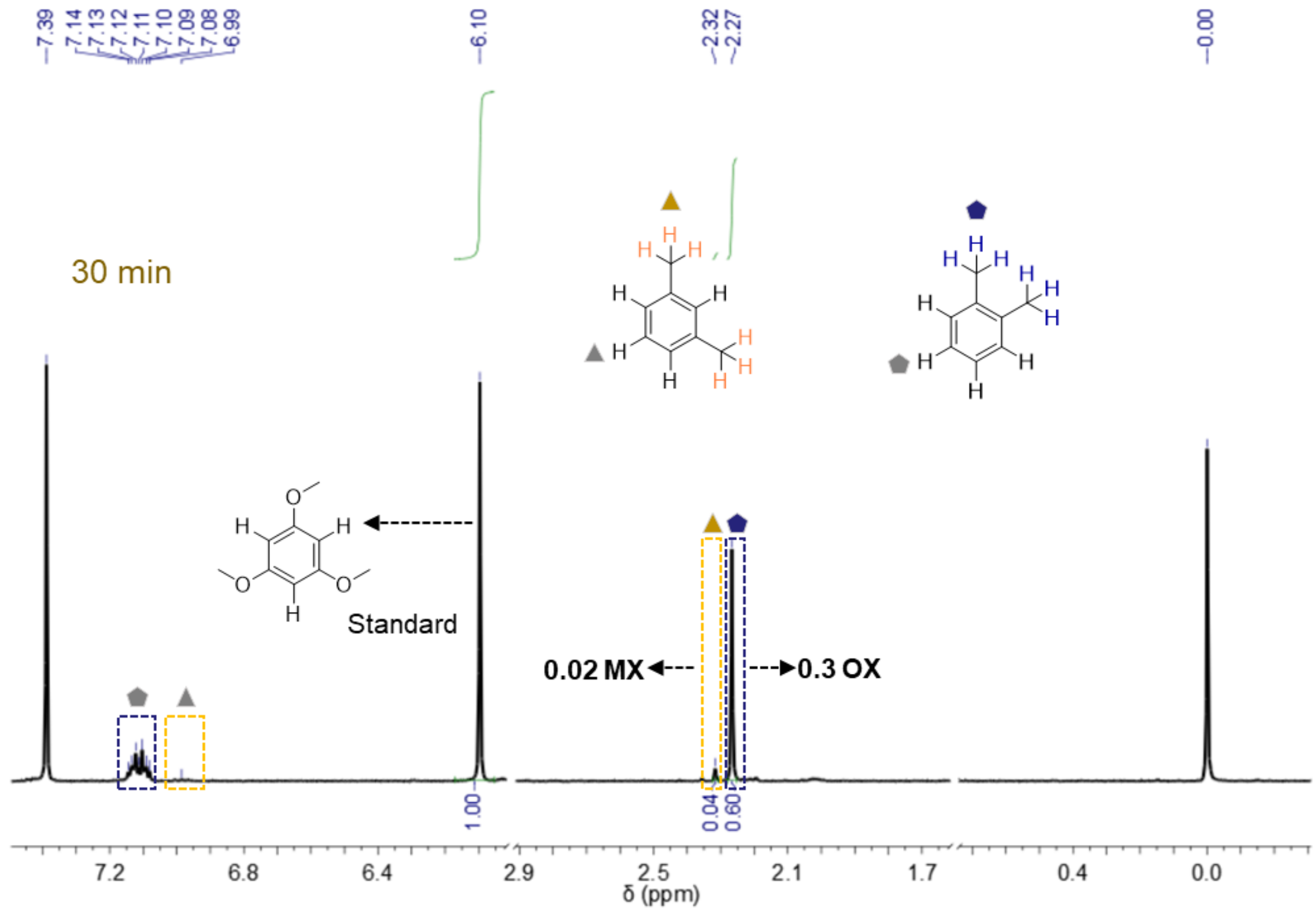

Figure S68. Relative uptake of OX and MX by **BN-DPTA2-A** after exposure to a 1:1 (mol/mol) binary mixture for 30 minutes, as determined by ^{1}H NMR spectrum (298K, $CDCl_3$, 400 MHz).

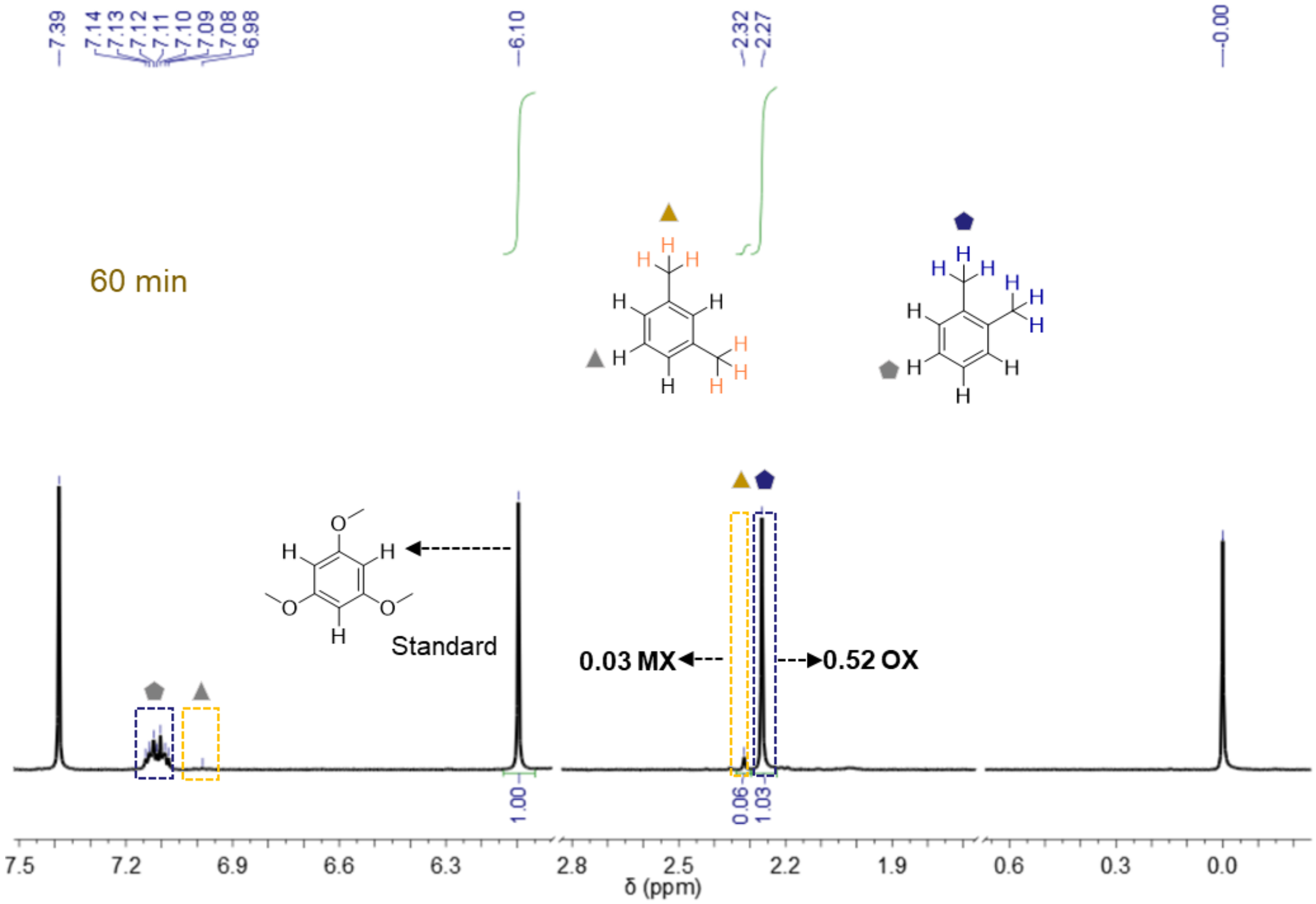

Figure S69. Relative uptake of OX and MX by **BN-DPTA2-A** after exposure to a 1:1 (mol/mol) binary mixture for 60 minutes, as determined by ^{1}H NMR spectrum (298K, $CDCl_3$, 400 MHz).

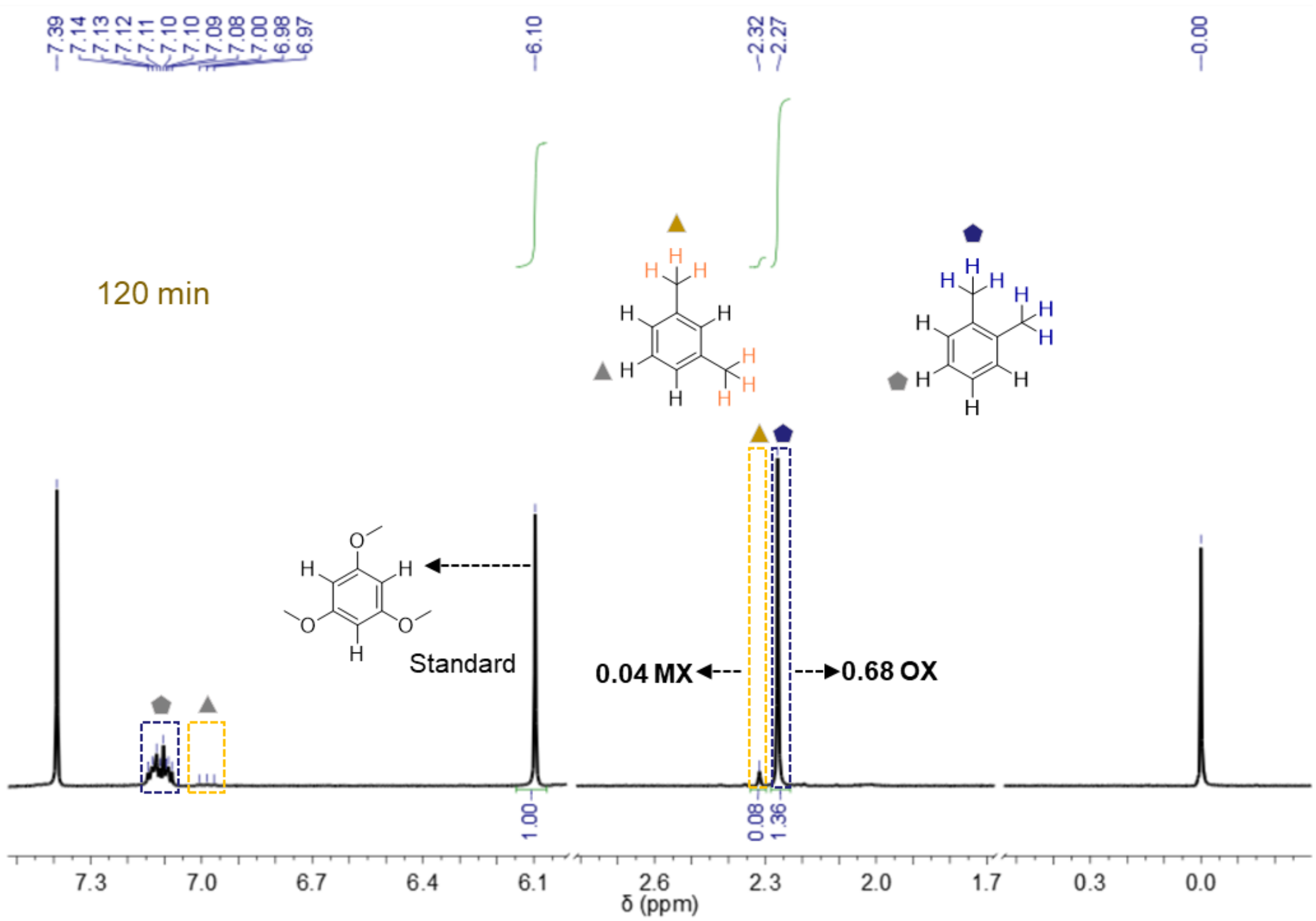

Figure S70. Relative uptake of OX and MX by **BN-DPTA2-A** after exposure to a 1:1 (mol/mol) binary mixture for 120 minutes, as determined by ^{1}H NMR spectrum (298K, $CDCl_3$, 400 MHz).

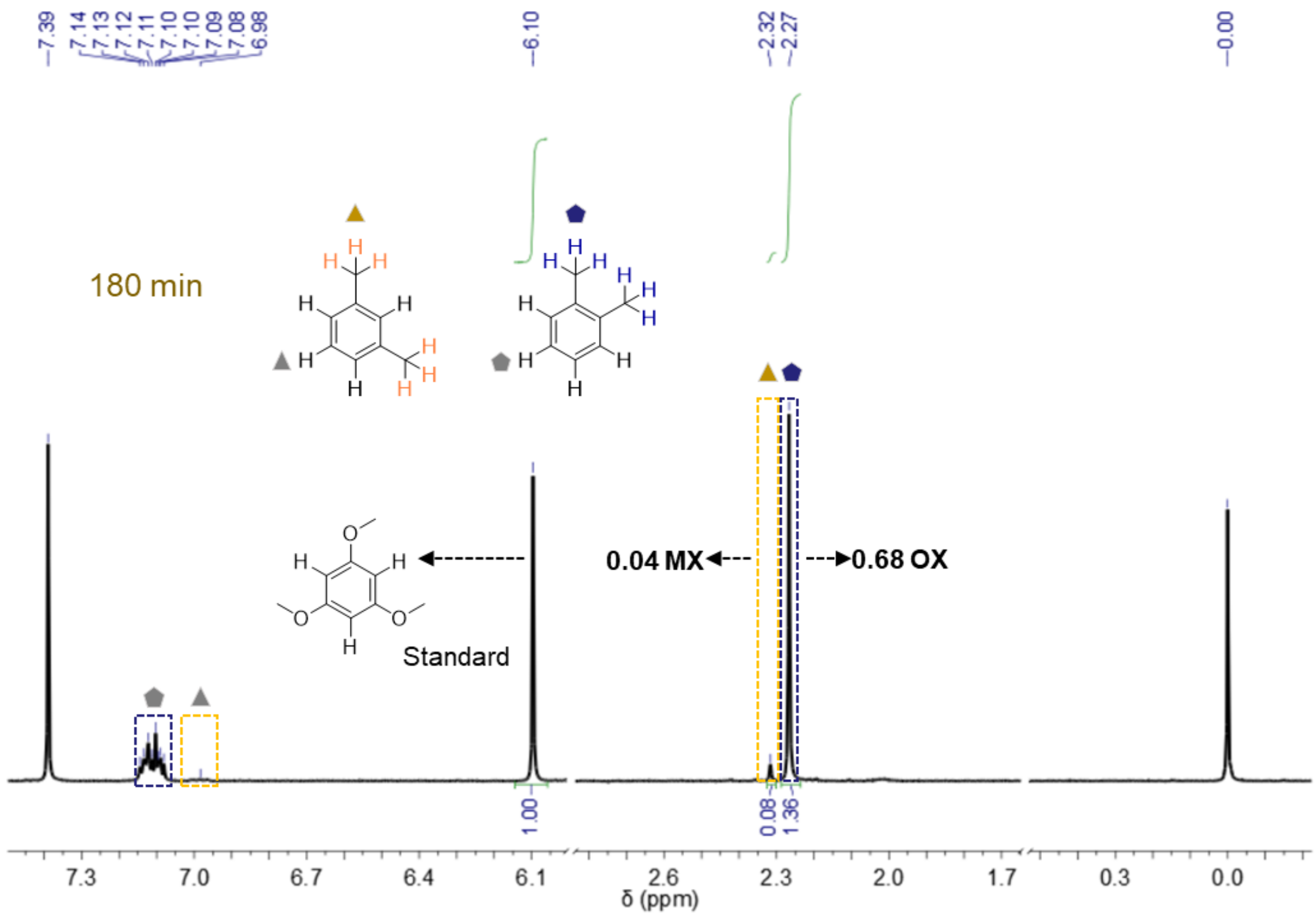

Figure S71. Relative uptake of OX and MX by **BN-DPTA2-A** after exposure to a 1:1 (mol/mol) binary mixture for 180 minutes, as determined by ^{1}H NMR spectrum (298K, $CDCl_3$, 400 MHz).

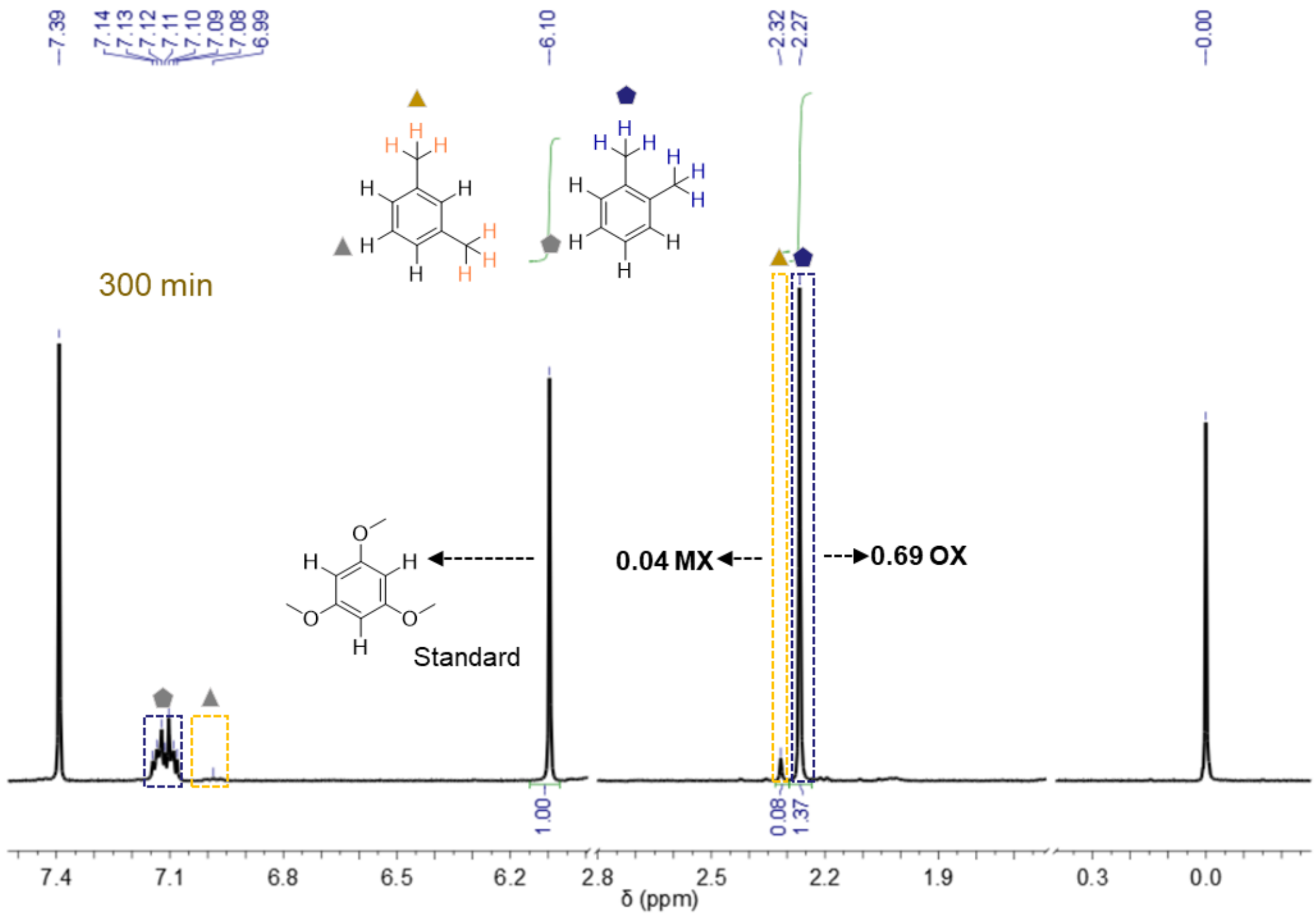

Figure S72. Relative uptake of OX and MX by **BN-DPTA2-A** after exposure to a 1:1 (mol/mol) binary mixture for 300 minutes, as determined by ^{1}H NMR spectrum (298K, $CDCl_3$, 400 MHz).

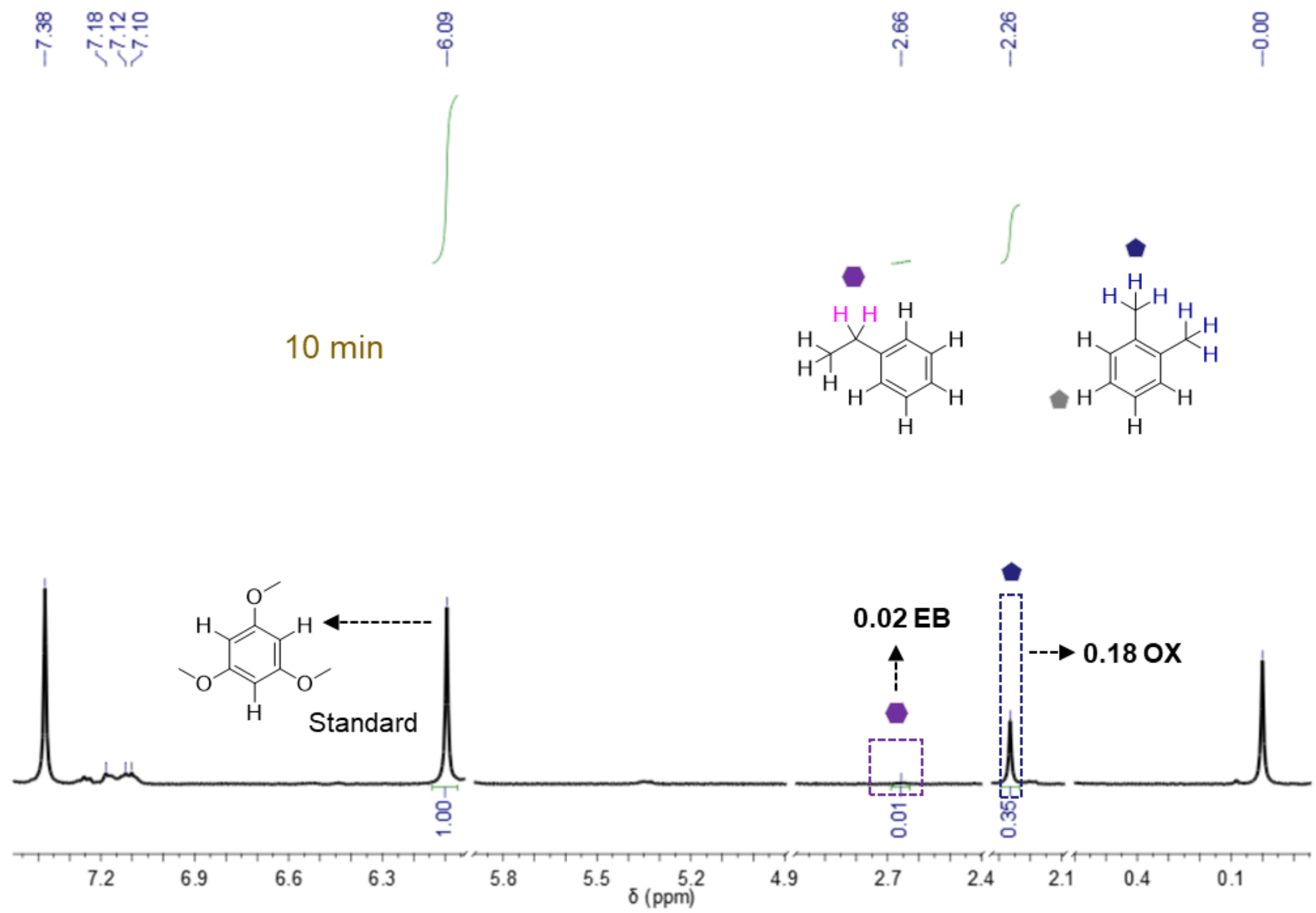

Figure S73. Relative uptake of OX and EB by **BN-DPTA2-A** after exposure to a 1:1 (mol/mol) binary mixture for 10 minutes, as determined by ^{1}H NMR spectrum (298K, $CDCl_3$, 400 MHz).

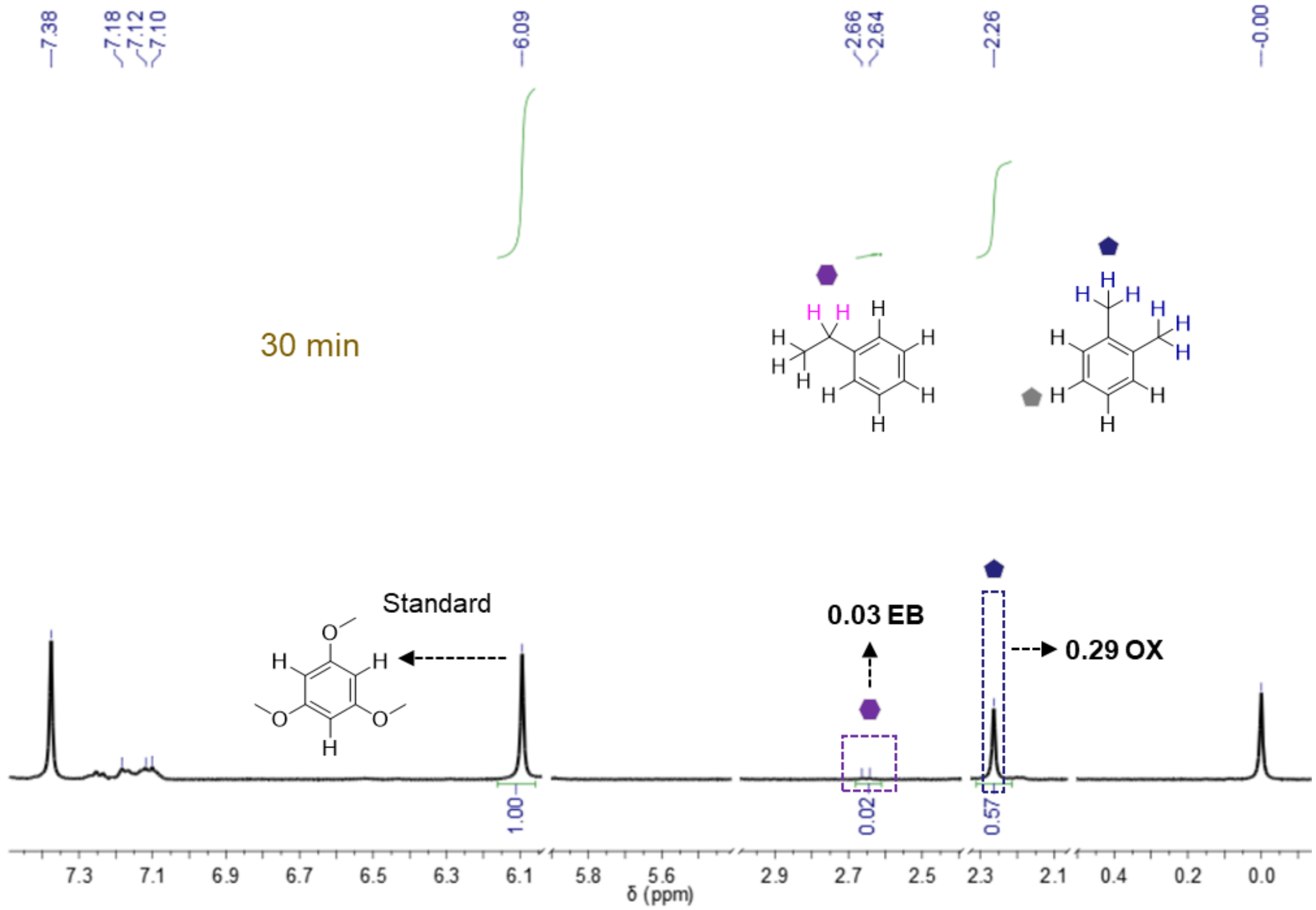

Figure S74. Relative uptake of OX and EB by **BN-DPTA2-A** after exposure to a 1:1 (mol/mol) binary mixture for 30 minutes, as determined by ^{1}H NMR spectrum (298K, $CDCl_3$, 400 MHz).

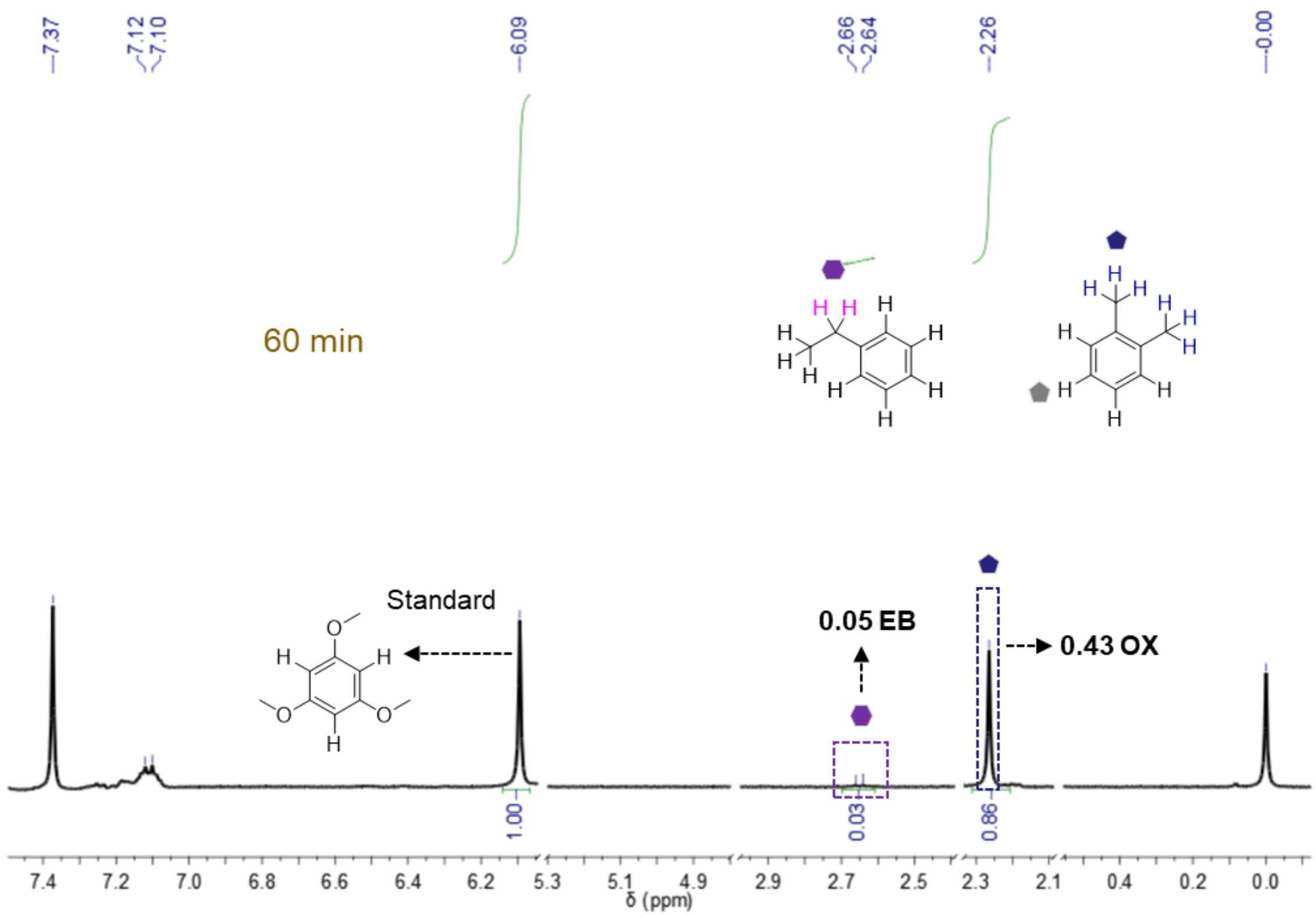

Figure S75. Relative uptake of OX and EB by **BN-DPTA2-A** after exposure to a 1:1 (mol/mol) binary mixture for 60 minutes, as determined by ^{1}H NMR spectrum (298K, $CDCl_3$, 400 MHz).

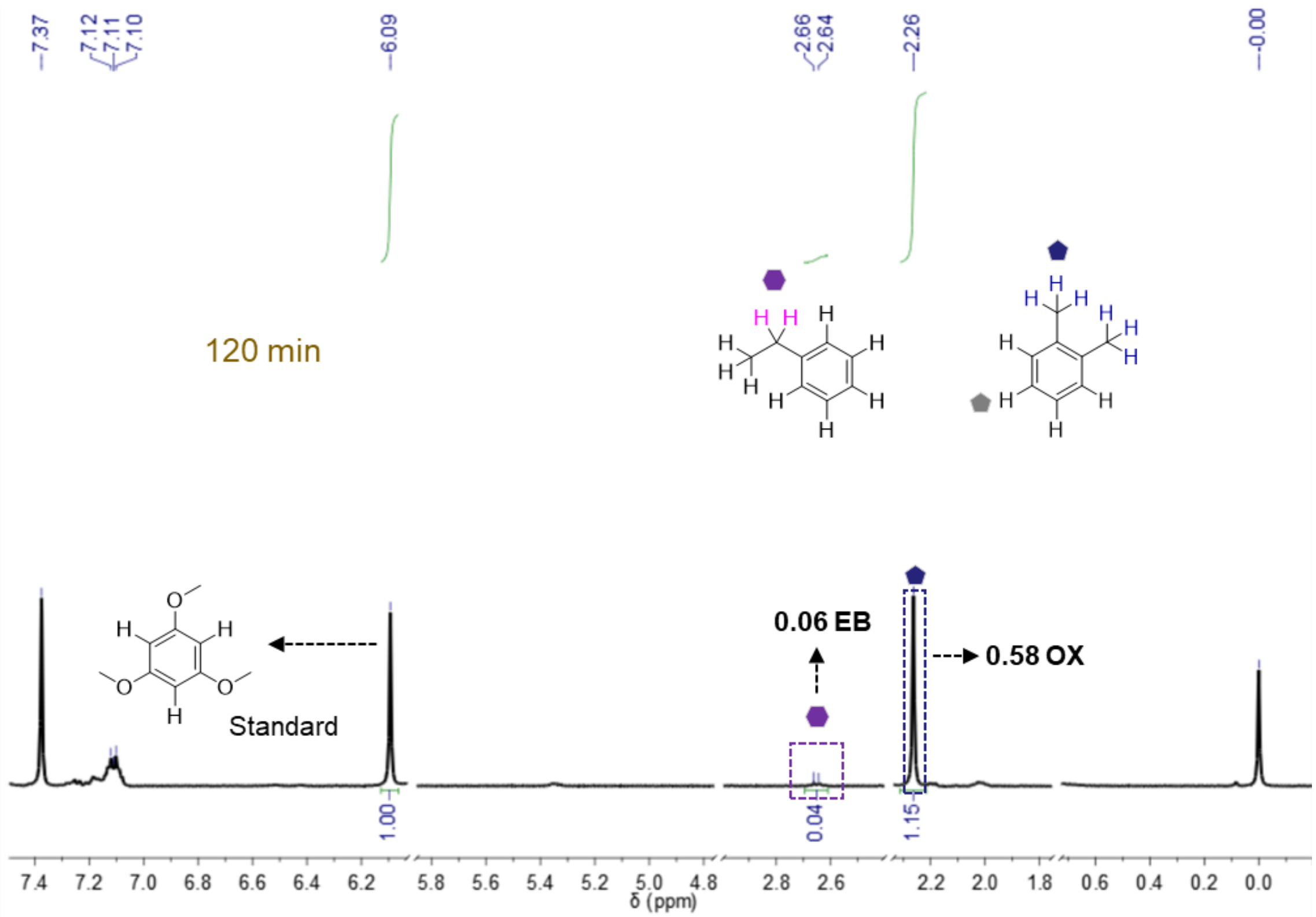

Figure S76. Relative uptake of OX and EB by **BN-DPTA2-A** after exposure to a 1:1 (mol/mol) binary mixture for 120 minutes, as determined by ^{1}H NMR spectrum (298K, $CDCl_3$, 400 MHz).

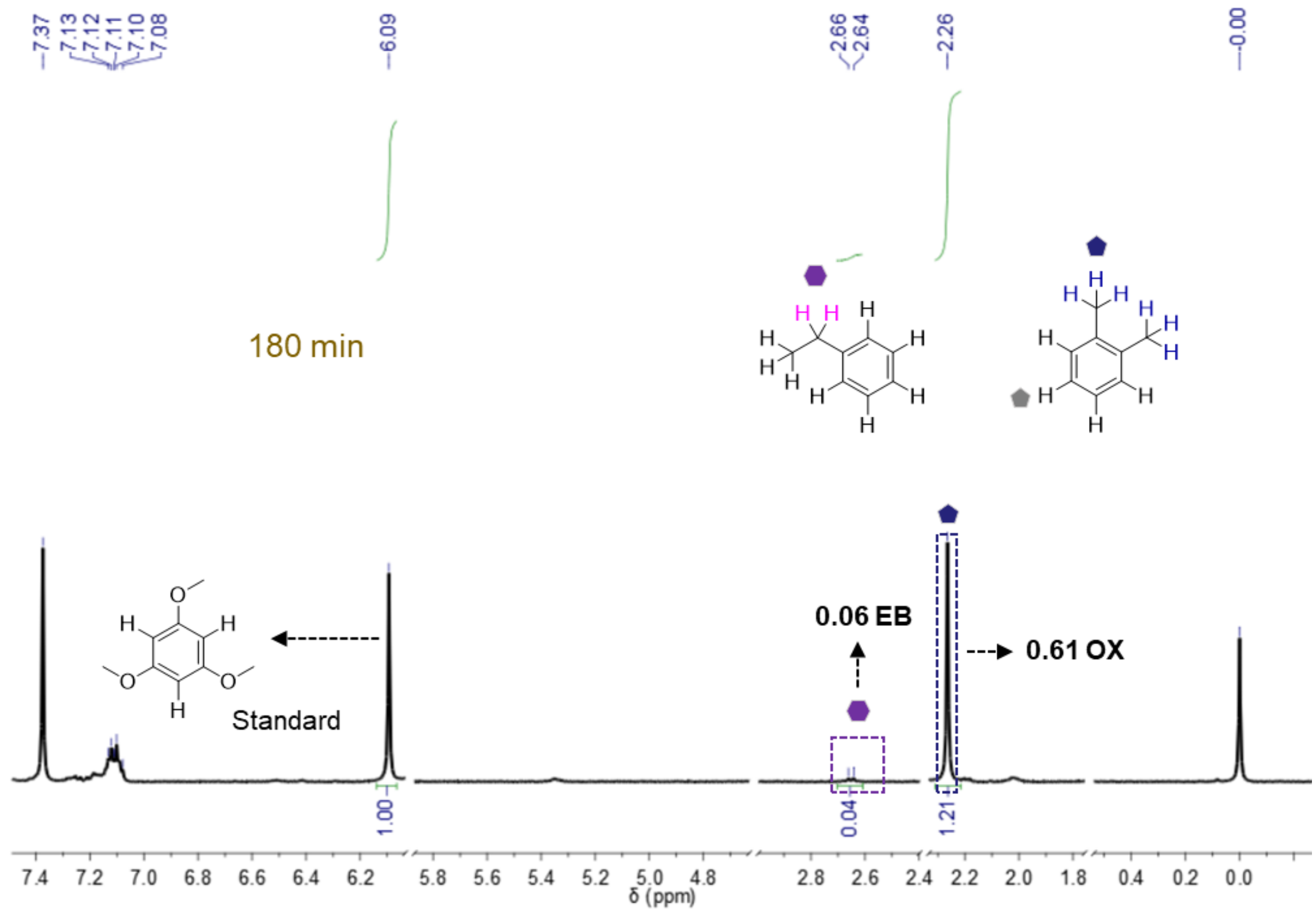

Figure S77. Relative uptake of OX and EB by **BN-DPTA2-A** after exposure to a 1:1 (mol/mol) binary mixture for 180 minutes, as determined by ^{1}H NMR spectrum (298K, $CDCl_3$, 400 MHz).

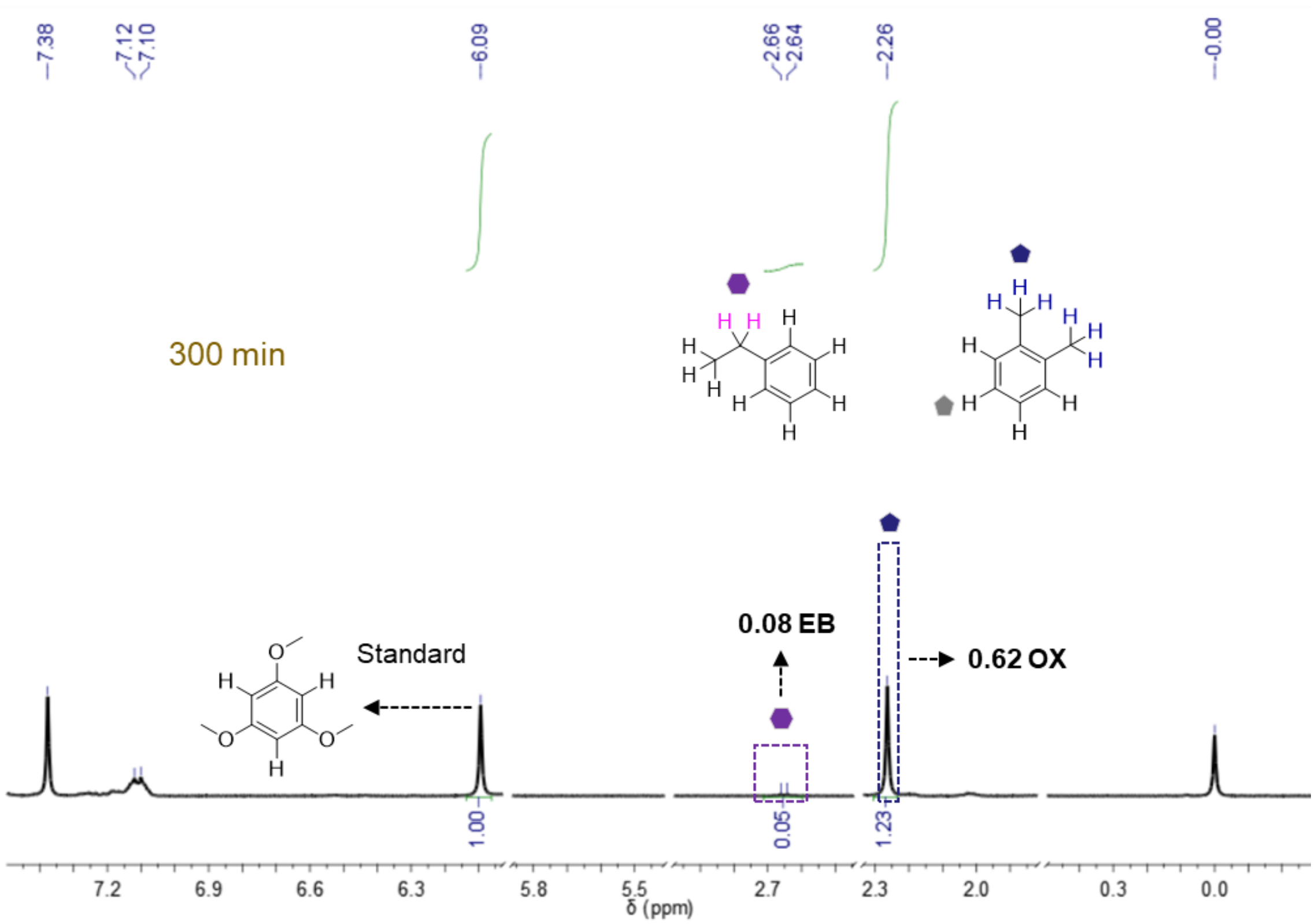

Figure S78. Relative uptake of OX and EB by **BN-DPTA2-A** after exposure to a 1:1 (mol/mol) binary mixture for 300 minutes, as determined by ^{1}H NMR spectrum (298K, $CDCl_3$, 400 MHz).

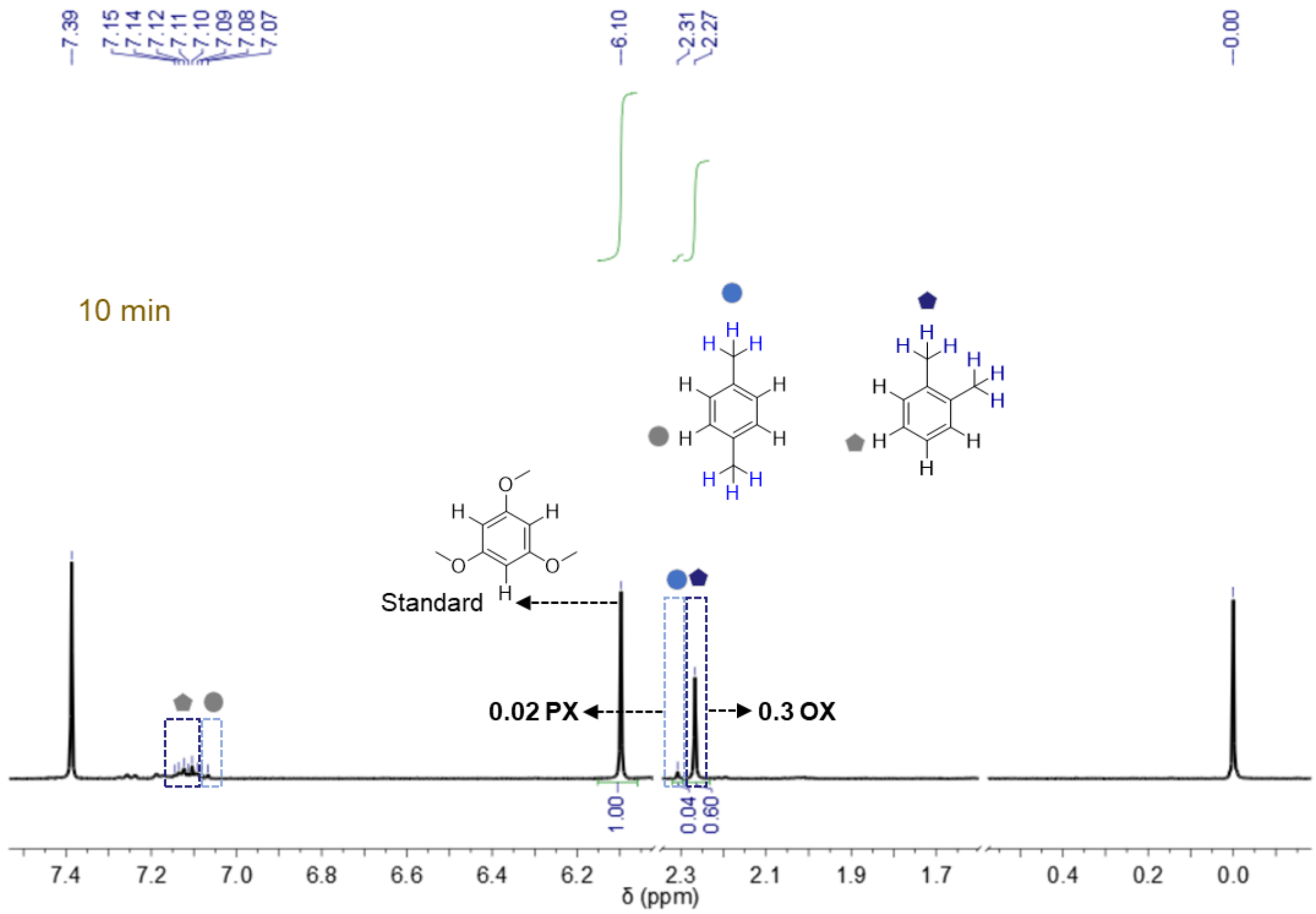

Figure S79. Relative uptake of OX and PX by **BN-DPTA2-A** after exposure to a 1:1 (mol/mol) binary mixture for 10 minutes, as determined by ^{1}H NMR spectrum (298K, $CDCl_3$, 400 MHz).

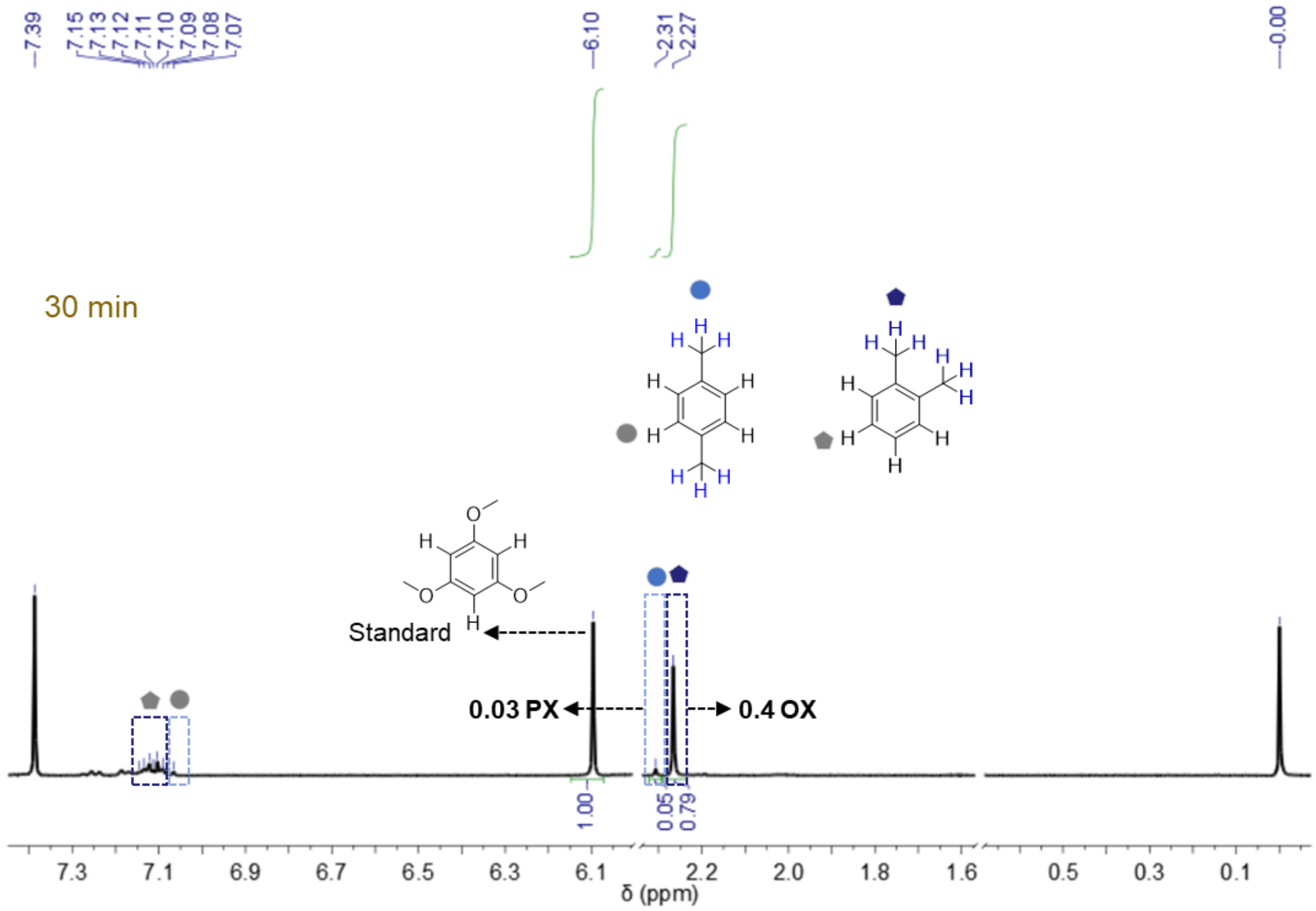

Figure S80. Relative uptake of OX and PX by **BN-DPTA2-A** after exposure to a 1:1 (mol/mol) binary mixture for 30 minutes, as determined by ^{1}H NMR spectrum (298K, $CDCl_3$, 400 MHz).

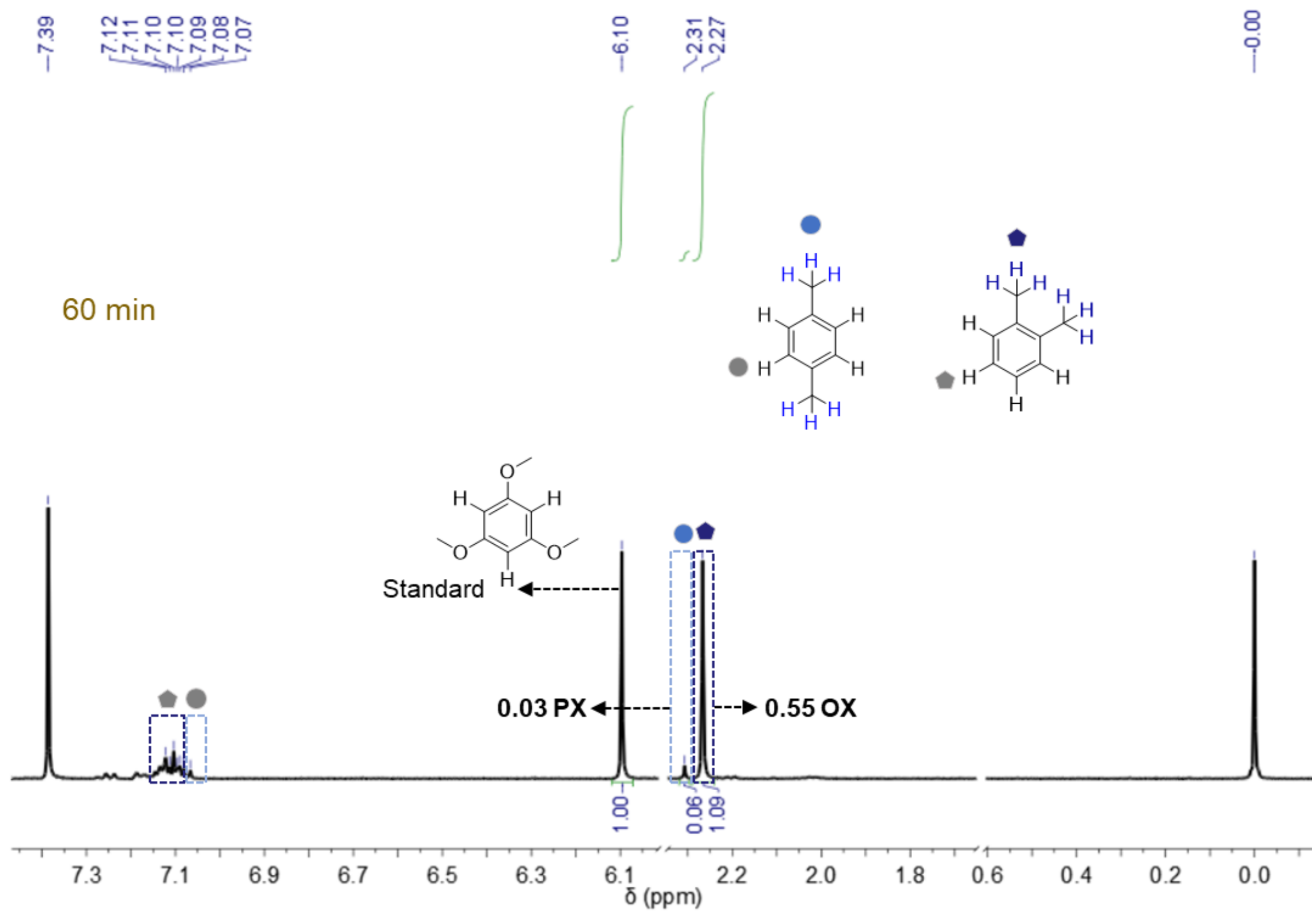

Figure S81. Relative uptake of OX and PX by **BN-DPTA2-A** after exposure to a 1:1 (mol/mol) binary mixture for 60 minutes, as determined by ^{1}H NMR spectrum (298K, $CDCl_3$, 400 MHz).

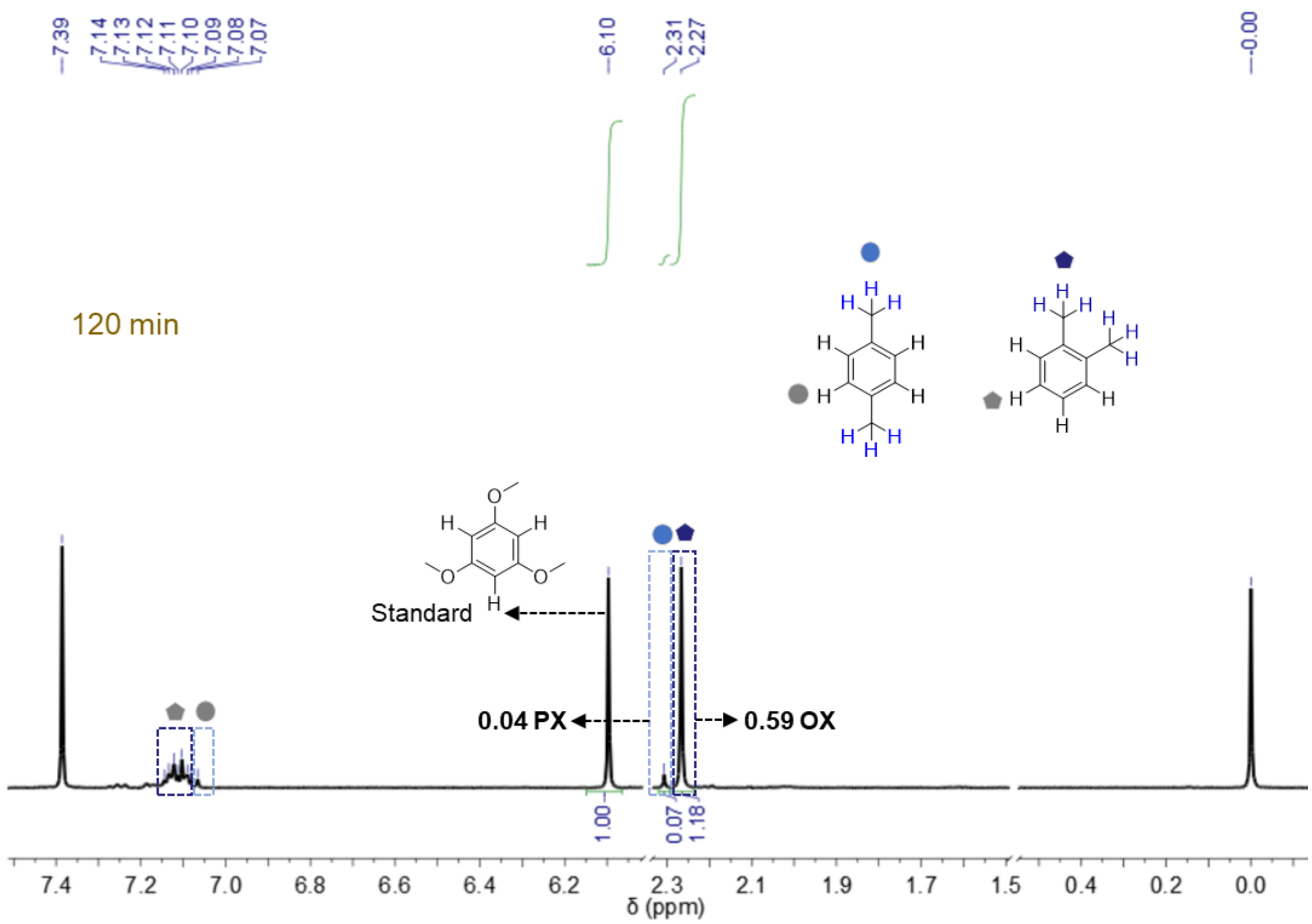

Figure S82. Relative uptake of OX and PX by **BN-DPTA2-A** after exposure to a 1:1 (mol/mol) binary mixture for 120 minutes, as determined by ^{1}H NMR spectrum (298K, $CDCl_3$, 400 MHz).

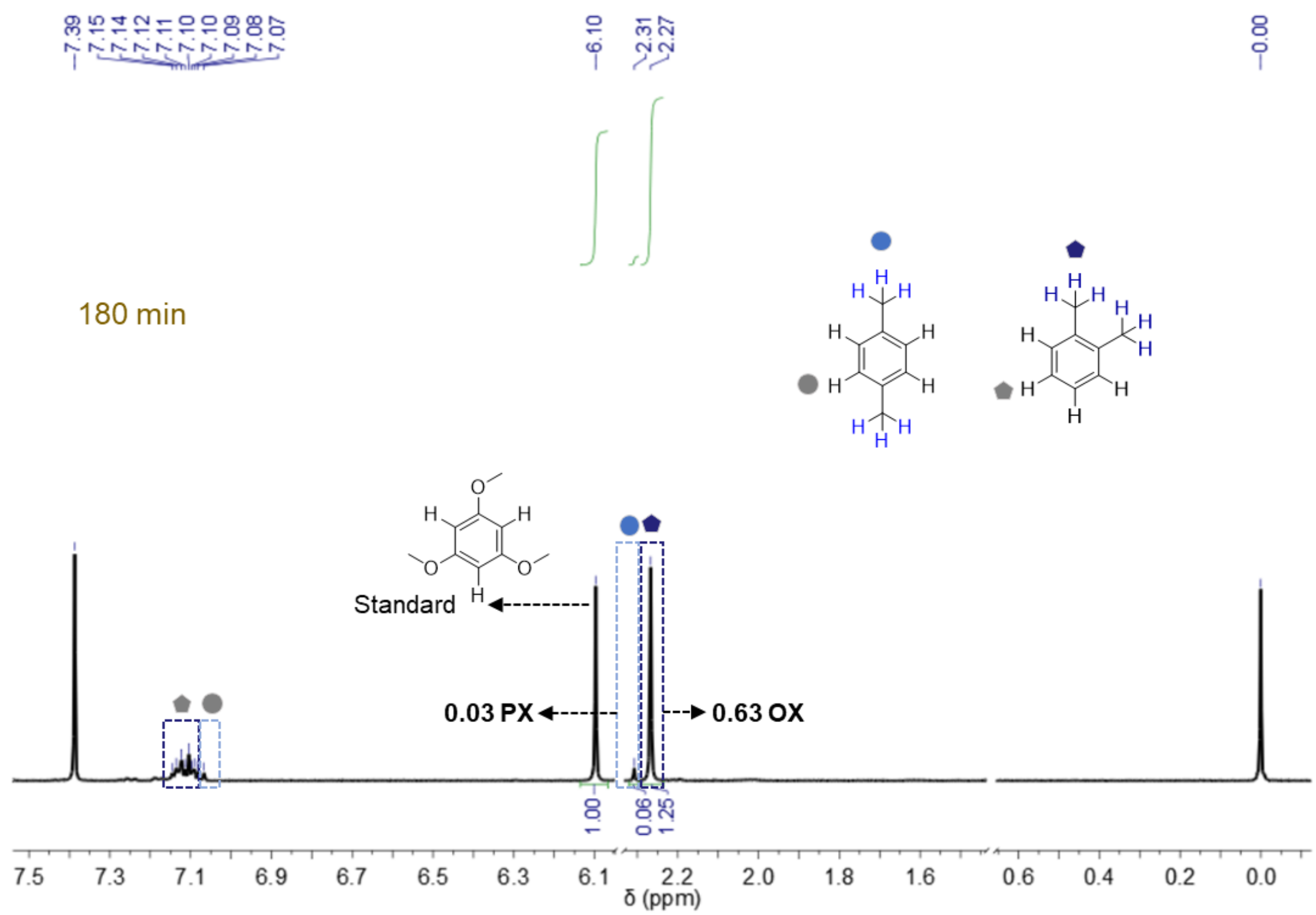

Figure S83. Relative uptake of OX and PX by **BN-DPTA2-A** after exposure to a 1:1 (mol/mol) binary mixture for 180 minutes, as determined by ^{1}H NMR spectrum (298K, $CDCl_3$, 400 MHz).

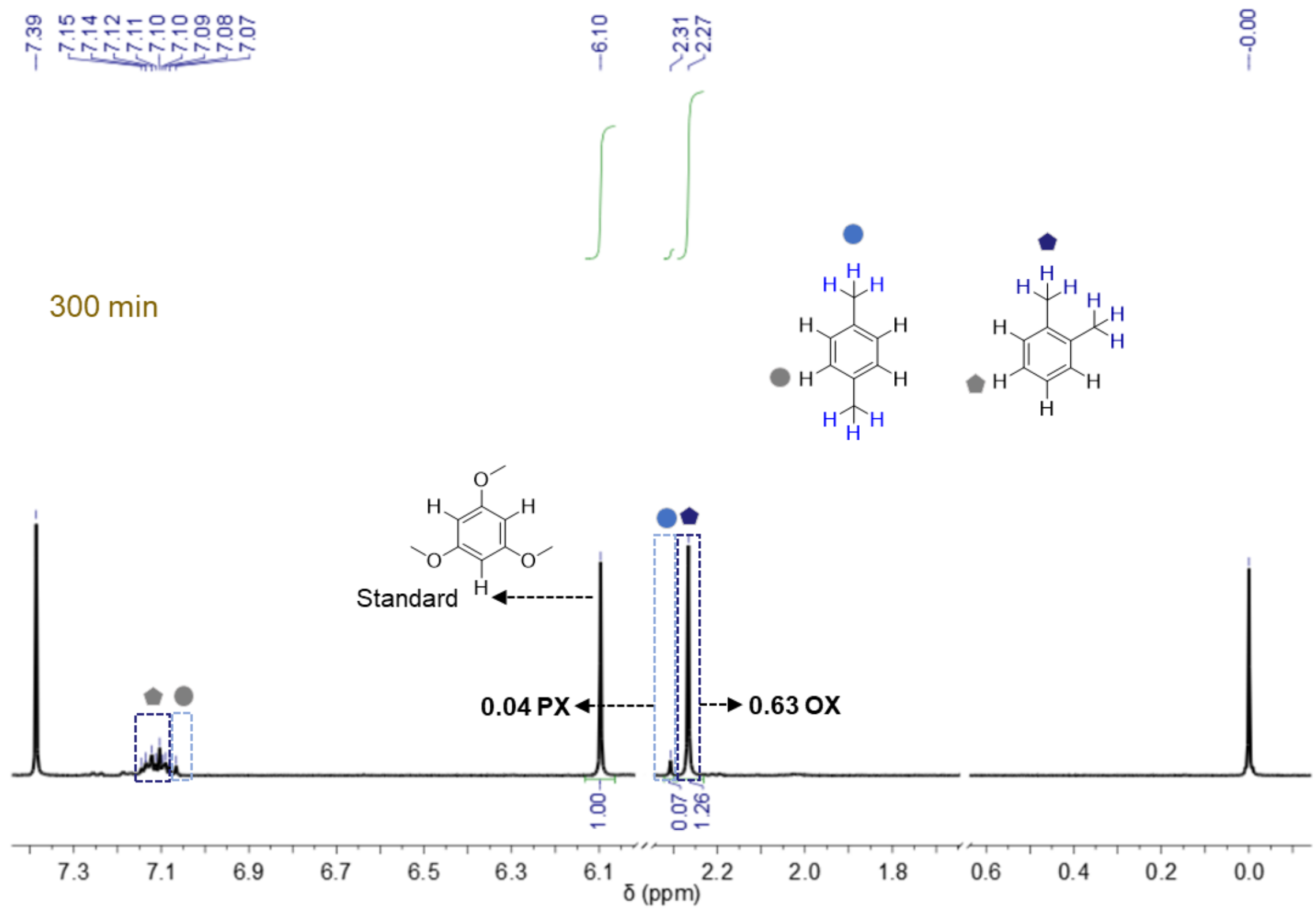

Figure S84. Relative uptake of OX and PX by **BN-DPTA2-A** after exposure to a 1:1 (mol/mol) binary mixture for 300 minutes, as determined by ^{1}H NMR spectrum (298K, $CDCl_3$, 400 MHz).

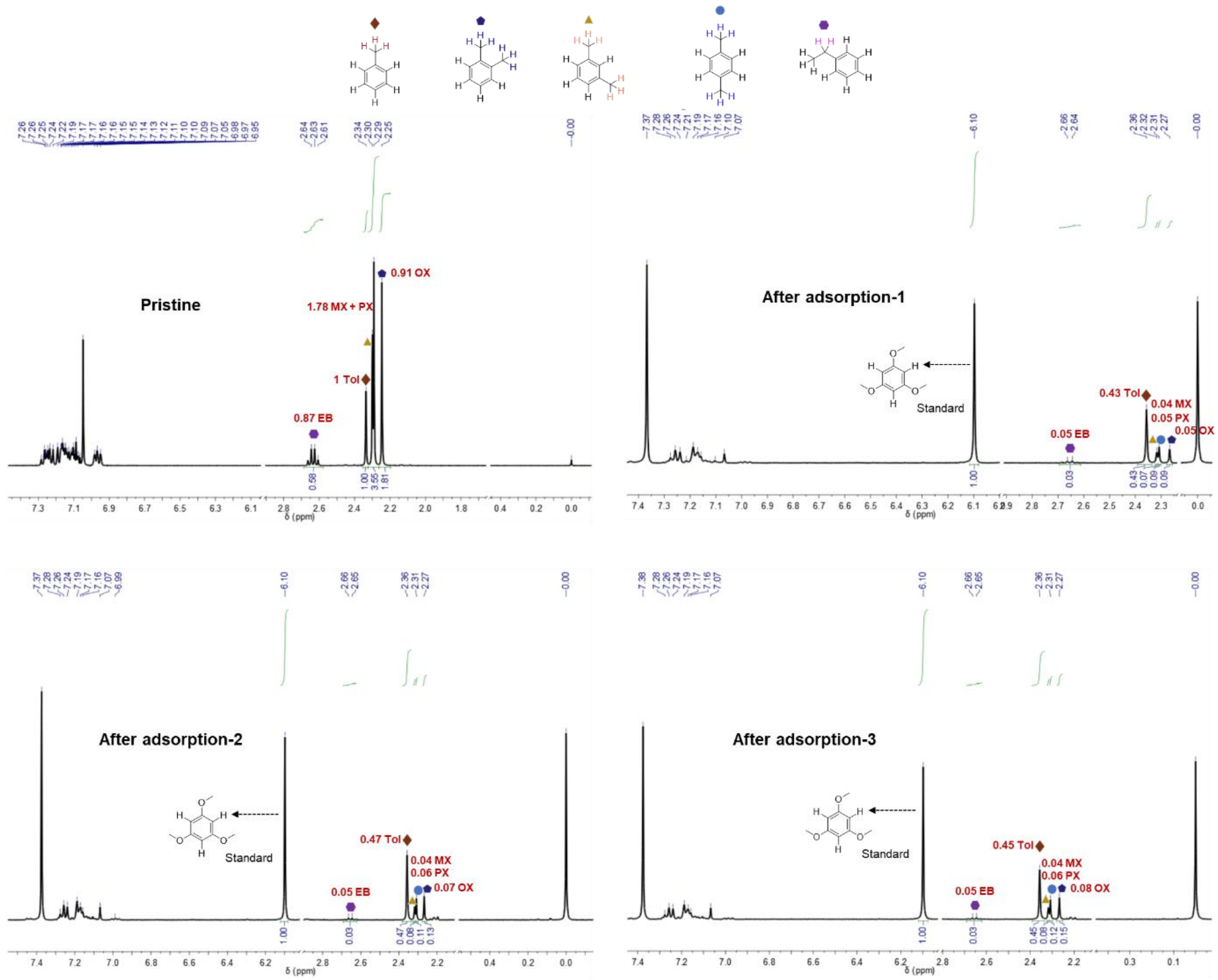

Figure S85. Relative uptake of Tol, OX, MX, EB, and PX by **BN-DPTA1-A** after exposure to an equimolar quinary mixture for 300 min, as determined by ^{1}H NMR spectra (298 K, $CDCl_3$, 400 MHz).

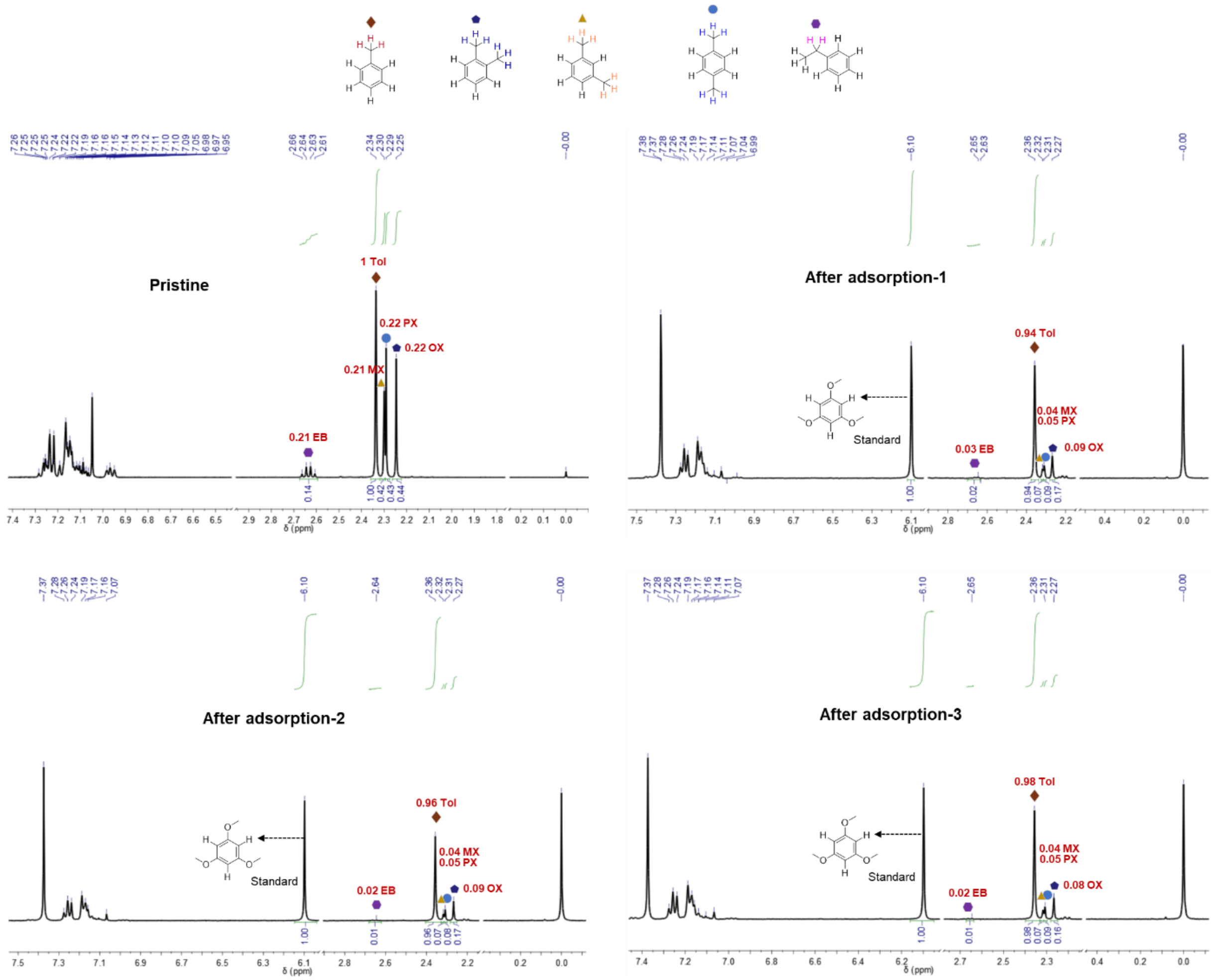

Figure S86. Relative uptake of Tol, OX, MX, EB and PX by **BN-DPTA1-A** after exposure to a quinary mixture containing Tol/xylene isomers in 1:1 molar ratio (xylene isomers were comprised of equimolar amount of OX, MX, EB and PX) for 300 min, as determined by ^{1}H NMR spectra (298 K, $CDCl_3$, 400 MHz).

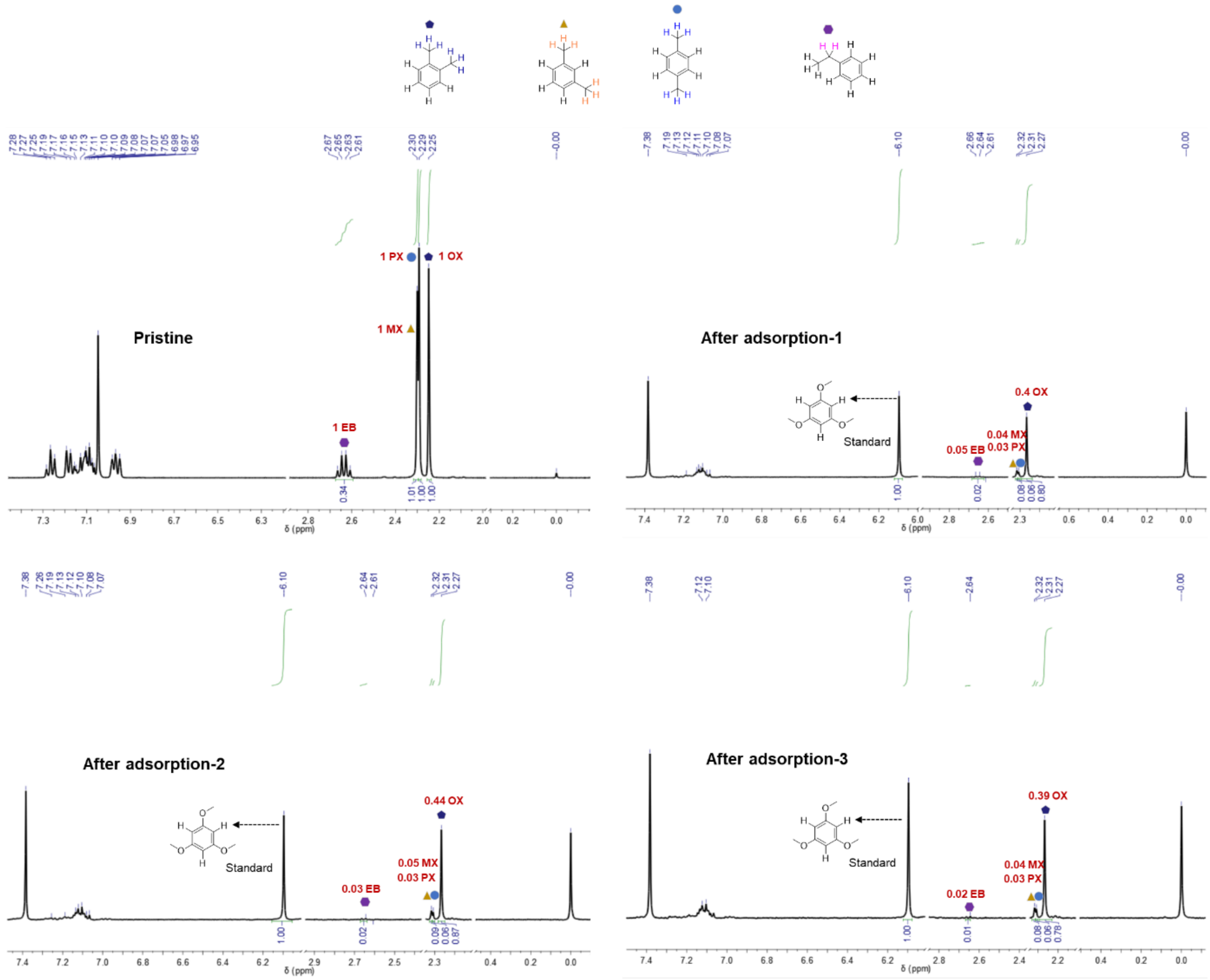

Figure S87. Relative uptake of OX, MX, EB, and PX by **BN-DPTA2-A** after exposure to an equimolar quaternary mixture for 300 min, as determined by ^{1}H NMR spectra (298 K, $CDCl_3$, 400 MHz).

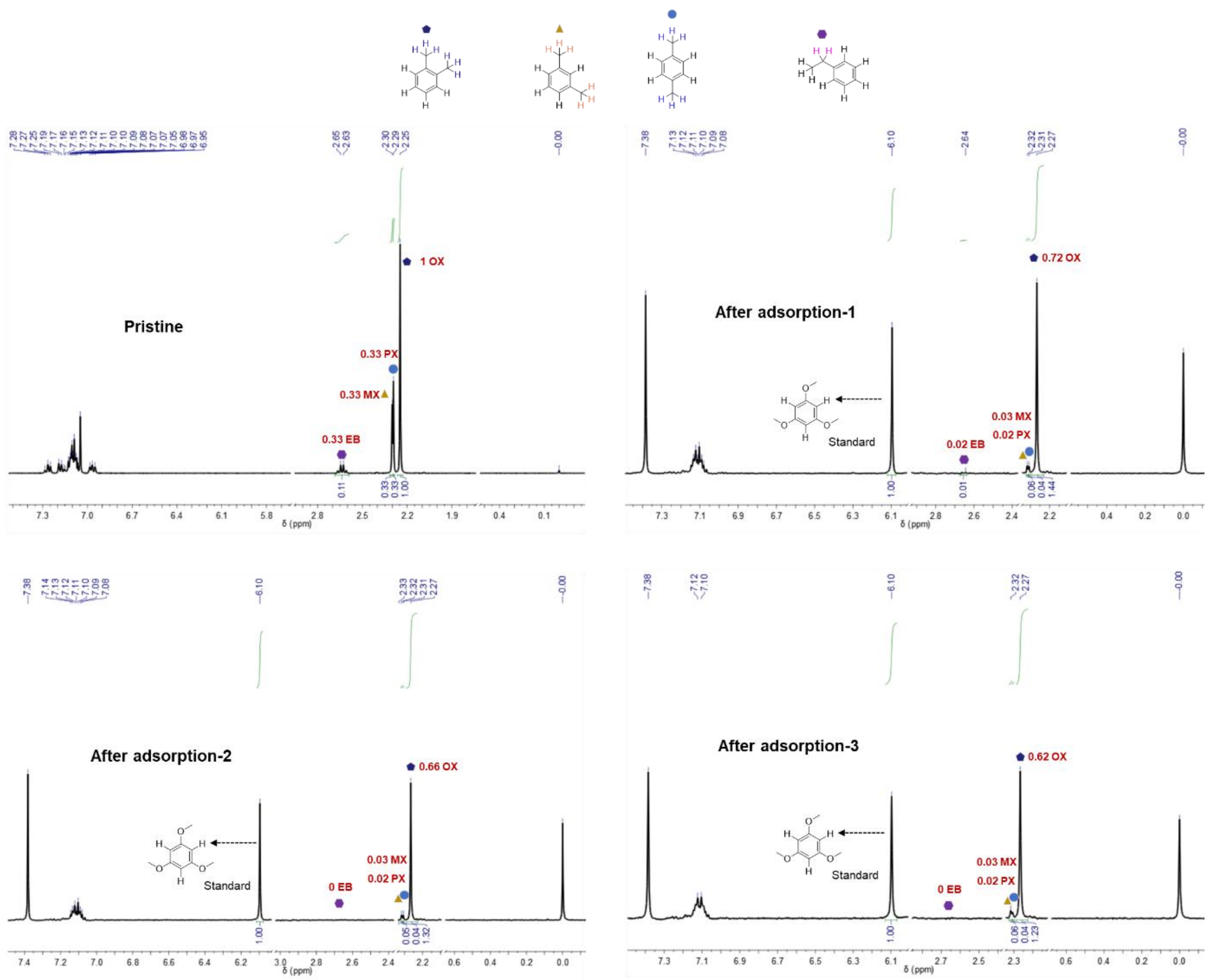

Figure S88. Relative uptake of OX, MX, EB, and PX by **BN-DPTA2-A** after exposure to a quaternary mixture containing OX/xylene isomers in 1:1 molar ratio (xylene isomers were comprised of equimolar amount of MX, EB and PX) for 300 min, as determined by ^{1}H NMR spectra (298 K, $CDCl_3$, 400 MHz).

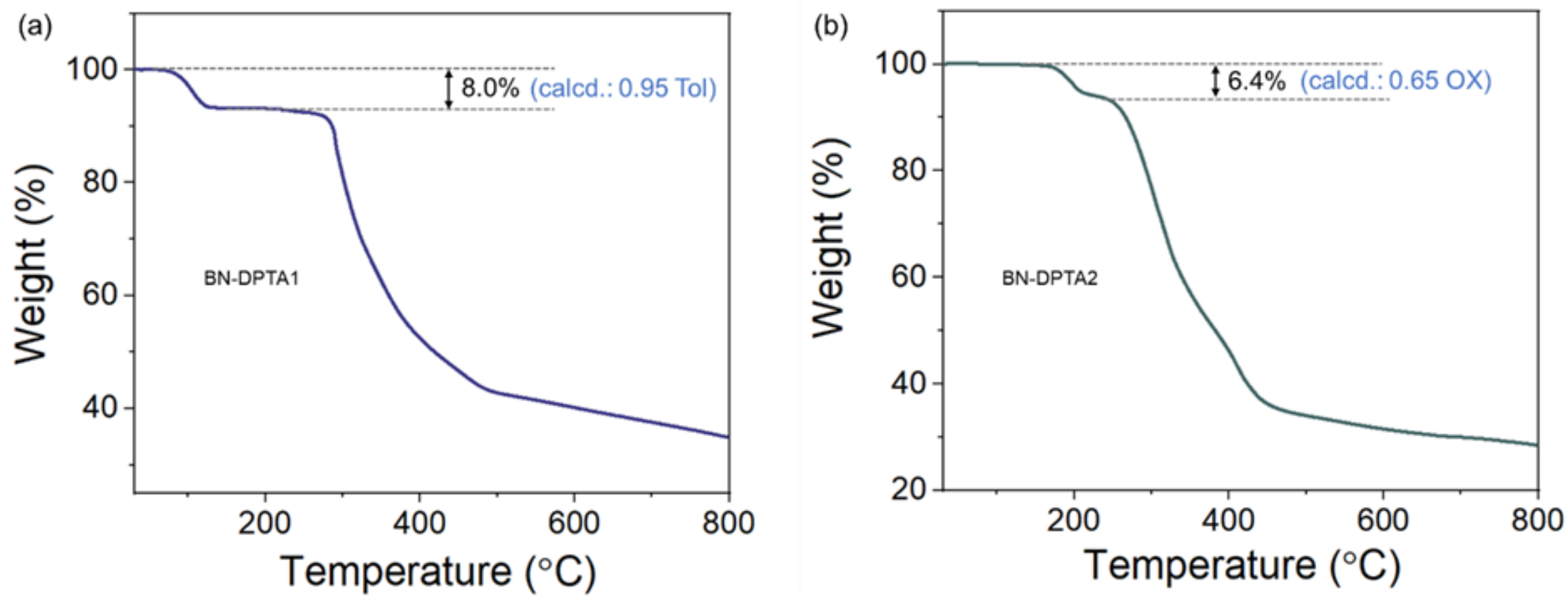

Figure S89. TGA curves of **BN-DPTA1-A** and **BN-DPTA2-A** after selective adsorption from (a) a 1:1 molar ratio mixture of Tol and C8 aromatic isomers (comprised of equimolar OX, MX, EB, and PX), and (b) a 1:1 molar ratio mixture of OX and other C8 aromatic isomers (comprised of equimolar MX, EB, and PX), respectively.

Table S6. Comparison in xylene adsorption among metal-free crystalline frameworks

Material	Preference	Mixture complexity (no. of components)	Selectivity	Conditions (Temperature, Pressure, phase)	Required time to reach saturated adsorption capacity	Ref.
**BN-DPTA2-A**	**OX**	**Quaternary mixture**	**$S_{OX/PX}$ = 13.7±0.8**<br>**$S_{OX/MX}$ = 9.5 ± 0.7**<br>**$S_{OX/EB}$ = 12.4±4.5**	**373 K, 101.3 kPa, liquid**	**~180 min**	**This work**
COF-AA-Me	OX	Ternary mixture	$S_{OX/PX}$ = 1.66	298 K, -, gas	Not mentioned	8
HOF-35a	OX	Ternary mixture	$S_{OX/MX/PX}$ = 4.4/1.5/1.0	298 K, 101.3 kPa, liquid	12h	9
HOF-PX-a	PX	Quaternary mixture	$S_{PX/OX}$ = 3.2<br>$S_{PX/MX}$ = 2.9	298 K, 101.3 kPa, liquid	8h	10

			$S_{PX/EB}$ = 4.9			
TPBD-αI	PX	Quaternary mixture	$S_{PX/OX/MX/EB}$ = 48.5	298 K, 101.3 kPa, liquid	4d	2
EtP6β	PX	Quaternary mixture	Not mentioned	298 K, 101.3 kPa, gas	20h	11
Bis-urea macrocycle	PX	Quaternary mixture	$S_{PX/EB/MX/OX}$ = 10.87/5.67/1.49/1	453 K, -, gas	overnight	12
EtFLP6	PX	Ternary mixture	Not mentioned	298 K, 101.3 kPa, gas	~60 min	13
COF-AB-Me	MX	Ternary mixture	$S_{MX/PX}$ = 1.05	298 K, -, gas	Not mentioned	8
POP-1	MX	Binary mixture	$S_{MX/PX}$ = 2.04<br>$S_{MX/OX}$ = 2.08	298 K, -, gas	Not mentioned	14

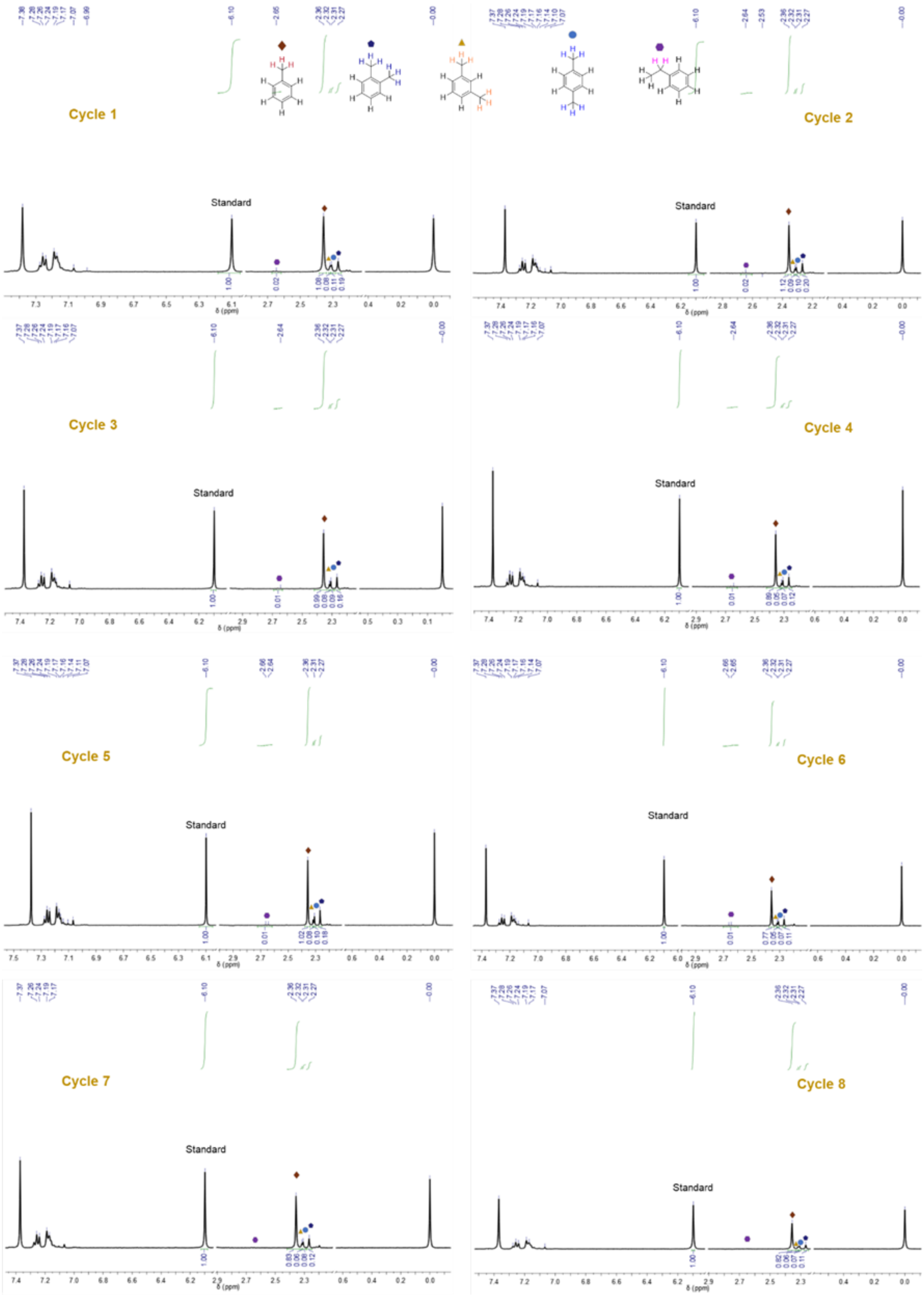
Cycle 1
Standard
Cycle 2
Standard
Cycle 3
Standard
Cycle 4
Standard
Cycle 5
Standard
Cycle 6
Standard
Cycle 7
Standard
Cycle 8
Standard
δ (ppm)

Figure S90. Relative uptake of Tol, OX, MX, EB and PX by **BN-DPTA1-A** after exposure to a quinary mixture containing Tol/C8 aromatics in 1:1 molar ratio (C8 aromatics were comprised of equimolar amount of OX, MX, EB and PX) for 8 cycles, as determined by ^1H NMR spectra (298 K, $CDCl_3$, 400 MHz).

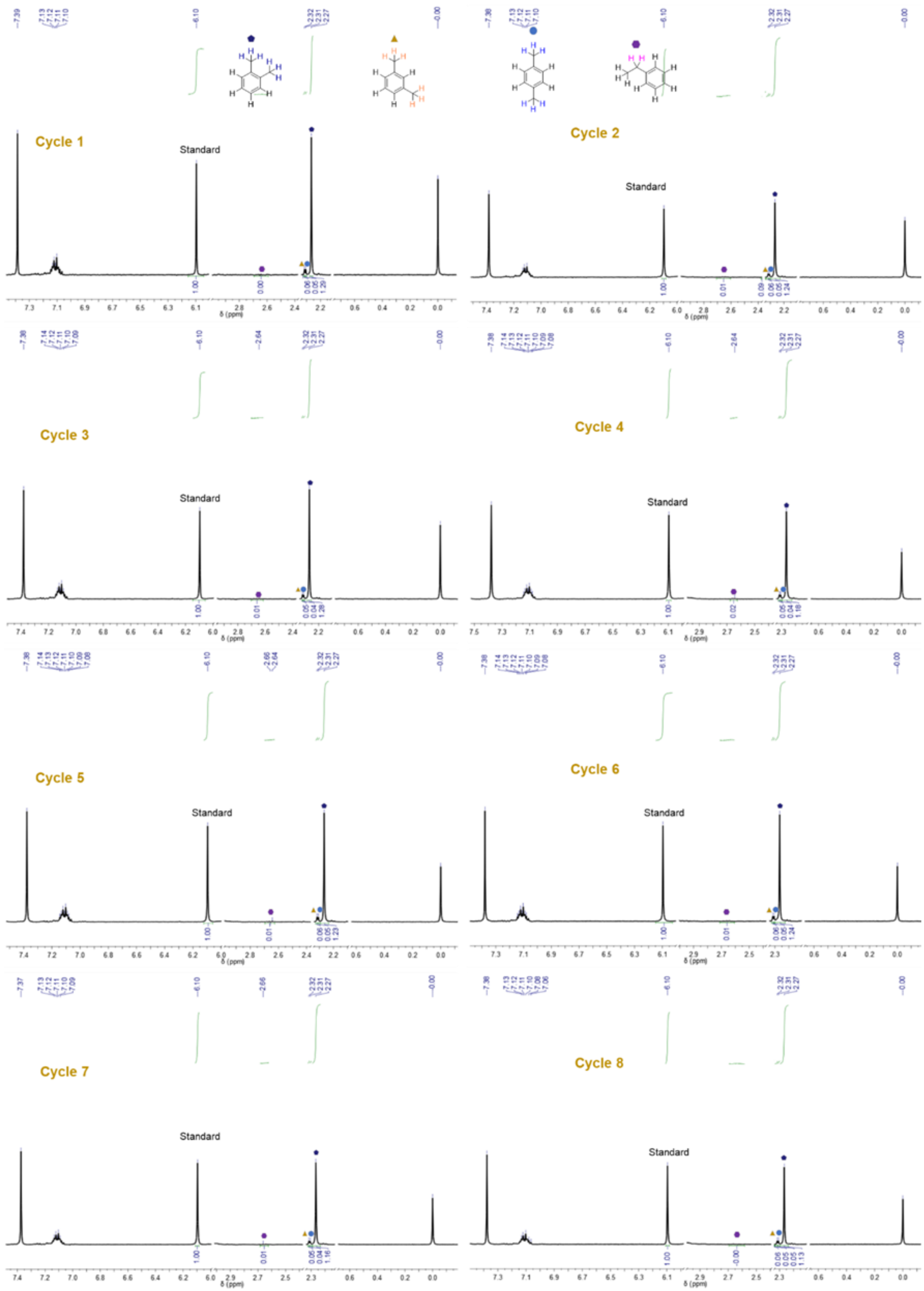
Cycle 1
Standard
Cycle 2
Standard
Cycle 3
Standard
Cycle 4
Standard
Cycle 5
Standard
Cycle 6
Standard
Cycle 7
Standard
Cycle 8
Standard

Figure S91. Relative uptake of OX, MX, EB, and PX by **BN-DPTA2-A** after exposure to a quaternary mixture containing OX/other C8 aromatics in 1:1 molar ratio (other C8 aromatics were comprised of equimolar amount of MX, EB and PX) for 8 cycles, as determined by ^{1}H NMR spectra (298 K, $CDCl_3$, 400 MHz).

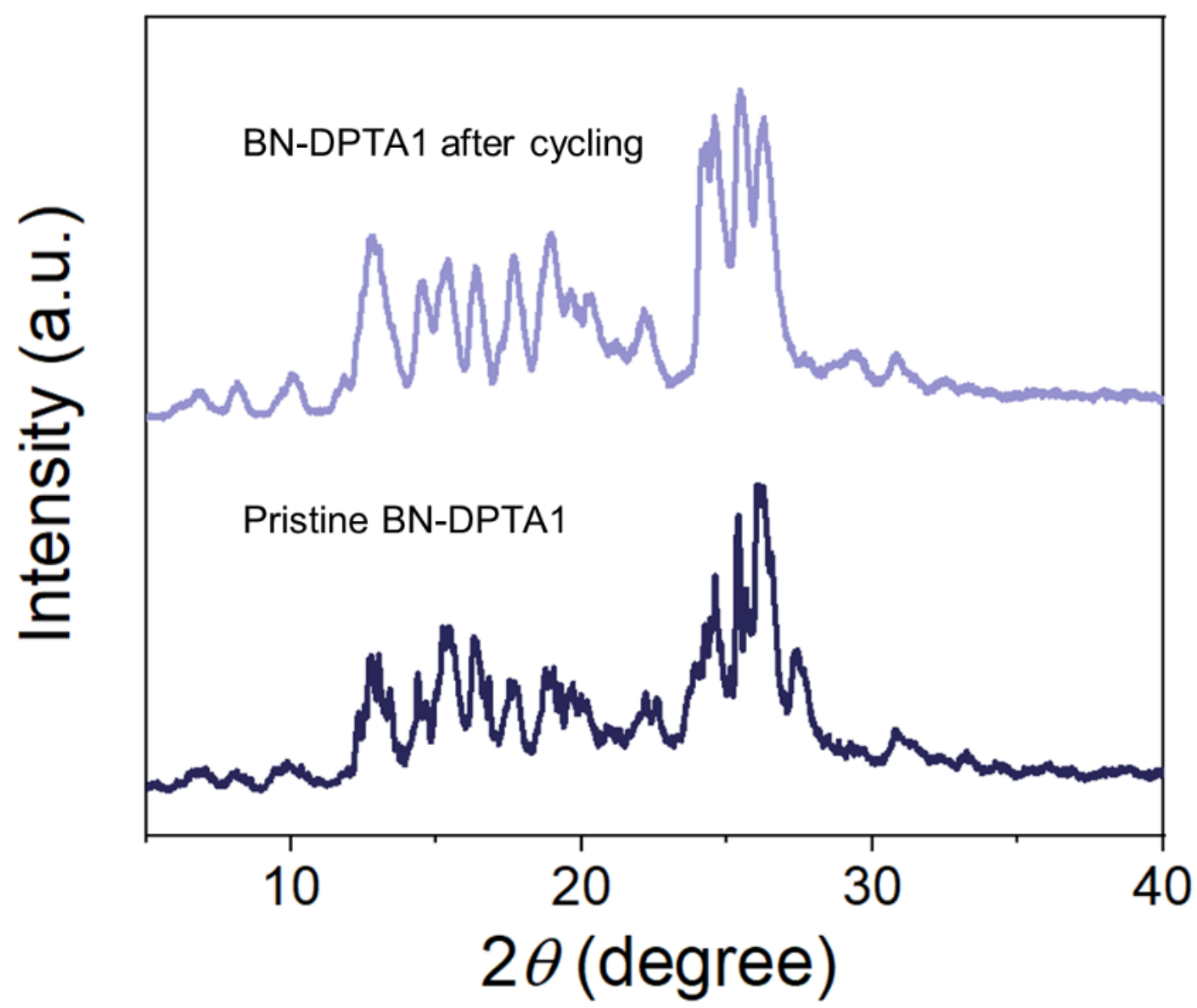

Figure S92. PXRD patterns of **BN-DPTA1** after 8 adsorption/desorption cycles.

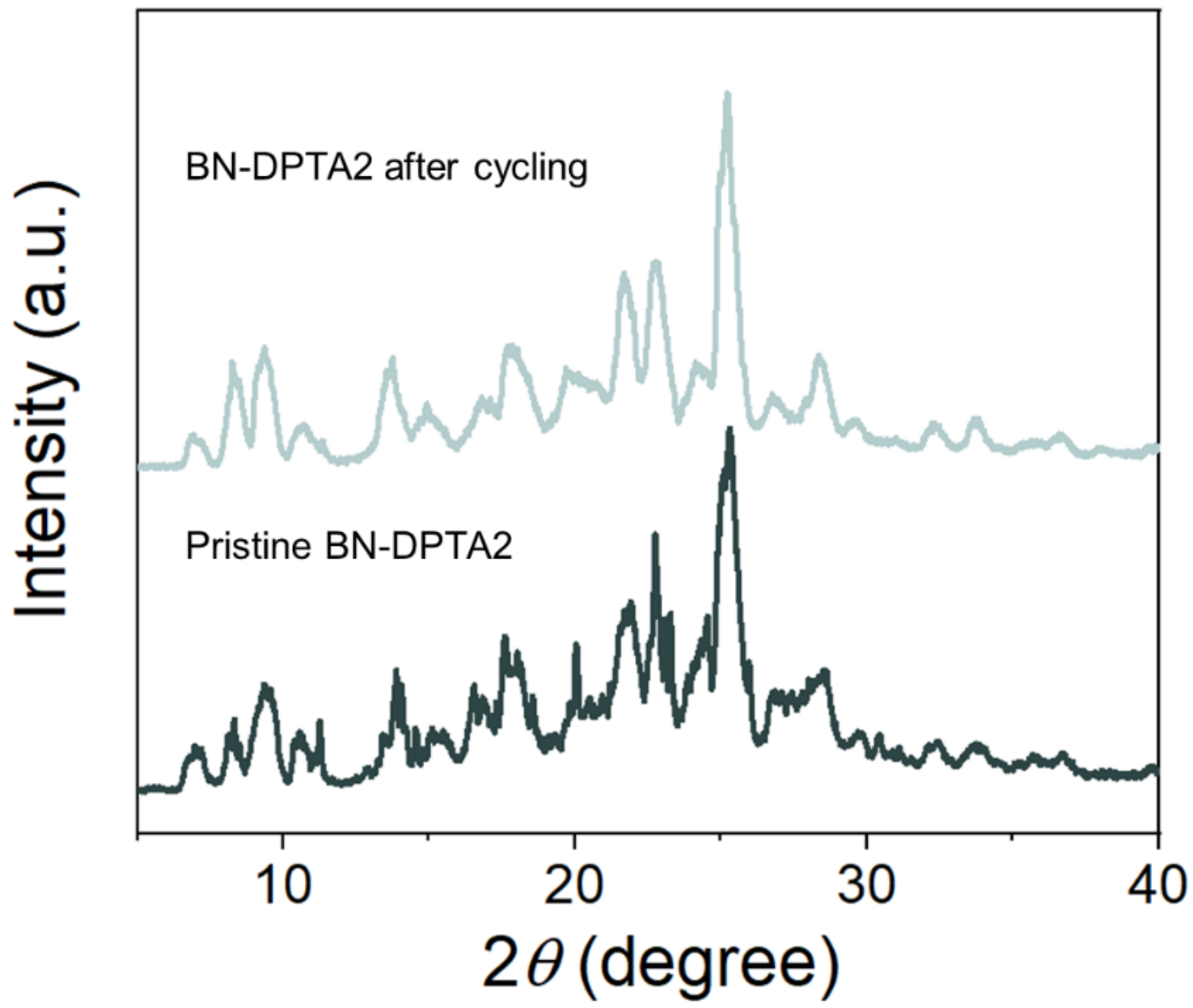

Figure S93. PXRD patterns of **BN-DPTA2** after 8 adsorption/desorption cycles.

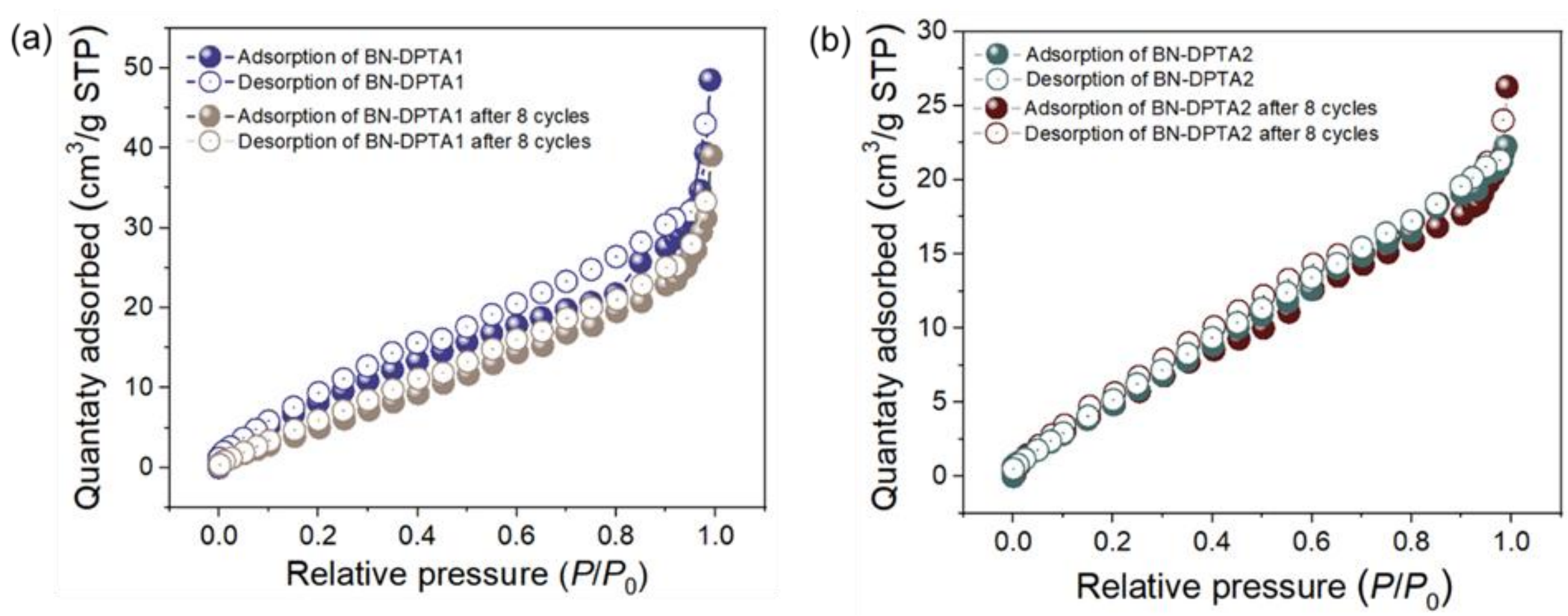

Figure S94. N_2 adsorption-desorption isotherms of (a) **BN-DPTA1** and (b) **BN-DPTA2** before and after cycling, measured at 77 K.

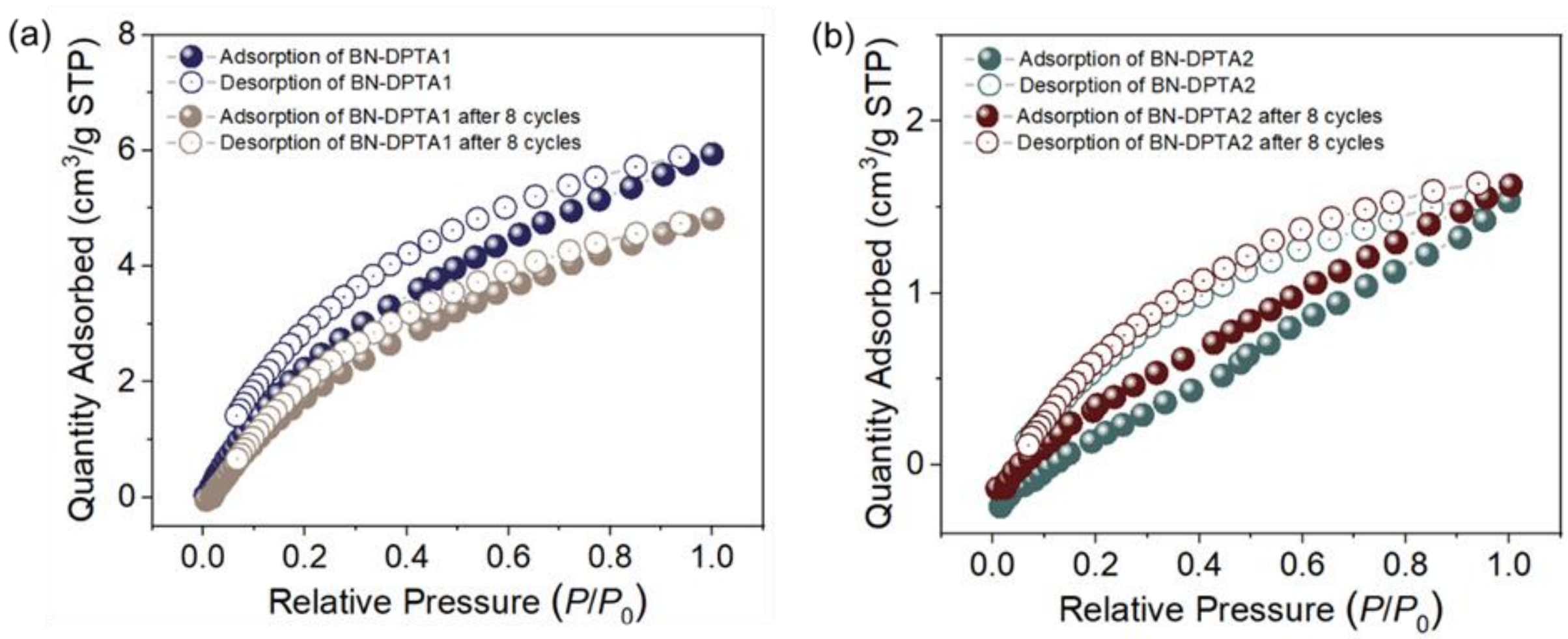

Figure S95. CO_2 adsorption-desorption isotherms of (a) **BN-DPTA1** and (b) **BN-DPTA2** before and after cycling, measured at 273 K.

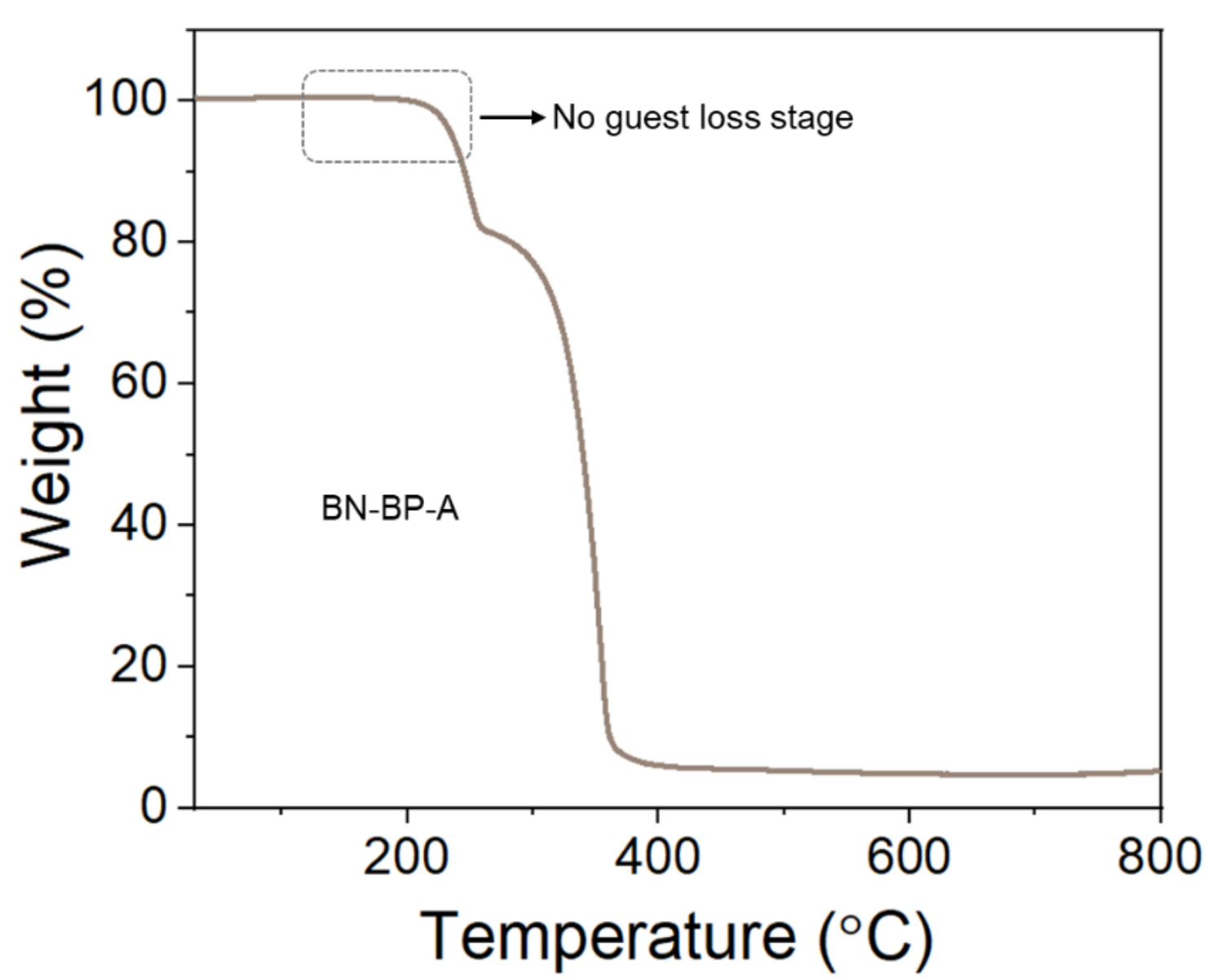

Figure S96. TGA curve of **BN-BP-A**.

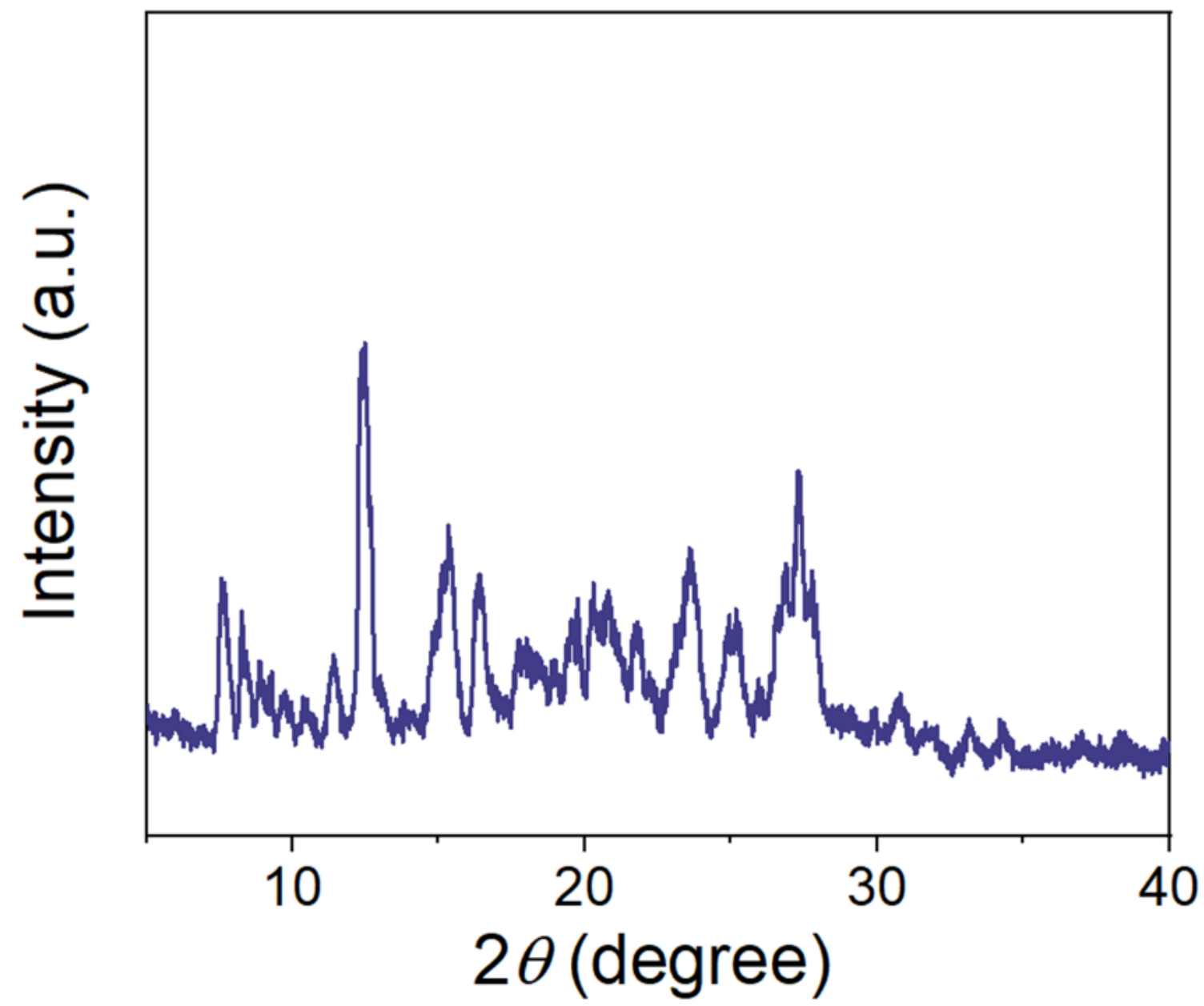

Figure S97. PXRD pattern of **BN-BP-A**.

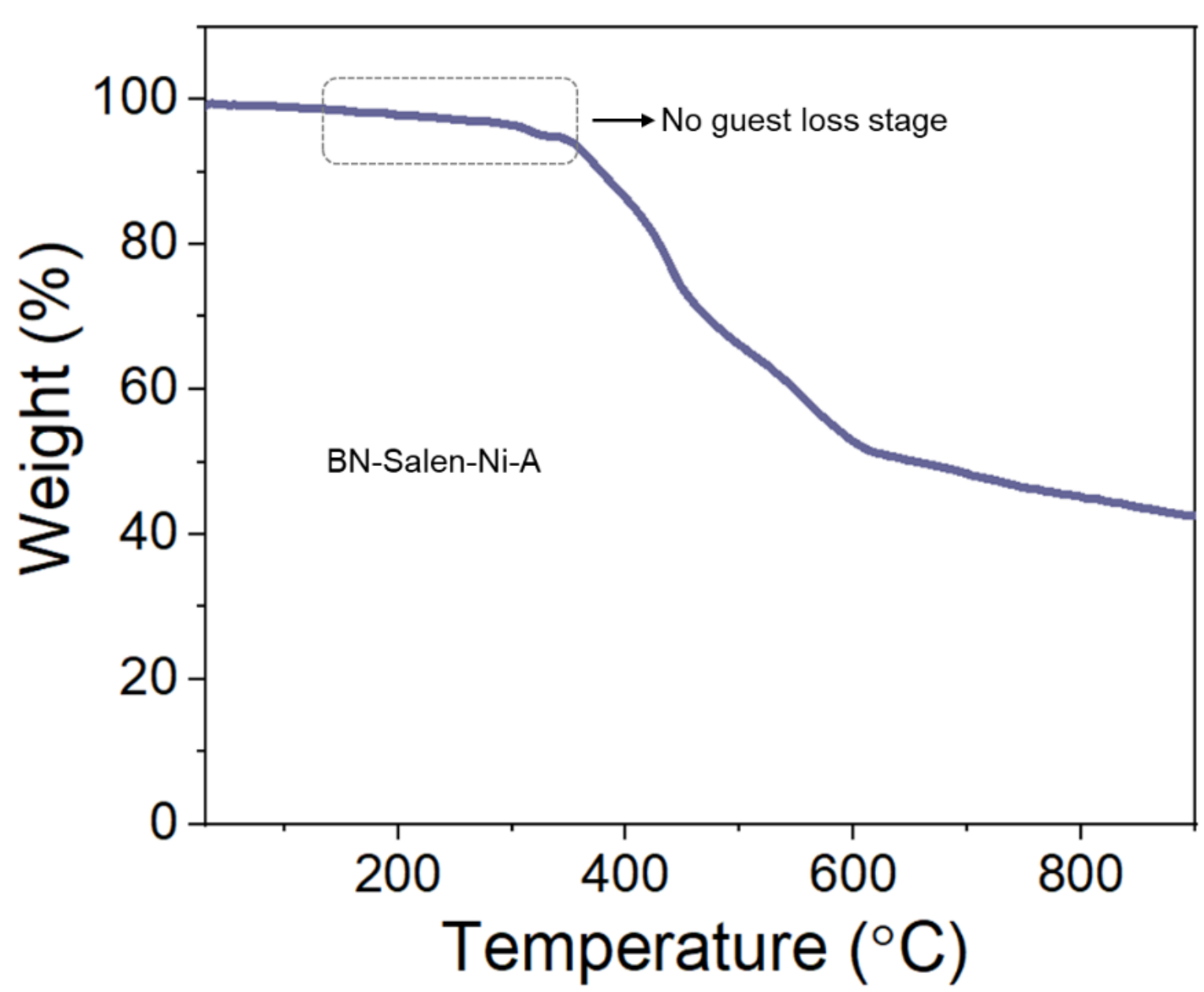

Figure S98. TGA curve of **BN-Salen-Ni-A**.

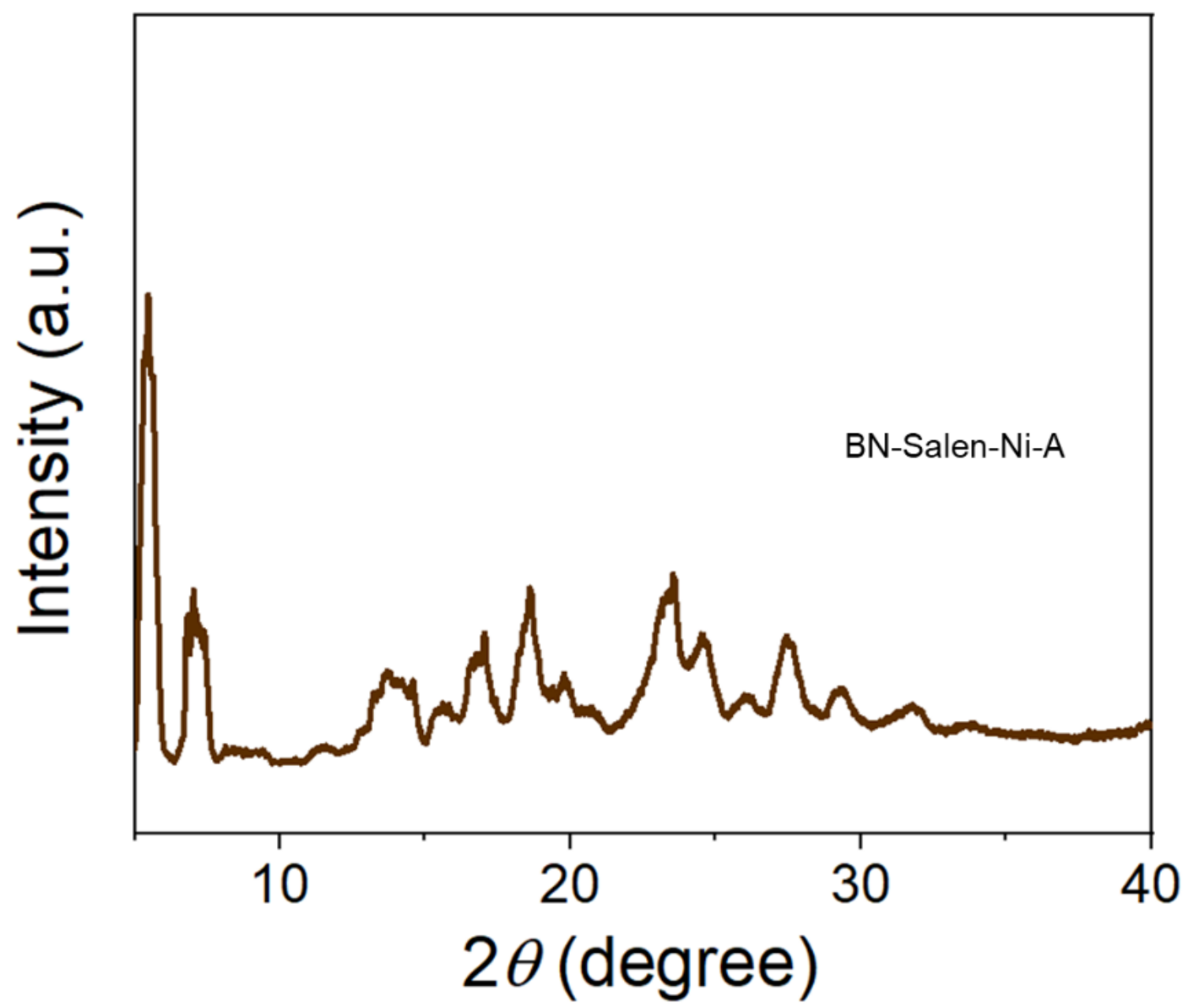

Figure S99. PXRD pattern of **BN-Salen-Ni-A**.

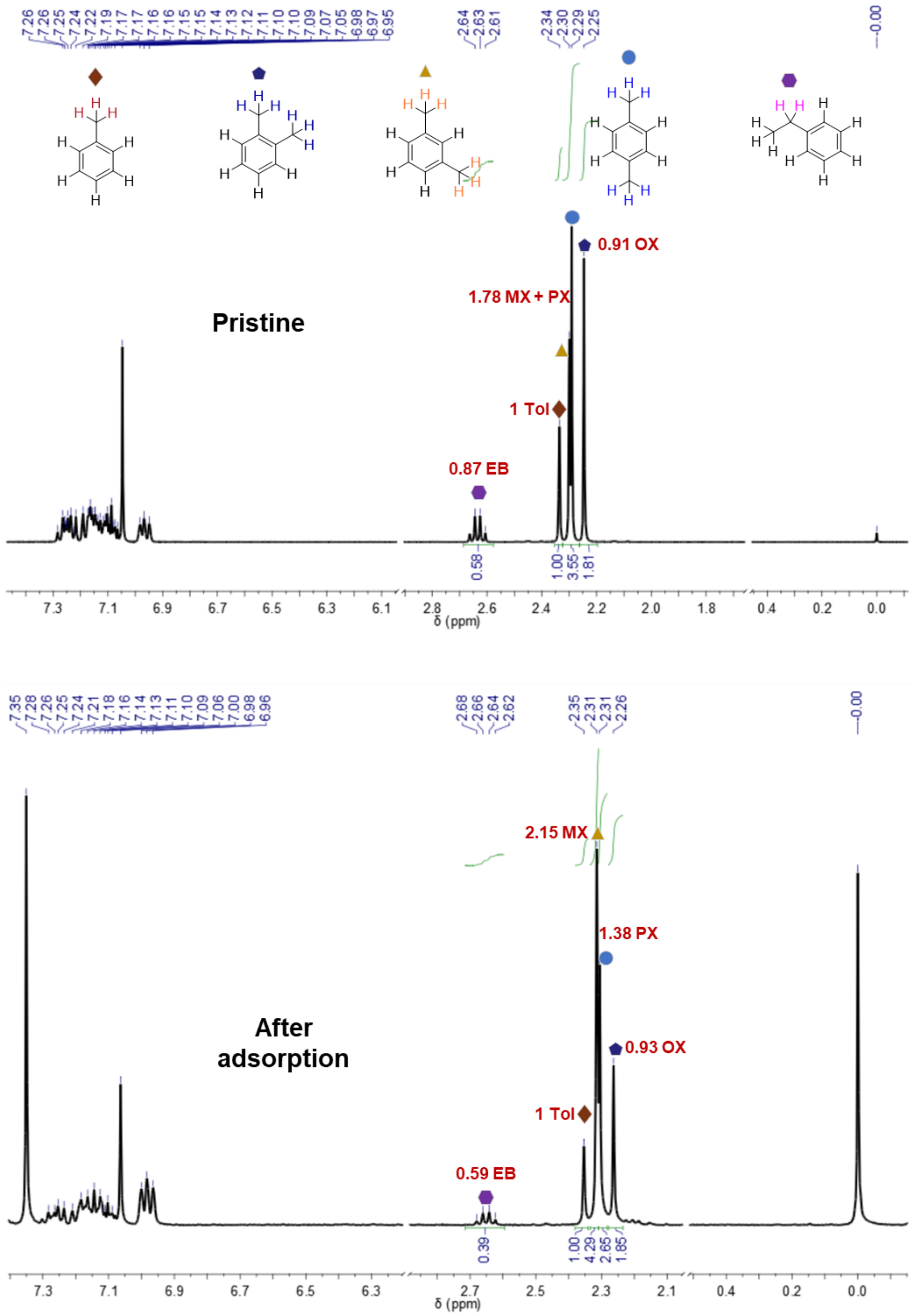

Figure S100. Relative uptake of Tol, OX, MX, EB, and PX by **BN-BP** after exposure to an equimolar quinary mixture for 300 min, as determined by ^{1}H NMR spectra (298 K, $CDCl_3$, 400 MHz).

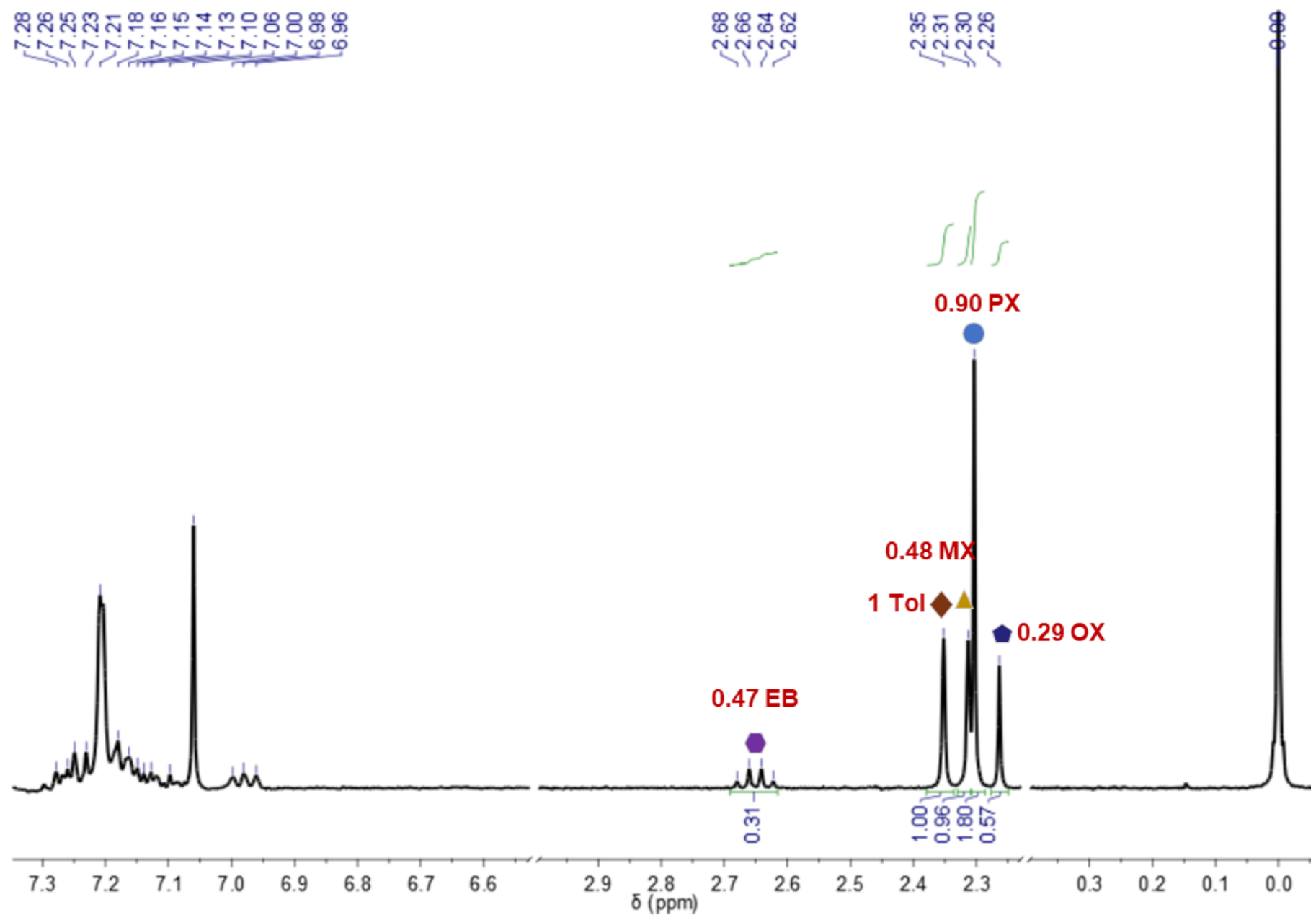

Figure S101. Relative uptake of Tol, OX, MX, EB, and PX by **BN-Salen-Ni** after exposure to an equimolar quinary mixture for 300 min, as determined by ^{1}H NMR spectra (298 K, $CDCl_3$, 400 MHz).

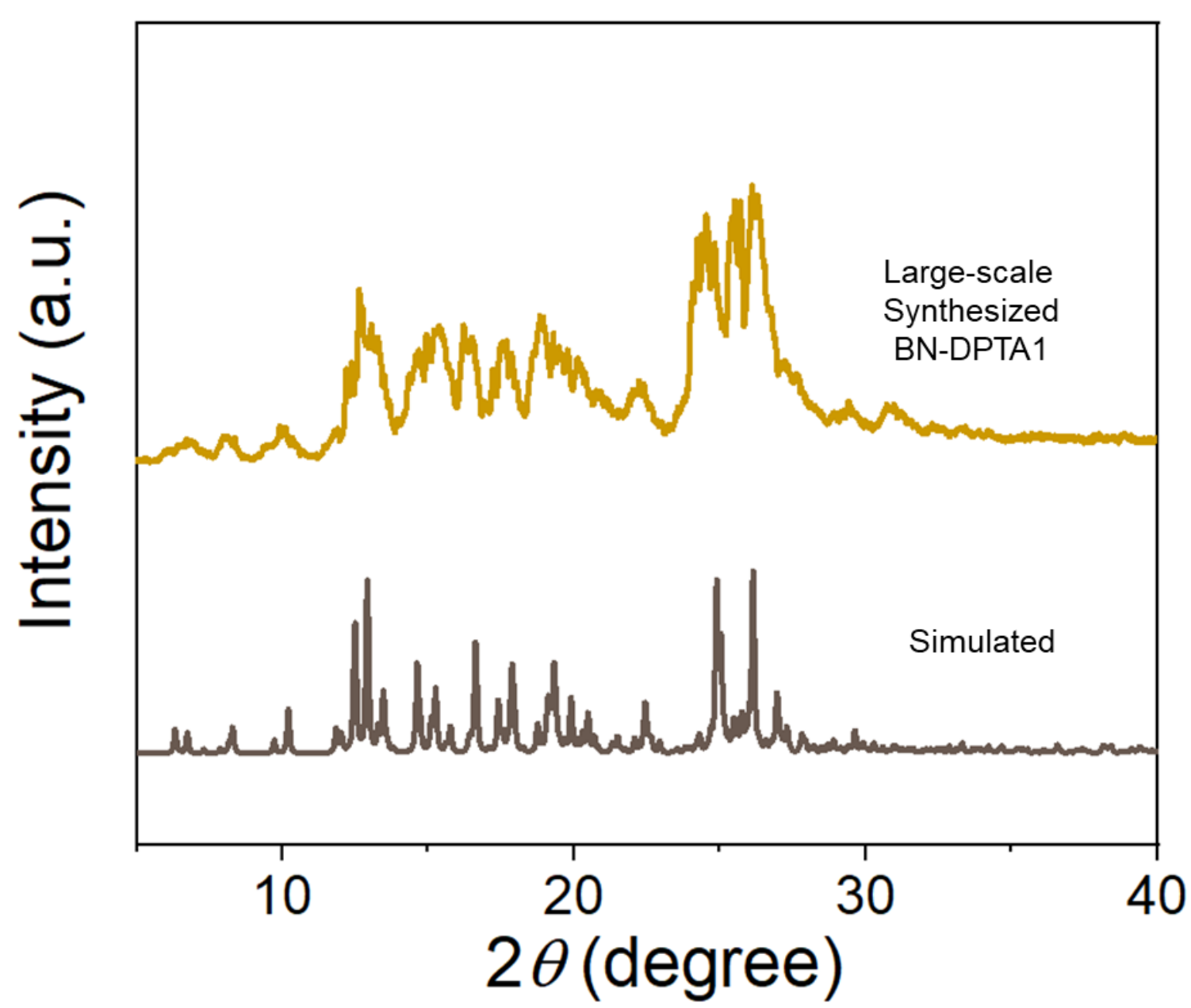

Figure S102. Experimental PXRD pattern of large-scale synthesized **BN-DPTA1** compared with the simulated pattern derived from the SC-XRD data.

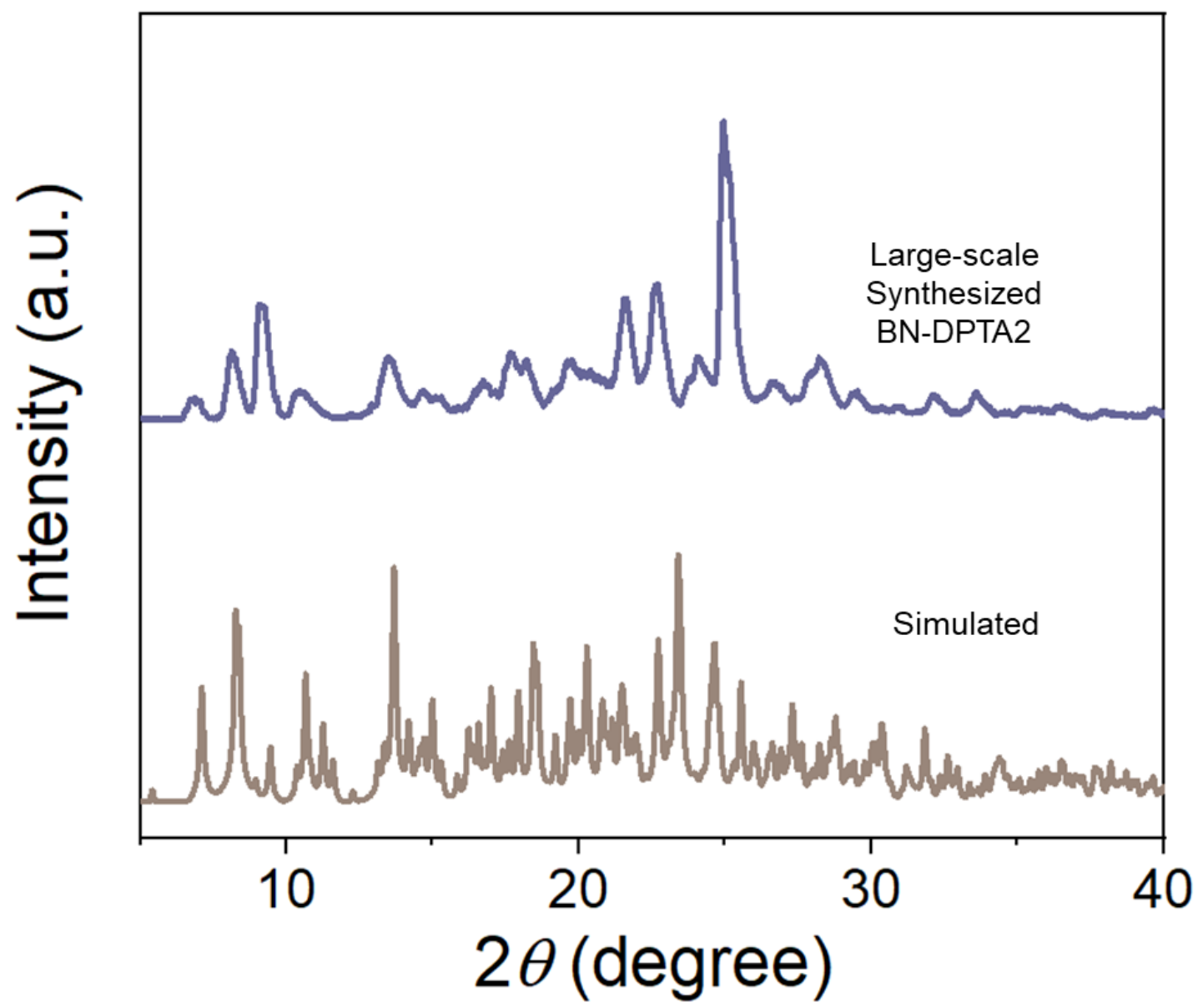

Figure S103. Experimental PXRD pattern of large-scale synthesized **BN-DPTA2** compared with the simulated pattern derived from the SC-XRD data.

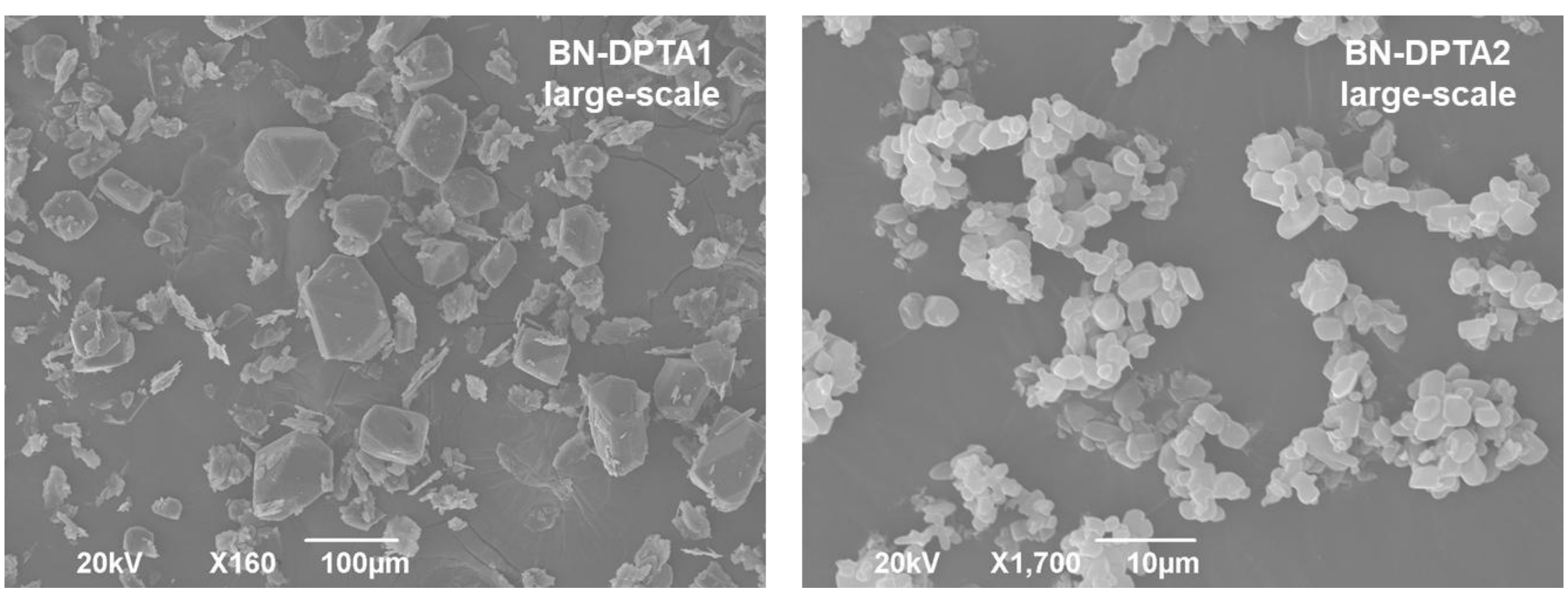

Figure S104. SEM images of large-scale synthesized **BN-DPTA1** and **BN-DPTA2**.

Table S7. Crystal data and structure refinement details for **BN-DPTA1** after soaking in oil for 1 week.

Identification code	**BN-DPTA1-after soaking**
Formula	$C_{61}H_{42}B_6N_{12}O_6$
Mr.	1106.92
Temperature (K)	173
Cryst. Syst.	triclinic
Space group	*P*-1
a (Å)	14.9920(8)
b (Å)	15.2739(10)
c (Å)	15.5078(10)
α, β, γ (°)	73.8, 63.4, 71.6
Volume (Å^3)	2972.5(3)
Z	2
μ (mm^{-1})	0.081
$F_{(000)}$	1144.0
Radiation	MoKα (λ = 0.71073)
2θ (°)	3.558 to 50.742
Reflections collected	123634
Independent Refl.	10885 [R_{int} = 0.1007, R_{sigma} = 0.0449]
Data/restraints/parameters	10885/60/768
GOF on F^2	1.029
R_1, wR_2 [$I > 2\sigma(I)$]	R_1 = 0.0624, wR_2 = 0.1643

R_1, wR_2 (all data)	$R_1 = 0.1122$, $wR_2 = 0.1983$
CCDC number	2543040

Table S8. Crystal data and structure refinement details for **BN-DPTA2** after soaking in oil for 1 week.

Identification code	**BN-DPTA2-after soaking**
Formula	$C_{62}H_{44}B_6N_{12}O_6$
Mr.	1117.95
Temperature (K)	173
Cryst. Syst.	triclinic
Space group	*P*-1
a (Å)	13.3519(11)
b (Å)	13.8062(12)
c (Å)	17.9620(14)
α, β, γ (°)	108.4, 93.3, 104.5
Volume (Å^3)	3008.4(4)
Z	2
μ (mm^{-1})	0.080
$F_{(000)}$	1156.0
Radiation	MoKα (λ = 0.71073)
2θ (°)	4.194 to 49.998
Reflections collected	147382
Independent Refl.	10576 [R_{int} = 0.0500, R_{sigma} = 0.0198]
Data/restraints/parameters	10576/112/834

GOF on F^2	1.041
R_1, wR_2 [I >2σ(I)]	R_1 = 0.0504, wR_2 = 0.1399
R_1, wR_2 (all data)	R_1 = 0.0639, wR_2 = 0.1546
CCDC number	2543041

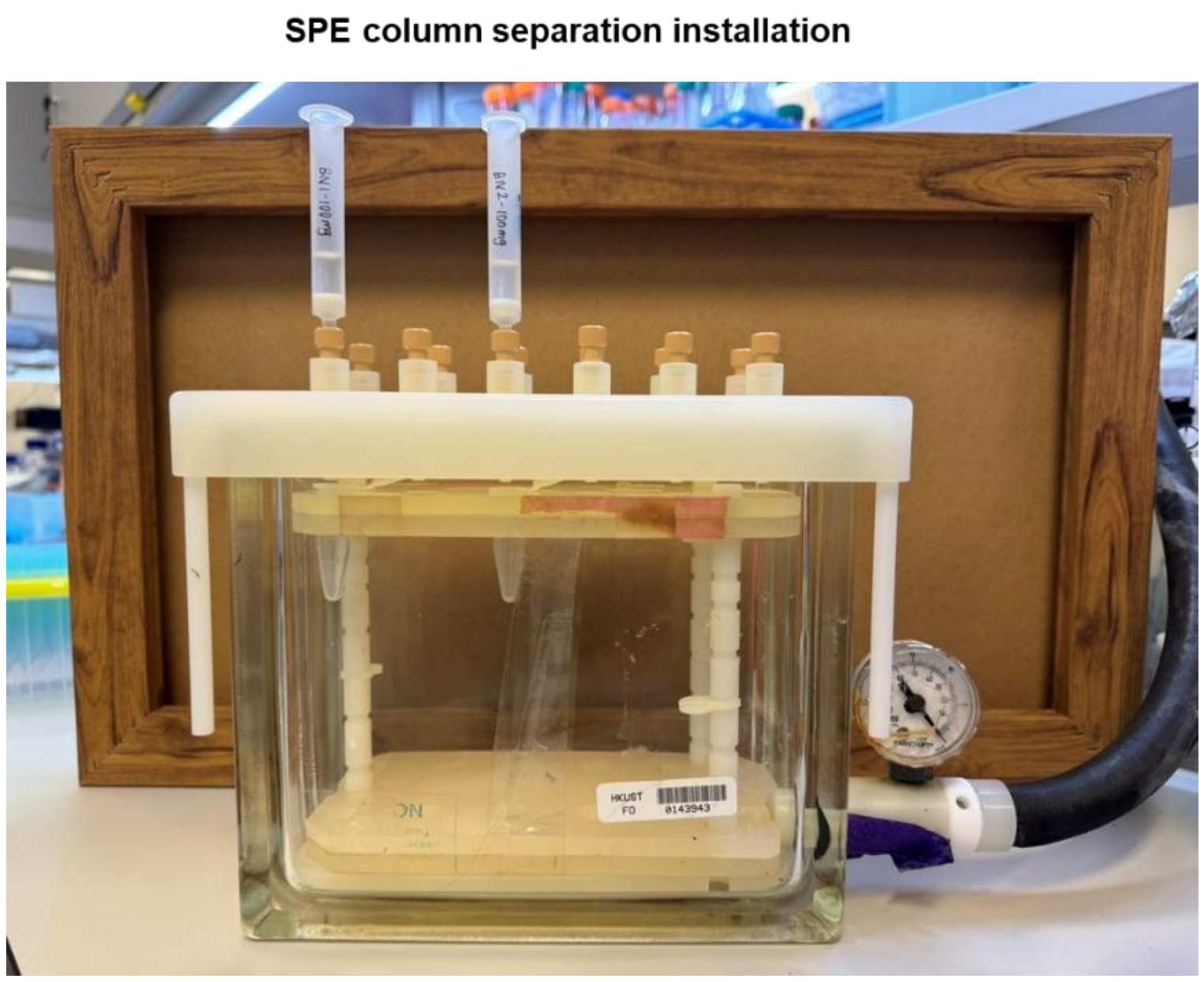

Figure S105. Digital photographs of the **BN-DPTA1-A** and **BN-DPTA2-A** based SPE columns and the separation and analysis setup.

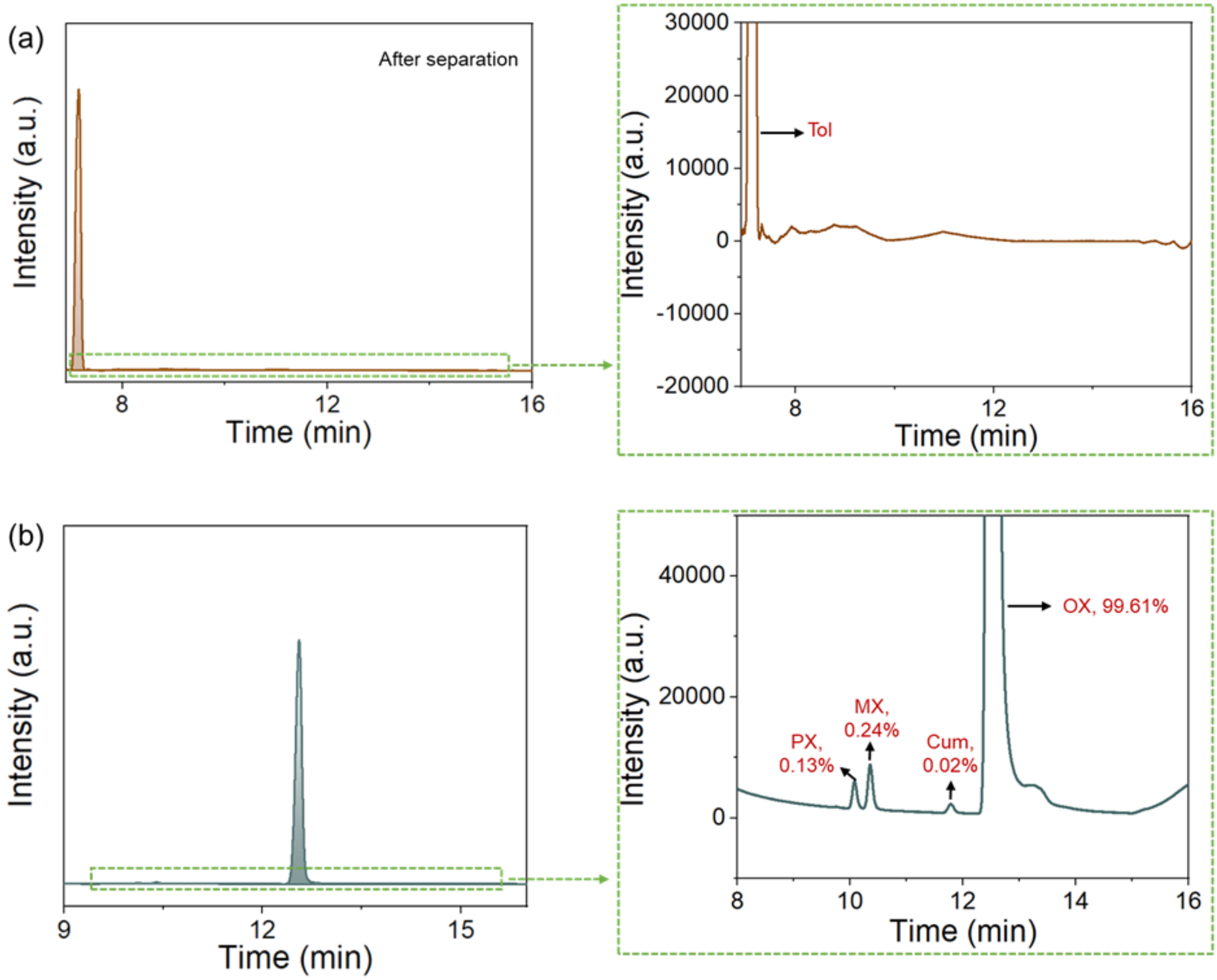

Figure S106. Amplified GC chromatograms showing the purified fractions of toluene and o-xylene after separation using (a) **BN-DPTA1**- and (b) **BN-DPTA2**-based SPE columns, respectively.

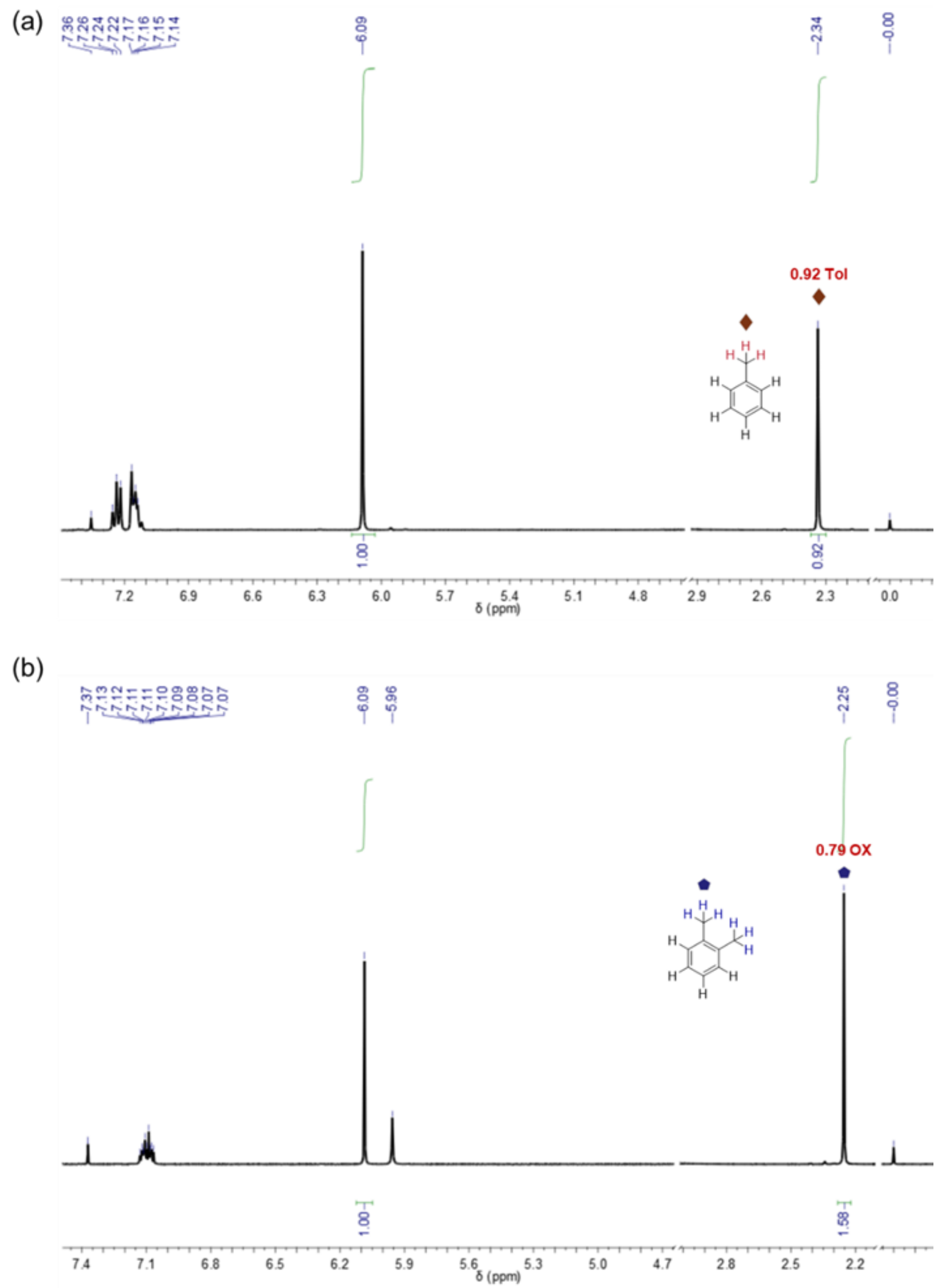

Figure S107. Adsorption capacities of **BN-DPTA1**- and **BN-DPTA2**-based SPE columns toward Tol and OX, respectively, determined by ^{1}H NMR spectra ($CDCl_3$, 400 MHz, 298K).

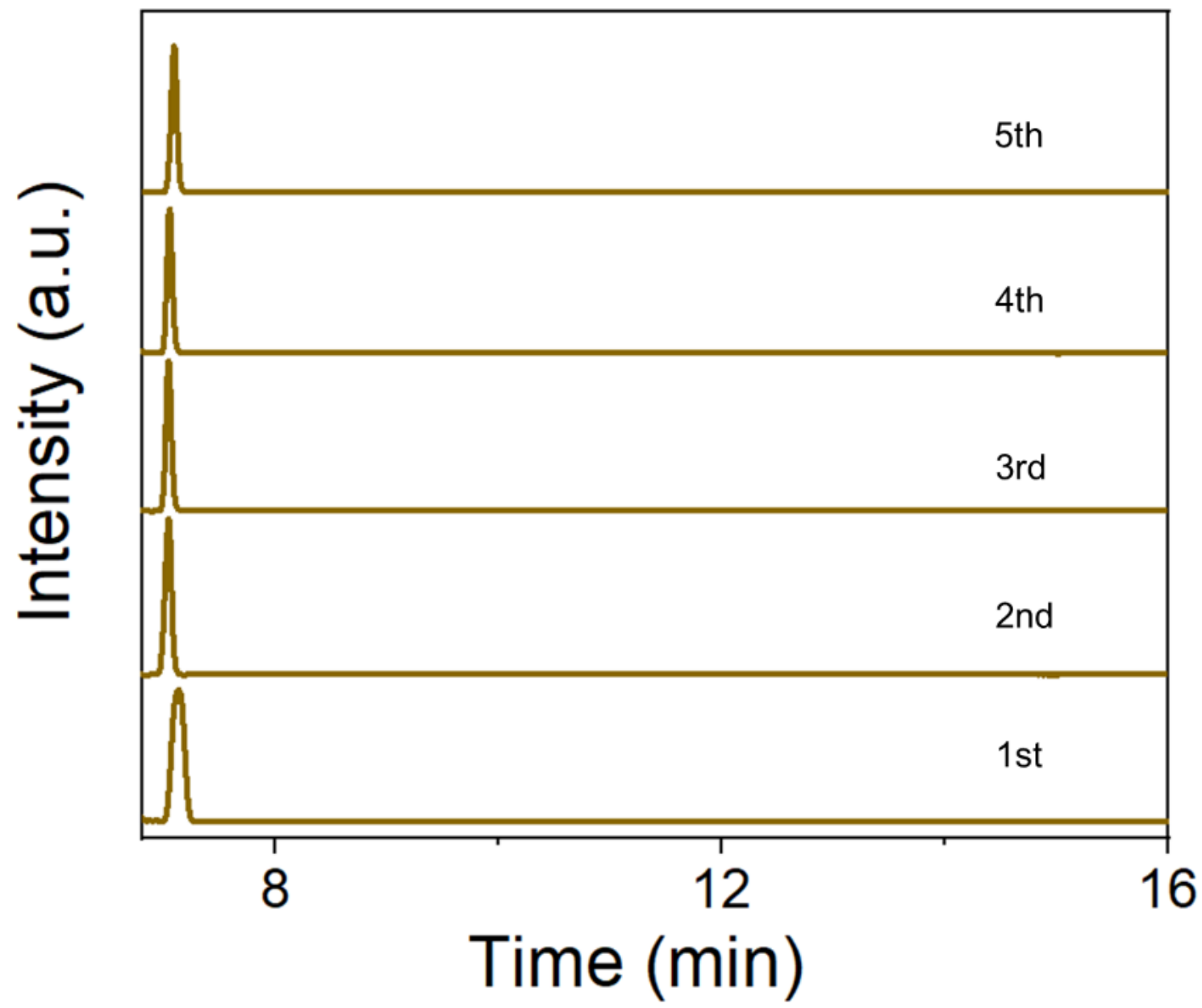

Figure S108. GC-FID analyses of separated analytes by **BN-DPTA1-A** based SPE column for 5 cycles.

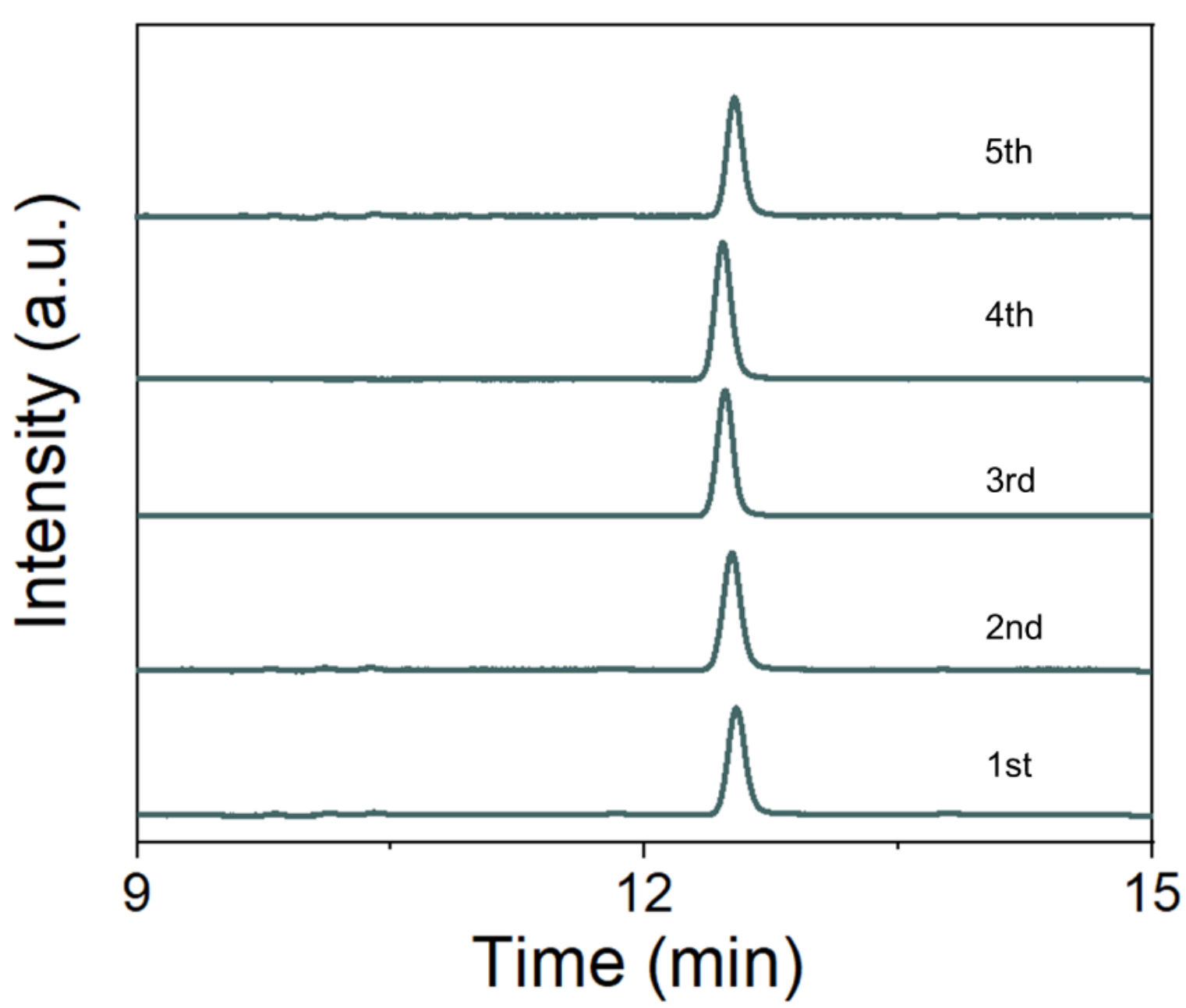

Figure S109. GC-FID analyses of separated analytes by **BN-DPTA2-A** based SPE column

for 5 cycles.

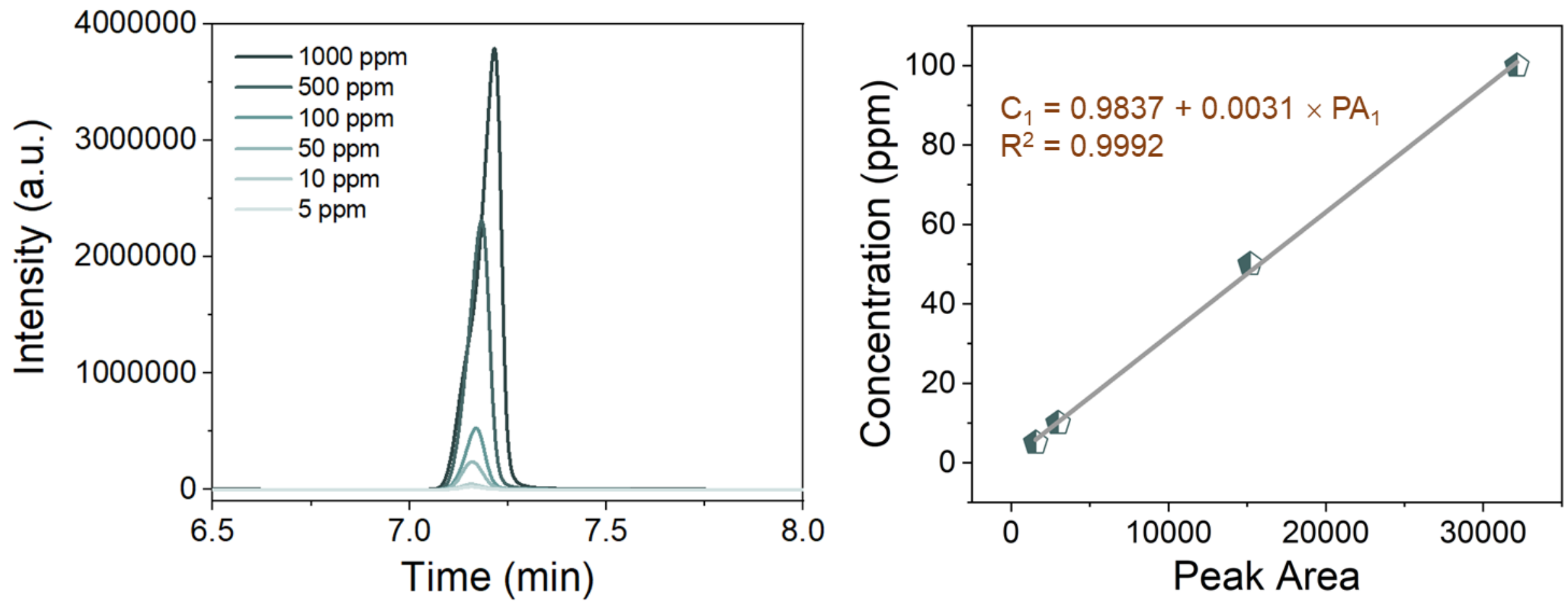

Figure S110. GC-FID analysis of Tol at different concentrations, and corresponding standard curve.

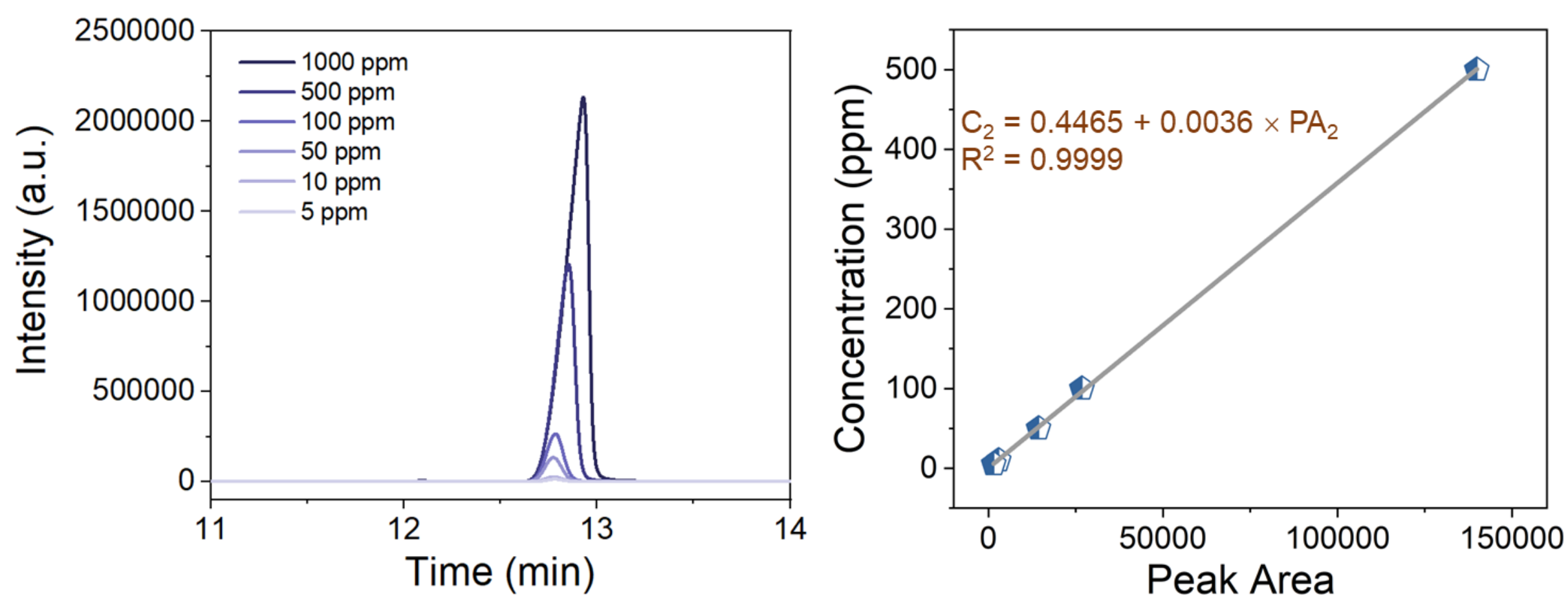

Figure S111. GC-FID analysis of OX at different concentrations, and corresponding standard curve.

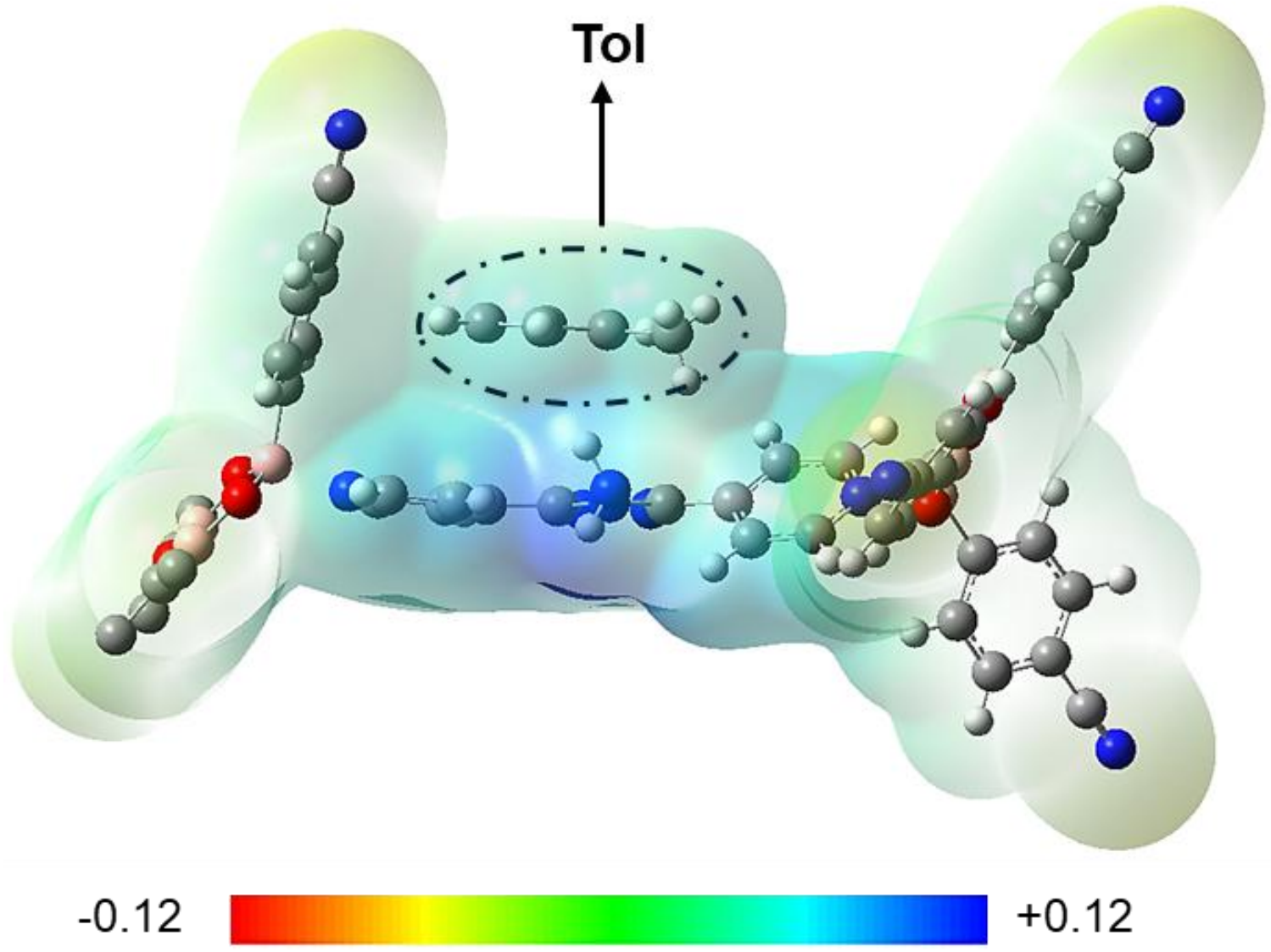

Figure S112. ESP map of the adduct between B←N unit in **BN-DPTA1** and Tol.

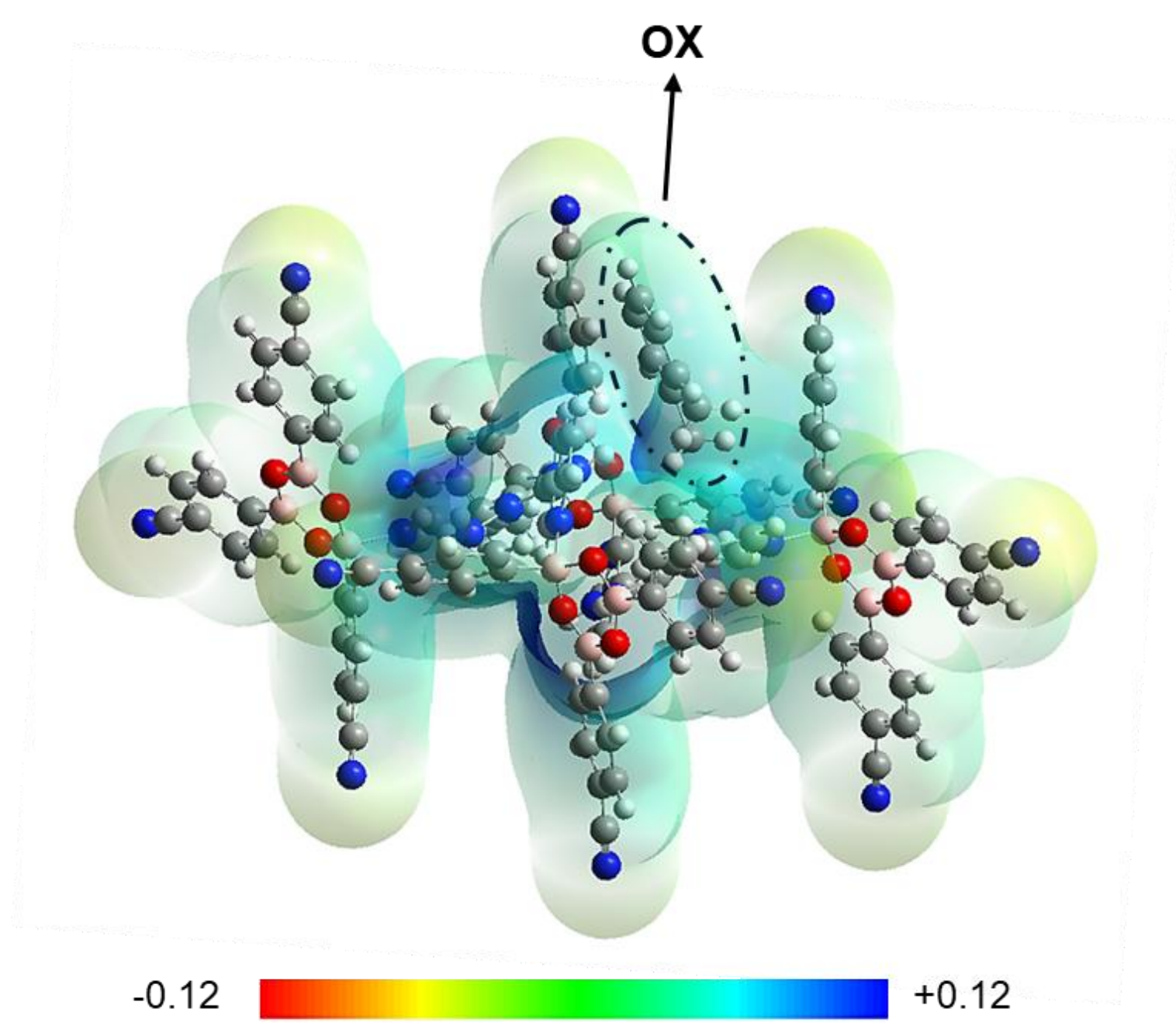

Figure S113. ESP map of the adduct between B←N unit in **BN-DPTA2** and OX.

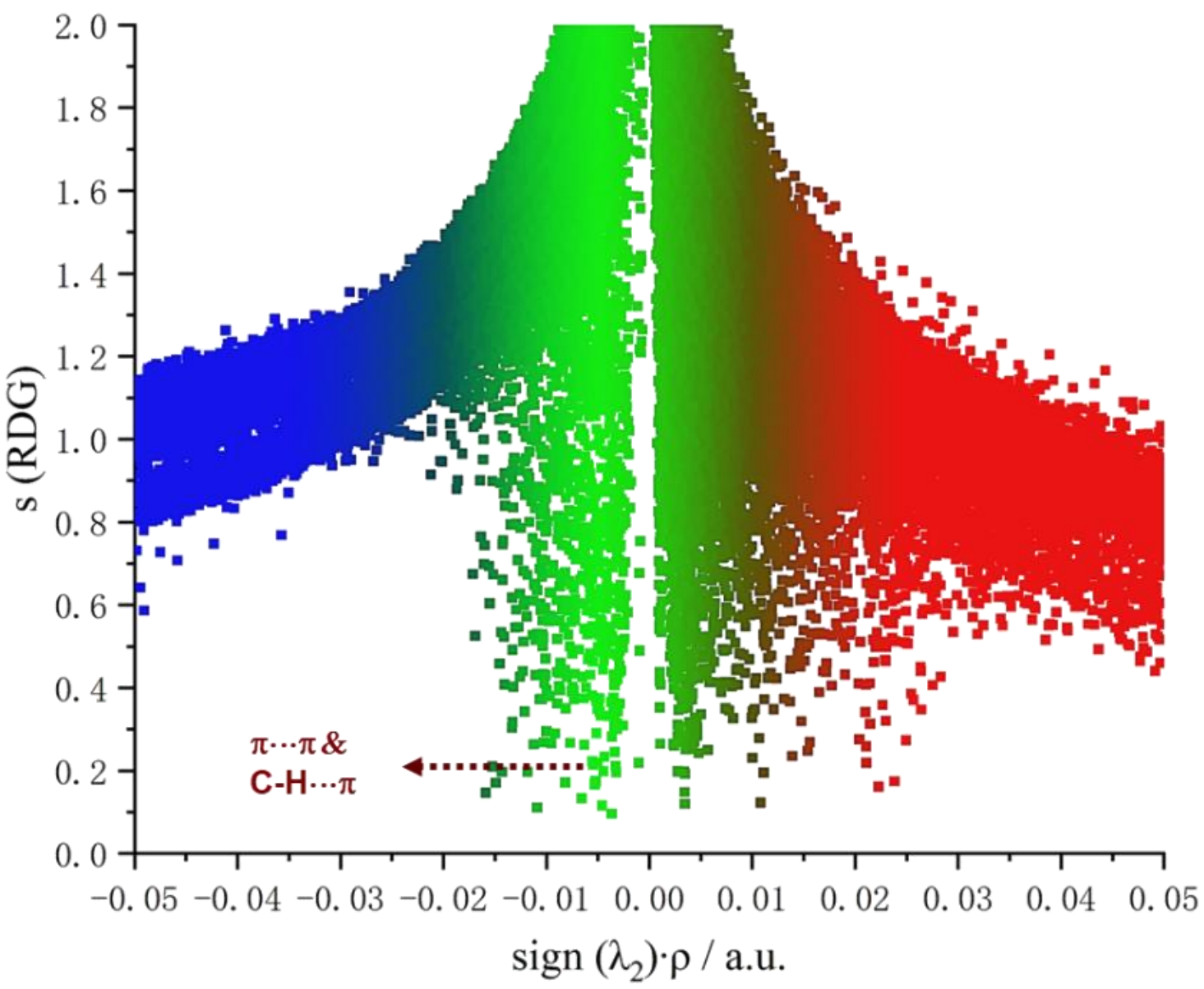

Figure S114. RDG analysis of the adduct between B←N unit in **BN-DPTA1** and Tol.

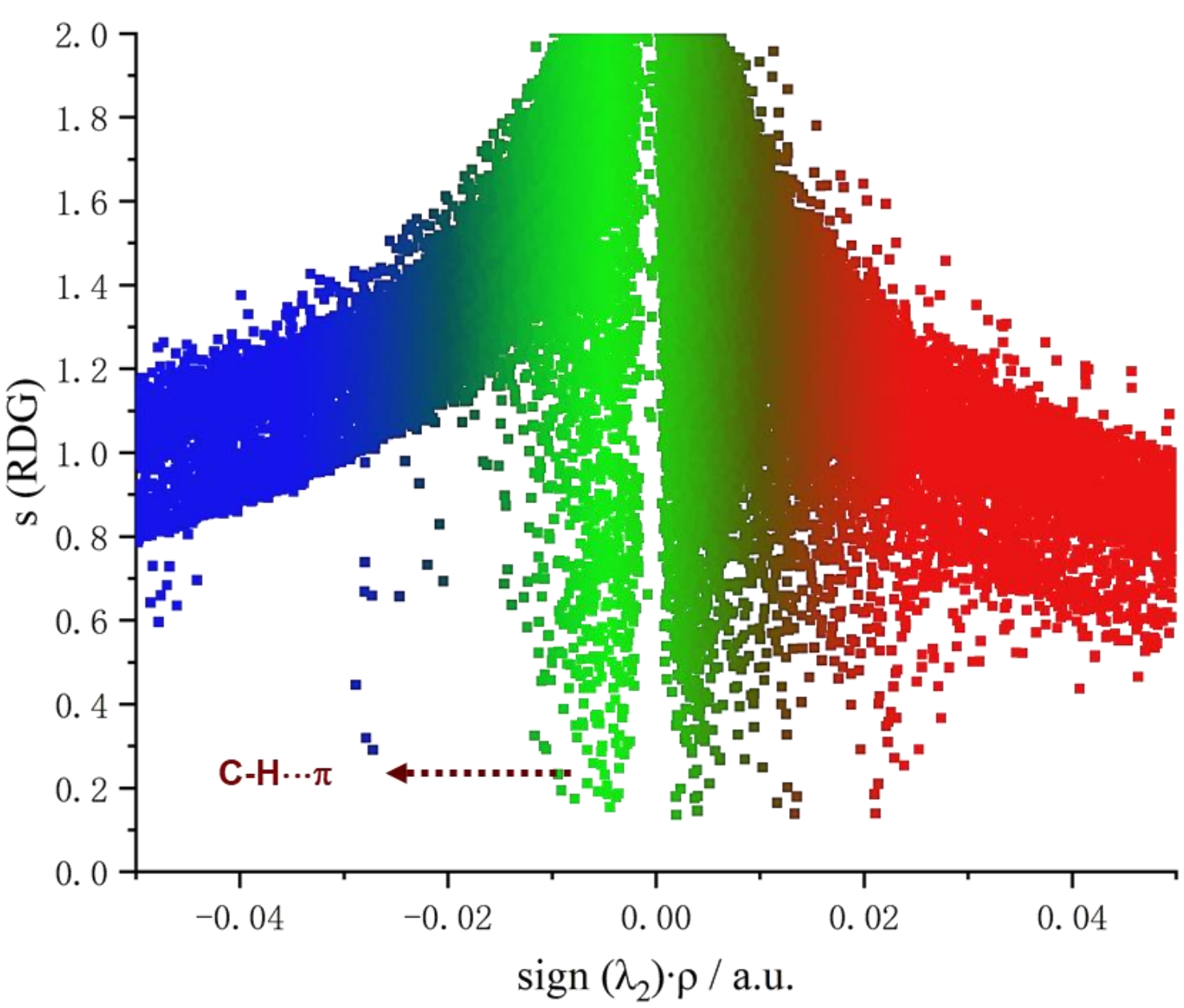

Figure S115. RDG analysis of the adduct between B←N unit in **BN-DPTA2** and OX.

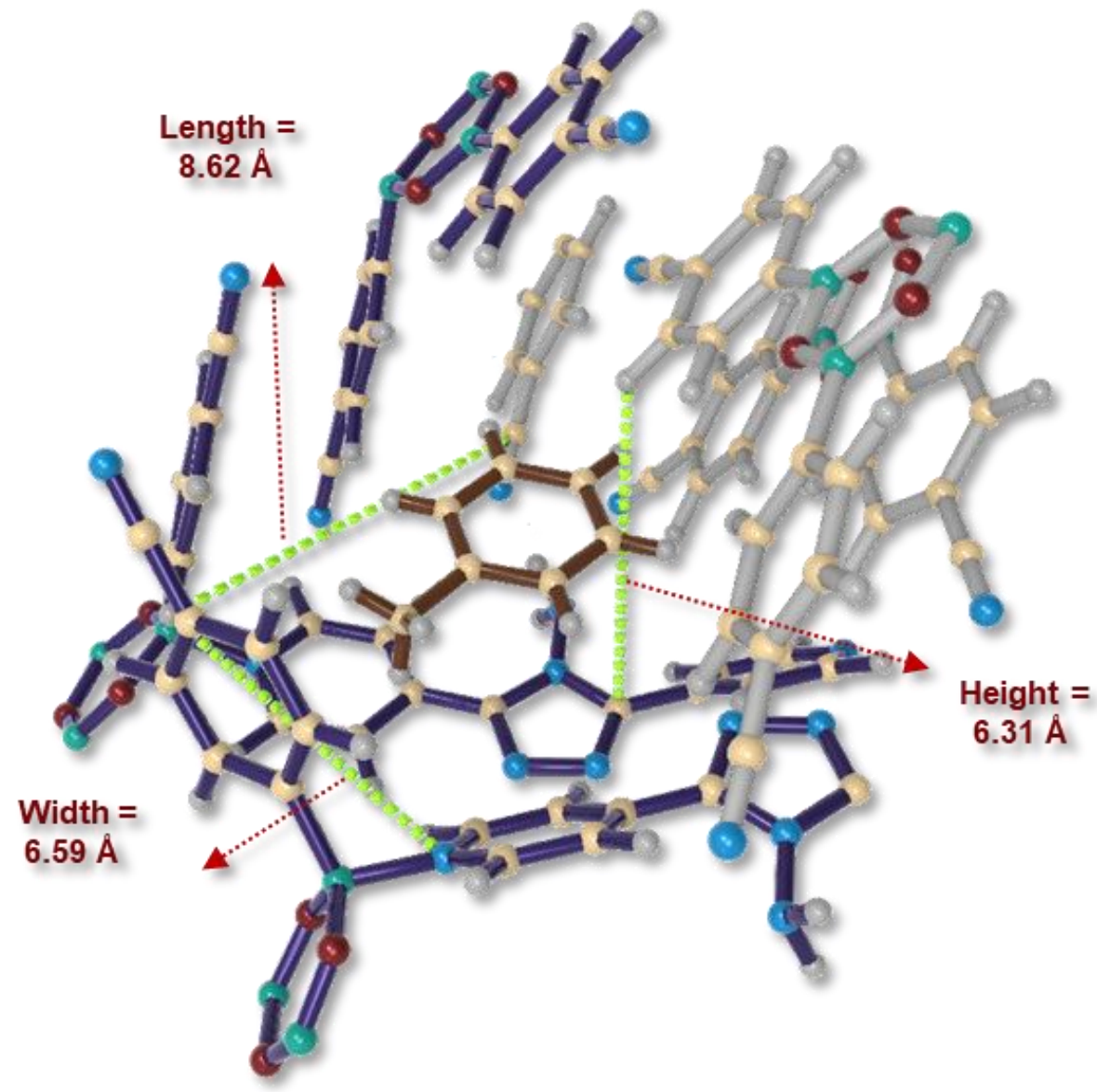

Figure S116. Illustration of the cavity size in **BN-DPTA1**.

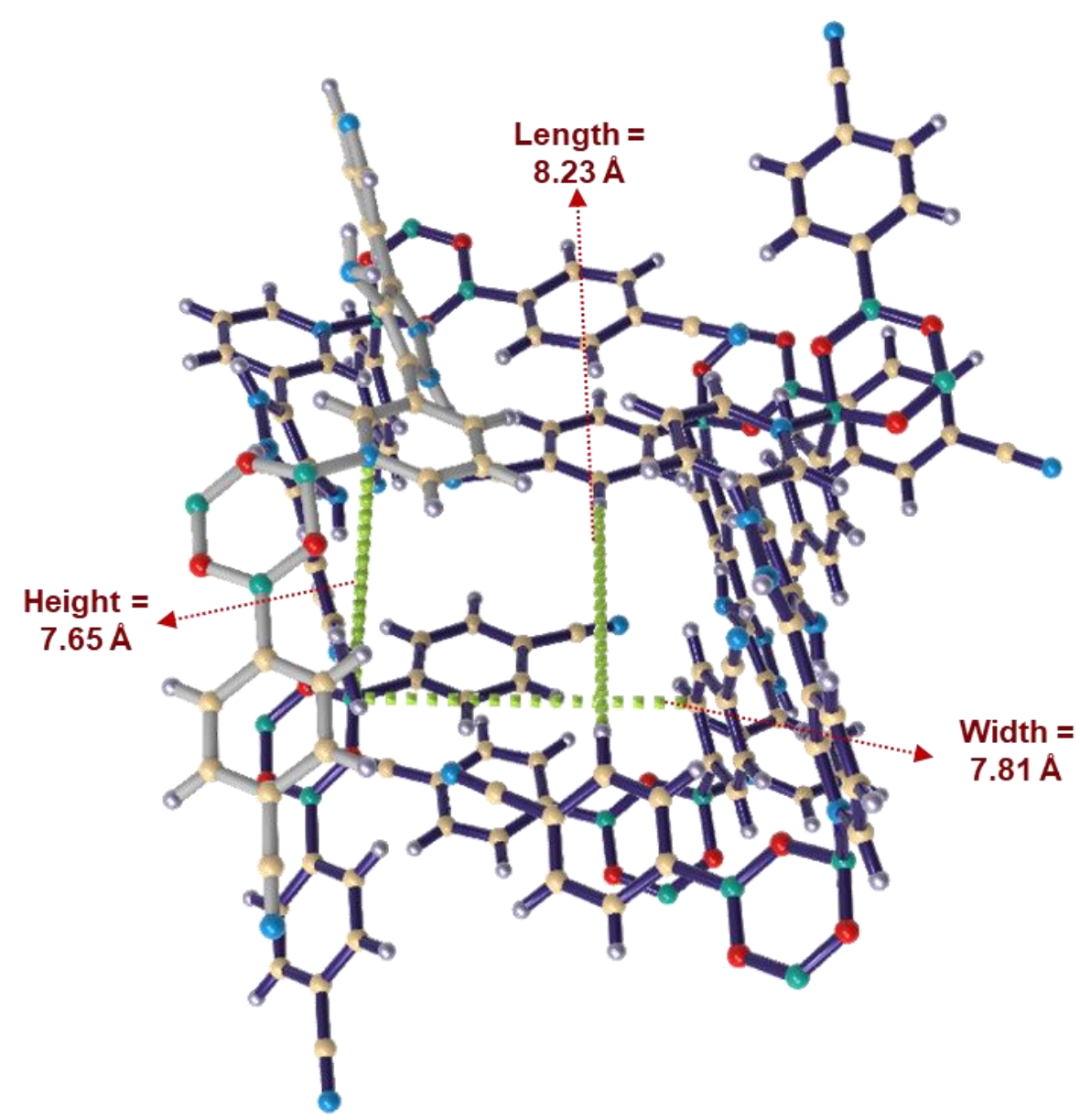

Figure S117. Illustration of the cavity size in **BN-DPTA2**.

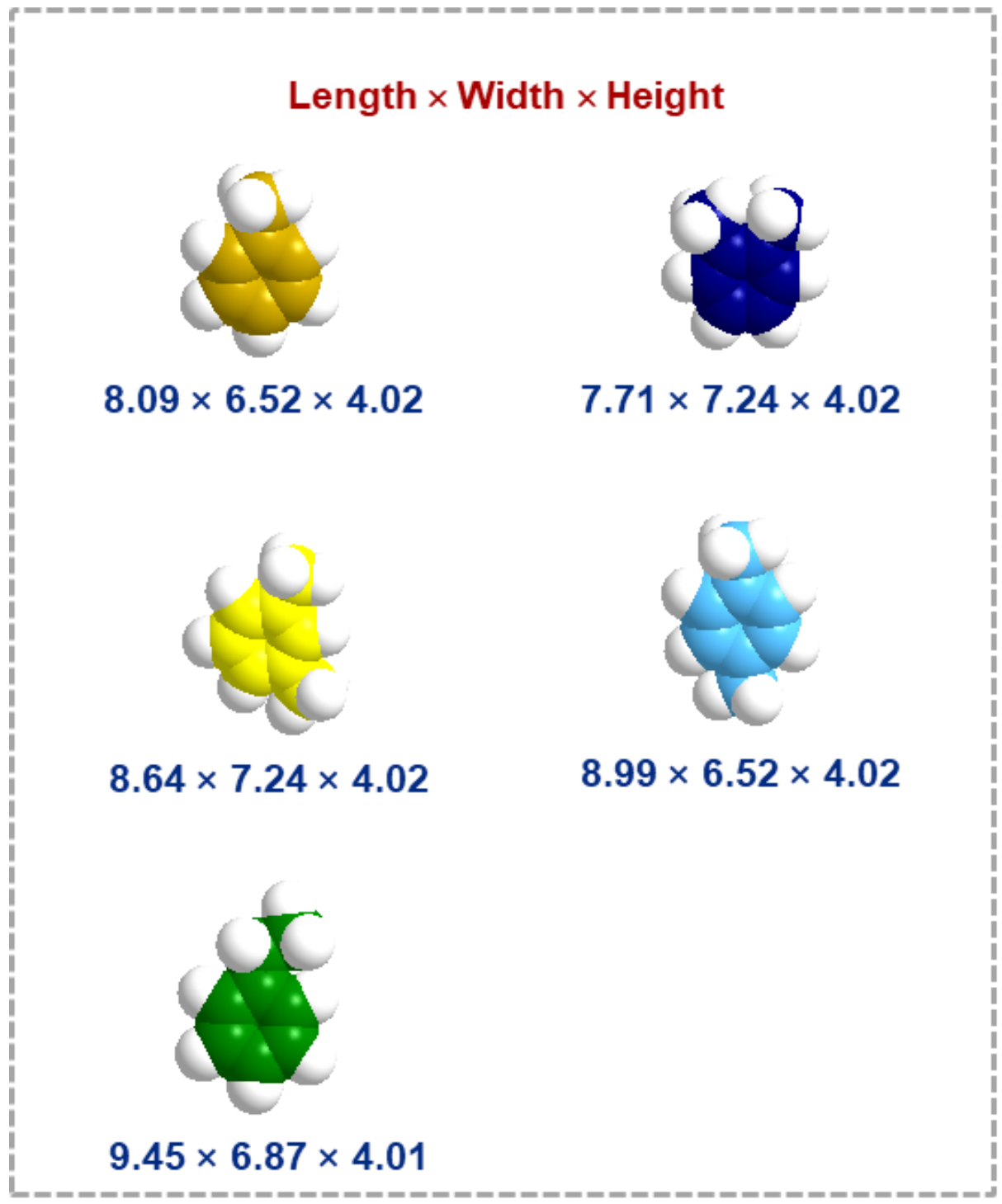

Figure S118. Dimensional sizes of Tol, OX, MX, PX, and EB.

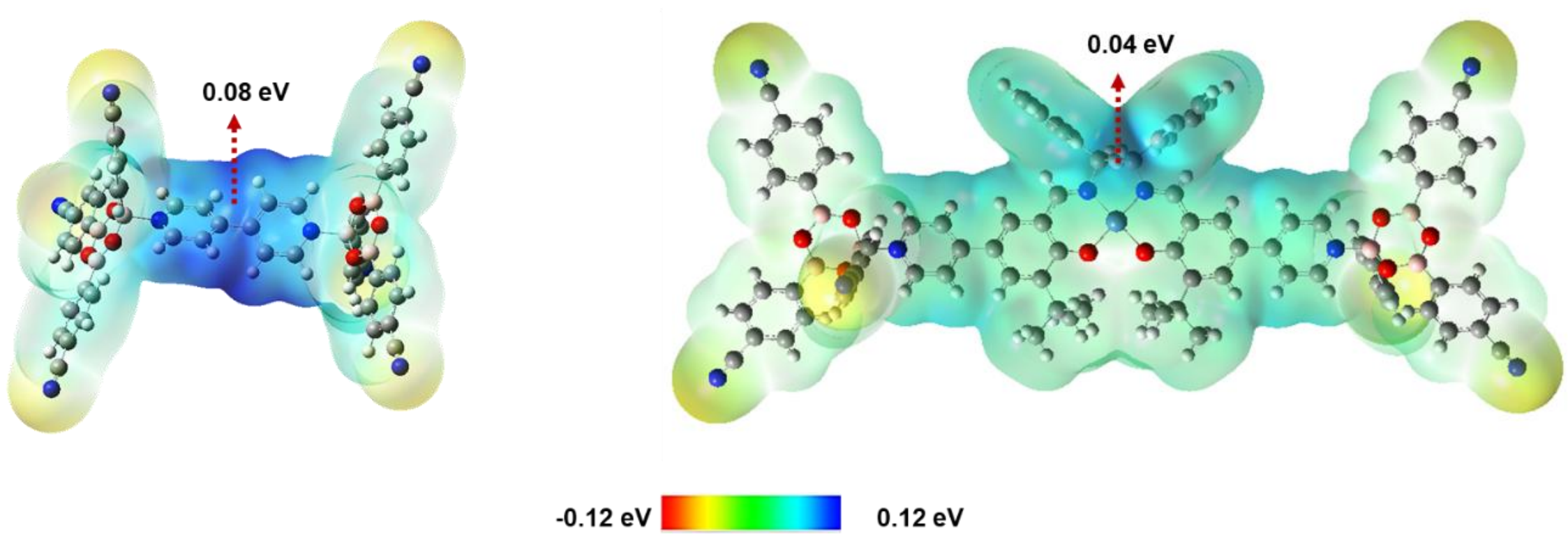

Figure S119. ESP analyses of B←N units in **BN-BP** and **BN-Salen-Ni**.

References

1. Li, J., Ren, Y., Qi, C., & Jiang H. The first porphyrin–salen based chiral metal–organic framework for asymmetric cyanosilylation of aldehydes. *Chem. Commun*. **53**, 8223 (2017).

2. Rahmani, M. *et al*. Highly Selective *p*-Xylene Separation from Mixtures of C8 Aromatics by a Nonporous Molecular Apohost. *J. Am. Chem. Soc*. **145**, 27316–27324 (2023).

3. Li, Y. *et al*. Self-Healing B←N-Based Hydrogen-Bonded Organic Framework for Exclusive Recognition and Separation of Toluene from Methyl Cyclohexane. *J. Am. Chem. Soc*. **146**, 19425–19433 (2024).

4. Li, E., Jie, K., Zhou, Y., Zhao, R. & Huang, F. Post-Synthetic Modification of Nonporous Adaptive Crystals of Pillar[4]arene[1]quinone by Capturing Vaporized Amines. *J. Am. Chem. Soc*. **140**, 15070–15079 (2018).

5. Gaussian 16 Rev. C.01 (Wallingford, CT, 2016).

6. Lu, T. & Chen, F. Multiwfn: A multifunctional wavefunction analyzer. *J. Comput. Chem*. **33**, 580–592 (2012).

7. Humphrey, W., Dalke, A. & Schulten, K. VMD: Visual molecular dynamics. *J. Molec. Graphics* **14**, 33–38 (1996).

8. Zheng, X. *et al*. Solvent-Directed in Situ Stacking Modulation of 2D Covalent Organic Frameworks for Selective Separation of Xylene Isomers. *Angew. Chem. Int. Ed*. **65**, e191502026 (2026).

9. Yu, B. *et al*. A charge-assisted hydrogen-bonded organic framework with polar porosities for selective separation of o-xylene from $C8H_{10}$ aromatic isomers. *Sci. China Chem*. **68**, 2415–2423 (2025).

10. Su, Y., Gao, Q., Ding, X. & Ye, G. From Guest-Induced Crystallization to Molecular Imprinting: Calculation-Guided Discovery of Hydrogen-Bonded Tetrazole Frameworks for p-Xylene Separation. *Angew. Chem. Int. Ed*. **64**, e202423932 (2025).

11. Jie, K. *et al*. Near-Ideal Xylene Selectivity in Adaptive Molecular Pillar[n]arene

Crystals. *J. Am. Chem. Soc*. **140**, 6921–6930 (2018).
12. Goodlett, R. L., Sindt, A. J., Islam, M. F., Smith, M. D. & Shimizu, L. S. Selective Loading of Xylene Isomers in Self-Assembled Triphenylamine bis-Urea Macrocycles. *Cryst. Growth Des*. **22**, 1017−1023 (2022).
13. Sun, Y.-X., Ren, S., Wu, G., Li, X.-S., Wang, H. & Wu, J.-R. Selective adsorptive separation of xylene isomers in vapor and liquid phases by nonporous adaptive crystals of leaning pillar[6]arenes. *Chem. Eng. J*. **521**, 166884 (2025).
14. Tan, H., Chen, Q., Chen, T. & Liu, H. Selective Adsorption and Separation of Xylene Isomers and Benzene/Cyclohexane with Microporous Organic Polymers POP-1. *ACS Appl. Mater. Interfaces* **10**, 32717−32725 (2018).
