# Supplementary material for: Selective Adsorption of o‐Xylene From all C8 Aromatics via Secondary Connectivity Manipulation in B←N Frameworks
Source: Angew Chem Int Ed Engl. 2026 Aug 19;65(42):e1406923. doi: 10.1002/anie.1406923 (PMC13653566; doi:10.1002/anie.1406923)

checkCIF/PLATON report

Structure factors have been supplied for datablock(s) mo_20250720_hujieying_bn_3

THIS REPORT IS FOR GUIDANCE ONLY. IF USED AS PART OF A REVIEW PROCEDURE FOR PUBLICATION, IT SHOULD NOT REPLACE THE EXPERTISE OF AN EXPERIENCED CRYSTALLOGRAPHIC REFEREE.

No syntax errors found. CIF dictionary Interpreting this report

Datablock: mo_20250720_hujieying_bn_3

Bond precision:	C-C = 0.0020 A		Wavelength=0.71073
Cell:	a=24.042(3)	b=8.4996(8)	c=26.442(4)
	alpha=90	beta=97.192(4)	gamma=90
Temperature:	100 K		

	Calculated	Reported
Volume	5360.8(12)	5360.7(12)
Space group	P 21/c	P 1 21/c 1
Hall group	-P 2ybc	-P 2ybc
Moiety formula	C52 H36 B6 N8 O6, C52 H32 B6 N8 O6, 2(C7 H8)	C26 H16 B3 N4 O3, C26 H18 B3 N4 O3, C7 H8
Sum formula	C118 H84 B12 N16 O12	C59 H42 B6 N8 O6
Mr	2047.73	1023.86
Dx,g cm-3	1.269	1.269
Z	2	4
Mu (mm-1)	0.082	0.082
F000	2120.0	2120.0
F000'	2120.84	
h,k,lmax	28,10,31	28,10,31
Nref	9432	9404
Tmin,Tmax	0.998,0.998	0.716,0.746
Tmin'	0.996	

Correction method= # Reported T Limits: Tmin=0.716 Tmax=0.746
AbsCorr = MULTI-SCAN

Data completeness= 0.997   Theta(max)= 25.000

R(reflections)= 0.0384(7538)   wR2(reflections)= 0.1049(9404)

S = 1.016   Npar= 791

The following ALERTS were generated. Each ALERT has the format
test-name_ALERT_alert-type_alert-level.
Click on the hyperlinks for more details of the test.

Alert level C

```
PLAT041_ALERT_1_C Calc. and Reported SumFormula    Strings  Differ     Please Check
              Calc: C118 H84 B12 N16 O12
              Rep.: C59 H42 B6 N8 O6
PLAT042_ALERT_1_C Calc. and Reported MoietyFormula Strings  Differ     Please Check
              Calc: C52 H36 B6 N8 O6, C52 H32 B6 N8 O6, 2(C7 H8)
              Rep.: C26 H16 B3 N4 O3, C26 H18 B3 N4 O3, C7 H8
PLAT222_ALERT_3_C NonSolvent Resd 1  H   Uiso(max)/Uiso(min) Range        4.6 Ratio
PLAT250_ALERT_2_C Large U3/U1 Ratio for <U(i,j)> Tensor(Resd    4)        3.3 Note
PLAT911_ALERT_3_C Missing FCF Refl Between Thmin & STh/L=    0.595         25 Report
                1  1  0,   2  2  0,   5  0  0,  -3  2  1,   4  1  1,   5  1  1,
               -5  1  2,  -2  0  2,   4  0  2,   4  1  2,   5  0  2,  -1  2  3,
                0  1  3,   1  1  3,  -4  1  4,  -1  1  4,  -1  2  4,   0  0  4,
                0  1  4,   0  2  4,   2  0  4,  -1  2  5,   0  2  5,   1  2  5,
                3  0  6,
PLAT913_ALERT_3_C Missing # of Very Strong Reflections in FCF ....         12 Note
               -2  0  2,   4  0  2,   4  1  2,  -1  2  3,  -4  1  4,  -1  1  4,
               -1  2  4,   0  2  4,   2  0  4,   0  2  5,   1  2  5,   3  0  6,
PLAT934_ALERT_3_C Number of (Iobs-Icalc)/Sigma(W) > 10 Outliers ..          1 Check
               -3  6  9,
PLAT977_ALERT_2_C Check Negative Difference Density on H02C      .      -0.34 eA-3
```

Alert level G

```
PLAT003_ALERT_2_G Number of Uiso or U(i,j)  Restrained non-H-Atoms          8 Report
PLAT045_ALERT_1_G Calculated and Reported Z Differ by a Factor ...      0.500 Check
PLAT174_ALERT_4_G The CIF-Embedded .res File Contains FLAT Records          2 Report
PLAT178_ALERT_4_G The CIF-Embedded .res File Contains SIMU Records          1 Report
PLAT187_ALERT_4_G The CIF-Embedded .res File Contains RIGU Records          1 Report
PLAT188_ALERT_3_G A Non-default SIMU Restraint Value has been used     0.0100 Report
PLAT230_ALERT_2_G Hirshfeld Test Diff for    N009     --C1        .       7.0 s.u.
PLAT230_ALERT_2_G Hirshfeld Test Diff for    C01R     --C01U      .       5.9 s.u.
PLAT230_ALERT_2_G Hirshfeld Test Diff for    C00V     --C019      .       5.7 s.u.
PLAT299_ALERT_4_G Atom Site Occupancy Constrained at ............         0.5 Check
              C1      C024    C025    C0
PLAT299_ALERT_4_G Atom Site Occupancy Constrained at ............        0.25 Check
              C2B     C3B     C4B     C5B     C7B     C8B     C9      H2B
              H3B     H4B     H5B     H7B     H9A     H9B     H9C
PLAT300_ALERT_4_G Atom Site Occupancy of C2A         Constrained at       0.75 Check
PLAT300_ALERT_4_G Atom Site Occupancy of C3A         Constrained at       0.75 Check
PLAT300_ALERT_4_G Atom Site Occupancy of C4A         Constrained at       0.75 Check
PLAT300_ALERT_4_G Atom Site Occupancy of C5A         Constrained at       0.75 Check
PLAT300_ALERT_4_G Atom Site Occupancy of C6          Constrained at       0.75 Check
```

```
PLAT300_ALERT_4_G Atom Site Occupancy of C7A       Constrained at       0.75 Check
PLAT300_ALERT_4_G Atom Site Occupancy of C8A       Constrained at       0.75 Check
PLAT300_ALERT_4_G Atom Site Occupancy of H2A       Constrained at       0.75 Check
PLAT300_ALERT_4_G Atom Site Occupancy of H3A       Constrained at       0.75 Check
PLAT300_ALERT_4_G Atom Site Occupancy of H4A       Constrained at       0.75 Check
PLAT300_ALERT_4_G Atom Site Occupancy of H6A       Constrained at       0.75 Check
PLAT300_ALERT_4_G Atom Site Occupancy of H6B       Constrained at       0.75 Check
PLAT300_ALERT_4_G Atom Site Occupancy of H6C       Constrained at       0.75 Check
PLAT300_ALERT_4_G Atom Site Occupancy of H7A       Constrained at       0.75 Check
PLAT300_ALERT_4_G Atom Site Occupancy of H8A       Constrained at       0.75 Check
PLAT301_ALERT_3_G Main Residue  Disorder .............(Resd    1)          6% Note
PLAT302_ALERT_4_G Anion/Solvent/Minor-Residue Disorder (Resd    3)        100% Note
PLAT302_ALERT_4_G Anion/Solvent/Minor-Residue Disorder (Resd    4)        100% Note
PLAT304_ALERT_4_G Non-Integer Number of Atoms in ..... (Resd    3)      11.25 Check
PLAT304_ALERT_4_G Non-Integer Number of Atoms in ..... (Resd    4)       3.75 Check
PLAT413_ALERT_2_G Short Inter XH3 .. XHn     H02B     ..H9C      .       2.02 Ang.
                                                x,1+y,z  =      1_565 Check
PLAT720_ALERT_4_G Number of Unusual/Non-Standard Labels ..........        107 Note
              O001    O002    O003    O004    O005    O006    N007    N008
              N009    C00A    C00B    N00C    C00D    H00D    C00E    C00F
              C00G    H00G    C00H    C00I    H00I    C00J    H00J    N00K
              C00L    H00L    C00M    H00M    C00N    H00N    C00O    H00O
              C00P    H00P    N00Q    C00R    H00R    C00S    H00S    C00T
              H00T    C00U    C00V    C00W    H00W    C00X    H00X    C00Y
              C00Z    C010    C011    C012    C013    H013    C014    H014
              C015    C016    H016    C017    C018    H018    C019    C01A
              H01A    N01B    C01C    H01C    C01D    H01D    C01E    H01E
              C01F    C01G    H01G    C01H    H01H    C01I    H01I    C01J
              N01K    C01L    H01L    B01M    C01N    H01N    C01O    C01P
              H01P    C01Q    H01Q    C01R    B01S    C01T    H01T    C01U
              B01V    B01W    B01X    B01Y    C024    H02A    C025    H02C
              C0      H02D    H02B
PLAT860_ALERT_3_G Number of Least-Squares Restraints ............          50 Note
PLAT909_ALERT_3_G Percentage of I>2sig(I) Data at Theta(Max) Still        61% Note
PLAT910_ALERT_3_G Missing FCF Reflection(s) Below Theta(Min)[Deg]=       1.71 Note
                1  0  0,  -1  0  2,   0  0  2,
PLAT967_ALERT_5_G Note: Two-Theta Cutoff Value in Embedded .res ..       50.0 Degree
PLAT969_ALERT_5_G The 'Henn et al.' R-Factor-gap value ..........       2.895 Note
              Predicted wR2: Based on SigI**2  3.62 or SHELX Weight 10.32
PLAT978_ALERT_2_G Number C-C Bonds with Positive Residual Density.          7 Info
PLAT994_ALERT_1_G SHELXL .ins Contains no or MERG 0 Instruction ..          ! Note
```

```
   0 ALERT level A = Most likely a serious problem - resolve or explain
   0 ALERT level B = A potentially serious problem, consider carefully
   8 ALERT level C = Check. Ensure it is not caused by an omission or oversight
  40 ALERT level G = General information/check it is not something unexpected

   4 ALERT type 1 CIF construction/syntax error, inconsistent or missing data
   8 ALERT type 2 Indicator that the structure model may be wrong or deficient
   9 ALERT type 3 Indicator that the structure quality may be low
  25 ALERT type 4 Improvement, methodology, query or suggestion
   2 ALERT type 5 Informative message, check
```

It is advisable to attempt to resolve as many as possible of the alerts in all categories. Often the minor alerts point to easily fixed oversights, errors and omissions in your CIF or refinement strategy, so attention to these fine details can be worthwhile. It is up to the individual to critically assess their own results and, if necessary, seek expert advice.

PLATON version of 15/01/2026; check.def file version of 02/01/2026

---

duplicate check

No duplication found

Datablock mo_20250720_hujieying_bn_3 - ellipsoid plot

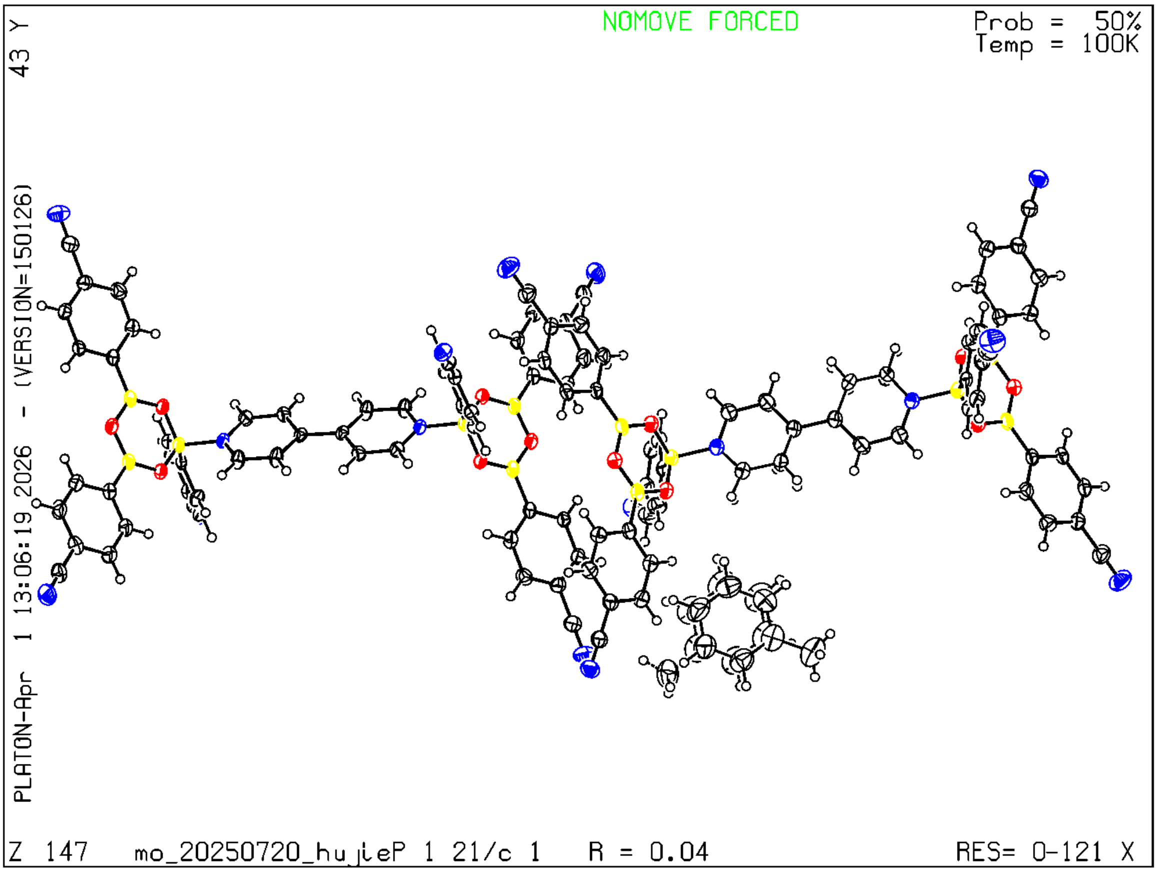

Supplement: Supplementary file 2 [file ANIE-65-e1406923-s001.zip › SC DATA/单晶/checkcif_BN-BP.pdf]
