# Supplementary material for: Selective Adsorption of o‐Xylene From all C8 Aromatics via Secondary Connectivity Manipulation in B←N Frameworks
Source: Angew Chem Int Ed Engl. 2026 Aug 19;65(42):e1406923. doi: 10.1002/anie.1406923 (PMC13653566; doi:10.1002/anie.1406923)

checkCIF/PLATON report

Structure factors have been supplied for datablock(s) mo_20250919_heqianlong_lxs_bn1

THIS REPORT IS FOR GUIDANCE ONLY. IF USED AS PART OF A REVIEW PROCEDURE FOR PUBLICATION, IT SHOULD NOT REPLACE THE EXPERTISE OF AN EXPERIENCED CRYSTALLOGRAPHIC REFEREE.

No syntax errors found. CIF dictionary Interpreting this report

Datablock: mo_20250919_heqianlong_lxs_bn1

```
Bond precision:   C-C = 0.0050 A           Wavelength=0.71073

Cell:           a=14.9920(8)      b=15.2739(10)     c=15.5078(10)
                alpha=73.821(2)   beta=63.395(2)    gamma=71.560(2)
Temperature:    173 K
```

	Calculated	Reported
Volume	2972.5(3)	2972.5(3)
Space group	P -1	P -1
Hall group	-P 1	-P 1
Moiety formula	C54 H34 B6 N12 O6, C7 H8	C54 H34 B6 N12 O6, C7 H8
Sum formula	C61 H42 B6 N12 O6	C61 H43 B6 N11 O7
Mr	1103.93	1106.92
Dx,g cm-3	1.233	1.237
Z	2	2
Mu (mm-1)	0.081	0.081
F000	1140.0	1144.0
F000’	1140.44	
h,k,lmax	18,18,18	18,18,18
Nref	10920	10885
Tmin,Tmax	0.996,0.996	0.716,0.745
Tmin’	0.996	

Correction method= # Reported T Limits: Tmin=0.716 Tmax=0.745
AbsCorr = MULTI-SCAN

Data completeness= 0.997 Theta(max)= 25.371

R(reflections)= 0.0624(6366) wR2(reflections)= 0.1983(10885)

S = 1.032 Npar= 739

The following ALERTS were generated. Each ALERT has the format
test-name_ALERT_alert-type_alert-level.
Click on the hyperlinks for more details of the test.

Alert level C

```
PLAT041_ALERT_1_C Calc. and Reported SumFormula    Strings  Differ     Please Check
              Calc: C61 H42 B6 N12 O6
              Rep.: C61 H43 B6 N11 O7
PLAT043_ALERT_1_C Calculated and Reported Mol. Weight Differ by ..       2.99 Check
PLAT068_ALERT_1_C Reported F000 Differs from Calcd (or Missing)...     Please Check
PLAT202_ALERT_3_C Isotropic non-H Atoms in Anion/Solvent .........          7 Check
              C1      C2      C3      C4      C5      C6      C7
PLAT220_ALERT_2_C NonSolvent   Resd 1  N   Ueq(max)/Ueq(min) Range        4.1 Ratio
PLAT241_ALERT_2_C High  MainResAtom  Ueq as Compared to Neighbours       C024 Check
PLAT242_ALERT_2_C Low   MainResAtom  Ueq as Compared to Neighbours       C00I Check
PLAT242_ALERT_2_C Low   MainResAtom  Ueq as Compared to Neighbours       C01Q Check
PLAT242_ALERT_2_C Low   MainResAtom  Ueq as Compared to Neighbours       C019 Check
PLAT242_ALERT_2_C Low   MainResAtom  Ueq as Compared to Neighbours       C026 Check
PLAT340_ALERT_3_C Low Bond Precision on  C-C Bonds ...............      0.005 Ang.
PLAT420_ALERT_2_C D-H Bond Without Acceptor  N00A     --H00B     .     Please Check
PLAT911_ALERT_3_C Missing FCF Refl Between Thmin & STh/L=    0.600         22 Report
               -2  2  0,  -2  3  0,   0  4  0,   1  4  0,   2  4  0,   2 -3  1,
                2  0  1,   3  0  1,  -1  1  1,  -2  2  1,   0  3  1,   3  4  1,
                1 -2  2,   0 -1  2,   2  0  2,   0  1  2,   0  2  2,   3  2  2,
               -1  3  2,   1  1  3,   2  1  3,   0  3  3,
PLAT913_ALERT_3_C Missing # of Very Strong Reflections in FCF ....         16 Note
               -2  2  0,  -2  3  0,   0  4  0,   1  4  0,   2  4  0,   2  0  1,
               -1  1  1,   3  4  1,   1 -2  2,   2  0  2,   0  2  2,   3  2  2,
               -1  3  2,   1  1  3,   2  1  3,   0  3  3,
PLAT976_ALERT_2_C Check Calcd Resid. Dens.  0.97Ang From N00A    .      -0.41 eA-3
PLAT977_ALERT_2_C Check Negative Difference Density on H00B      .      -0.35 eA-3
```

Alert level G

```
FORMU01_ALERT_1_G  There is a discrepancy between the atom counts in the
            _chemical_formula_sum and _chemical_formula_moiety. This is
            usually due to the moiety formula being in the wrong format.
            Atom count from _chemical_formula_sum:   C61 H43 B6 N11 O7
            Atom count from _chemical_formula_moiety:C61 H42 B6 N12 O6
FORMU01_ALERT_2_G  There is a discrepancy between the atom counts in the
            _chemical_formula_sum and the formula from the _atom_site* data.
            Atom count from _chemical_formula_sum:C61 H43 B6 N11 O7
            Atom count from the _atom_site data:  C61 H42 B6 N12 O6
CELLZ01_ALERT_1_G Difference between formula and atom_site contents detected.
CELLZ01_ALERT_1_G ALERT: Large difference may be due to a
            symmetry error - see SYMMG tests
           From the CIF: _cell_formula_units_Z    2
```

```
From the CIF: _chemical_formula_sum  C61 H43 B6 N11 O7
TEST: Compare cell contents of formula and atom_site data

atom    Z*formula  cif sites diff
C        122.00    122.00    0.00
H         86.00     84.00    2.00
B         12.00     12.00    0.00
N         22.00     24.00   -2.00
O         14.00     12.00    2.00
PLAT002_ALERT_2_G Number of Distance or Angle Restraints on AtSite         14 Note
PLAT003_ALERT_2_G Number of Uiso or U(i,j)  Restrained non-H-Atoms         14 Report
PLAT007_ALERT_5_G Number of Unrefined Donor-H Atoms .............           2 Report
             H00A    H00B
PLAT154_ALERT_1_G The s.u.'s on the Cell Angles are Equal ..(Note)      0.002 Degree
PLAT173_ALERT_4_G The CIF-Embedded .res File Contains DANG Records          3 Report
PLAT174_ALERT_4_G The CIF-Embedded .res File Contains FLAT Records          1 Report
PLAT175_ALERT_4_G The CIF-Embedded .res File Contains SAME Records          1 Report
PLAT178_ALERT_4_G The CIF-Embedded .res File Contains SIMU Records          1 Report
PLAT188_ALERT_3_G A Non-default SIMU Restraint Value has been used     0.0100 Report
PLAT189_ALERT_3_G A Non-default SAME Restraint Value for First Par     0.0100 Report
PLAT302_ALERT_4_G Anion/Solvent/Minor-Residue Disorder (Resd    2)       100% Note
PLAT302_ALERT_4_G Anion/Solvent/Minor-Residue Disorder (Resd    3)       100% Note
PLAT304_ALERT_4_G Non-Integer Number of Atoms in ..... (Resd    2)       8.37 Check
PLAT304_ALERT_4_G Non-Integer Number of Atoms in ..... (Resd    3)       6.63 Check
PLAT720_ALERT_4_G Number of Unusual/Non-Standard Labels ..........        115 Note
             O001    O002    O003    O004    O005    O006    N027    N00A
             H00A    H00B    N00B    N00C    C00F    N01J    N01O    N01P
             N01V    N01Z    N008    N009    C00D    C00E    C00G    C00H
             H00H    C00I    C00J    C00K    H00K    C00L    H00L    C00M
             C00N    H00N    C00O    C00P    H00P    C00Q    C00R    H00R
             C00S    C00T    H00T    C00U    H00U    C00V    H00V    C00W
             H00W    C00X    H00X    C00Y    H00Y    C00Z    H00Z    C01A
             H01A    C01B    H01B    C01C    H01C    C01E    H01E    C01F
             H01F    C01G    C01H    H01H    C01K    H01K    C01L    H01L
             C01N    H01N    C01Q    C01R    C01S    H01S    C01T    H01T
             C01W    N007    C010    C011    H011    C012    C013    H013
             C014    H014    C015    H015    C016    C017    H017    C018
             H018    C019    C020    C021    C022    C023    H023    C024
             H024    C026    B01D    B01I    B01M    B01U    B01X    B01Y
             H7AA    H7AB    H7AC
PLAT860_ALERT_3_G Number of Least-Squares Restraints ............          60 Note
PLAT883_ALERT_1_G Absent Datum for _atom_sites_solution_primary ..     Please Do !
PLAT910_ALERT_3_G Missing FCF Reflection(s) Below Theta(Min)[Deg]=       1.78 Note
               1  0  0,   0  1  0,   0  0  1,   1  0  1,
PLAT912_ALERT_4_G Missing # of FCF Reflections Above STh/L=  0.600          8 Note
PLAT969_ALERT_5_G The 'Henn et al.' R-Factor-gap value ..........       4.502 Note
             Predicted wR2: Based on SigI**2  4.41 or SHELX Weight 19.21
PLAT978_ALERT_2_G Number C-C Bonds with Positive Residual Density.          4 Info
PLAT992_ALERT_5_G Repd & Actual _reflns_number_gt Values Differ by          2 Check
PLAT994_ALERT_1_G SHELXL .ins Contains no or MERG 0 Instruction ..          ! Note
```

---

0 **ALERT level A** = Most likely a serious problem - resolve or explain
0 **ALERT level B** = A potentially serious problem, consider carefully
16 **ALERT level C** = Check. Ensure it is not caused by an omission or oversight
27 **ALERT level G** = General information/check it is not something unexpected

```
 9 ALERT type 1 CIF construction/syntax error, inconsistent or missing data
13 ALERT type 2 Indicator that the structure model may be wrong or deficient
 8 ALERT type 3 Indicator that the structure quality may be low
10 ALERT type 4 Improvement, methodology, query or suggestion
 3 ALERT type 5 Informative message, check
```

It is advisable to attempt to resolve as many as possible of the alerts in all categories. Often the minor alerts point to easily fixed oversights, errors and omissions in your CIF or refinement strategy, so attention to these fine details can be worthwhile. It is up to the individual to critically assess their own results and, if necessary, seek expert advice.

PLATON version of 15/01/2026; check.def file version of 02/01/2026

---

duplicate check

No duplication found

Datablock mo_20250919_heqianlong_lxs_bn1 - ellipsoid plot

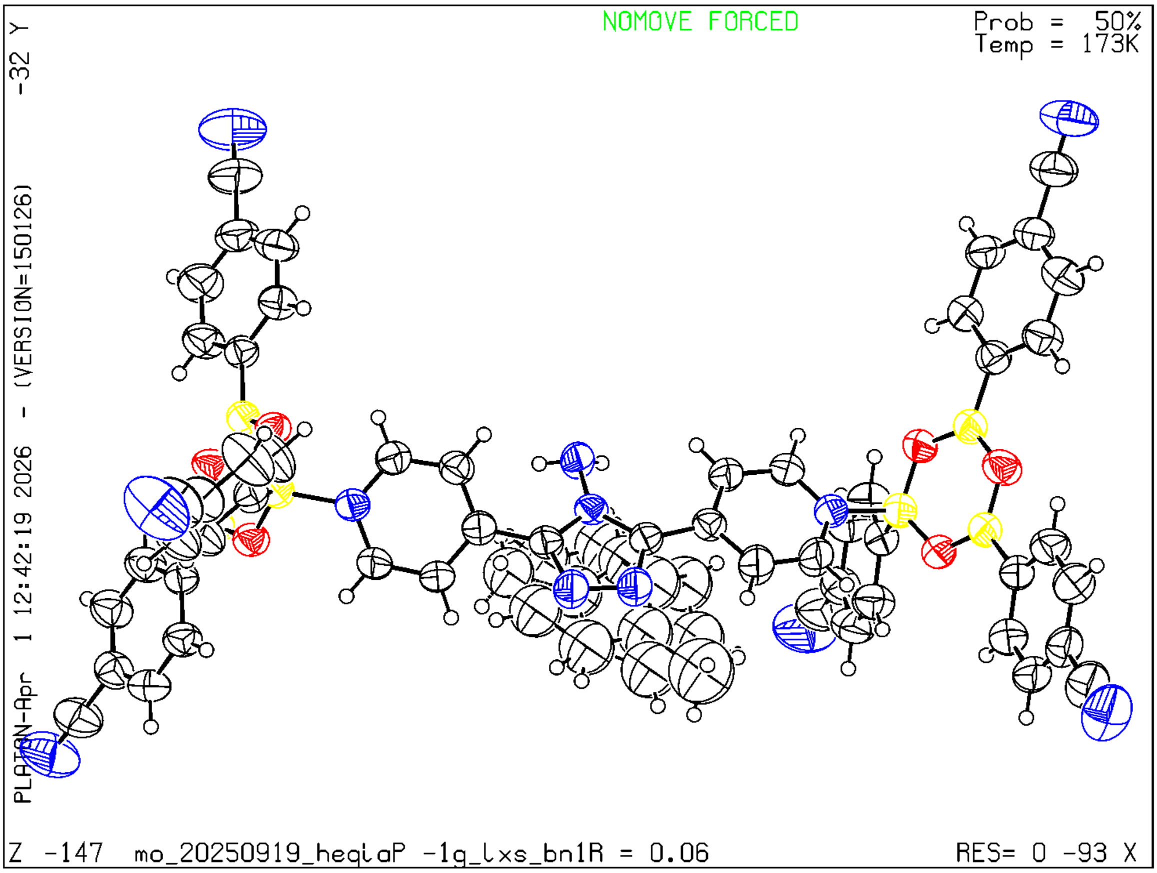

Supplement: Supplementary file 2 [file ANIE-65-e1406923-s001.zip › SC DATA/单晶/checkcif_BN-DPTA1-after soaking.pdf]
