# Supplementary material for: Selective Adsorption of o‐Xylene From all C8 Aromatics via Secondary Connectivity Manipulation in B←N Frameworks
Source: Angew Chem Int Ed Engl. 2026 Aug 19;65(42):e1406923. doi: 10.1002/anie.1406923 (PMC13653566; doi:10.1002/anie.1406923)

checkCIF/PLATON report

Structure factors have been supplied for datablock(s) xiangshui12_Mo100K_auto

THIS REPORT IS FOR GUIDANCE ONLY. IF USED AS PART OF A REVIEW PROCEDURE FOR PUBLICATION, IT SHOULD NOT REPLACE THE EXPERTISE OF AN EXPERIENCED CRYSTALLOGRAPHIC REFEREE.

No syntax errors found. CIF dictionary Interpreting this report

Datablock: xiangshui12_Mo100K_auto

```
Bond precision:   C-C = 0.0037 A           Wavelength=0.71073

Cell:            a=14.8594(8)       b=14.9439(6)      c=15.4382(6)
                 alpha=64.041(4)    beta=74.405(4)    gamma=72.145(4)
Temperature:     100 K
```

	Calculated	Reported
Volume	2897.3(3)	2897.3(3)
Space group	P -1	P -1
Hall group	-P 1	-P 1
Moiety formula	C54 H34 B6 N12 O6, C7 H8	C54 H34 B6 N12 O6, C7 H8
Sum formula	C61 H42 B6 N12 O6	C61 H42 B6 N12 O6
Mr	1103.93	1104.04
Dx,g cm-3	1.265	1.266
Z	2	2
Mu (mm-1)	0.083	0.083
F000	1140.0	1140.7
F000'	1140.44	
h,k,lmax	18,18,19	18,18,19
Nref	11391	11230
Tmin,Tmax	0.984,0.987	0.897,1.000
Tmin'	0.984	

```
Correction method= # Reported T Limits: Tmin=0.897 Tmax=1.000
AbsCorr = MULTI-SCAN

Data completeness= 0.986            Theta(max)= 26.000

R(reflections)= 0.0580( 7107)          wR2(reflections)= 0.1257( 11230)

S = 1.024                   Npar= 934
```

The following ALERTS were generated. Each ALERT has the format
test-name_ALERT_alert-type_alert-level.
Click on the hyperlinks for more details of the test.

Alert level C

```
PLAT220_ALERT_2_C NonSolvent   Resd 1  N   Ueq(max)/Ueq(min) Range        3.5 Ratio
PLAT906_ALERT_3_C Large K Value in the Analysis of Variance ......     14.990 Check
PLAT906_ALERT_3_C Large K Value in the Analysis of Variance ......      2.136 Check
PLAT910_ALERT_3_C Missing FCF Reflection(s) Below Theta(Min)[Deg]=       2.53 Note
                1  0  0,  -1  1  0,   0  1  0,   1  1  0,  -1  0  1,   0  0  1,
                1  0  1,   0  1  1,   1  1  1,
PLAT911_ALERT_3_C Missing FCF Refl Between Thmin & STh/L=    0.600          90 Report
              -14  6  0, -13  6  0, -13  7  0,  13 -7  1,  13 -6  1,  14 -6  1,
               -1 -1  1,   0  2  1,   0  3  1,  -1  4  1,  -2  5  1,  -1  5  1,
                8  5  1, -14  6  1, -13  6  1,  -3  6  1,  -2  6  1, -13  7  1,
               -3  7  1,  -2  7  1,  -2  8  1,  13 -6  2,   1  1  2,   0  4  2,
              -14  6  2, -13  6  2,  -2  6  2, -13  7  2, -12  7  2,  -3  7  2,
                   (   60 More Missing: see the .ckf listing file)
```

Alert level G

```
PLAT002_ALERT_2_G Number of Distance or Angle Restraints on AtSite         14 Note
PLAT003_ALERT_2_G Number of Uiso or U(i,j)  Restrained non-H-Atoms         14 Report
PLAT068_ALERT_1_G Reported F000 Differs from Calcd (or Missing)...     Please Check
PLAT154_ALERT_1_G The s.u.'s on the Cell Angles are Equal ..(Note)      0.004 Degree
PLAT174_ALERT_4_G The CIF-Embedded .res File Contains FLAT Records          1 Report
PLAT176_ALERT_4_G The CIF-Embedded .res File Contains SADI Records         15 Report
PLAT186_ALERT_4_G The CIF-Embedded .res File Contains ISOR Records          1 Report
PLAT187_ALERT_4_G The CIF-Embedded .res File Contains RIGU Records          2 Report
PLAT190_ALERT_3_G A Non-default RIGU Restraint Value for First Par     0.0010 Report
PLAT190_ALERT_3_G A Non-default RIGU Restraint Value for SecondPar     0.0010 Report
PLAT190_ALERT_3_G A Non-default RIGU Restraint Value for First Par     0.0010 Report
PLAT190_ALERT_3_G A Non-default RIGU Restraint Value for SecondPar     0.0010 Report
PLAT191_ALERT_3_G A Non-default SADI Restraint Value has been used     0.0400 Report
PLAT191_ALERT_3_G A Non-default SADI Restraint Value has been used     0.0400 Report
PLAT191_ALERT_3_G A Non-default SADI Restraint Value has been used     0.0400 Report
PLAT191_ALERT_3_G A Non-default SADI Restraint Value has been used     0.0400 Report
PLAT191_ALERT_3_G A Non-default SADI Restraint Value has been used     0.0400 Report
PLAT191_ALERT_3_G A Non-default SADI Restraint Value has been used     0.0400 Report
PLAT191_ALERT_3_G A Non-default SADI Restraint Value has been used     0.0400 Report
PLAT191_ALERT_3_G A Non-default SADI Restraint Value has been used     0.0400 Report
PLAT302_ALERT_4_G Anion/Solvent/Minor-Residue Disorder (Resd    2)       100% Note
PLAT302_ALERT_4_G Anion/Solvent/Minor-Residue Disorder (Resd    3)       100% Note
PLAT304_ALERT_4_G Non-Integer Number of Atoms in ..... (Resd    2)      12.75 Check
PLAT304_ALERT_4_G Non-Integer Number of Atoms in ..... (Resd    3)       2.25 Check
PLAT720_ALERT_4_G Number of Unusual/Non-Standard Labels .........          18 Note
              H7SA    H7SB    H7SC    C1SA    C2SA    H2SA    C3SA    H3SA
              C4SA    H4SA    C5SA    H5SA    C6SA    H6SA    C7SA    H7SD
              H7SE    H7SF
PLAT802_ALERT_4_G CIF Input Record(s) with more than 80 Characters          1 Info
PLAT860_ALERT_3_G Number of Least-Squares Restraints ............         166 Note
PLAT883_ALERT_1_G Absent Datum for _atom_sites_solution_primary ..     Please Do !
PLAT912_ALERT_4_G Missing # of FCF Reflections Above STh/L=  0.600         61 Note
PLAT933_ALERT_2_G Number of HKL-OMIT Records in Embedded .res File         10 Note
               -3  7  2,  -2  8  1,  -1 -1  1,  -1  0  1,  -1  8  4,   1 -1  0,
```

```
                1  1  0,   1  1  1,   1  1  2,   8  5  1,
PLAT941_ALERT_3_G Average HKL Measurement Multiplicity ..........        1.7 Low
PLAT961_ALERT_5_G Dataset Contains no Negative Intensities ......     Please Check
PLAT967_ALERT_5_G Note: Two-Theta Cutoff Value in Embedded .res ..       52.0 Degree
PLAT969_ALERT_5_G The 'Henn et al.' R-Factor-gap value ..........      1.974 Note
              Predicted wR2: Based on SigI**2  6.37 or SHELX Weight 12.28
PLAT978_ALERT_2_G Number C-C Bonds with Positive Residual Density.         11 Info
PLAT979_ALERT_1_G NoSpherA2 Scattering Factors Used .............     Please Note
```

```
   0 ALERT level A = Most likely a serious problem - resolve or explain
   0 ALERT level B = A potentially serious problem, consider carefully
   5 ALERT level C = Check. Ensure it is not caused by an omission or oversight
  36 ALERT level G = General information/check it is not something unexpected

   4 ALERT type 1 CIF construction/syntax error, inconsistent or missing data
   5 ALERT type 2 Indicator that the structure model may be wrong or deficient
  18 ALERT type 3 Indicator that the structure quality may be low
  11 ALERT type 4 Improvement, methodology, query or suggestion
   3 ALERT type 5 Informative message, check
```

It is advisable to attempt to resolve as many as possible of the alerts in all categories. Often the minor alerts point to easily fixed oversights, errors and omissions in your CIF or refinement strategy, so attention to these fine details can be worthwhile. In order to resolve some of the more serious problems it may be necessary to carry out additional measurements or structure refinements. However, the purpose of your study may justify the reported deviations and the more serious of these should normally be commented upon in the discussion or experimental section of a paper or in the "special_details" fields of the CIF. checkCIF was carefully designed to identify outliers and unusual parameters, but every test has its limitations and alerts that are not important in a particular case may appear. Conversely, the absence of alerts does not guarantee there are no aspects of the results needing attention. It is up to the individual to critically assess their own results and, if necessary, seek expert advice.

Publication of your CIF in IUCr journals

A basic structural check has been run on your CIF. These basic checks will be run on all CIFs submitted for publication in IUCr journals (*Acta Crystallographica*, *Journal of Applied Crystallography*, *Journal of Synchrotron Radiation*); however, if you intend to submit to *Acta Crystallographica Section C* or *E* or *IUCrData*, you should make sure that full publication checks are run on the final version of your CIF prior to submission.

Publication of your CIF in other journals

Please refer to the *Notes for Authors* of the relevant journal for any special instructions relating to CIF submission.

PLATON version of 04/06/2025; check.def file version of 30/05/2025

Datablock xiangshui12_Mo100K_auto - ellipsoid plot

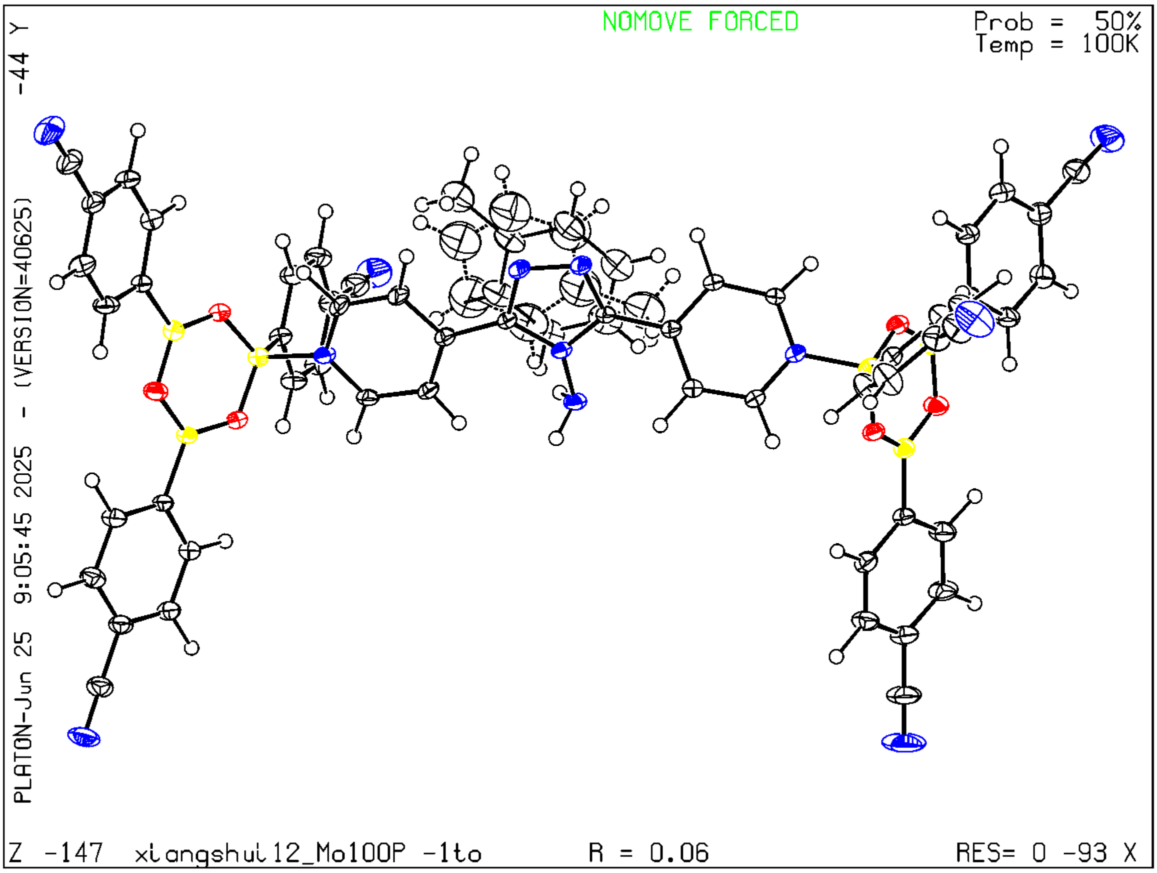

Supplement: Supplementary file 2 [file ANIE-65-e1406923-s001.zip › SC DATA/单晶/checkcif_BN-DPTA1.pdf]
