# Supplementary material for: Selective Adsorption of o‐Xylene From all C8 Aromatics via Secondary Connectivity Manipulation in B←N Frameworks
Source: Angew Chem Int Ed Engl. 2026 Aug 19;65(42):e1406923. doi: 10.1002/anie.1406923 (PMC13653566; doi:10.1002/anie.1406923)

checkCIF/PLATON report

Structure factors have been supplied for datablock(s) mo_20251218_hujieying_bm2

THIS REPORT IS FOR GUIDANCE ONLY. IF USED AS PART OF A REVIEW PROCEDURE FOR PUBLICATION, IT SHOULD NOT REPLACE THE EXPERTISE OF AN EXPERIENCED CRYSTALLOGRAPHIC REFEREE.

No syntax errors found.   CIF dictionary   Interpreting this report

Datablock: mo_20251218_hujieying_bm2

```
Bond precision:   C-C = 0.0032 A          Wavelength=0.71073

Cell:           a=13.3519(11)      b=13.8062(12)     c=17.9620(14)
                alpha=108.408(3)   beta=93.299(3)    gamma=104.464(3)
Temperature:    173 K

                 Calculated                   Reported
Volume           3008.4(4)                    3008.4(4)
Space group      P -1                         P -1
Hall group       -P 1                         -P 1
Moiety formula   C54 H34 B6 N12 O6, C8 H10    C54 H34 B6 N12 O6, C8 H10
Sum formula      C62 H44 B6 N12 O6            C62 H44 B6 N12 O6
Mr               1117.95                      1117.95
Dx,g cm-3        1.234                        1.234
Z                2                            2
Mu (mm-1)        0.080                        0.080
F000             1156.0                       1156.0
F000'            1156.44
h,k,lmax         15,16,21                     15,16,21
Nref             10603                        10576
Tmin,Tmax        0.988,0.993                  0.719,0.746
Tmin'            0.988

Correction method= # Reported T Limits: Tmin=0.719 Tmax=0.746
AbsCorr = MULTI-SCAN
```

```
Data completeness= 0.997            Theta(max)= 24.999

                                                          wR2(reflections)=
R(reflections)= 0.0504( 8241)                             0.1546( 10576)

S = 1.041                Npar= 834
```

The following ALERTS were generated. Each ALERT has the format
test-name_ALERT_alert-type_alert-level.
Click on the hyperlinks for more details of the test.

Alert level C

```
PLAT220_ALERT_2_C NonSolvent   Resd 1  N   Ueq(max)/Ueq(min) Range        4.0 Ratio
PLAT221_ALERT_2_C Solv./Anion  Resd 3  C   Ueq(max)/Ueq(min) Range        6.1 Ratio
PLAT223_ALERT_4_C Solv./Anion  Resd 3  H   Ueq(max)/Ueq(min) Range        4.8 Ratio
PLAT241_ALERT_2_C High  MainResAtom  Ueq as Compared to Neighbours         N7 Check
PLAT241_ALERT_2_C High  MainResAtom  Ueq as Compared to Neighbours        C52 Check
PLAT242_ALERT_2_C Low   MainResAtom  Ueq as Compared to Neighbours         N1 Check
PLAT242_ALERT_2_C Low   MainResAtom  Ueq as Compared to Neighbours        C44 Check
PLAT260_ALERT_2_C Large Average Ueq of Residue Including       C51      0.131 Check
PLAT910_ALERT_3_C Missing FCF Reflection(s) Below Theta(Min)[Deg]=       2.10 Note
                1  0  0,  -1  1  0,   0  1  0,   0 -1  1,  -1  0  1,   0  0  1,
PLAT911_ALERT_3_C Missing FCF Refl Between Thmin & STh/L=    0.595         21 Report
               -3 -4  1,  -1 -3  2,   0 -1  2,  -2  0  2,   1  1  2,   5  1  2,
                0-12  3,  -3 -9  3,   2 -1  3,   1  0  3,  -5  3  3,  -6  6  3,
               -3  9  3,   0 -3  4,   5 -1  4,   1  0  4,   5 -3  5,  -3 -9  6,
                0 -1  6,   3 -4  7,   0  3  7,
PLAT977_ALERT_2_C Check Negative Difference Density on H4A       .      -0.33 eA-3
```

Alert level G

```
PLAT002_ALERT_2_G Number of Distance or Angle Restraints on AtSite          6 Note
PLAT003_ALERT_2_G Number of Uiso or U(i,j)  Restrained non-H-Atoms         10 Report
PLAT007_ALERT_5_G Number of Unrefined Donor-H Atoms .............           2 Report
              H4A     H4B
PLAT154_ALERT_1_G The s.u.'s on the Cell Angles are Equal ..(Note)      0.003 Degree
PLAT171_ALERT_4_G The CIF-Embedded .res File Contains EADP Records          1 Report
PLAT172_ALERT_4_G The CIF-Embedded .res File Contains DFIX Records          4 Report
PLAT174_ALERT_4_G The CIF-Embedded .res File Contains FLAT Records          3 Report
PLAT178_ALERT_4_G The CIF-Embedded .res File Contains SIMU Records          1 Report
PLAT186_ALERT_4_G The CIF-Embedded .res File Contains ISOR Records          6 Report
PLAT187_ALERT_4_G The CIF-Embedded .res File Contains RIGU Records          2 Report
PLAT188_ALERT_3_G A Non-default SIMU Restraint Value has been used     0.0100 Report
PLAT190_ALERT_3_G A Non-default RIGU Restraint Value for First Par     0.0100 Report
PLAT190_ALERT_3_G A Non-default RIGU Restraint Value for SecondPar     0.0200 Report
PLAT190_ALERT_3_G A Non-default RIGU Restraint Value for First Par     0.0100 Report
PLAT190_ALERT_3_G A Non-default RIGU Restraint Value for SecondPar     0.0200 Report
PLAT231_ALERT_4_G Hirshfeld Test (Solvent)   C51      --C57      .        9.8 s.u.
PLAT231_ALERT_4_G Hirshfeld Test (Solvent)   C51      --C59      .       15.9 s.u.
PLAT231_ALERT_4_G Hirshfeld Test (Solvent)   C54      --C58      .       11.2 s.u.
PLAT231_ALERT_4_G Hirshfeld Test (Solvent)   C55      --C57      .        7.2 s.u.
PLAT231_ALERT_4_G Hirshfeld Test (Solvent)   C55      --C61      .        5.5 s.u.
PLAT231_ALERT_4_G Hirshfeld Test (Solvent)   C57      --C60      .        8.0 s.u.
PLAT231_ALERT_4_G Hirshfeld Test (Solvent)   C58      --C59      .       13.1 s.u.
```

```
PLAT300_ALERT_4_G Atom Site Occupancy of C51        Constrained at        0.9 Check
PLAT300_ALERT_4_G Atom Site Occupancy of C54        Constrained at        0.9 Check
PLAT300_ALERT_4_G Atom Site Occupancy of C55        Constrained at        0.9 Check
PLAT300_ALERT_4_G Atom Site Occupancy of C57        Constrained at        0.9 Check
PLAT300_ALERT_4_G Atom Site Occupancy of C58        Constrained at        0.9 Check
PLAT300_ALERT_4_G Atom Site Occupancy of C59        Constrained at        0.9 Check
PLAT300_ALERT_4_G Atom Site Occupancy of C60        Constrained at        0.9 Check
PLAT300_ALERT_4_G Atom Site Occupancy of C61        Constrained at        0.9 Check
PLAT300_ALERT_4_G Atom Site Occupancy of H51        Constrained at        0.9 Check
PLAT300_ALERT_4_G Atom Site Occupancy of H54        Constrained at        0.9 Check
PLAT300_ALERT_4_G Atom Site Occupancy of H58        Constrained at        0.9 Check
PLAT300_ALERT_4_G Atom Site Occupancy of H59        Constrained at        0.9 Check
PLAT300_ALERT_4_G Atom Site Occupancy of H60A       Constrained at        0.9 Check
PLAT300_ALERT_4_G Atom Site Occupancy of H60B       Constrained at        0.9 Check
PLAT300_ALERT_4_G Atom Site Occupancy of H60C       Constrained at        0.9 Check
PLAT300_ALERT_4_G Atom Site Occupancy of H61A       Constrained at        0.9 Check
PLAT300_ALERT_4_G Atom Site Occupancy of H61B       Constrained at        0.9 Check
PLAT300_ALERT_4_G Atom Site Occupancy of H61C       Constrained at        0.9 Check
PLAT300_ALERT_4_G Atom Site Occupancy of C63        Constrained at        0.1 Check
PLAT300_ALERT_4_G Atom Site Occupancy of C64        Constrained at        0.1 Check
PLAT300_ALERT_4_G Atom Site Occupancy of C65        Constrained at        0.1 Check
PLAT300_ALERT_4_G Atom Site Occupancy of C66        Constrained at        0.1 Check
PLAT300_ALERT_4_G Atom Site Occupancy of C67        Constrained at        0.1 Check
PLAT300_ALERT_4_G Atom Site Occupancy of C68        Constrained at        0.1 Check
PLAT300_ALERT_4_G Atom Site Occupancy of C69        Constrained at        0.1 Check
PLAT300_ALERT_4_G Atom Site Occupancy of C70        Constrained at        0.1 Check
PLAT300_ALERT_4_G Atom Site Occupancy of H64A       Constrained at        0.1 Check
PLAT300_ALERT_4_G Atom Site Occupancy of H64B       Constrained at        0.1 Check
PLAT300_ALERT_4_G Atom Site Occupancy of H64C       Constrained at        0.1 Check
PLAT300_ALERT_4_G Atom Site Occupancy of H65A       Constrained at        0.1 Check
PLAT300_ALERT_4_G Atom Site Occupancy of H65B       Constrained at        0.1 Check
PLAT300_ALERT_4_G Atom Site Occupancy of H65C       Constrained at        0.1 Check
PLAT300_ALERT_4_G Atom Site Occupancy of H67        Constrained at        0.1 Check
PLAT300_ALERT_4_G Atom Site Occupancy of H68        Constrained at        0.1 Check
PLAT300_ALERT_4_G Atom Site Occupancy of H69        Constrained at        0.1 Check
PLAT300_ALERT_4_G Atom Site Occupancy of H70        Constrained at        0.1 Check
PLAT302_ALERT_4_G Anion/Solvent/Minor-Residue Disorder (Resd    2)       100% Note
PLAT302_ALERT_4_G Anion/Solvent/Minor-Residue Disorder (Resd    3)       100% Note
PLAT304_ALERT_4_G Non-Integer Number of Atoms in ..... (Resd    2)      16.20 Check
PLAT304_ALERT_4_G Non-Integer Number of Atoms in ..... (Resd    3)       1.80 Check
PLAT335_ALERT_2_G Check Large C6 Ring C-C Range C51      -C59            0.26 Ang.
PLAT860_ALERT_3_G Number of Least-Squares Restraints ............         112 Note
PLAT909_ALERT_3_G Percentage of I>2sig(I) Data at Theta(Max) Still        52% Note
PLAT913_ALERT_3_G Missing # of Very Strong Reflections in FCF ....          2 Note
               -1 -3  2,   0 -3  4,
PLAT933_ALERT_2_G Number of HKL-OMIT Records in Embedded .res File         20 Note
               -6  6  3,  -5  3  3,  -3 -9  3,  -3 -9  6,  -3 -4  1,  -3  9  3,
               -2  0  2,  -1  0  1,  -1  1  0,   0-12  3,   0 -1  2,   0 -1  6,
                0  3  7,   1  0  3,   1  0  4,   1  1  2,   3 -4  7,   5 -3  5,
                5 -1  4,   5  1  2,
PLAT967_ALERT_5_G Note: Two-Theta Cutoff Value in Embedded .res ..       50.0 Degree
PLAT969_ALERT_5_G The 'Henn et al.' R-Factor-gap value ..........      6.483 Note
              Predicted wR2: Based on SigI**2  2.38 or SHELX Weight 14.85
PLAT978_ALERT_2_G Number C-C Bonds with Positive Residual Density.          2 Info
PLAT992_ALERT_5_G Repd & Actual _reflns_number_gt Values Differ by          2 Check
PLAT994_ALERT_1_G SHELXL .ins Contains no or MERG 0 Instruction ..          ! Note
```

```
 0 ALERT level A = Most likely a serious problem - resolve or explain
 0 ALERT level B = A potentially serious problem, consider carefully
11 ALERT level C = Check. Ensure it is not caused by an omission or oversight
72 ALERT level G = General information/check it is not something unexpected

 2 ALERT type 1 CIF construction/syntax error, inconsistent or missing data
13 ALERT type 2 Indicator that the structure model may be wrong or deficient
10 ALERT type 3 Indicator that the structure quality may be low
54 ALERT type 4 Improvement, methodology, query or suggestion
 4 ALERT type 5 Informative message, check
```

It is advisable to attempt to resolve as many as possible of the alerts in all categories. Often the minor alerts point to easily fixed oversights, errors and omissions in your CIF or refinement strategy, so attention to these fine details can be worthwhile. It is up to the individual to critically assess their own results and, if necessary, seek expert advice.

PLATON version of 15/01/2026; check.def file version of 02/01/2026

duplicate check

No duplication found

Datablock mo_20251218_hujieying_bm2 - ellipsoid plot

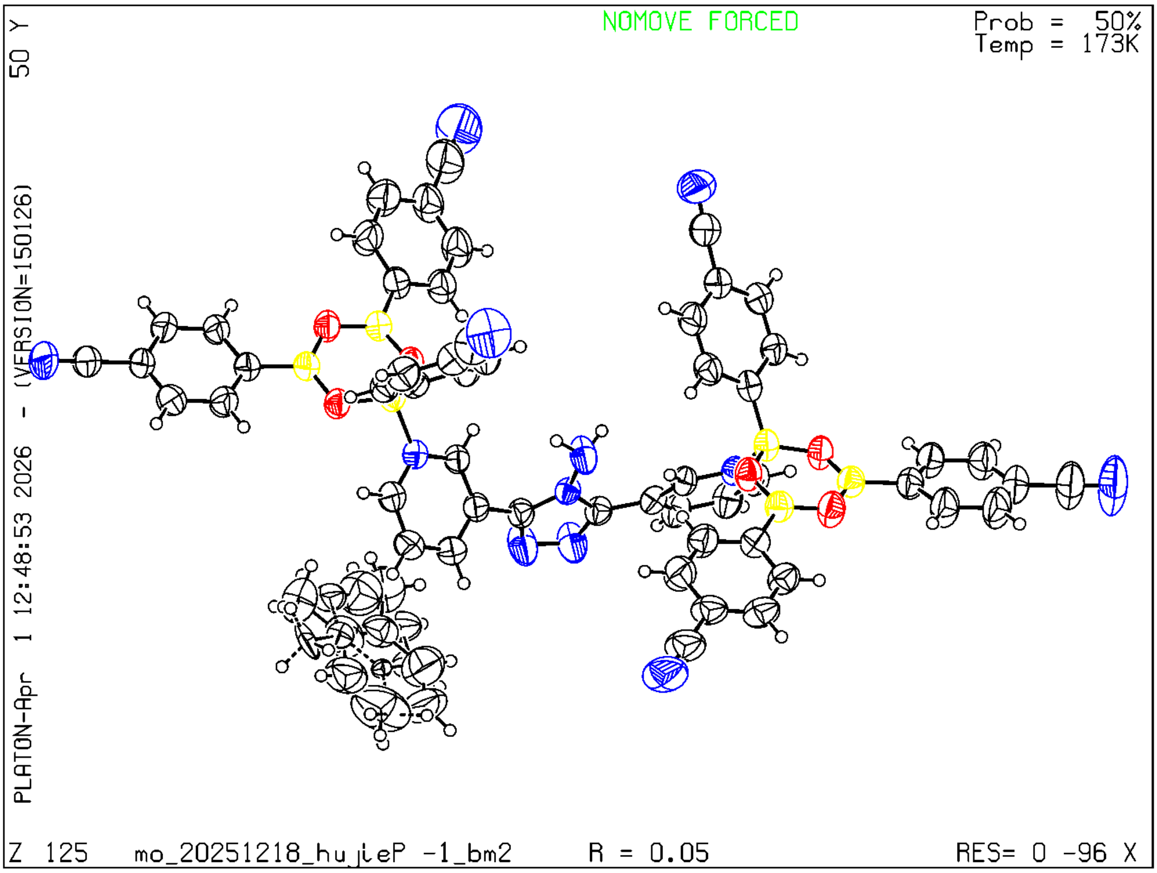

Supplement: Supplementary file 2 [file ANIE-65-e1406923-s001.zip › SC DATA/单晶/checkcif_BN-DPTA2-after soaking.pdf]
