# Supplementary material for: Selective Adsorption of o‐Xylene From all C8 Aromatics via Secondary Connectivity Manipulation in B←N Frameworks
Source: Angew Chem Int Ed Engl. 2026 Aug 19;65(42):e1406923. doi: 10.1002/anie.1406923 (PMC13653566; doi:10.1002/anie.1406923)

checkCIF/PLATON report

Structure factors have been supplied for datablock(s) xiangshuai24a

THIS REPORT IS FOR GUIDANCE ONLY. IF USED AS PART OF A REVIEW PROCEDURE FOR PUBLICATION, IT SHOULD NOT REPLACE THE EXPERTISE OF AN EXPERIENCED CRYSTALLOGRAPHIC REFEREE.

No syntax errors found. CIF dictionary Interpreting this report

Datablock: xiangshuai24a

Bond precision: C-C = 0.0033 A Wavelength=0.71073

Cell:	a=13.2726(6)	b=13.6408(6)	c=17.7273(8)
	alpha=107.466(4)	beta=93.284(4)	gamma=104.389(4)
Temperature:	100 K		

	Calculated	Reported
Volume	2935.1(2)	2935.1(3)
Space group	P -1	P -1
Hall group	-P 1	-P 1
Moiety formula	C54 H34 B6 N12 O6, C8 H10	C54 H34 B6 N12 O6, C8 H10
Sum formula	C62 H44 B6 N12 O6	C62 H44 B6 N12 O6
Mr	1117.95	1118.06
Dx,g cm-3	1.265	1.265
Z	2	2
Mu (mm-1)	0.082	0.082
F000	1156.0	1156.7
F000'	1156.44	
h,k,lmax	16,16,21	16,16,21
Nref	11555	11432
Tmin,Tmax	0.995,0.998	0.327,1.000
Tmin'	0.988	

Correction method= # Reported T Limits: Tmin=0.327 Tmax=1.000
AbsCorr = MULTI-SCAN

```
Data completeness= 0.989            Theta(max)= 26.000

                                                          wR2(reflections)=
R(reflections)= 0.0579( 6931)                             0.0797( 11432)

S = 1.002                 Npar= 907
```

```
The following ALERTS were generated. Each ALERT has the format
       test-name_ALERT_alert-type_alert-level.
Click on the hyperlinks for more details of the test.
```

Alert level C

```
DIFMN02_ALERT_2_C  The minimum difference density is < -0.1*ZMAX*0.75
            _refine_diff_density_min given =     -0.603
            Test value =     -0.600
DIFMN03_ALERT_1_C  The minimum difference density is < -0.1*ZMAX*0.75
            The relevant atom site should be identified.
DIFMX02_ALERT_1_C  The maximum difference density is > 0.1*ZMAX*0.75
            The relevant atom site should be identified.
PLAT097_ALERT_2_C Large Reported Max.  (Positive) Residual Density       0.62 eA-3
PLAT213_ALERT_2_C Atom N9              has ADP max/min Ratio .....        3.2 prolat
PLAT220_ALERT_2_C NonSolvent   Resd 1  C   Ueq(max)/Ueq(min) Range        3.3 Ratio
PLAT220_ALERT_2_C NonSolvent   Resd 1  N   Ueq(max)/Ueq(min) Range        5.2 Ratio
PLAT906_ALERT_3_C Large K Value in the Analysis of Variance ......     16.704 Check
PLAT906_ALERT_3_C Large K Value in the Analysis of Variance ......      2.904 Check
PLAT910_ALERT_3_C Missing FCF Reflection(s) Below Theta(Min)[Deg]=       2.12 Note
                1  0  0,  -1  1  0,   0  1  0,   0 -1  1,  -1  0  1,   0  0  1,
PLAT911_ALERT_3_C Missing FCF Refl Between Thmin & STh/L=    0.600         70 Report
                1  1  0,   0 -2  1,   0  1  1,   0 -1  2,   1 -1  2,  -1  0  2,
                0  0  2,  -3  9  3,  -3 -9  6,  -7  1  6,  -4 -7  7,  -4 -6  7,
               -2-12  8,  -3-11  8,  -3-10  8,  -7 12  8,  -2-14 10,  -3-13 10,
               -7 11 10,  -6 11 10,  -5 11 10,  -4 11 10,  -1-15 11,   0-15 11,
               -3-14 11,  -3-13 11,   7  6 11,   5  7 11,  -2  9 11,  -1  9 11,
                   (   40 More Missing: see the .ckf listing file)
```

Alert level G

```
PLAT068_ALERT_1_G Reported F000 Differs from Calcd (or Missing)...     Please Check
PLAT073_ALERT_1_G H-atoms ref., but hydrogen treatment Reported as     constr Check
PLAT154_ALERT_1_G The s.u.'s on the Cell Angles are Equal ..(Note)      0.004 Degree
PLAT720_ALERT_4_G Number of Unusual/Non-Standard Labels .........           6 Note
              H7SA    H7SB    H7SC    H8SA    H8SB    H8SC
PLAT790_ALERT_4_G Centre of Gravity not Within Unit Cell: Resd.  #          2 Note
              C8 H10
PLAT802_ALERT_4_G CIF Input Record(s) with more than 80 Characters          1 Info
PLAT912_ALERT_4_G Missing # of FCF Reflections Above STh/L=  0.600         47 Note
PLAT933_ALERT_2_G Number of HKL-OMIT Records in Embedded .res File         10 Note
               -7  1  6,  -3 -9  6,  -3  9  3,  -1  0  2,  -1  1  0,   0 -1  2,
                0  0  2,   0  1  1,   1 -1  2,   1  1  0,
PLAT941_ALERT_3_G Average HKL Measurement Multiplicity ..........         1.7 Low
PLAT961_ALERT_5_G Dataset Contains no Negative Intensities ......     Please Check
PLAT967_ALERT_5_G Note: Two-Theta Cutoff Value in Embedded .res ..       52.0 Degree
PLAT969_ALERT_5_G The 'Henn et al.' R-Factor-gap value ..........      1.046 Note
              Predicted wR2: Based on SigI**2  7.62 or SHELX Weight  7.95
```

```
PLAT978_ALERT_2_G Number C-C Bonds with Positive Residual Density.         15 Info
PLAT979_ALERT_1_G NoSpherA2 Scattering Factors Used .............     Please Note
```

```
  0 ALERT level A = Most likely a serious problem - resolve or explain
  0 ALERT level B = A potentially serious problem, consider carefully
 11 ALERT level C = Check. Ensure it is not caused by an omission or oversight
 14 ALERT level G = General information/check it is not something unexpected

  6 ALERT type 1 CIF construction/syntax error, inconsistent or missing data
  7 ALERT type 2 Indicator that the structure model may be wrong or deficient
  5 ALERT type 3 Indicator that the structure quality may be low
  4 ALERT type 4 Improvement, methodology, query or suggestion
  3 ALERT type 5 Informative message, check
```

It is advisable to attempt to resolve as many as possible of the alerts in all categories. Often the minor alerts point to easily fixed oversights, errors and omissions in your CIF or refinement strategy, so attention to these fine details can be worthwhile. It is up to the individual to critically assess their own results and, if necessary, seek expert advice.

PLATON version of 04/06/2025; check.def file version of 30/05/2025

---

duplicate check

No duplication found

Datablock xiangshuai24a - ellipsoid plot

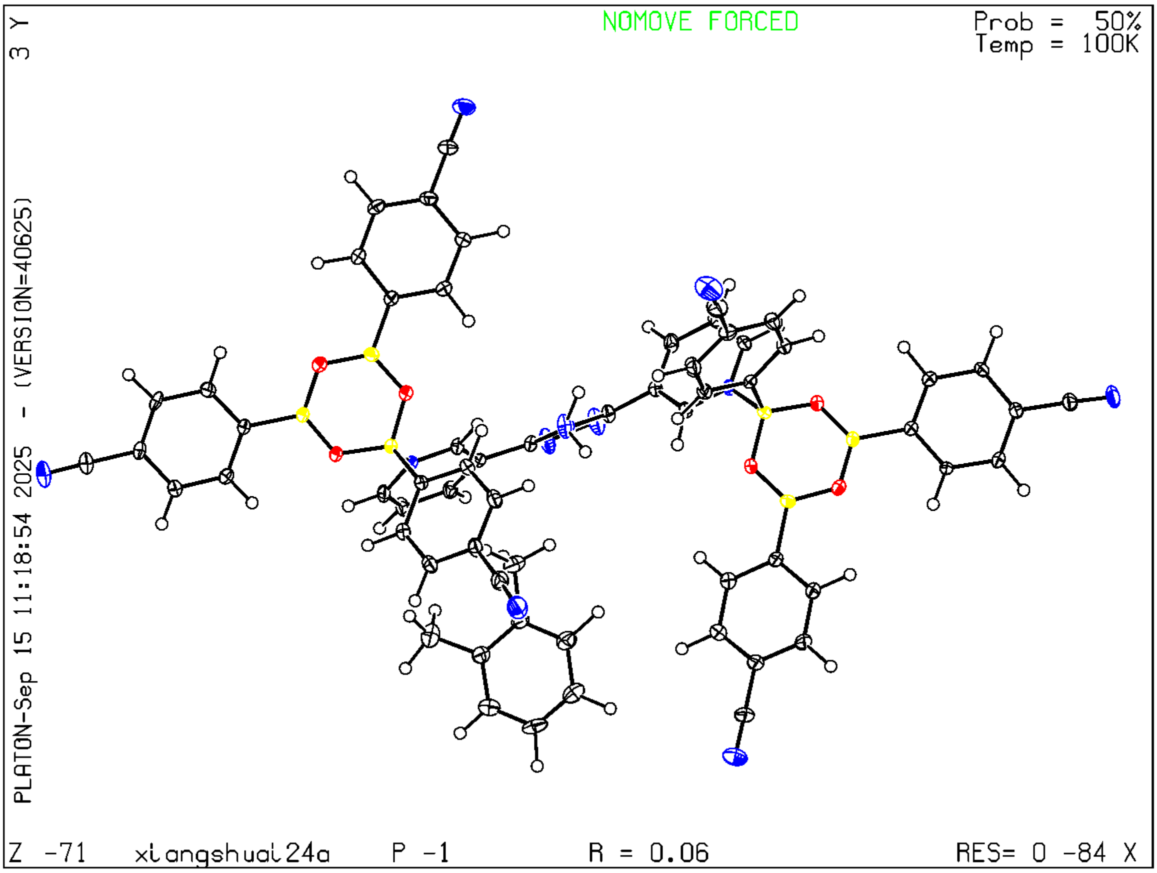

Supplement: Supplementary file 2 [file ANIE-65-e1406923-s001.zip › SC DATA/单晶/checkcif_BN-DPTA2.pdf]
