# Supplementary material for: Selective Adsorption of o‐Xylene From all C8 Aromatics via Secondary Connectivity Manipulation in B←N Frameworks
Source: Angew Chem Int Ed Engl. 2026 Aug 19;65(42):e1406923. doi: 10.1002/anie.1406923 (PMC13653566; doi:10.1002/anie.1406923)

checkCIF/PLATON report

Structure factors have been supplied for datablock(s) xiangshuai23_mo100k_auto

THIS REPORT IS FOR GUIDANCE ONLY. IF USED AS PART OF A REVIEW PROCEDURE FOR PUBLICATION, IT SHOULD NOT REPLACE THE EXPERTISE OF AN EXPERIENCED CRYSTALLOGRAPHIC REFEREE.

No syntax errors found. CIF dictionary Interpreting this report

Datablock: xiangshuai23_mo100k_auto

```
Bond precision:   C-C = 0.0049 A          Wavelength=0.71073

Cell:            a=16.1375(5)     b=24.7146(7)       c=22.1396(7)
                 alpha=90         beta=110.179(4)    gamma=90
Temperature:     100 K
```

	Calculated	Reported
Volume	8288.0(5)	8288.0(5)
Space group	I 2/a	I 1 2/a 1
Hall group	-I 2ya	-I 2ya
Moiety formula	C88 H68 B6 N10 Ni O8 [+ solvent]	C88 H68 B6 N10 Ni O8, 1.334[C7H8]
Sum formula	C88 H68 B6 N10 Ni O8 [+ solvent]	C97.34 H78.67 B6 N10 Ni O8
Mr	1517.07	1640.02
Dx,g cm-3	1.216	1.314
Z	4	4
Mu (mm-1)	0.295	0.301
F000	3152.0	3419.0
F000’	3154.52	
h,k,lmax	19,29,26	19,29,26
Nref	7713	7705
Tmin,Tmax	0.965,0.982	0.394,1.000
Tmin’	0.914	

Correction method= # Reported T Limits: Tmin=0.394 Tmax=1.000
AbsCorr = MULTI-SCAN

Data completeness= 0.999 Theta(max)= 25.499

R(reflections)= 0.0646(5994) wR2(reflections)= 0.1432(7705)

S = 1.093 Npar= 577

The following ALERTS were generated. Each ALERT has the format
test-name_ALERT_alert-type_alert-level.
Click on the hyperlinks for more details of the test.

Alert level C

```
PLAT220_ALERT_2_C NonSolvent   Resd 1  C   Ueq(max)/Ueq(min) Range        3.2 Ratio
PLAT220_ALERT_2_C NonSolvent   Resd 1  N   Ueq(max)/Ueq(min) Range        3.5 Ratio
PLAT410_ALERT_2_C Short Intra H...H Contact  H5       ..H15       .       1.99 Ang.
                                                      x,y,z  =      1_555 Check
PLAT906_ALERT_3_C Large K Value in the Analysis of Variance ......      5.086 Check
PLAT911_ALERT_3_C Missing FCF Refl Between Thmin & STh/L=    0.600          6 Report
                0  6  0,  -1  2  1,  -1  1  2,  -1  1  8,  -1  6 11, -18  0 16,
```

Alert level G

```
FORMU01_ALERT_2_G  There is a discrepancy between the atom counts in the
            _chemical_formula_sum and the formula from the _atom_site* data.
            Atom count from _chemical_formula_sum:C97.34 H78.67 B6 N10 Ni1 O8
            Atom count from the _atom_site data:  C88 H68 B6 N10 Ni1 O8
CELLZ01_ALERT_1_G Difference between formula and atom_site contents detected.
CELLZ01_ALERT_1_G ALERT: Large difference may be due to a
            symmetry error - see SYMMG tests
           From the CIF: _cell_formula_units_Z    4
           From the CIF: _chemical_formula_sum  C97.34 H78.67 B6 N10 Ni O8
           TEST: Compare cell contents of formula and atom_site data

           atom    Z*formula  cif sites diff
           C        389.36    352.00   37.36
           H        314.68    272.00   42.68
           B         24.00     24.00    0.00
           N         40.00     40.00    0.00
           Ni         4.00      4.00    0.00
           O         32.00     32.00    0.00
PLAT002_ALERT_2_G Number of Distance or Angle Restraints on AtSite         15 Note
PLAT003_ALERT_2_G Number of Uiso or U(i,j)  Restrained non-H-Atoms         14 Report
PLAT041_ALERT_1_G Calc. and Reported SumFormula    Strings  Differ     Please Check
             Calc: C88 H68 B6 N10 Ni O8
             Rep.: C97.34 H78.67 B6 N10 Ni O8
PLAT042_ALERT_1_G Calc. and Reported MoietyFormula Strings  Differ     Please Check
             Calc: C88 H68 B6 N10 Ni O8
             Rep.: C88 H68 B6 N10 Ni O8, 1.334[C7H8]
PLAT083_ALERT_2_G SHELXL Second Parameter in WGHT  Unusually Large      29.02 Why ?
PLAT174_ALERT_4_G The CIF-Embedded .res File Contains FLAT Records          2 Report
```

```
PLAT176_ALERT_4_G The CIF-Embedded .res File Contains SADI Records         21 Report
PLAT186_ALERT_4_G The CIF-Embedded .res File Contains ISOR Records          4 Report
PLAT187_ALERT_4_G The CIF-Embedded .res File Contains RIGU Records          4 Report
PLAT190_ALERT_3_G A Non-default RIGU Restraint Value for First Par     0.0010 Report
PLAT190_ALERT_3_G A Non-default RIGU Restraint Value for SecondPar     0.0010 Report
PLAT190_ALERT_3_G A Non-default RIGU Restraint Value for First Par     0.0010 Report
PLAT190_ALERT_3_G A Non-default RIGU Restraint Value for SecondPar     0.0010 Report
PLAT190_ALERT_3_G A Non-default RIGU Restraint Value for First Par     0.0010 Report
PLAT190_ALERT_3_G A Non-default RIGU Restraint Value for SecondPar     0.0010 Report
PLAT190_ALERT_3_G A Non-default RIGU Restraint Value for First Par     0.0010 Report
PLAT190_ALERT_3_G A Non-default RIGU Restraint Value for SecondPar     0.0010 Report
PLAT191_ALERT_3_G A Non-default SADI Restraint Value has been used     0.0400 Report
PLAT191_ALERT_3_G A Non-default SADI Restraint Value has been used     0.0400 Report
PLAT191_ALERT_3_G A Non-default SADI Restraint Value has been used     0.0400 Report
PLAT191_ALERT_3_G A Non-default SADI Restraint Value has been used     0.0400 Report
PLAT191_ALERT_3_G A Non-default SADI Restraint Value has been used     0.0400 Report
PLAT191_ALERT_3_G A Non-default SADI Restraint Value has been used     0.0400 Report
PLAT191_ALERT_3_G A Non-default SADI Restraint Value has been used     0.0400 Report
PLAT191_ALERT_3_G A Non-default SADI Restraint Value has been used     0.0400 Report
PLAT191_ALERT_3_G A Non-default SADI Restraint Value has been used     0.0400 Report
PLAT191_ALERT_3_G A Non-default SADI Restraint Value has been used     0.0400 Report
PLAT301_ALERT_3_G Main Residue  Disorder ..............(Resd    1)        12% Note
PLAT605_ALERT_4_G Largest Solvent Accessible VOID in the Structure        301 A**3
PLAT793_ALERT_4_G Model has Chirality at C17         (Centro SpGr)          R Verify
PLAT860_ALERT_3_G Number of Least-Squares Restraints .............        261 Note
PLAT868_ALERT_4_G ALERTS Due to the Use of _smtbx_masks Suppressed          ! Info
PLAT910_ALERT_3_G Missing FCF Reflection(s) Below Theta(Min)[Deg]=       1.96 Note
                1  1  0,   0  2  0,   0  1  1,
PLAT933_ALERT_2_G Number of HKL-OMIT Records in Embedded .res File          5 Note
               -1  1  2,  -1  1  8,  -1  2  1,  -1  6 11,   0  6  0,
PLAT941_ALERT_3_G Average HKL Measurement Multiplicity ...........        3.0 Low
PLAT961_ALERT_5_G Dataset Contains no Negative Intensities .......     Please Check
PLAT967_ALERT_5_G Note: Two-Theta Cutoff Value in Embedded .res ..       51.0 Degree
PLAT969_ALERT_5_G The 'Henn et al.' R-Factor-gap value ...........      3.201 Note
              Predicted wR2: Based on SigI**2  4.47 or SHELX Weight 13.10 Note
PLAT978_ALERT_2_G Number C-C Bonds with Positive Residual Density.          1 Info
```

```
   0 ALERT level A = Most likely a serious problem - resolve or explain
   0 ALERT level B = A potentially serious problem, consider carefully
   5 ALERT level C = Check. Ensure it is not caused by an omission or oversight
  42 ALERT level G = General information/check it is not something unexpected

   4 ALERT type 1 CIF construction/syntax error, inconsistent or missing data
   9 ALERT type 2 Indicator that the structure model may be wrong or deficient
  24 ALERT type 3 Indicator that the structure quality may be low
   7 ALERT type 4 Improvement, methodology, query or suggestion
   3 ALERT type 5 Informative message, check
```

It is advisable to attempt to resolve as many as possible of the alerts in all categories. Often the minor alerts point to easily fixed oversights, errors and omissions in your CIF or refinement strategy, so attention to these fine details can be worthwhile. It is up to the individual to critically assess their own results and, if necessary, seek expert advice.

PLATON version of 23/04/2026; check.def file version of 30/03/2026

duplicate check

No duplication found

Datablock xiangshuai23_mo100k_auto - ellipsoid plot

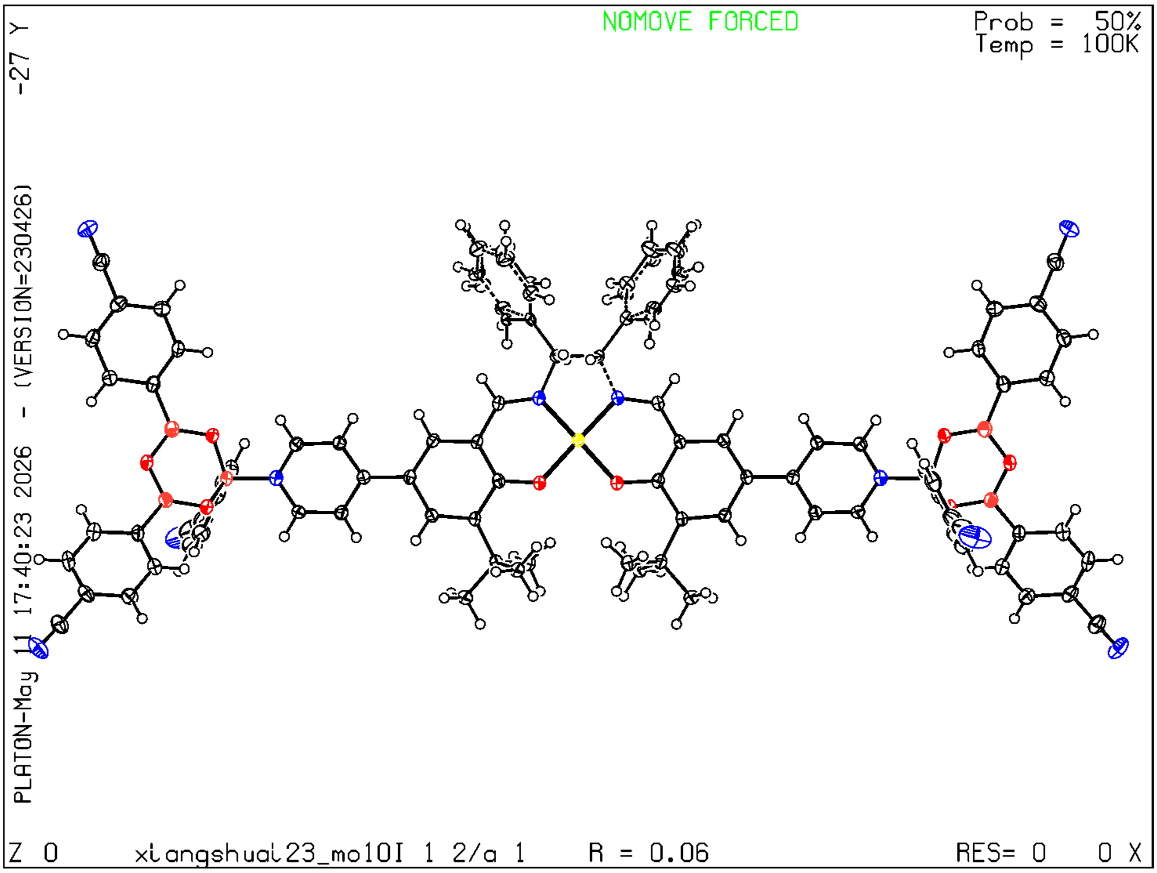

Supplement: Supplementary file 2 [file ANIE-65-e1406923-s001.zip › SC DATA/单晶/checkcif_BN-Salen-Ni.pdf]
